# Offspring thermal demands and parental brooding efficiency differ for precocial birds living in contrasting climates

**DOI:** 10.1186/s12983-023-00492-1

**Published:** 2023-04-10

**Authors:** Veronika Kolešková, Miroslav E. Šálek, Kateřina Brynychová, Petr Chajma, Lucie Pešková, Esmat Elhassan, Eva Petrusová Vozabulová, Veronika Janatová, Aisha Almuhery, Martin Sládeček

**Affiliations:** 1 Faculty of Environmental Sciences, Czech University of Life Sciences Prague, Kamýcká 129, 165 00 Prague, Czech Republic; 2 Natural Resources Conservation Section, Environment Department, Dubai Municipality, Abu Hail, Dubai, UAE

**Keywords:** Brooding, Shorebirds, Accelerometer, Multisensory datalogger, Hidden Markov models

## Abstract

**Background:**

Chicks of precocial birds hatch well-developed and can search actively for food but their homeothermy develops gradually during growth. This makes them dependent on heat provided by parents (“brooding”), which is then traded off against other activities, mainly foraging. Although brooding has been documented in many precocial birds, little is known about the differences in the amount and efficiency of brooding care, brooding diel rhythmicity, and impact on the chick’s growth, particularly between species living in different climatic conditions.

**Results:**

We used multisensory dataloggers to evaluate brooding patterns in two congeneric species inhabiting contrasting climate zones: temperate Northern lapwing (*Vanellus vanellus*) and desert Red-wattled lapwing (*Vanellus indicus*). In accordance with our expectation, the adult desert lapwings brooded the chicks slightly less compared to the adult temperate lapwings. However, the desert lapwings brooded their chicks in higher ambient temperatures and less efficiently (i.e. they could not reach the same brooding temperature as the temperate lapwings), which are new and hitherto unknown brooding patterns in precocial birds. In both species, night brooding prevailed even during warm nights, suggesting a general brooding rule among birds. Although the high rates of brooding can reduce the time spent by foraging, we found no negative effect of the high brooding rate on the growth rate in either species.

**Conclusions:**

Our data suggest that the chicks of species breeding in colder climates may reduce their thermal demands, while their parents may increase the efficiency of parental brooding care. More research is however needed to confirm this as a rule across species.

**Supplementary Information:**

The online version contains supplementary material available at 10.1186/s12983-023-00492-1.

## Background

The maintenance of a constant body temperature (i.e. homeothermy) is essential for the survival of most endotherms [1, 2], providing high muscle performance, rapid growth, and independence from the ambient temperature [1]. However, maintaining homeothermy is energy-intensive [1, 3]. Therefore, during early life, the energy needed to maintain homeothermy might be traded off against energetic demands on growth [4, 5]. In endotherms, young are usually born before they fully achieve homeothermy, and in the early stages of life they are often dependent on the additional thermoregulation provided by their parents [6–8].

An interesting group of animals in this context are the chicks of precocial birds. Although they are active in foraging and in escaping from predators after hatching [9, 10], their full homeothermy develops gradually during growth before fledging [8, 9, 11, 12]. An essential component of parental care is therefore the provision of supplemental heat, so-called ‘brooding’. This is realised through the chicks being hidden in their parent’s feathers and being covered by the parent’s abdomen [13].

In general, most of the daily activity of precocial chicks consists of alternating bouts of foraging and parental brooding [11, 14, 15]. The time spent on being brooded decreases with the age of the chicks in correspondence with the increasing degree of homeothermy [8, 9, 16], but increases in lower ambient temperatures [11, 16, 17]. As shown by Beintema and Visser [11] on several temperate meadow shorebird species, the proportion of time that parents spend brooding their chicks under cold weather conditions may rise to more than 80% of the daylight time. This may severely constrain the foraging time that is necessary for maintaining body weight and growth [11].

Although brooding as a phenomenon has been documented in a variety of precocial species [11, 15, 18–21], detailed knowledge about how ambient temperature modulates brooding patterns for the chicks of different ages throughout the day is scarce. Particularly rare are the studies based on continuous monitoring of the whole 24-h cycle, which can provide robust data unbiased by the uneven sampling effort in the observational studies. While the brooding patterns are predictable in altricial birds who are generally known to spend most of the night-time sitting on the nest, [22, 23] this might be an important knowledge gap in precocial birds, who often use the night-time for foraging [24–26]. The only available data suggest that brooding may clearly prevail during the night even in precocial shorebirds, but are constraint only to the arctic areas [9, 27]. Because of the seasonal and latitudinal night-time length variation, the lack of night-time data in non-arctic regions may have led to published brooding rates that underestimate the true rate of brooding activity. In addition, observations not including night-time disable relevant comparisons of brooding behaviour between (e.g. related) populations living at different latitudes. Finally, to the best of our knowledge, there has been no study quantifying the thermal efficiency of brooding.

Because brooding is closely linked to thermoregulation and energy expenditure, an important unanswered question concerns how brooding patterns differ between related species inhabiting completely different environments, for example, temperate regions and hot (tropical) regions. Species in a warmer climate tend to have a slower lifestyle than species living in higher latitudes with a colder climate [28–30]. The slower lifestyle, associated among others with a less efficient metabolism [29], is very likely also to affect brooding patterns. Therefore, different life histories in related species breeding in different climates may also be translated into the brooding, a key component of parental care in precocial species, which remains a poorly studied life history trait in birds.

In our study, we used miniaturised multisensory dataloggers with continuous recording to reveal patterns of parental brooding, its efficiency, and its impact on chick growth. We studied two related species of precocial shorebirds breeding in highly contrasting climatic conditions and pursuing different life-histories. Specifically, we selected two lapwings from the family Charadriidae: the Northern lapwing (*Vanellus vanellus*), breeding in a temperate climate, and the Red-wattled lapwing (*Vanellus indicus*), from a hot desert climate. Compared to the Red-wattled lapwing, the Northern lapwing exhibits higher reproductive effort (relative clutch volume; own data), and faster chick growth [31], indicating a more efficient metabolism [28, 29].

We tested the following predictions. First, in both species, we predicted that the time spent on being brooded (i.e. the “brooding rate”) is strongly modulated by diel timing, ambient temperature and by the chick’s age, with increased effort during the night, in lower ambient temperatures, and in younger chicks. Second, we expected the overall brooding rate to be significantly lower in desert species, where the chicks spend less time in low ambient temperatures. Alternatively, this difference may be mitigated by the shift in the thermal comfort zone of the desert species inhabiting environments with generally higher temperatures [32]. We also assumed that the parents of the temperate species inhabiting an environment in which it is necessary to withstand the cold are able to maintain higher brooding temperatures under similar ambient conditions (i.e., will brood the chicks more efficiently). Finally, we predicted that the brooding rates in both species are negatively correlated with the growth rate of the chicks during the observational period, assuming that higher brooding rates may limit foraging time.

## Methods

### Study area and populations

The study was conducted on two different sites. Northern lapwing (NL) was investigated in the České Budějovice basin, South Bohemia, Czech Republic (48.99°N 14.38°E). The study area (~ 40 km^2^) consisted of a mosaic of arable land, meadows, fishponds, forests, and human settlements. A population of ~ 100 pairs of the model species inhabited various habitats on arable land and meadows [33, 34].

Red-wattled lapwing (RWL) was investigated in the Al Marmoom Desert Conservation Reserve, located about 30 km from Dubai, United Arab Emirates (24.83°N 55.36°E). The study area (6.63 km^2^) consisted of a mixture of desert, artificial lakes supplied with desalinated water from the sea, and sparse tree plantations [31, 35]. The study area was inhabited by a population of ~ 80 pairs of the model species.

## Data collection

Fieldwork took place during breeding seasons 2019 and 2020, between March and June. We applied the same field protocol in both localities. We searched for families of both model species with chicks by carefully scanning the area with binoculars on foot or from a car. Then we captured the chicks by hand, ringed them with a metal ring, weighed them to the nearest 0.1 g with an electronic scale, and took the following measurements using a calliper with accuracy to the nearest 0.1 mm: the bill length (“BNprox”), the length of the head (“HL”) and the length of the tarsus (“Tar1”) (following [36]). For the analyses, we used the head length as a proxy for the chick age, because it performs best from all size measurements. For the dataset of 514 measurements of chicks with known age taken for another project, the relationship between the age and the head length was perfectly linear at the age range found in this study, with the R^2^ = 0.88.

To monitor the activity and behaviour, we randomly selected 1–3 chicks from each family (depending on the size of the family) and equipped each with a multisensory datalogger (weight: ~ 1.1 g; size: 20.6 × 19.0 mm), developed by the research team (DAL 2, Additional file 2: Picture S3). The chicks without dataloggers were subsequently used a control group to check the effect of datalogger deployment on growth parameters. The logger can measure temperature, humidity, light level, three-axial acceleration, and three-axial magnetometry at the same time, with adjustable frequencies, for approximately 72 h of continuous recording. For the purposes of our study, we used temperature (1 Hz), light-level (1 Hz), and three-axial accelerometry (25 Hz) measurements. We set 14 g as the minimum chick weight for the installation of the datalogger. Although the load in such a case was more than the usually recommended 3–5% [37], the deployment of the logger did not reduce the growth rate, compared with the chicks’ siblings without the logger (wilcox.test, *p* value = 0.99, n = 217).

We attached the datalogger with a small drop of superglue to the down feathers in the lower back, with a light-level sensor oriented towards the head (Additional file 2: Picture S4). We made every effort to place the device in the same position for all individuals. We recaptured the chicks and collected the datalogger after 24–72 h, preferentially first collecting the dataloggers from the smaller chicks. We then reweighed the chicks and released them. In one case (1%), we found only the datalogger that dropped from the chick. In two cases (2%), we found the datalogger together with the remains of the depredated chick.

## Sample sizes

Overall, 38 chicks from 26 families of Northern lapwings (20 in 2019 and 18 in 2020), and 65 chicks from 42 families of Red-wattled lapwings (62 in 2019 and 4 in 2020) were monitored. In total, 4084 h of recordings from dataloggers were obtained (1327 for Northern lapwings and 2824 for Red-wattled lapwings). The chicks of the Northern lapwings were monitored for 27.5 h (median, range 14–73 h), while the chicks of the Red-wattled lapwings were monitored for 42.6 h (median, range 19.1–92.9 h). To assess the brooding efficiency (see below), 4946 brooding bouts longer than 5 min were assessed (1693 for Northern lapwings and 3253 for Red-wattled lapwings).

## Data preparation

We performed all data processing, statistical analyses, and visualizations in R 3.5.3. [38]. We pre-processed the accelerometry data in several steps (see supplementary script: 45). Initially, to minimize the calibration error of the accelerometer sensor, we used an iterative closest-point fitting autocalibration process, as recommended by van Hees et al. [39]. To remove the gravitational part of the acceleration, we smoothed the accelerometric measurements in all three axes by a median filter with a cut-off frequency of 0.2 Hz and subtracted the smoothed measurements from the raw measurements (i.e. High-pass filter). To eliminate the random noise present in all sensory data, we further smoothed the data by moving the median filter with a cut-off frequency of 2 Hz (i.e. Low-pass filter) [40]. We then calculated the overall dynamic body acceleration (ODBA) [41] representing the rate of aggregated acceleration of an individual by summing the absolute values of the dynamic parts of the acceleration of all axes. To reduce the computational intensity in subsequent data processing, we aggregated the ODBA, temperature and light-level measurements by averaging over periods of 5 s. Finally, to ensure that only natural behaviour without observer disturbance was analysed, we cut the first and the last 30 min from each dataset.

## Detection of brooding

**Fig. 1:**
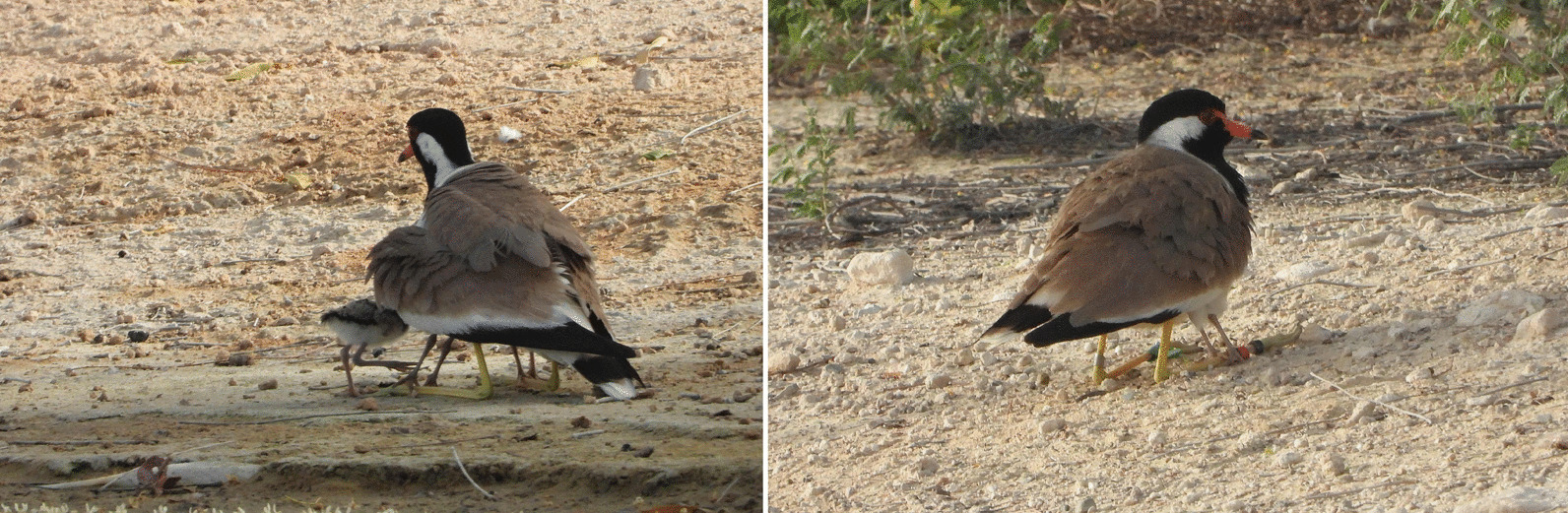
Illustration of parental brooding of small chicks of the Red-wattled lapwing during daylight

**Fig. 2:**
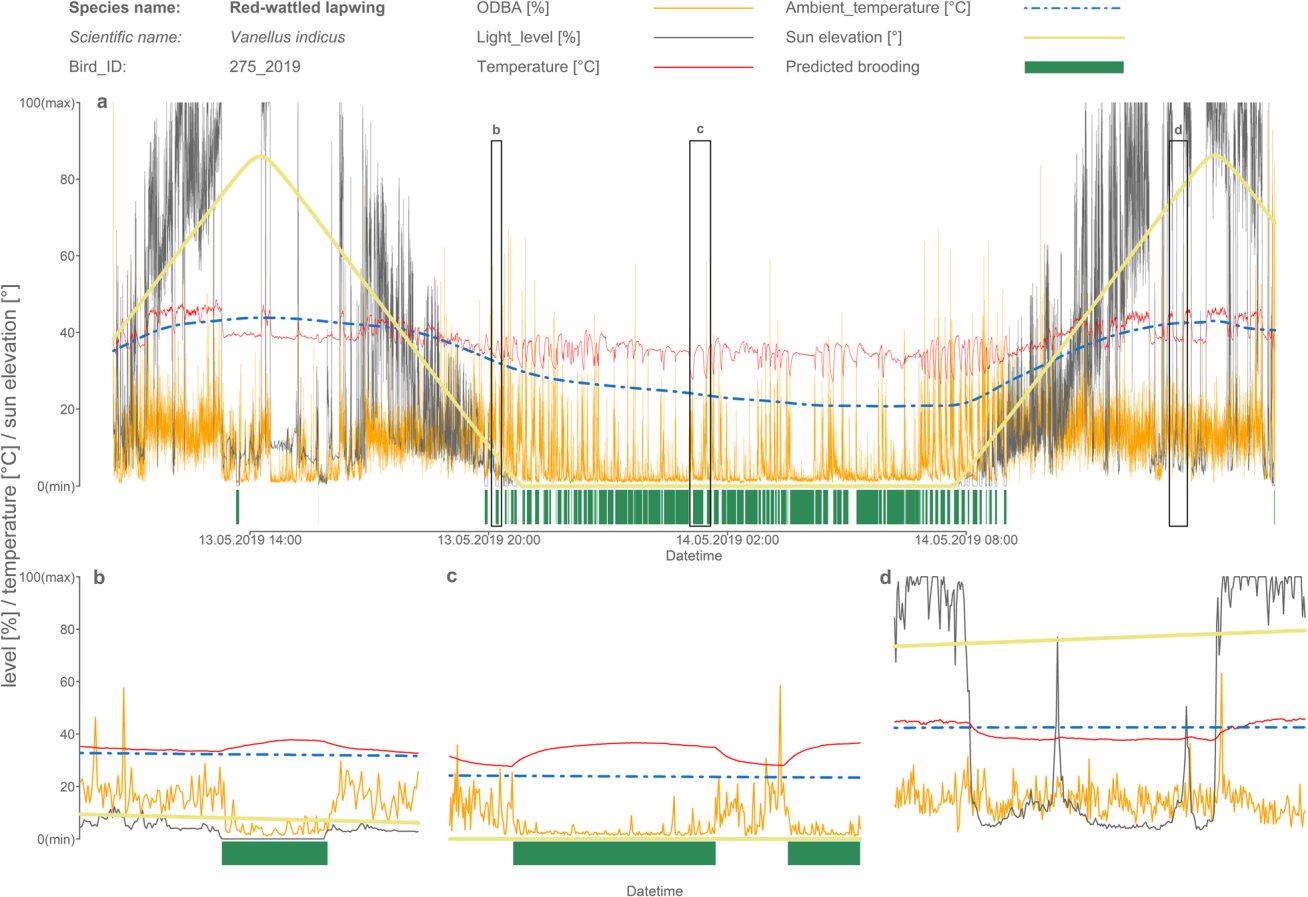
**a** Sample actogram showing the data taken from one Red-wattled lapwing chick. The orange line represents the activity of the chick expressed as ODBA, the blue dashed line represents the ambient temperature, the red line the on-body temperature measured by the datalogger on the chick’s dorsal part, the black line the light-level, and the yellow line the sun elevation. The green polygons below represents predicted brooding. **b**–**d** are zoomed parts of original actogram highlighted by black frames. **b** shows the typical brooding bout during the daylight—see the low activity level, together with the increasing on-body temperature and close to zero light-level. **c** shows the typical brooding bout during the night—see the low activity level, together with the increasing on-body temperature; light-level is during the night constantly close to zero. **d** shows the typical pattern connected to the stay in the shade. Although there is visible temperature drop from very high temperatures (i.e. much above the birds body temperature), this is not associated with the drop in the activity level. Moreover, light-level is much higher than expected during the brooding and is in the range typical for stay in the shade of plants

We defined brooding as a state when the chick is hidden under the parent with its dorsal part pressed against the parent’s abdomen (Fig. 1). Within such behaviour, there are several typical patterns observable in the data from the selected sensor combination which we used to determine when the parent actively brooded the chicks (Fig. 2). First, during the brooding, chick activity (expressed as ODBA) is generally low. Second, the temperature measured on the dorsal part of a chick tends to converge to the parent’s body temperature level (~ 40 °C: [8, 42]), which was previously used as a sole indicator of the brooding in a different study [9]. This temperature will increase in lower ambient temperatures and conversely will decrease in hot ambient temperatures. Third, while brooding during daylight, the light-level measured on the dorsal part of the chick will be very low (Fig. 2b). This light level should be close to zero, or at least much lower than the light level provided at the same time by the shield of trees, shrubs, or plants in the locality (Additional file 2: methods SM1). These assumptions were clearly confirmed both by inspections of visualized recordings (see the clear signal of all above-mentioned patterns in Fig. 2 and Additional file 1: actograms) and by observations, including analyses of video-recorded data (total 48 min of 22 chicks) and referential light-level measurements obtained under the plants (N = 559, see Additional file 2: methods SM1).

During the night the temperature pattern is usually strong, clearly visible, and consistent with the assumptions described above. The temperature decreases to/is similar to the ambient temperature during the high activity bouts and increases/is high during low-activity bouts (see the example actogram Fig. 2a, c). Conversely, during the daylight the ambient temperatures were often high and this consistency in temperature and activity patterns was only weak. However, daylight brooding bouts are clearly distinguishable when light-level measurements are used. The best conditions, when the consistency of all three described patterns may be clearly seen, are daylight with low ambient temperatures.

To predict when brooding behaviour occurs, we first fitted the ODBA time-series with the two-state hidden Markov model (HMM), using unsupervised learning via the Baum–Welch algorithm [43]. We estimated the HMM for each chick separately. This process found the most likely way to distinguish between “active” and “non-active” behaviour in the analysed time-series, where brooding (among others) is classified as “non-active” behaviour. Then, we used the Viterbi algorithm to detect the most likely path of active/non-active behaviour within the dataset for a chick, based on model-estimated parameters [44].

Subsequently, to distinguish real bouts of brooding from “non-brooding” bouts of low activity, it was necessary to proceed with a series of post-processing steps, including an automated algorithm for correcting common extraction errors, and finally a thorough manual inspection and correction of errors arising from the evaluation using automated algorithms.

The automated part of the post-processing consisted of several steps. First, we considered low-activity daylight bouts to be brooding only if they were (at least for a part of the bout of low activity level) associated with a light-level lower than 50 lx. We selected this arbitrary threshold based on analyses of luxmeter measurements as being improbably low, not reachable during any activity other than brooding (e.g. not due to hiding in a shield of dense vegetation, or cloudy weather). In particular, the selected threshold is a ~ 1% quantile of the measurements obtained by loggers placed under various sources of shade (for details, see Additional file 2: methods SM1). Note that this threshold is exceeded when the chicks are hidden in the shade created by the parent’s body but are not pressed to the parent’s abdomen. However, with the selected approach we could not distinguish between shading by the parent’s body and shading by plants or trees, which is frequently used by chicks of both species (unpublished data). We therefore adopted the definition of brooding involving direct contact between the dorsal part of a chick and the abdomen of a parent. It cannot be ruled out that the threshold of 50 lx may not be exceeded even if the chick crouches very tightly under e.g. a tree root. However, these situations can be revealed because the brooding parent, unlike the root, raises the logger temperature. Only during the hottest part of the day is it impossible to distinguish these situations, as the parent (similar to the root) cools down during brooding. However, as we will show, these events are very rare (less than 0.5% of the brooding time at ambient temperature above 35 °C) and therefore they cannot significantly affect the interpretation of the results. It may also be because there are few such structures (dark shelters under the roots) in the study area, as opposed to shrubs with partial shade where the chicks preferentially seek occasional protection from heat or predators.

Second, because chicks sometimes began to increase their activity before the end of the brooding bout or began to be inactive slightly before the brooding started, we shifted the starts and ends of the brooding bouts where the temperature changes were not consistent with the prediction. In particular, we set the beginning of each brooding bout to the earliest point where the temperature difference was above the median of the temperature differences during the brooding bouts of a particular chick. Similarly, we shifted the start of each break after a brooding bout to the first temperature difference above the median of the temperature differences of the “active state”, after the period of low activity. Third, we omitted very short brooding bouts and breaks in brooding (up to 20 s) as likely falsely determined (usually associated with a change of position during an uninterrupted brooding bout). These post-processing steps greatly improved the consistency of the brooding prediction with observed patterns. Finally, we visualised all datasets with predicted brooding bouts [45], and one author (VK) inspected them the datasets for apparent failures of the prediction process and corrected them manually. These corrections affected only ~ 1% of the monitoring time.

An important limitation of the methodological approach that was used is that once the growing feathers cover the light-level sensor (~ 65 g, ~ 20 days in the Northern lapwing, ~ 25 days in the Red-wattled lapwing), brooding becomes virtually undetectable in high ambient temperatures. Therefore, to avoid bias, we identified such chicks during the visual inspection and excluded them from further analyses.

## Ambient temperatures

Because the chicks move close to the ground and most often in places without any shade, we decided not to use the standard ambient temperature measurements taken two metres above the ground and in the shade. Rather, we defined the ambient temperature as a near-ground temperature taken on the direct sun. For our estimates of the ambient temperature, we used temperature measurements at ~ 5 cm above the ground around the nests of the model species taken as a part of other project [35]. If the data from more loggers were available at the same time, we used the median temperature of all loggers. For the estimates for the time periods in which no data loggers were installed near to the nests, we used the estimates from regression models that predicted the near-ground temperatures on the basis of the weather measurements from meteorological stations. For more details about temperature measurements, sample sizes, exact specifications of the models and evaluations of the models, see Additional file 2: methods SM2.

## Statistical analyses

### General procedure

We performed all statistical analyses in R 3.5.3. [38]. The general linear models were fitted using the ‘lm’ function [38], while the general(/generalised) mixed-effects models were fitted using the ‘lmer‘(/‘glmer’) functions from the ‘lme4’ library [46]. We z-transformed (mean-centred and divided by SD) all numerical predictors in the models (except for the time of day) [47]. Whenever the time of day was included in the model, we transformed it to radians ((2*time*π)/24) and then fitted it as the sine and cosine of the radians [48]. We visually inspected model assumptions from diagnostic plots [45]. For all fitted linear and generalised linear models, we used the “sim” function from the “arm” R package and noninformative prior distribution [49] to create a sample of 5000 simulated values for each model parameter (posterior distribution). We then reported the effect sizes and the model predictions as the medians and the uncertainty of the estimates and predictions as Bayesian 95% credible intervals (95% CrI) represented by the 2.5 and 97.5 percentiles of the posterior distribution of the 5000 simulated values.

### Hourly brooding rate

We defined the hourly brooding rate as the proportion of the hour for which the chicks were brooded. We fitted a generalised linear mixed-effect model with a binomial error structure and logit-link function. As a response, we used the number of minutes in which the chick was brooded and the number of minutes without brooding in a binomial denominator. The following predictors were included: species, the time of day, night (yes/no whether the sun was > 6° below the horizon), chick head length (as a proxy for its age), and ambient ground temperature, as well as two-way interactions of the species with all three numerical predictors, and the time of day with the length of the head and the ambient ground temperature. To eliminate temporal autocorrelation, we also included the probability of being brooded in the previous hour. As the data included repeated measurements of the same chick, and sometimes also simultaneous measurements of multiple chicks from the same family, we specified the chick identity nested in the family identity as a random intercept, and the ambient ground temperature and the time of day (both the sine and cosine terms) as random slopes [50]. To avoid overdispersion of the model, we further implemented the Observation-Level Random Effect [51]. Finally, because some hours did not have a complete set of measurements, we weighted the model by the square-root of the number of minutes with the measurements available for a given hour.

### Brooding efficiency

We defined the brooding efficiency (i.e. the quality of brooding care) as the median temperature measured by the datalogger on the chicks’ dorsal part during the brooding bout after temperature becomes stable. Because the temperature was usually stabilised after no longer than 5 min after brooding bout initiation [45], we selected only bouts longer than 5 min, and we excluded the first 5 min from the median calculation.

We then used the median temperatures measured during the brooding bouts as a response in a linear mixed-effects model with the Gaussian error structure. The following predictors were included: species, night (yes/no whether the sun was > 6° below horizon), chick head length (as a proxy for its age), and the ambient ground temperature, as well as two-way interactions of the species with all remaining predictors and the night with the ambient ground temperature. We included the chick identity nested in the family identity as a random intercept, and the ambient ground temperature as a random slope [50].

### Effect of the time of brooding on chick growth

To test whether the time spent on being brooded impacted the growth rate of the chick, we first calculated the differences in the weights of the chicks before and after installation of the datalogger and divided them by the length of datalogger deployment (in hours). Then, because this growth rate is species- and age-specific, we used it as a response in a general linear model with the species and head length (as a proxy for age) as predictors (including their interaction). Finally, we used residuals from this model as a response in a general mixed-effects model with the Gaussian error structure. The following predictors were included: proportion of time for which the chicks were brooded (within the whole sampling period), and the species (including the interaction between both predictors). Because the growth rate of the chicks belonging to the same family may not be independent of each other, we included the family identity as a random intercept.

## Results

### Variation in brooding rates

**Fig. 3:**
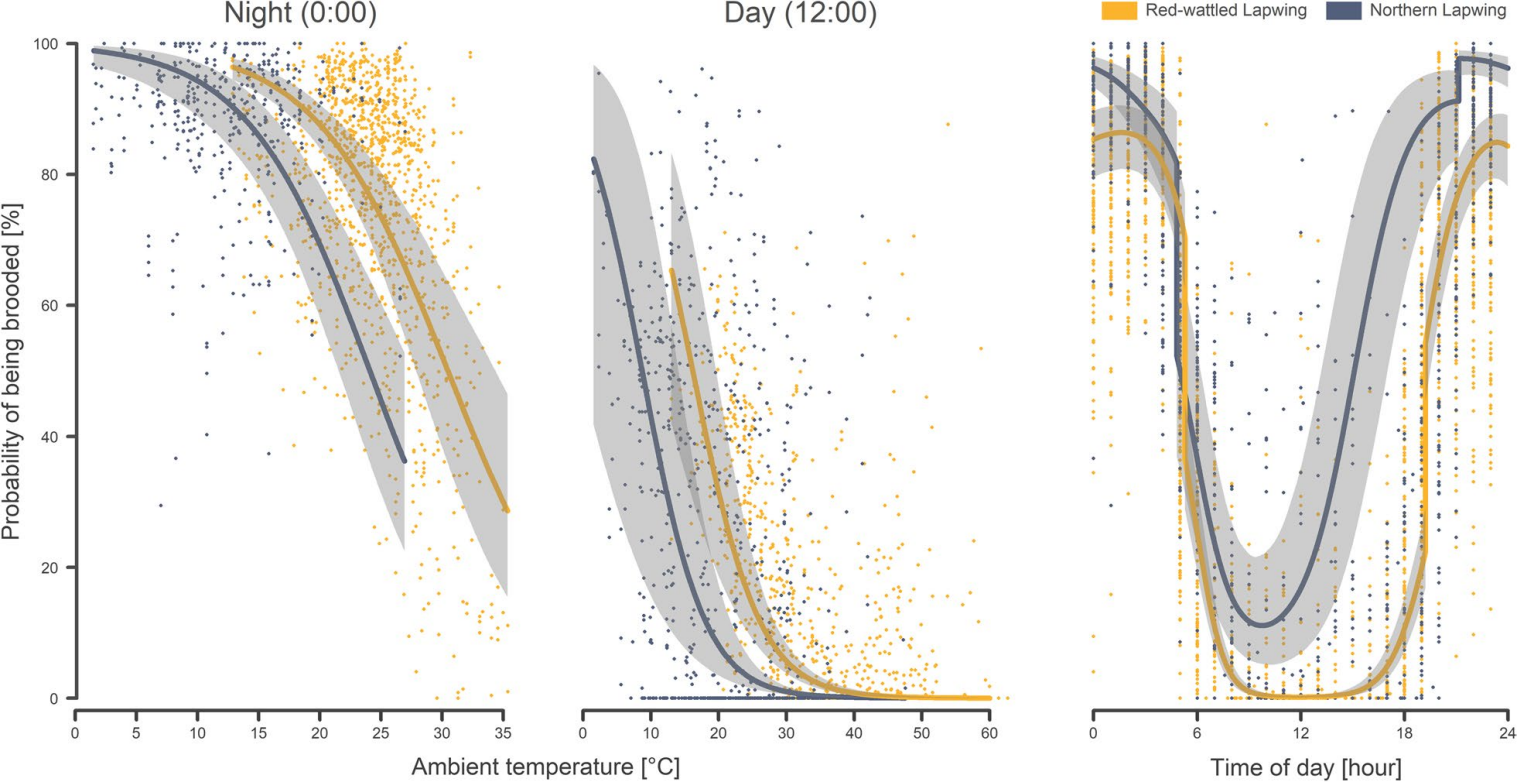
The probability of being brooded—in relation to species, ambient temperature, and the time of day. The points show the probability of Red-wattled lapwing chicks (yellow) and Northern lapwing chicks (blue) being brooded during nighttime (**a**) and during daytime; (**b**) in relation to the ambient temperature. **c **presents the diel pattern of brooding. The curves with a shaded area indicate a model prediction with a 95% credible interval [49]. Variance components were estimated by the ‘glmer’ function for binomial errors with the logit link function [46]

Overall, the proportion of time spent on being brooded (hereafter “the brooding rate”) was relatively similar for chicks of both studied species. The Northern lapwings brooded chicks for 40% of the time (median, 25–75th quantile: 35–45%; n = 1367 h of 38 chicks), while the Red-wattled lapwings brooded their chicks for 35% of the time (median, 25–75th quantile: 29–39%; n = 2824 h of 65 chicks). In both species, the hourly brooding rates decreased steeply with increasing ambient temperature (Table 1; Fig. 3a, b). In addition, and beyond the ambient temperature effect, the hourly brooding rates showed a similarly strong diel pattern, being higher during the night than in the light period of the day (Table 1; Fig. 3c). Furthermore, the brooding rates tended to decrease during chick growth in both species, although the effect of chick size was much smaller than the effect of temperature or the effect of the time of the day (Table 1). This resulted in relatively high overnight brooding rates, even in the eldest chicks included in the study.

While both species were consistent in their brooding patterns, the effects differed between species in magnitudes (Table 1; Fig. 3). In particular, the brooding rates of the Northern lapwing decreased much more rapidly during the chick growth. Also, the diel pattern of brooding differed between the species, mostly because of the differences in the length of the daytime (Fig. 3c). Importantly, within comparable temperature conditions, the Northern lapwings brooded their chicks much less (Fig. 3a, b). For example, assuming 20 °C (~ 71% and ~ 9% quantiles of the temperatures during the breeding seasons in the Czech Republic and in Dubai, respectively), and an approximately 1-week-old chick, the mean model-predicted brooding rate during daylight is only 1.5% for the Northern lapwing (95% CrI 1–3%) and 58% for the Red-wattled lapwing (95% CrI 38–75%). In contrast, during the night (assuming identical temperature conditions and chick age), the brooding rates are much higher in both species, i.e. 73% for the Northern lapwings (95% CrI 63–80%) and 85% for the Red-wattled lapwing (95% CrI 79–89%).

**Table 1:** Hourly brooding rates

Response	Effect type	Effect	Estimate	95% CrI	
				Lower	Upper
% of brooding in 1 h	Fixed	**Intercept**	**– 2.302**	**– 2.521**	**– 2.091**
		**Brooding before**	**0.574**	**0.468**	**0.679**
		**Night (yes)**	**1.434**	**1.266**	**1.607**
		**SpeciesVV**	**– 1.886**	**– 2.398**	**– 1.379**
		**Sin (time)**	**– 0.557**	**– 0.743**	**– 0.373**
		**Cos (time)**	**0.97**	**0.584**	**1.369**
		**Ambient temperature**	**– 3.01**	**– 3.505**	**– 2.503**
		**Head length**	**– 0.526**	**– 0.693**	**– 0.357**
		**SpeciesVV: Sin (time)**	**– 0.889**	**– 1.285**	**– 0.495**
		**SpeciesVV: Cos (time)**	**1.476**	**0.897**	**2.053**
		SpeciesVV: Ambient temp.	0.408	– 0.356	1.163
		SpeciesVV:Head length	– 0.251	– 0.618	0.132
		**Sin (time): Ambient temp**	**– 0.379**	**– 0.528**	**– 0.227**
		**Cos (time): Ambient temp**	**0.77**	**0.56**	**0.986**
		**Sin (time):Head length**	**0.189**	**0.1**	**0.281**
		**Cos (time):Head length**	**0.428**	**0.241**	**0.629**
	Random % variance	ID chick nested in family (Intercept)	1%		
		Sin (time)	1%		
		Cos (time)	1%		
		Ambient temperature	8%		
		ID family (Intercept)	8%		
		Sin (time)	3%		
		Cos (time)	21%		
		Ambient temperature	29%		
		OLRE	28%		

Posterior estimates (medians) of effect sizes with 95% credible intervals (Crl) from a posterior distribution of 5000 simulated values generated by the ‘sim’ function in R [49]. Variance components were estimated by the ‘glmer’ function for binomial errors with the logit link function [46]. Continuous predictors and the random slope were z-transformed (mean-centred and divided by SD). Those contrasts with 95% credible intervals not containing 0 are highlighted in bold.

### Brooding efficiency

**Fig. 4:**
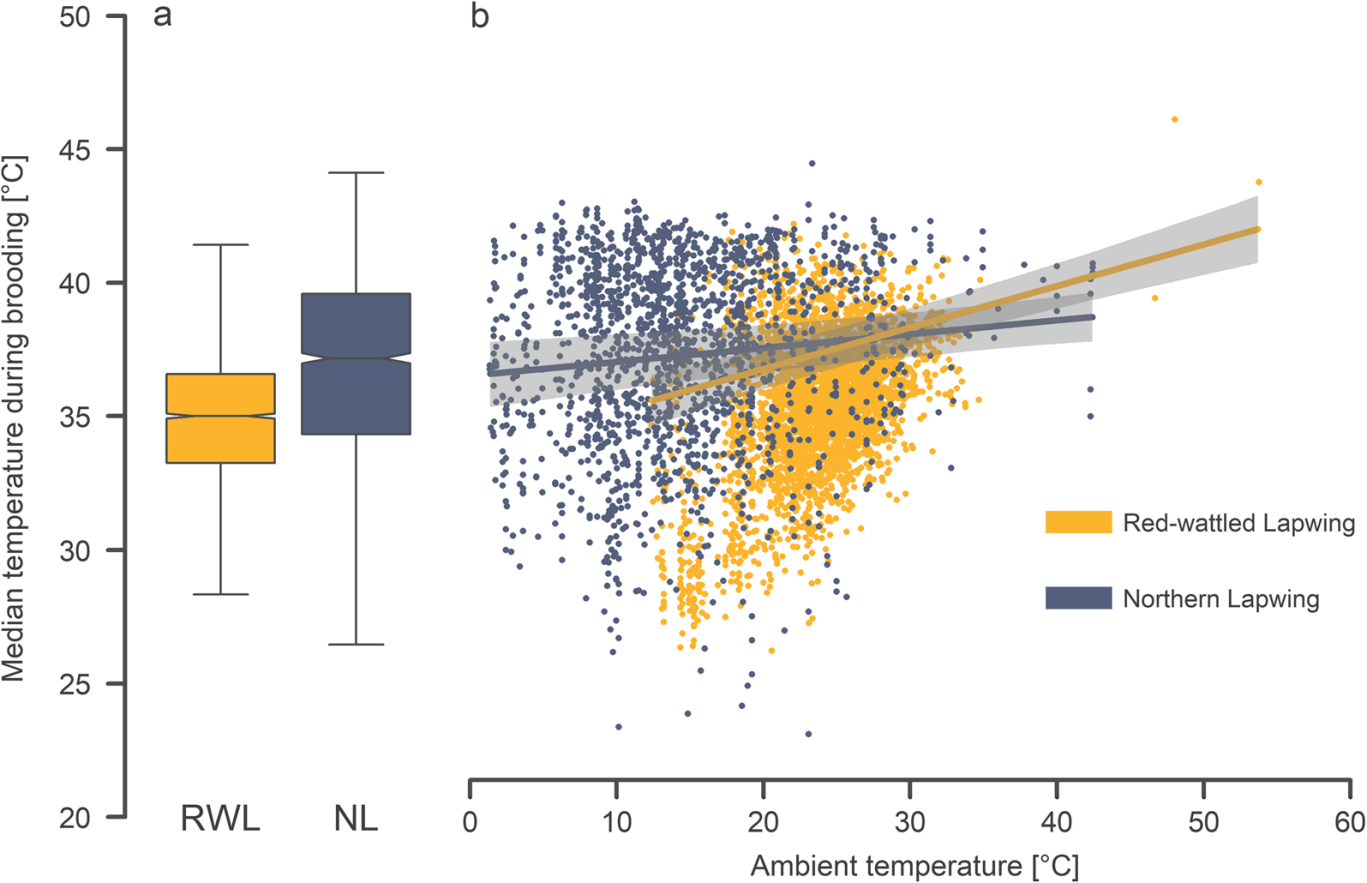
**a** The boxplots summarise the median temperature during brooding after stabilisation (i.e. after the end of the 5th min) in relation to species: Red-wattled Lapwing (yellow) and Northern Lapwing (blue). The median temperature during brooding is depicted by the vertical line inside the box, its 95% confidence interval is depicted by the notch, and the 25–75% quantiles are depicted by a box. **b** The median temperature during brooding in relation to the ambient temperature for the Red-wattled lapwing (yellow) and for the Northern lapwing (blue). The curves with a shaded area indicate the model prediction with a 95% credible interval

Parents of the Northern lapwings were generally more efficient in heating their chicks than the Red-wattled lapwing parents (Fig. 4). Although the Northern lapwings were living in much lower ambient temperatures, the median temperature measured on the back of the brooded chicks (median = 37.2 °C; 25–75th percentile: 34.3–39.6 °C; n = 1816 bouts longer than 5 min) was more than 2 °C higher than on the Red-wattled lapwing chicks (median = 35.0 °C; 25-75th percentile: 33.3–36.6 °C; n = 3260 bouts longer than 5 min.; Fig. 4a). Moreover, while the Northern lapwing parents were able to maintain similar brooding temperatures even in very cold ambient temperatures, the brooding temperatures dropped with decreasing ambient temperatures in the Red-wattled lapwings (Fig. 4b; Table 2).

In addition, both species tended to show slightly lower (~ 2 °C; Table 2) brooding efficiency during the night and, surprisingly, the relationship between brooding temperature and chick size differed between the two species. While in the Northern lapwing the smaller chicks tended to have lower brooding temperatures, in the case of the Red-wattled lapwing it was the larger chicks that tended to have lower brooding temperatures.

**Table 2:** Brooding efficiency

Response	Effect type	Effect	Estimate	95% CrI	
				Lower	Upper
Median temperature during brooding [°C]	Fixed	**Intercept**	**36.414**	**35.741**	**37.099**
		Species (VV)	0.418	− 0.71	1.642
		**Night (Yes)**	**− 2.119**	**− 2.386**	**− 1.856**
		**Ambient temperature**	**1.11**	**0.825**	**1.403**
		Head length	0.331	− 0.048	0.716
		SpeciesVV: Night (Yes)	0.346	− 0.063	0.772
		**SpeciesVV: Ambient temperature**	**− 0.737**	**− 1.089**	**− 0.391**
		Night (Yes): Ambient temperature	− 0.074	− 0.262	0.112
		**SpeciesVV: Head length**	**− 1.084**	**− 2.043**	**− 0.12**
	Random % variance	ID chick nested in family (Intercept)	25%		
		Ambient temperature	2%		
		ID family (Intercept)	27%		
		Ambient temperature	1%		
		Residual	45%		

The response variable is the median temperature measured on the chicks’ dorsal part after stabilisation (i.e. after the end of the 5th min). Posterior estimates (medians) of the effect sizes with the 95% credible intervals (Crl) from a posterior distribution of 5000 simulated values generated by the ´sim´ function in R [49]. Variance components were estimated by the ‘lmer’ function with the Gaussian error structure [50]. Continuous predictors and the random slope were z-transformed (mean-centred and divided by SD). The contrasts with 95% credible intervals not containing 0 are highlighted in bold

### The effect of brooding rate on chick growth

The speed of chick growth during the observation period after accounting for chick size and species did not depend on the brooding rate (Table 3).

**Table 3:** Effect of brooding rate on chick growth

Response	Effect type	Effect	Estimate	95% CrI	
				Lower	Upper
Residual growth speed [g/day]	Fixed	Intercept	0.434	− 1.078	1.96
		Brooding rate	− 1.34	− 5.624	2.86
		SpeciesVV	− 1.608	− 4.646	1.314
		Brooding rate:SpeciesVV	4.318	− 2.827	11.692
	Random % variance	ID family (Intercept)	72%		
		Residual	28%		

Posterior estimates (medians) of the effect sizes with 95% credible intervals (Crl) from a posterior distribution of 5000 simulated values generated by the ‘sim’ function in R [49]. Variance components were estimated by the “lmer” function with the Gaussian error structure [50]. The contrasts with 95% credible intervals not containing 0 are highlighted in bold

## Discussion

In this study, we have revealed the patterns of parental brooding care for chicks of two congeneric species living under contrasting environmental conditions. We found that the overall brooding rates were relatively similar in both species and were shaped by the ambient temperature, the chick’s age, and most strongly by the alternation of day and night. However, the adults of species breeding in hot climates brooded their chicks at higher temperatures and less efficiently than the adults of their temperate relatives. Finally, the time spent on brooding did not affect the growth rate of the chicks. We discuss all these aspects in detail below.

### Factors influencing the brooding rates

#### Ambient temperature

In line with expectations and as reported elsewhere [9–11, 16, 52], the time spent on brooding by both species decreased steeply with increasing ambient temperatures. An interesting question was whether brooding may also serve to cool the chicks down under extremely high temperatures. This was a relevant question, especially because one of the studied species, the Red-wattled lapwing, inhabits environments with often extremely high temperatures, which may be harmful or lethal for chicks without fully-developed homeothermy [53]. Our results indicate that brooding in extremely high temperatures probably occurs (see brooding bouts at more than ~ 40 °C in Fig. 3), but only very rarely. Usually, chicks facing extremely high temperatures choose other forms of cooling, such as shade of plants or staying next to water resources (our own observation). This can be well observed in actograms (Fig. 2d), where we can frequently see bouts of low activity connected with a decrease in temperature and a partially reduced light level (to a level corresponding to staying in the shade). However, it cannot be excluded that chicks may in such cases use the parent only as a shield (i.e. lie or stay in the shade of the parent), without direct contact with the parent’s body. Such behaviour is indistinguishable from the approach used in this study and does not fall within the definition of brooding adopted in this study.

### Chick´s age effect

Contrary to expectation, we found a weak decrease in the brooding rate with the age of the chick. This is surprising because, according to current knowledge, the need for brooding should decrease as the chick´s thermoregulation improves [52]. In related shorebird species, brooding was never or rarely observed in Piping plover (*Charadrius melodus*) chicks older than 14 days [54], in Semipalmated plover (*Charadrius semipalmatus*) chicks older than 5 days [55] or in Red knot (*Calidris canutus*) chicks older than 9 days [16]. However, it is important to note that all these studies cover only daylight observations, while we recorded the highest brooding rates (even in older chicks) during the night.

It is important to admit a methodological limitation of our study associated with the development of the chick´s plumage. Well-grown feathers on the back of chicks weighing ~ 70 g at the age of ca. 14 days in the Northern lapwing and ca. 21 days in the Red-wattled lapwing may overlay the sensor and may make it impossible to distinguish reliably between brooding and inactivity, in particular at ambient temperatures close to the body temperature of the parents. Since these problematic chicks were excluded from the analysis, it is possible that the steepest decline in the brooding rates, related to the achievement of thermoregulation independence, only occurs in older chicks. As found in laboratory conditions, the chicks of Crowned plover (*Vanellus coronatus*), an another congeneric, become thermoregulation independent in the 33–39% adult weight [8], i.e. ~ 70 g, which well corresponds to the stage when the Northern lapwing and the Red-wattle lapwing feathers develop to the state preventing reliable use of sensors [56].

However, our episodic observations indicate continued chick brooding in both studied species even after a weight of 70 g is exceeded, sometimes almost until fledging. First, even the chicks excluded from the analysis frequently showed overnight bouts of low activity at high ambient temperatures, indicating brooding. Second, we recorded brooding of a 45-day-old chick on the nest where the chicks from the subsequent clutch were hatching (Additional file 2: Picture S5). However, given the relatively favorable temperature during the nights (especially in the case of the Red-wattled lapwing), we suggest that such overnight brooding of nearly-fledged chicks may have an antipredatory function, rather than a thermoregulation function (discussed below).

### Diel pattern

The brooding rates of both species were far higher during the night than during the daylight hours. Importantly, although temperatures are typically lower during the night, such a high need for overnight brooding cannot be explained by the need to be warmed up alone, because, at comparable temperatures, the chicks were brooded much more at night than during the daylight. We offer two possible (not mutually exclusive) explanations for this finding.

First, although foraging has frequently been observed during the night in adult plovers Charadriidae [24, 25], the night foraging efficiency of the chicks may be low. It may therefore be more beneficial to save energy and maintain body temperature with the help of their parents than to forage with the prospect of low energy intake. Second, there can be a higher risk of predation during the night [56–59], or at least, the parents have a more limited ability to detect and repel the predator in advance [60]. Thus, being motionless and close to a vigilant parent may increase the chick survival rate. This explanation may be particularly valid even for chicks shortly before fledging (as discussed above).

From the methodological point of view, it is worth mentioning that the demonstrated difference between the behaviour during the night and during the day also underlines the extreme importance of studying behavioural phenomena throughout the 24-h cycle. Omitting the night-time in behavioural studies of wild animals may cause strong bias in comparing estimates of the rate of a particular behaviour, and may also hamper the ability to reveal the true latitudinal trends [61]. The need to investigate the whole 24-h cycle is demonstrated in the comparison between the brooding rates found in this study and the results of other published estimates. In particular, in our study, the chicks of the Red-wattled lapwing were brooded for 35% of the time, while the Northern lapwing chicks were brooded for 40% of the time. These estimates seem to be similar to the 38% level found for the chicks of Snowy plover (*Charadrius alexandrinus*) [10], and the 34% level found for Crowned lapwing [19]. However, both referenced studies are based only on daylight observations. Therefore, if these species exhibit much higher overnight brooding rates, similarly to the model species of our study, the comparison is probably misleading, and the true brooding rates in Snowy plovers and Crowned lapwing are probably much higher in comparison with Red-wattled lapwing and Northern lapwing.

The importance of night-time observations for revealing latitudinal trends may be shown in a comparison between our two model species. Chicks of the Northern lapwing generally live in a much colder environment and consequently need to be brooded much more during daylight. But they also live much more northerly (i.e. at a latitude with much shorter nights). Because the brooding rates differ considerably between day and night, the difference in the length of the night strongly mitigates the observed difference between the brooding rates of the two species. The day-night change in animal behaviour should therefore definitely be taken into consideration in all field observational studies.

### Between-species differences

Overall, the adults of the temperate Northern lapwing brooded chicks for only ~ 5% more time than the adults of the Red-wattled lapwing living in a hot desert. This difference is rather small, given the magnitude of the differences in the climatic conditions faced by the two species. To demonstrate this difference, the studied chicks of the Northern lapwing faced temperatures lower than 10 °C for ~ 20% of the time, but such temperatures never occurred during the observations of the Red-wattled lapwings. On the other hand, temperatures higher than 30 °C were reached during ~ 50% of the observation time in the Red-wattled lapwing, but only during ~ 10% of the observation time in the Northern lapwing. A much higher level of necessary brooding might therefore have been expected for the species living in a colder environment. The most likely explanation for this discrepancy lies in the different lifestyles of the two related species. Our results show at least two ways in which the Northern lapwing shows better adaptations to breeding in colder climates.

First, the Northern lapwing chicks seem to have a significantly reduced threshold below which they need to be brooded (i.e. they are brooded much less in comparable temperatures). Second, the Northern lapwing parents seem to be more efficient during brooding. Notably, the Northern lapwing adults were able to maintain a higher brooding temperature of their chicks even in ambient temperatures close to 0 °C, while the brooding temperature of Red-wattled lapwing parents tended to drop during brooding in lower ambient temperatures. This may be caused by the generally faster metabolism [28] and thus the ability of the Northern lapwing parents to produce and provide more heat to the chicks. Moreover, the ability to transfer heat to their chicks efficiently during brooding may be linked to adaptations enabling efficient incubation of the eggs, especially the extent and the level of development of the brood patch [62]. The lower efficiency in brooding the chicks may be a consequence of the fact that, during egg incubation in an extremely hot desert climate, the most critical task is to cool the eggs during the hottest parts of the day, rather than heating them during the still relatively warm nights [63]. However, to disentangle the proximate mechanisms underlying the differences in brooding efficiency, and to confirm the general validity of the patterns discussed above, we need more studies on different species.

### The effect of brooding rate on growth

Contrary to our expectations, we failed to find any clear relationship between the brooding rate and the growth rate. This might be considered surprising, because other studies have reported that small chicks of the Northern lapwing, the Black-tailed godwit (*Limosa limosa*) and the Redshank (*Tringa totanus*) cannot maintain their weight when the time available for foraging drops below 25–30%, and their growth is hampered even if the time available for foraging drops below 50% [11]. When averaging the brooding rates over the whole period of recording for a particular chick in our study, we found a high degree of variation (18–51% in the Red-wattled lapwing, 24–64% in the Northern lapwing). However, as discussed above, the brooding rates estimated by previous studies are probably underestimated because of the absence of night-time measurements. It therefore seems probable that brooding rates within the range captured by our data still leave the chicks sufficient time for foraging and for other important activities that are needed for successful growth.

## Conclusions

To the best of our knowledge, this is the first study that has evaluated brooding simultaneously from the perspective of the brooding rate, the efficiency, and the impact on the growth rate of precocial bird chicks. Although the two studied species did share similarities in brooding patterns during the 24-h cycle and in response to the ambient temperature, they clearly differed in the thermal comfort range of the chicks and in the brooding efficiency of their parents. Day-night alternation and ambient temperature thus seem to be general drivers of brooding patterns in birds across the latitudes and environments. In contrast, the temperature range within the chicks need to be brooded, and the brooding efficiency of the parents, may be the traits closely linked to the species-specific adaptations to climate. Adaptation on colder climate may include less thermal demands of the young but may at the same time increase the efficiency of parental brooding care. These are hitherto undocumented traits in the life histories of birds living in a hot environment. However, confirming the general validity of this finding across species, latitudes and environments will require a thorough comparative analysis.

## Supplementary Information

**Additional file 1:** Actograms of all individual chicks.

**Additional file 2:** **Supplementary pictures: Picture S1**. Illustration of the study area of the Northern lapwing, including typical habitats of species occurrence. **Picture S2**. Illustration of the study area of the Red-wattled lapwing, including typical habitats of species occurrence. **Picture S3**. Illustration of the used multisensory datalogger DAL2. **Picture S4**. Illustration of the deployment of the DAL 2 logger on Chicks of the Red-wattled lapwing. **Picture S5**. Camera footage showing the brooding of a 45-days old chick of a Red-wattled lapwing with a newly hatched chick from a subsequent breeding attempt. **Supplementary methods: SM1**: Procedure for obtaining reference values of light-levels in different types of natural shade. **SM2**: Procedure to estimate near-ground temperature from ambient temperature.

## Data Availability

All data and computer code to replicate our analyses are freely available from OSF: https://osf.io/xjc49/.
