# Supplementary material for: Offspring thermal demands and parental brooding efficiency differ for precocial birds living in contrasting climates
Source: Front Zool. 2023 Apr 10;20:12. doi: 10.1186/s12983-023-00492-1 (PMC10084700; doi:10.1186/s12983-023-00492-1)
Supplement: Supplementary file 1 — Additional file 1: Actograms of all individual chicks. [file 12983_2023_492_MOESM1_ESM.pdf]

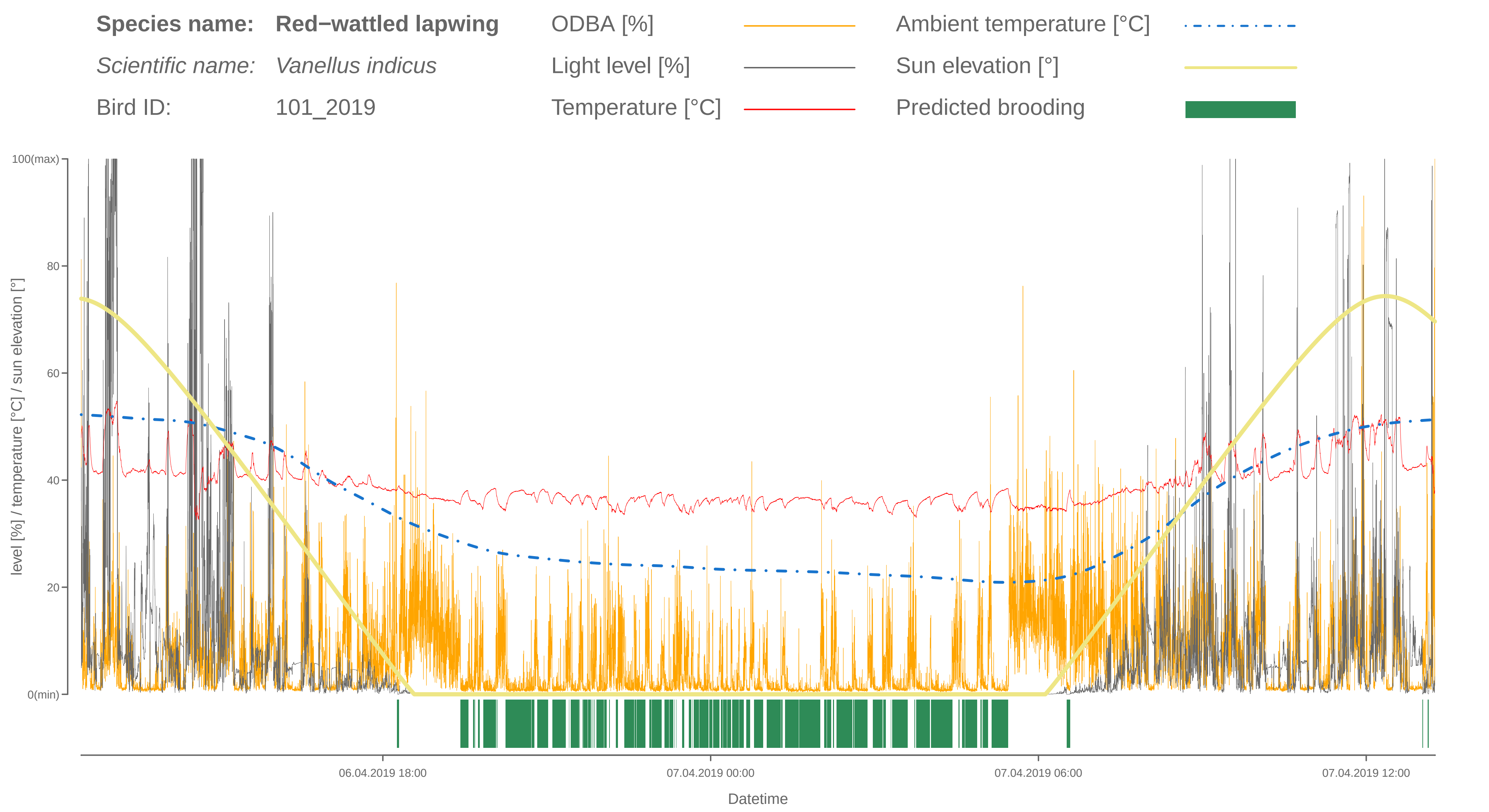

Species name: Red−wattled lapwing
Scientific name: Vanellus indicus
Bird ID: 101_2019
ODBA [%]
Light level [%]
Temperature [°C]
Ambient temperature [°C]
Sun elevation [°]
Predicted brooding
100(max)
80
60
40
20
0(min)
level [%] / temperature [°C] / sun elevation [°]
06.04.2019 18:00
07.04.2019 00:00
07.04.2019 06:00
07.04.2019 12:00
Datetime

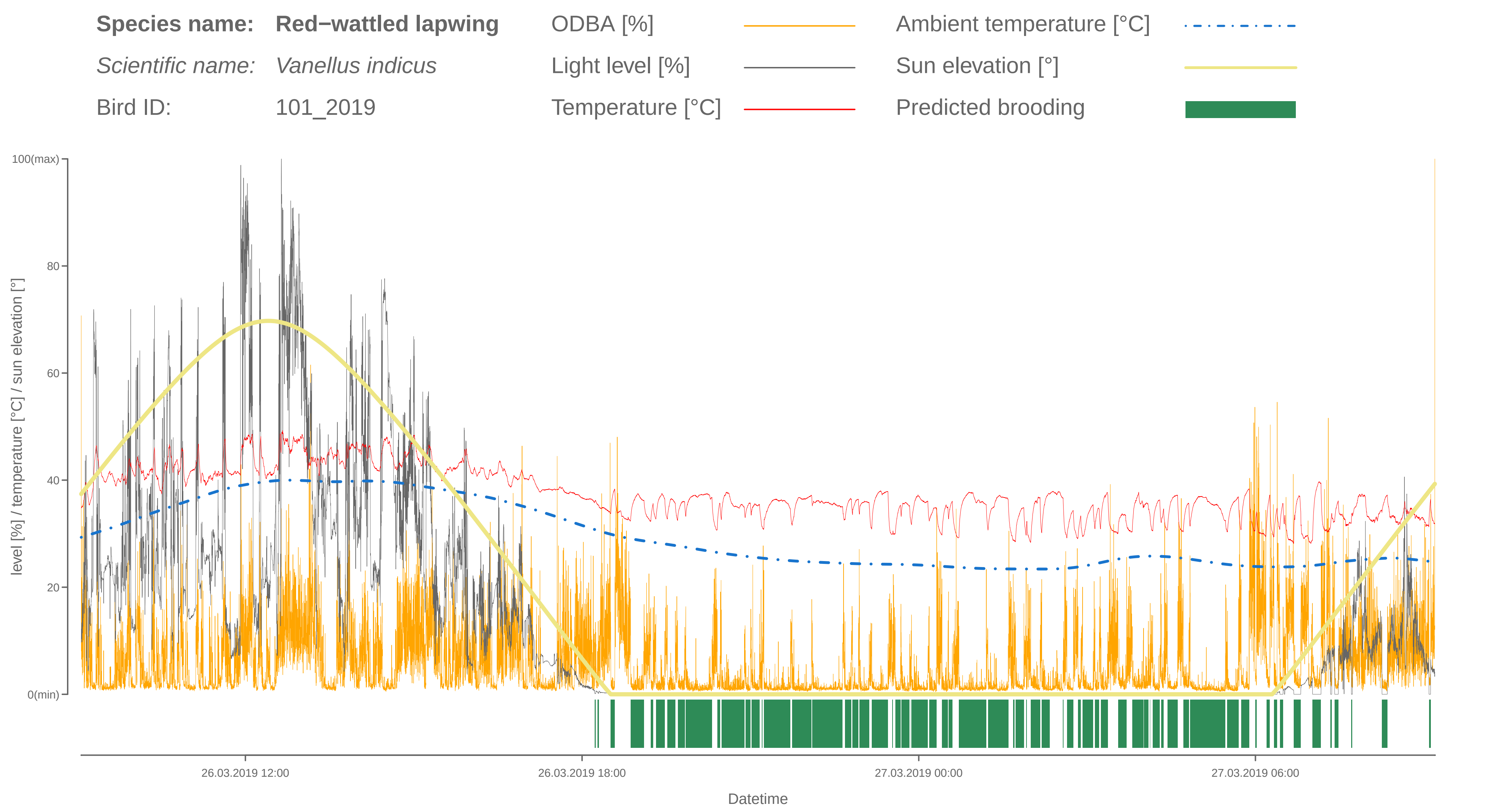

Species name: Red−wattled lapwing
Scientific name: Vanellus indicus
Bird ID: 101_2019
ODBA [%]
Light level [%]
Temperature [°C]
Ambient temperature [°C]
Sun elevation [°]
Predicted brooding
100(max)
80
60
40
20
0(min)
level [%] / temperature [°C] / sun elevation [°]
26.03.2019 12:00
26.03.2019 18:00
27.03.2019 00:00
27.03.2019 06:00
Datetime

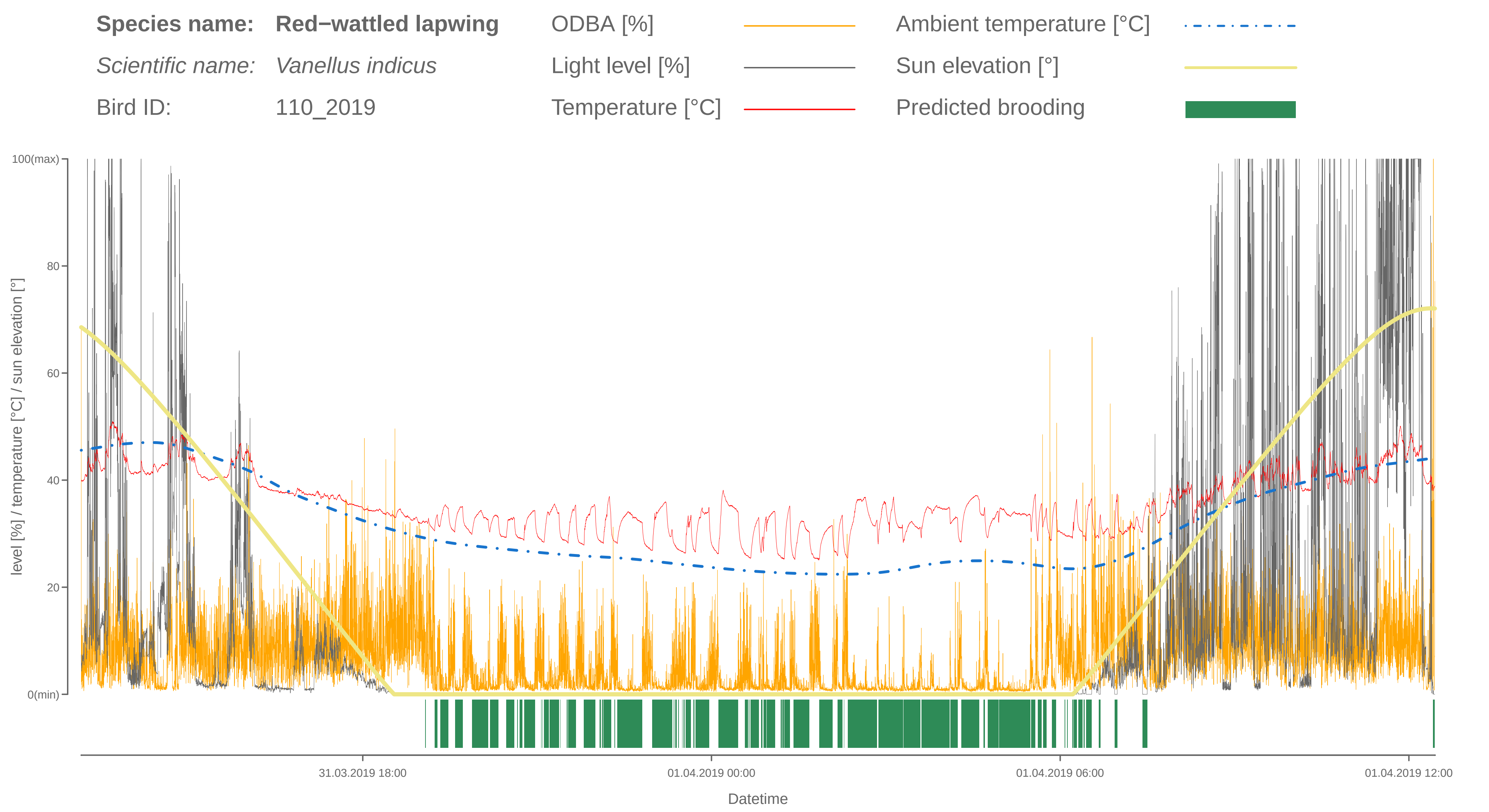
Species name: Red−wattled lapwing
Scientific name: Vanellus indicus
Bird ID: 110_2019
ODBA [%]
Light level [%]
Temperature [°C]
Ambient temperature [°C]
Sun elevation [°]
Predicted brooding
100(max)
80
60
40
20
0(min)
level [%] / temperature [°C] / sun elevation [°]
31.03.2019 18:00
01.04.2019 00:00
01.04.2019 06:00
01.04.2019 12:00
Datetime

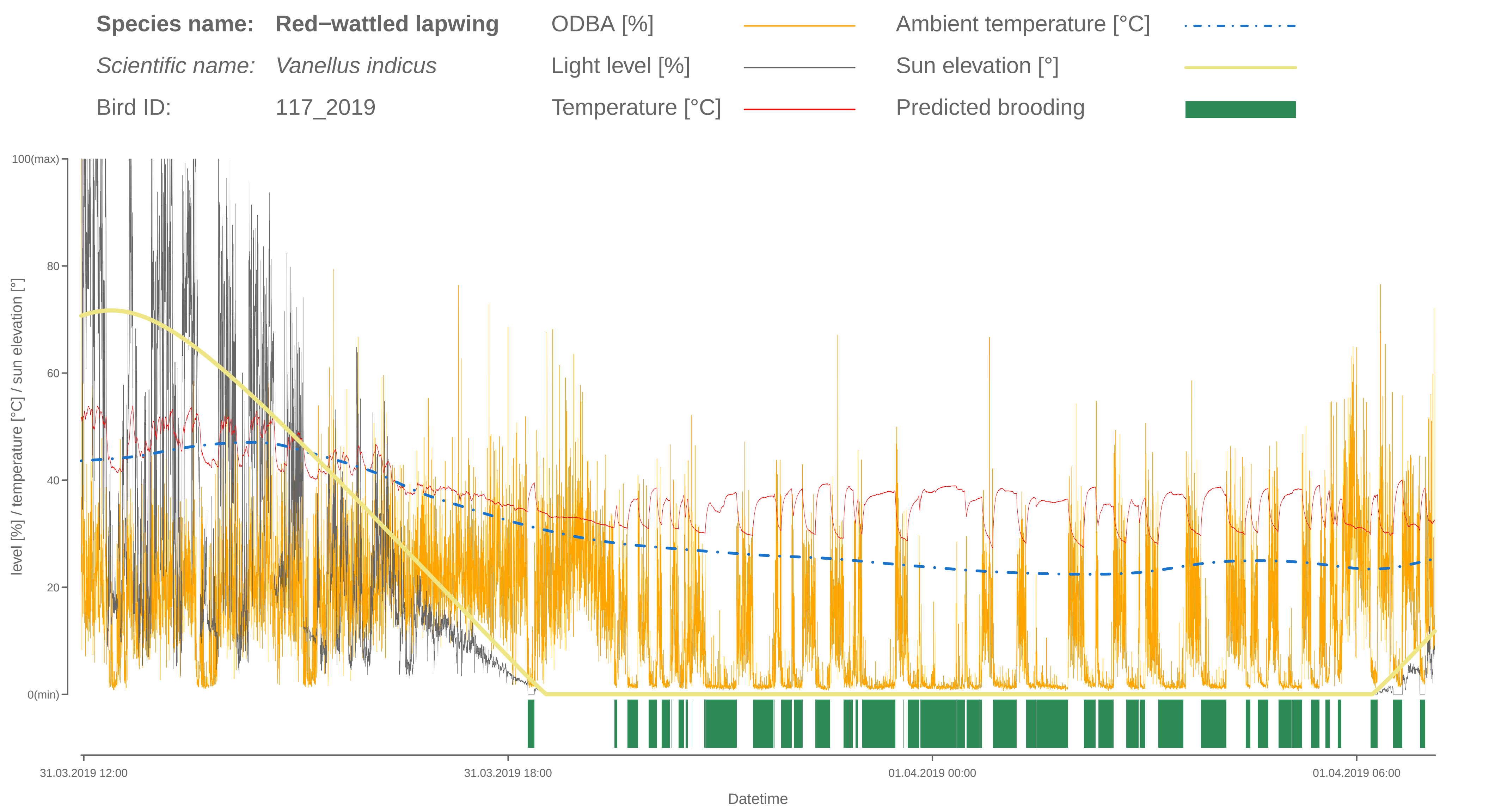

Species name: Red−wattled lapwing
Scientific name: Vanellus indicus
Bird ID: 117_2019
ODBA [%]
Light level [%]
Temperature [°C]
Ambient temperature [°C]
Sun elevation [°]
Predicted brooding
100(max)
80
60
40
20
0(min)
level [%] / temperature [°C] / sun elevation [°]
31.03.2019 12:00
31.03.2019 18:00
01.04.2019 00:00
01.04.2019 06:00
Datetime

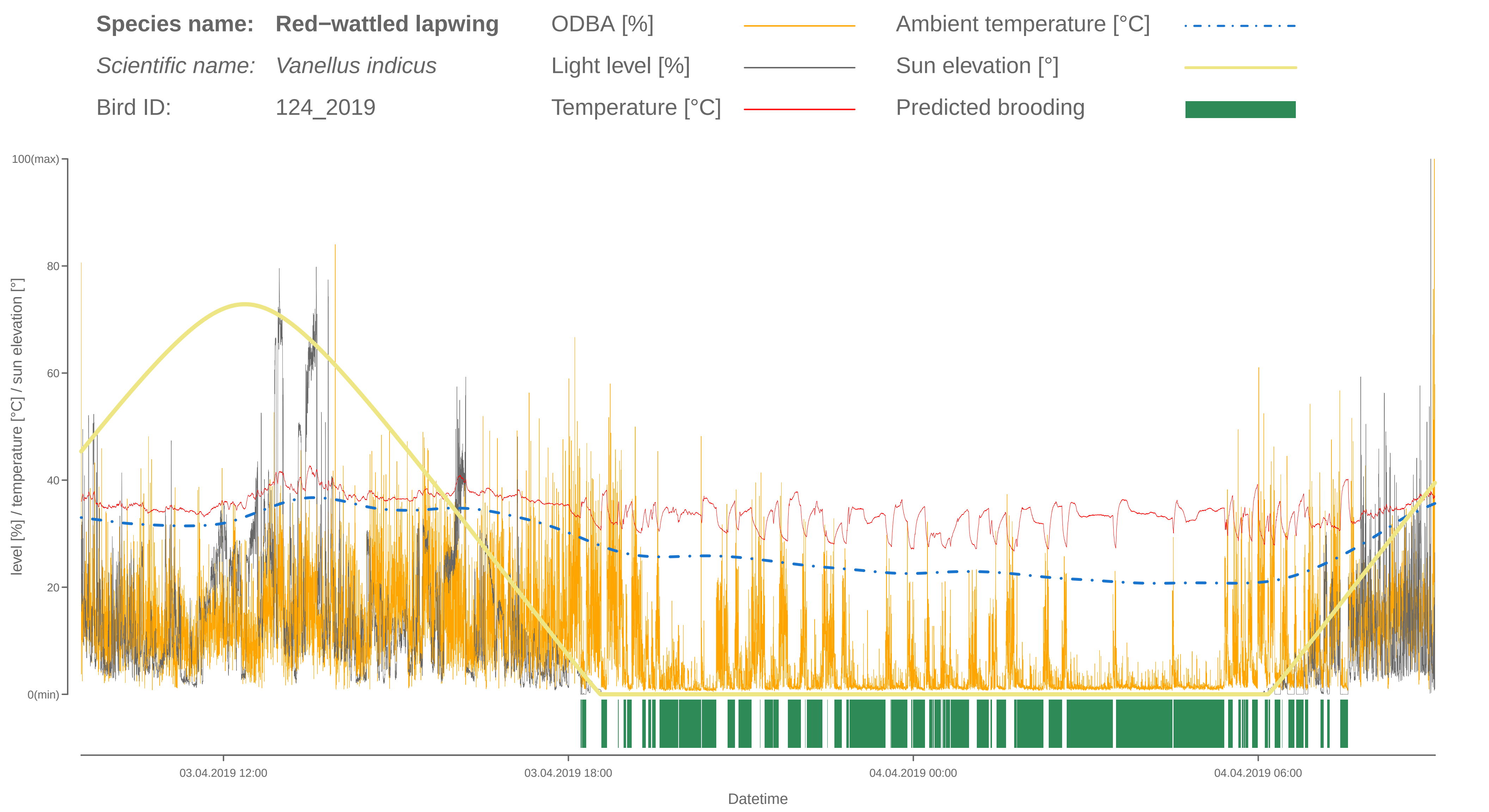

Species name: Red−wattled lapwing
Scientific name: Vanellus indicus
Bird ID: 124_2019
ODBA [%]
Light level [%]
Temperature [°C]
Ambient temperature [°C]
Sun elevation [°]
Predicted brooding
100(max)
80
60
40
20
0(min)
level [%] / temperature [°C] / sun elevation [°]
03.04.2019 12:00
03.04.2019 18:00
04.04.2019 00:00
04.04.2019 06:00
Datetime

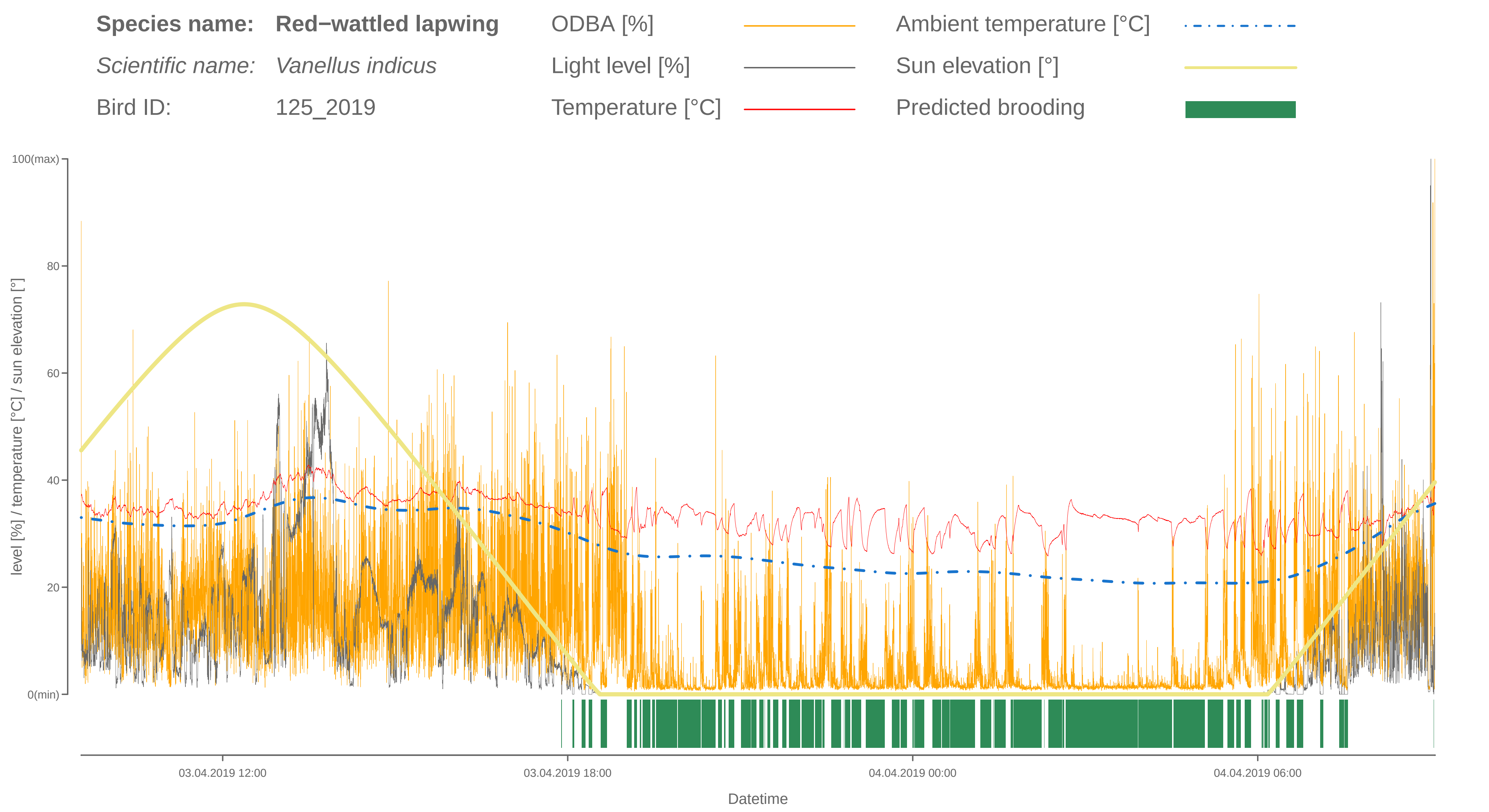
Species name: Red−wattled lapwing
Scientific name: Vanellus indicus
Bird ID: 125_2019
ODBA [%]
Light level [%]
Temperature [°C]
Ambient temperature [°C]
Sun elevation [°]
Predicted brooding
100(max)
80
60
40
20
0(min)
level [%] / temperature [°C] / sun elevation [°]
03.04.2019 12:00
03.04.2019 18:00
04.04.2019 00:00
04.04.2019 06:00
Datetime

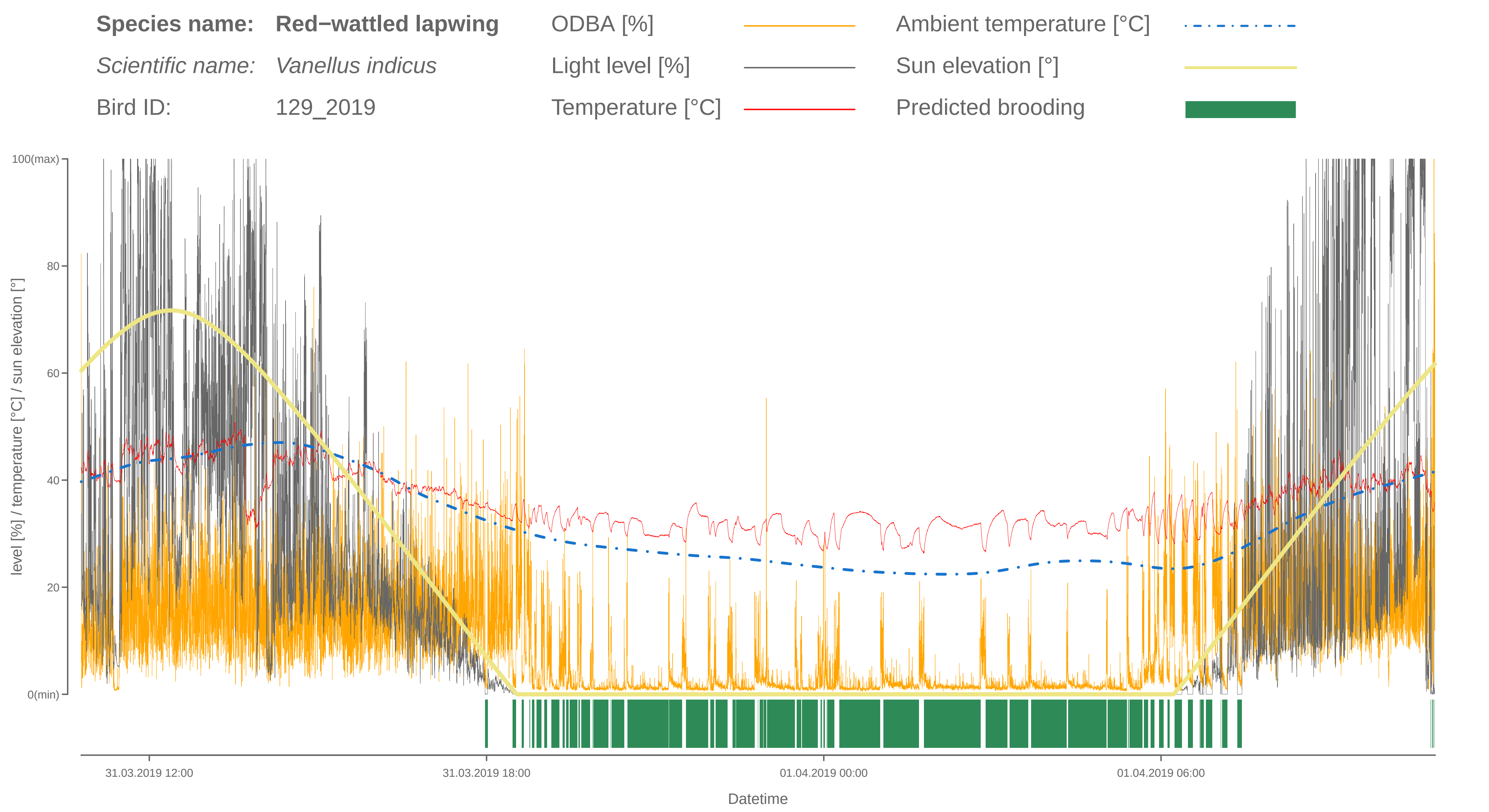
Species name: Red−wattled lapwing
Scientific name: Vanellus indicus
Bird ID: 129_2019
ODBA [%]
Light level [%]
Temperature [°C]
Ambient temperature [°C]
Sun elevation [°]
Predicted brooding
100(max)
80
60
40
20
0(min)
level [%] / temperature [°C] / sun elevation [°]
31.03.2019 12:00
31.03.2019 18:00
01.04.2019 00:00
01.04.2019 06:00
Datetime

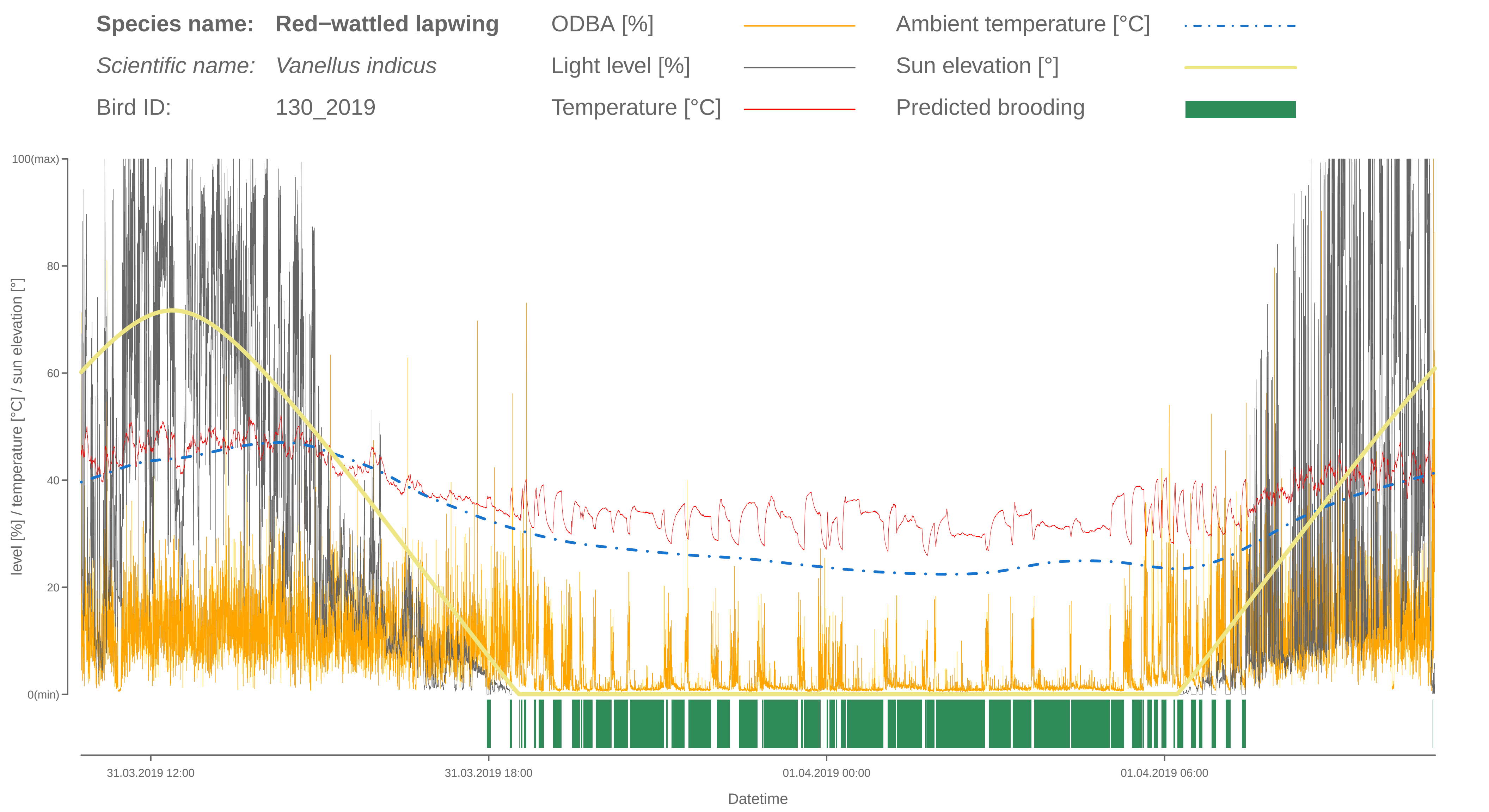
Species name: Red−wattled lapwing
Scientific name: Vanellus indicus
Bird ID: 130_2019
ODBA [%]
Light level [%]
Temperature [°C]
Ambient temperature [°C]
Sun elevation [°]
Predicted brooding
100(max)
80
60
40
20
0(min)
level [%] / temperature [°C] / sun elevation [°]
31.03.2019 12:00
31.03.2019 18:00
01.04.2019 00:00
01.04.2019 06:00
Datetime

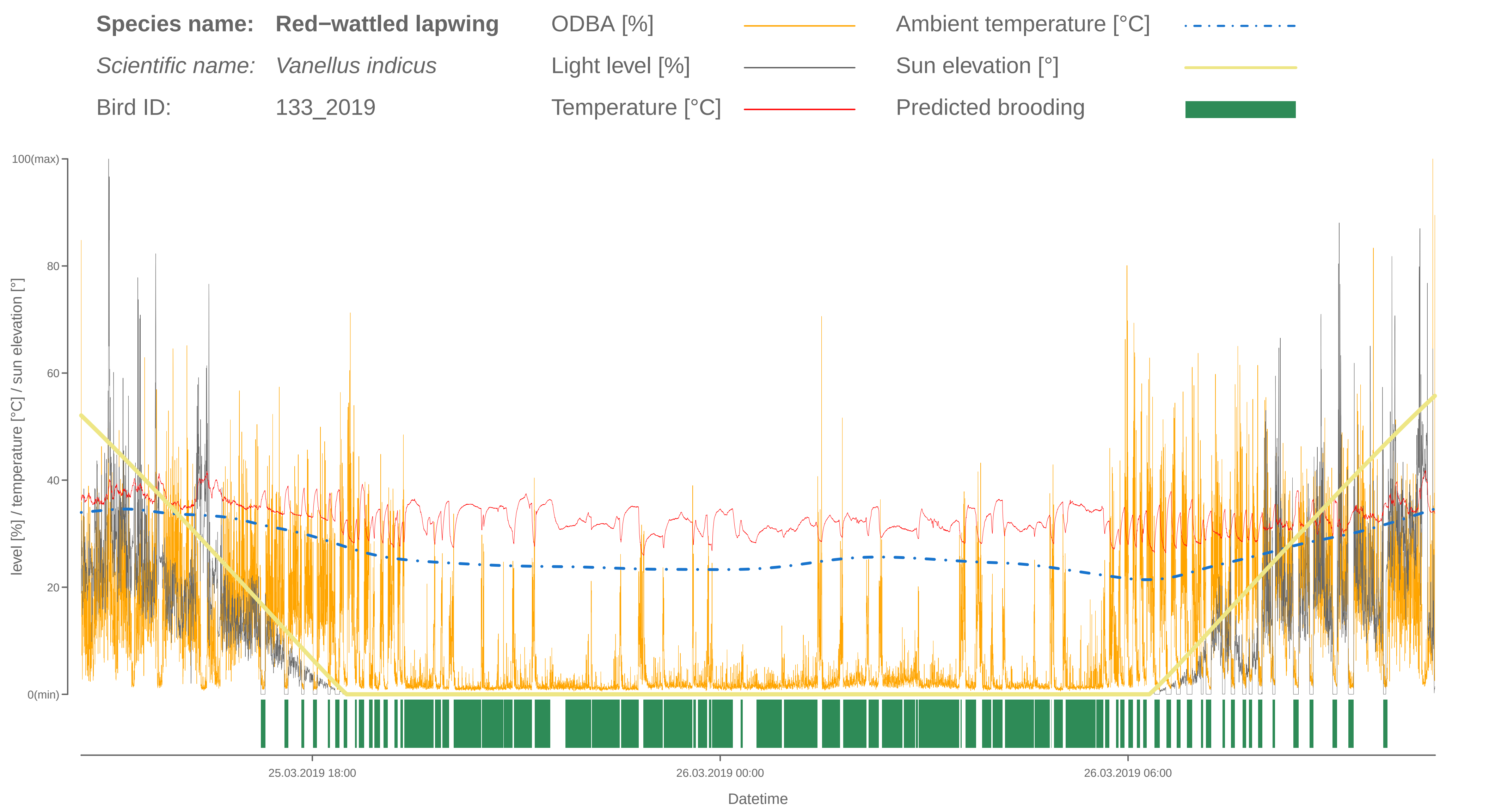
Species name: Red−wattled lapwing
Scientific name: Vanellus indicus
Bird ID: 133_2019
ODBA [%]
Light level [%]
Temperature [°C]
Ambient temperature [°C]
Sun elevation [°]
Predicted brooding
100(max)
80
60
40
20
0(min)
level [%] / temperature [°C] / sun elevation [°]
25.03.2019 18:00
26.03.2019 00:00
26.03.2019 06:00
Datetime

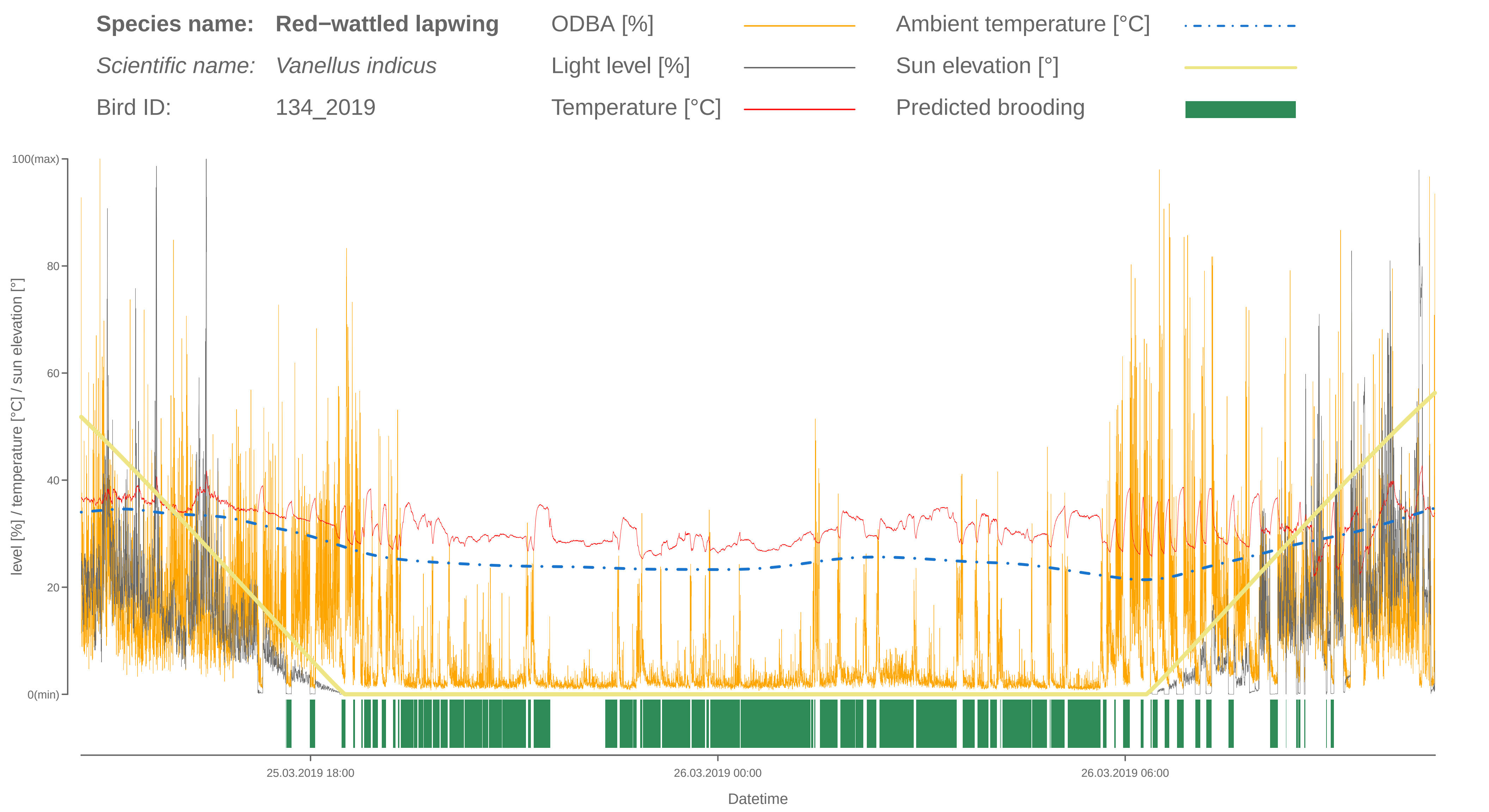
Species name: Red−wattled lapwing
Scientific name: Vanellus indicus
Bird ID: 134_2019
ODBA [%]
Light level [%]
Temperature [°C]
Ambient temperature [°C]
Sun elevation [°]
Predicted brooding
100(max)
80
60
40
20
0(min)
level [%] / temperature [°C] / sun elevation [°]
25.03.2019 18:00
26.03.2019 00:00
26.03.2019 06:00
Datetime

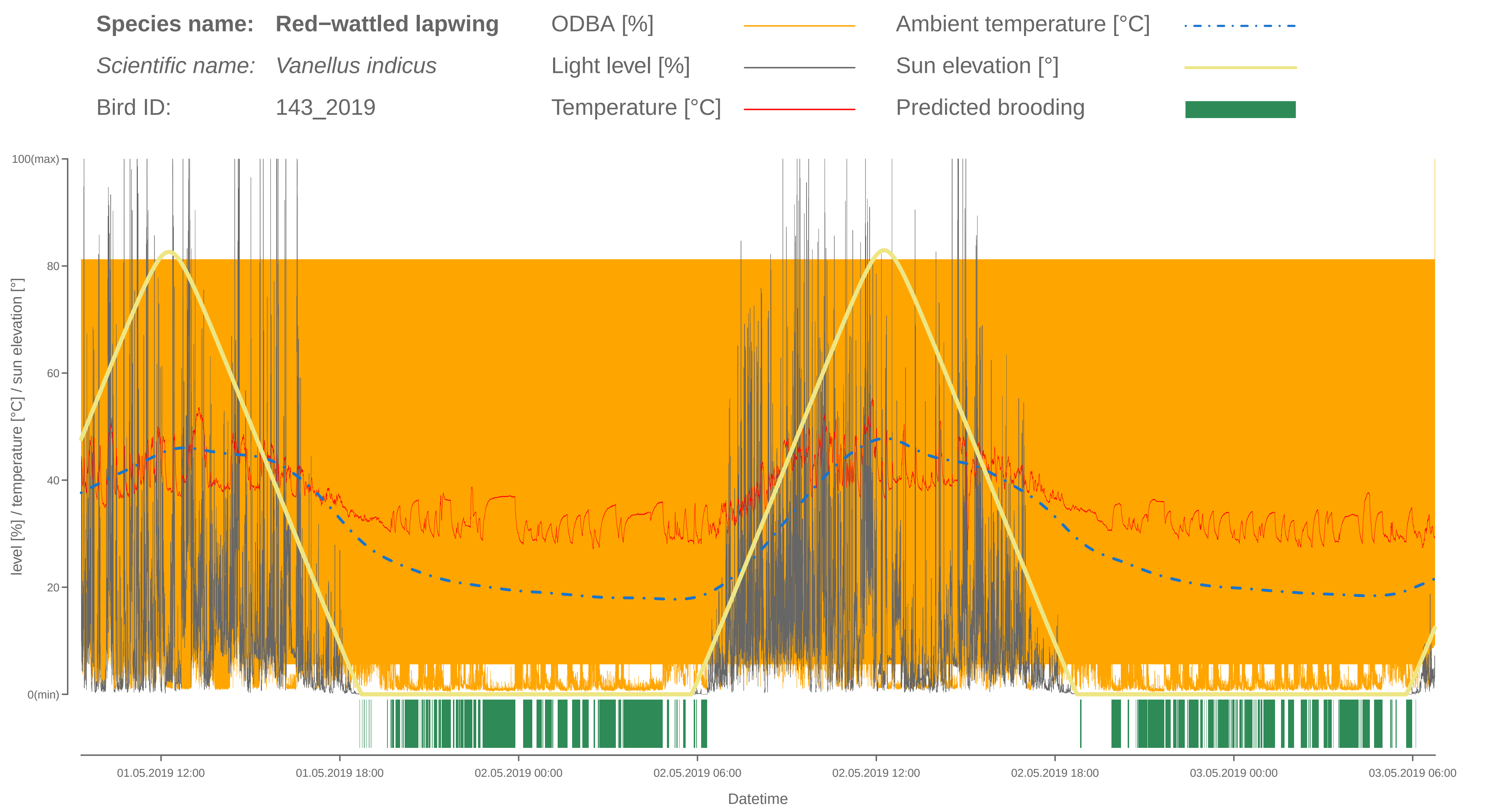
Species name: Red−wattled lapwing
Scientific name: Vanellus indicus
Bird ID: 143_2019
ODBA [%]
Light level [%]
Temperature [°C]
Ambient temperature [°C]
Sun elevation [°]
Predicted brooding
100(max)
80
60
40
20
0(min)
level [%] / temperature [°C] / sun elevation [°]
01.05.2019 12:00
01.05.2019 18:00
02.05.2019 00:00
02.05.2019 06:00
02.05.2019 12:00
02.05.2019 18:00
03.05.2019 00:00
03.05.2019 06:00
Datetime

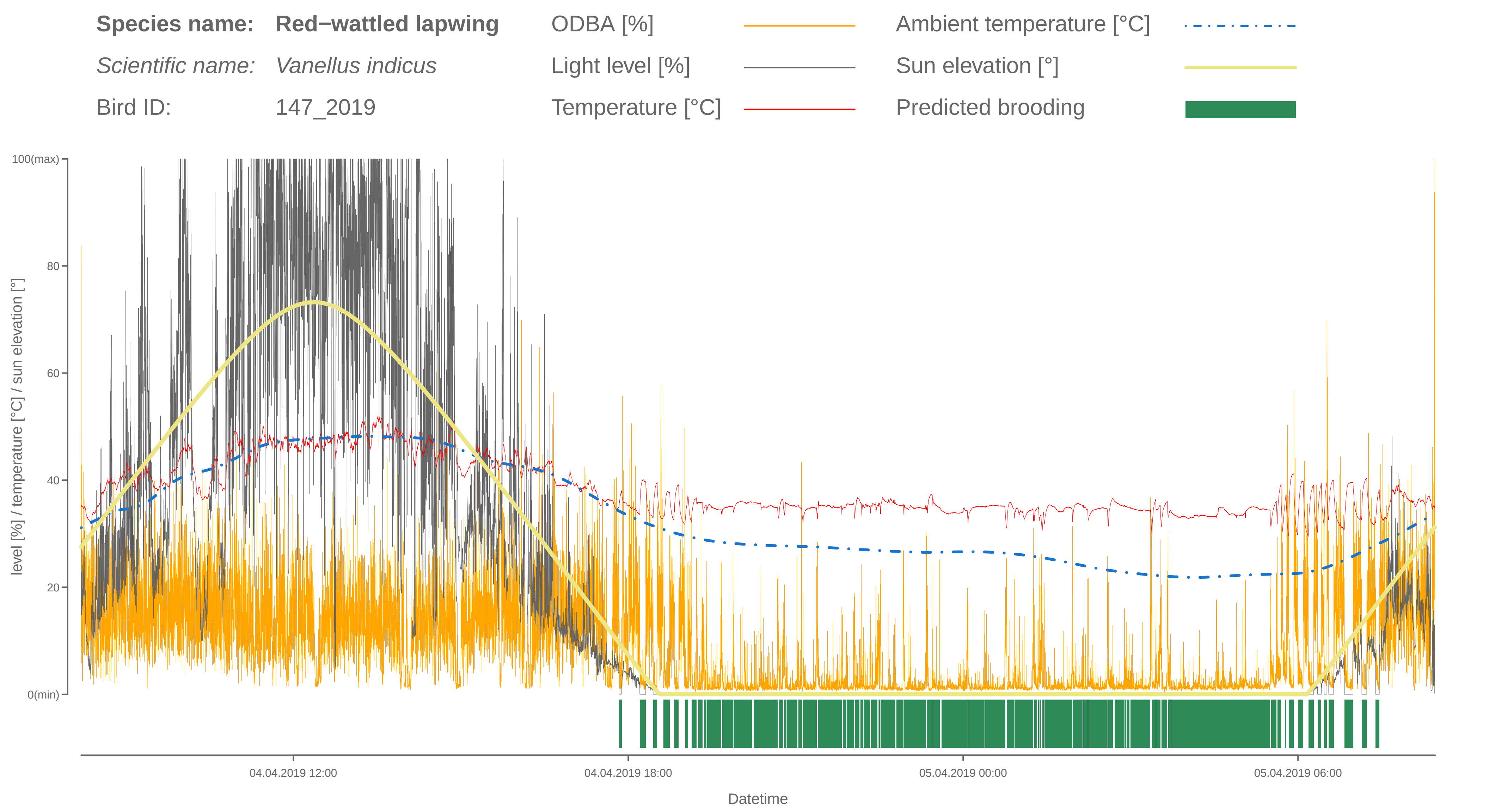

Species name: Red−wattled lapwing
Scientific name: Vanellus indicus
Bird ID: 147_2019
ODBA [%]
Light level [%]
Temperature [°C]
Ambient temperature [°C]
Sun elevation [°]
Predicted brooding
level [%] / temperature [°C] / sun elevation [°]
100(max)
80
60
40
20
0(min)
04.04.2019 12:00
04.04.2019 18:00
05.04.2019 00:00
05.04.2019 06:00
Datetime

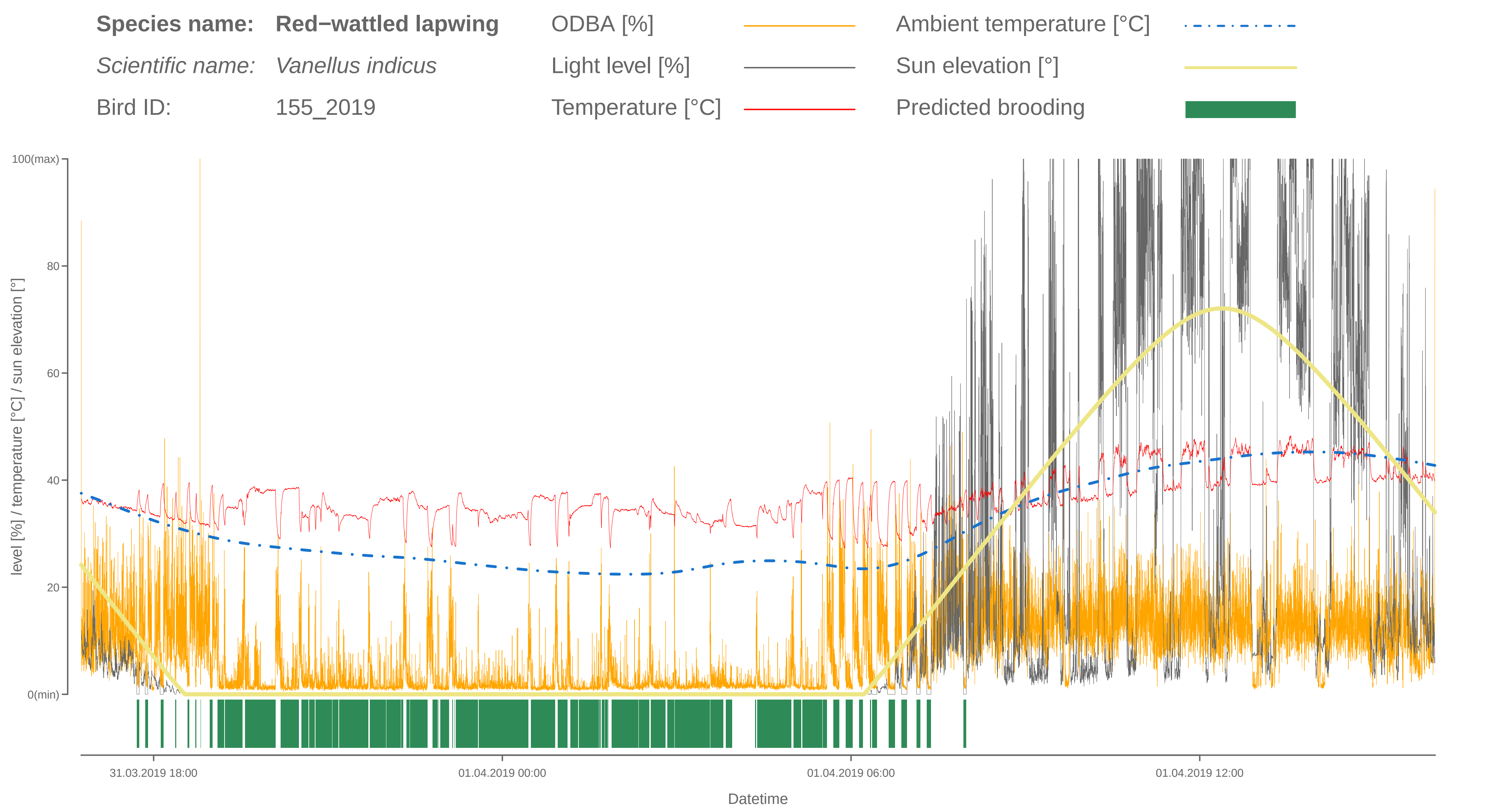

Species name: Red−wattled lapwing
Scientific name: Vanellus indicus
Bird ID: 155_2019
ODBA [%]
Light level [%]
Temperature [°C]
Ambient temperature [°C]
Sun elevation [°]
Predicted brooding
100(max)
80
60
40
20
0(min)
level [%] / temperature [°C] / sun elevation [°]
31.03.2019 18:00
01.04.2019 00:00
01.04.2019 06:00
01.04.2019 12:00
Datetime

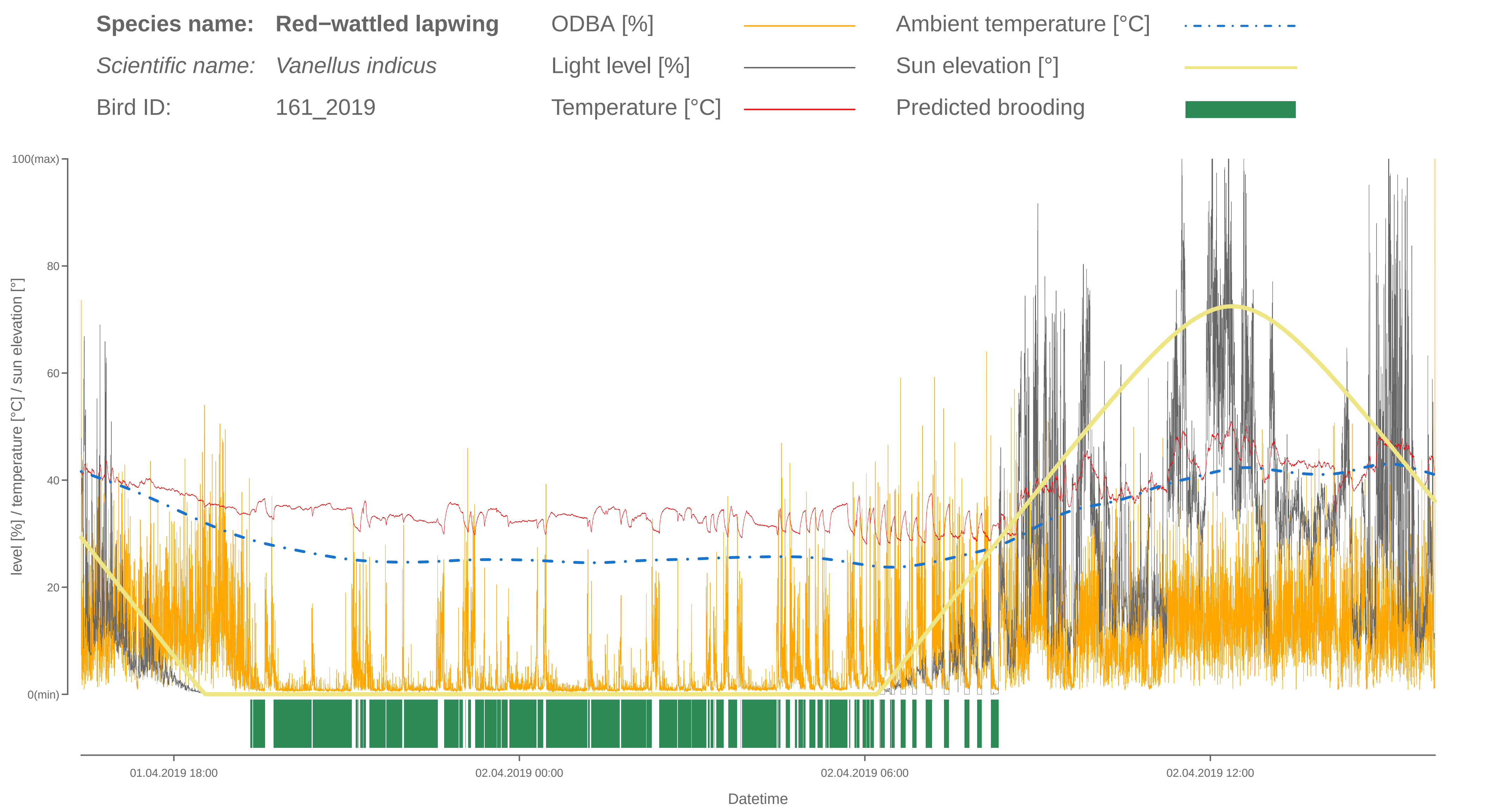

Species name: Red−wattled lapwing
Scientific name: Vanellus indicus
Bird ID: 161_2019
ODBA [%]
Light level [%]
Temperature [°C]
Ambient temperature [°C]
Sun elevation [°]
Predicted brooding
100(max)
80
60
40
20
0(min)
level [%] / temperature [°C] / sun elevation [°]
01.04.2019 18:00
02.04.2019 00:00
02.04.2019 06:00
02.04.2019 12:00
Datetime

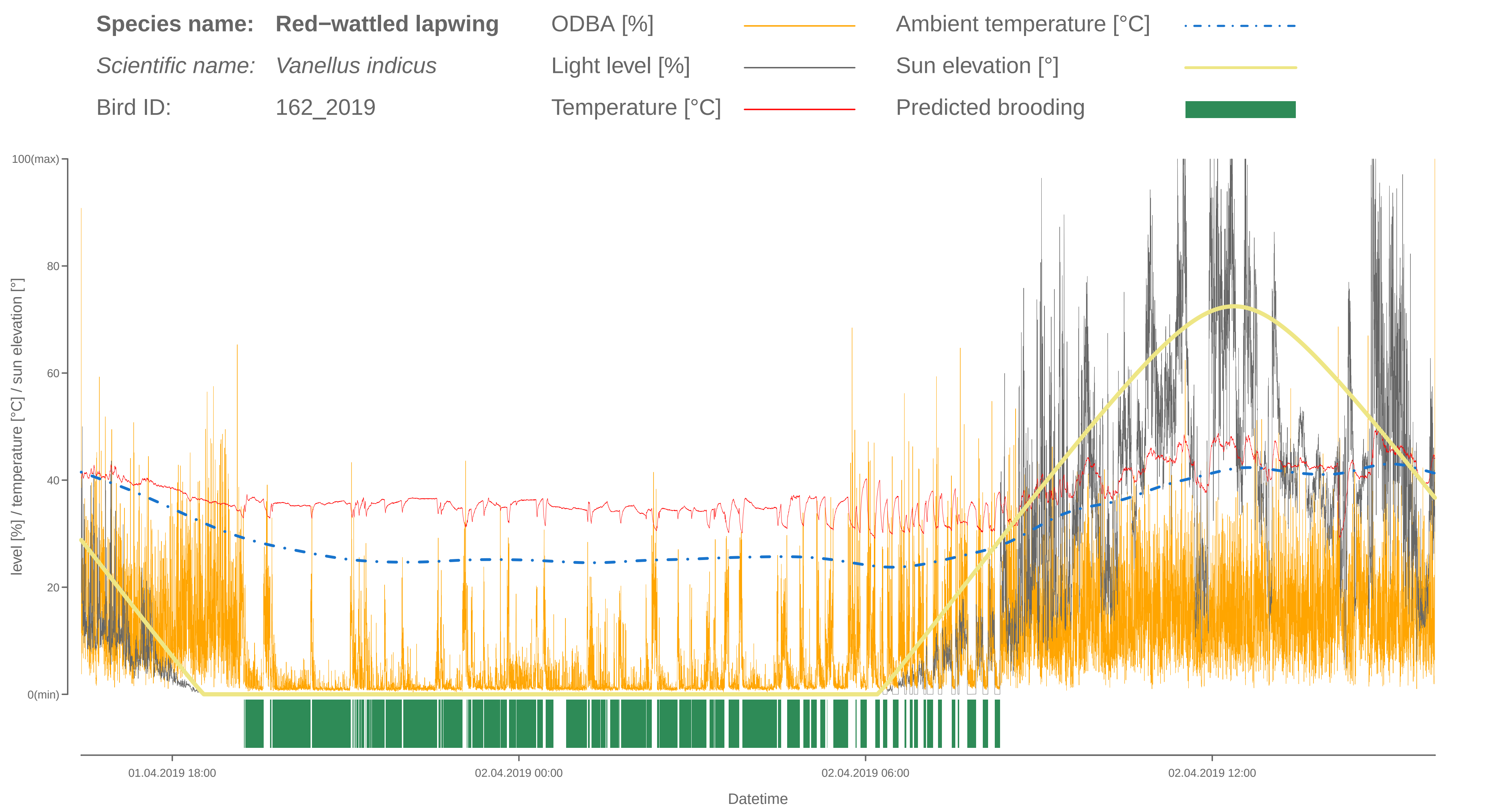
Species name: Red−wattled lapwing
Scientific name: Vanellus indicus
Bird ID: 162_2019
ODBA [%]
Light level [%]
Temperature [°C]
Ambient temperature [°C]
Sun elevation [°]
Predicted brooding
100(max)
80
60
40
20
0(min)
level [%] / temperature [°C] / sun elevation [°]
01.04.2019 18:00
02.04.2019 00:00
02.04.2019 06:00
02.04.2019 12:00
Datetime

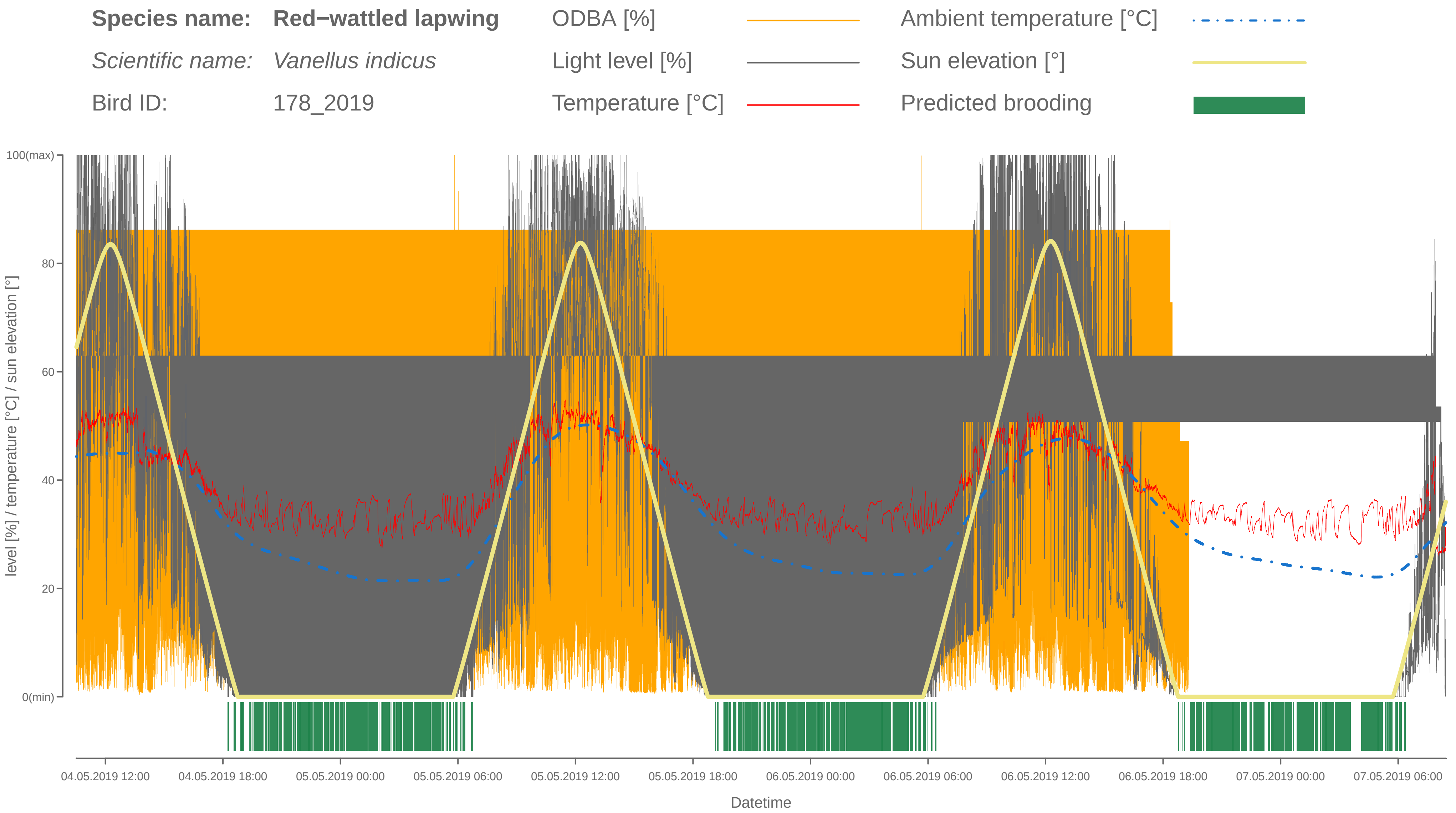
Species name: Red−wattled lapwing
Scientific name: Vanellus indicus
Bird ID: 178_2019
ODBA [%]
Light level [%]
Temperature [°C]
Ambient temperature [°C]
Sun elevation [°]
Predicted brooding
100(max)
80
60
40
20
0(min)
level [%] / temperature [°C] / sun elevation [°]
04.05.2019 12:00
04.05.2019 18:00
05.05.2019 00:00
05.05.2019 06:00
05.05.2019 12:00
05.05.2019 18:00
06.05.2019 00:00
06.05.2019 06:00
06.05.2019 12:00
06.05.2019 18:00
07.05.2019 00:00
07.05.2019 06:00
Datetime

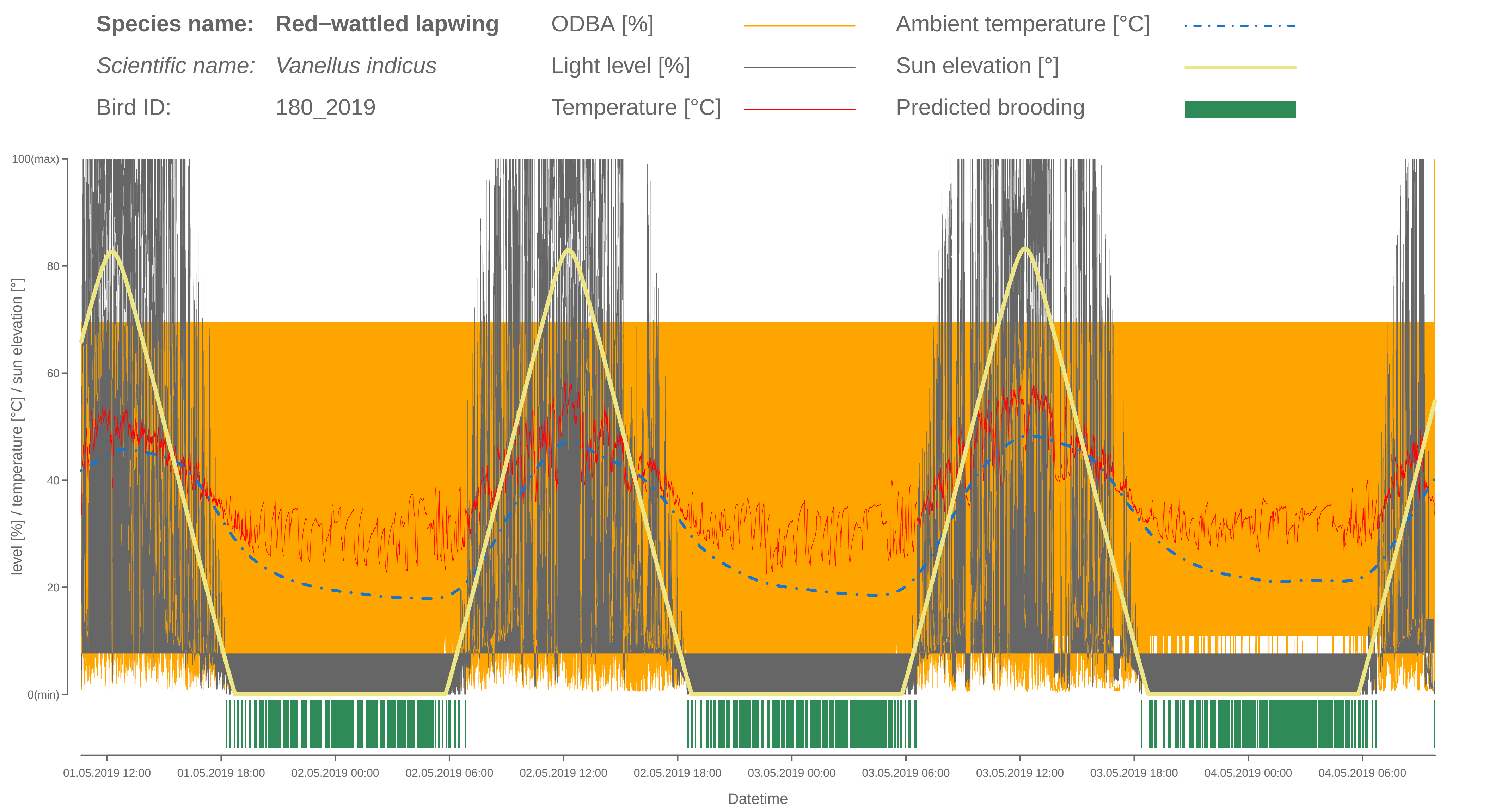
Species name: Red−wattled lapwing
Scientific name: Vanellus indicus
Bird ID: 180_2019
ODBA [%]
Light level [%]
Temperature [°C]
Ambient temperature [°C]
Sun elevation [°]
Predicted brooding
100(max)
80
60
40
20
0(min)
level [%] / temperature [°C] / sun elevation [°]
01.05.2019 12:00
01.05.2019 18:00
02.05.2019 00:00
02.05.2019 06:00
02.05.2019 12:00
02.05.2019 18:00
03.05.2019 00:00
03.05.2019 06:00
03.05.2019 12:00
03.05.2019 18:00
04.05.2019 00:00
04.05.2019 06:00
Datetime

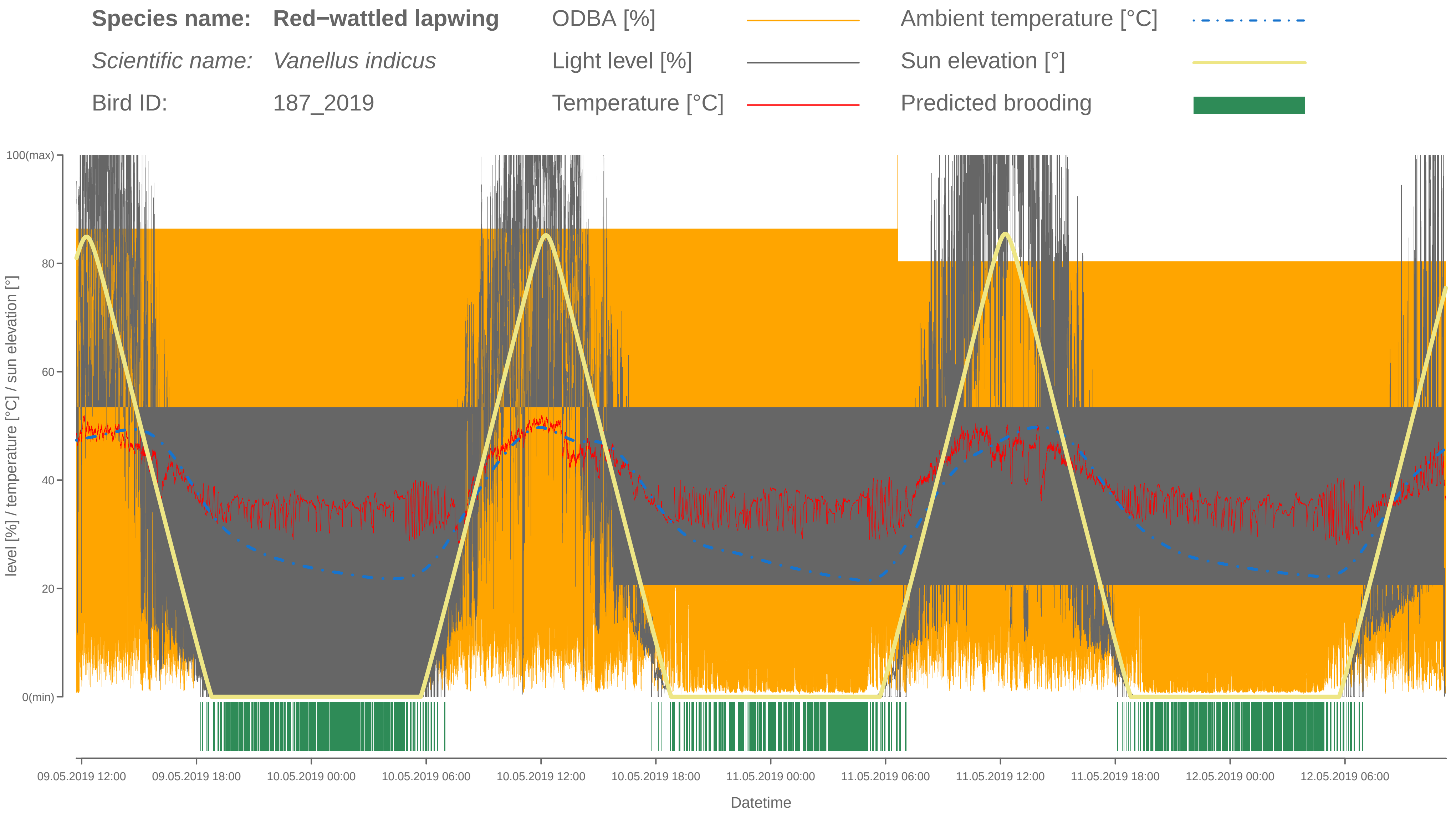
Species name: Red−wattled lapwing
Scientific name: Vanellus indicus
Bird ID: 187_2019
ODBA [%]
Light level [%]
Temperature [°C]
Ambient temperature [°C]
Sun elevation [°]
Predicted brooding
level [%] / temperature [°C] / sun elevation [°]
100(max)
80
60
40
20
0(min)
09.05.2019 12:00
09.05.2019 18:00
10.05.2019 00:00
10.05.2019 06:00
10.05.2019 12:00
10.05.2019 18:00
11.05.2019 00:00
11.05.2019 06:00
11.05.2019 12:00
11.05.2019 18:00
12.05.2019 00:00
12.05.2019 06:00
Datetime

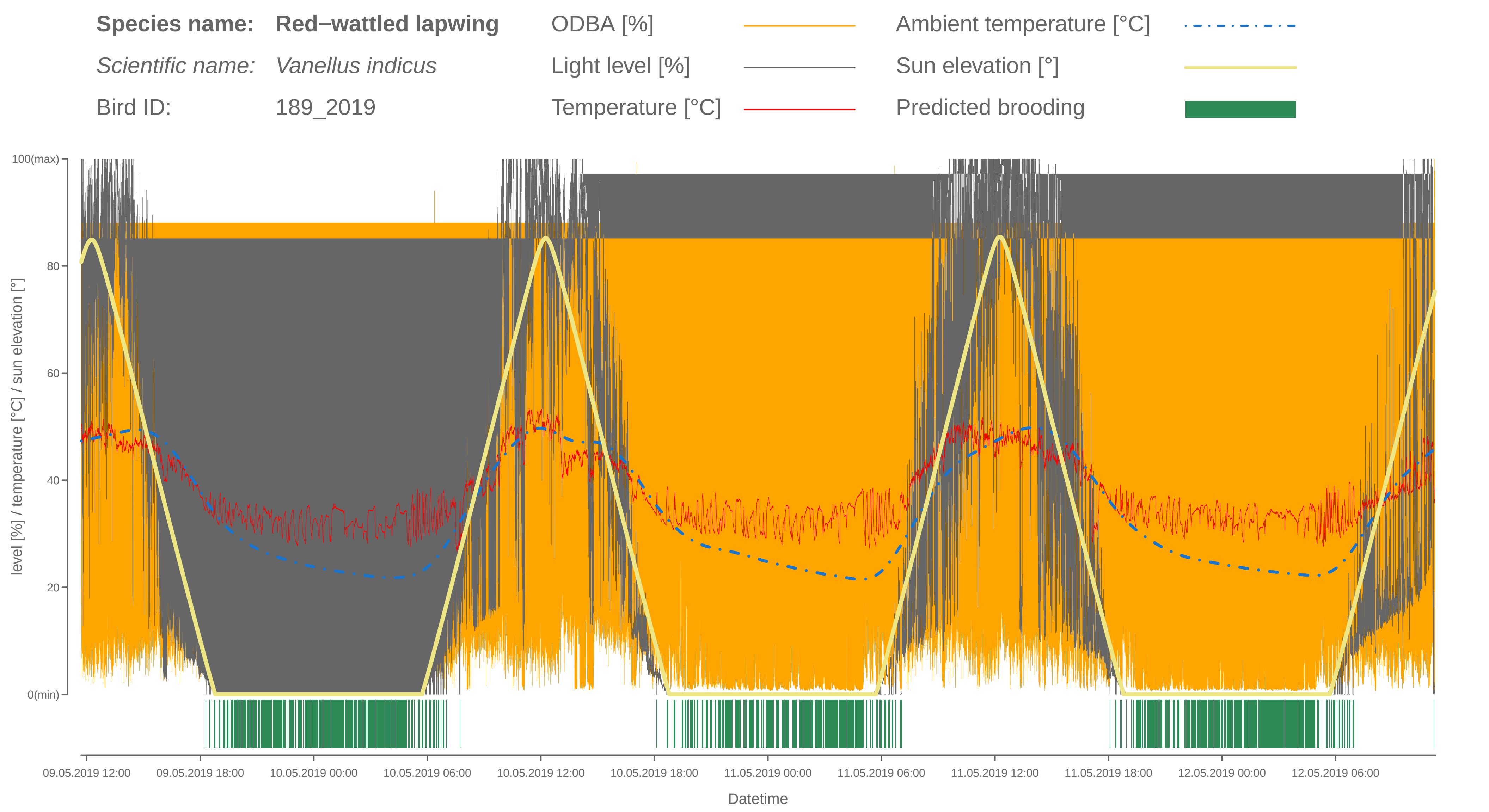

Species name: Red−wattled lapwing
Scientific name: Vanellus indicus
Bird ID: 189_2019
ODBA [%]
Light level [%]
Temperature [°C]
Ambient temperature [°C]
Sun elevation [°]
Predicted brooding
100(max)
80
60
40
20
0(min)
level [%] / temperature [°C] / sun elevation [°]
09.05.2019 12:00
09.05.2019 18:00
10.05.2019 00:00
10.05.2019 06:00
10.05.2019 12:00
10.05.2019 18:00
11.05.2019 00:00
11.05.2019 06:00
11.05.2019 12:00
11.05.2019 18:00
12.05.2019 00:00
12.05.2019 06:00
Datetime

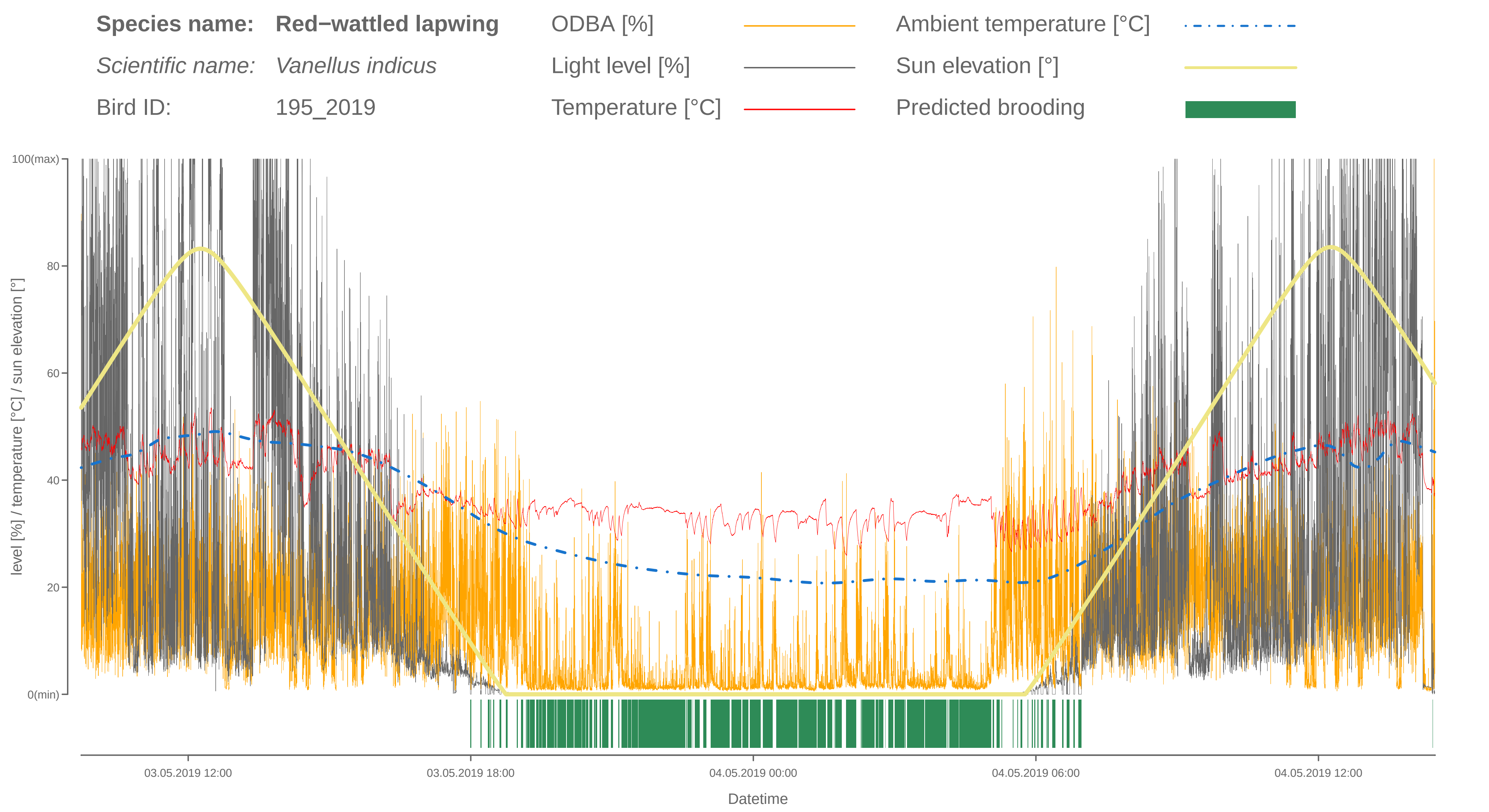
Species name: Red−wattled lapwing
Scientific name: Vanellus indicus
Bird ID: 195_2019
ODBA [%]
Light level [%]
Temperature [°C]
Ambient temperature [°C]
Sun elevation [°]
Predicted brooding
100(max)
80
60
40
20
0(min)
level [%] / temperature [°C] / sun elevation [°]
03.05.2019 12:00
03.05.2019 18:00
04.05.2019 00:00
04.05.2019 06:00
04.05.2019 12:00
Datetime

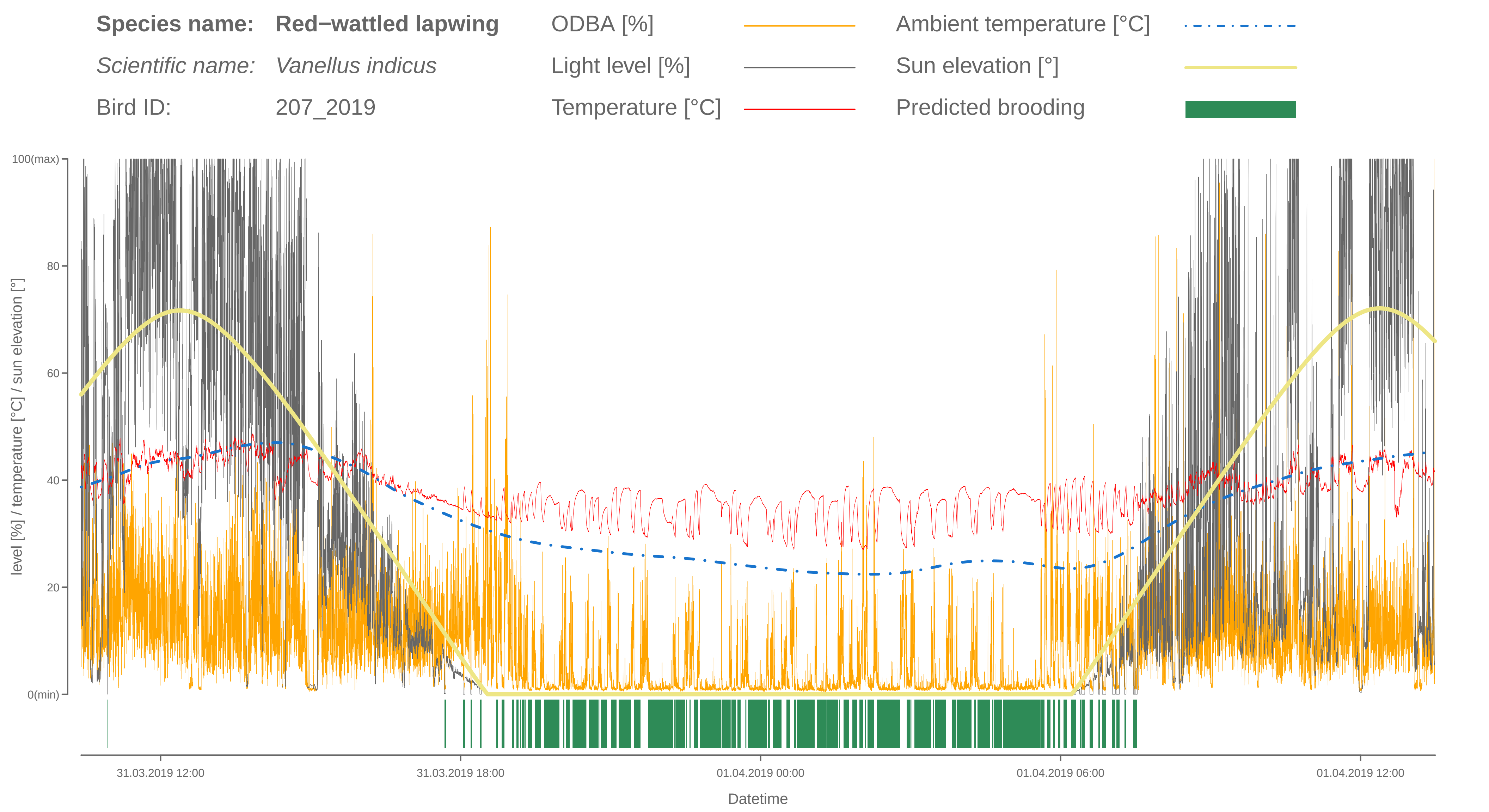
Species name: Red−wattled lapwing
Scientific name: Vanellus indicus
Bird ID: 207_2019
ODBA [%]
Light level [%]
Temperature [°C]
Ambient temperature [°C]
Sun elevation [°]
Predicted brooding
level [%] / temperature [°C] / sun elevation [°]
100(max)
80
60
40
20
0(min)
31.03.2019 12:00
31.03.2019 18:00
01.04.2019 00:00
01.04.2019 06:00
01.04.2019 12:00
Datetime

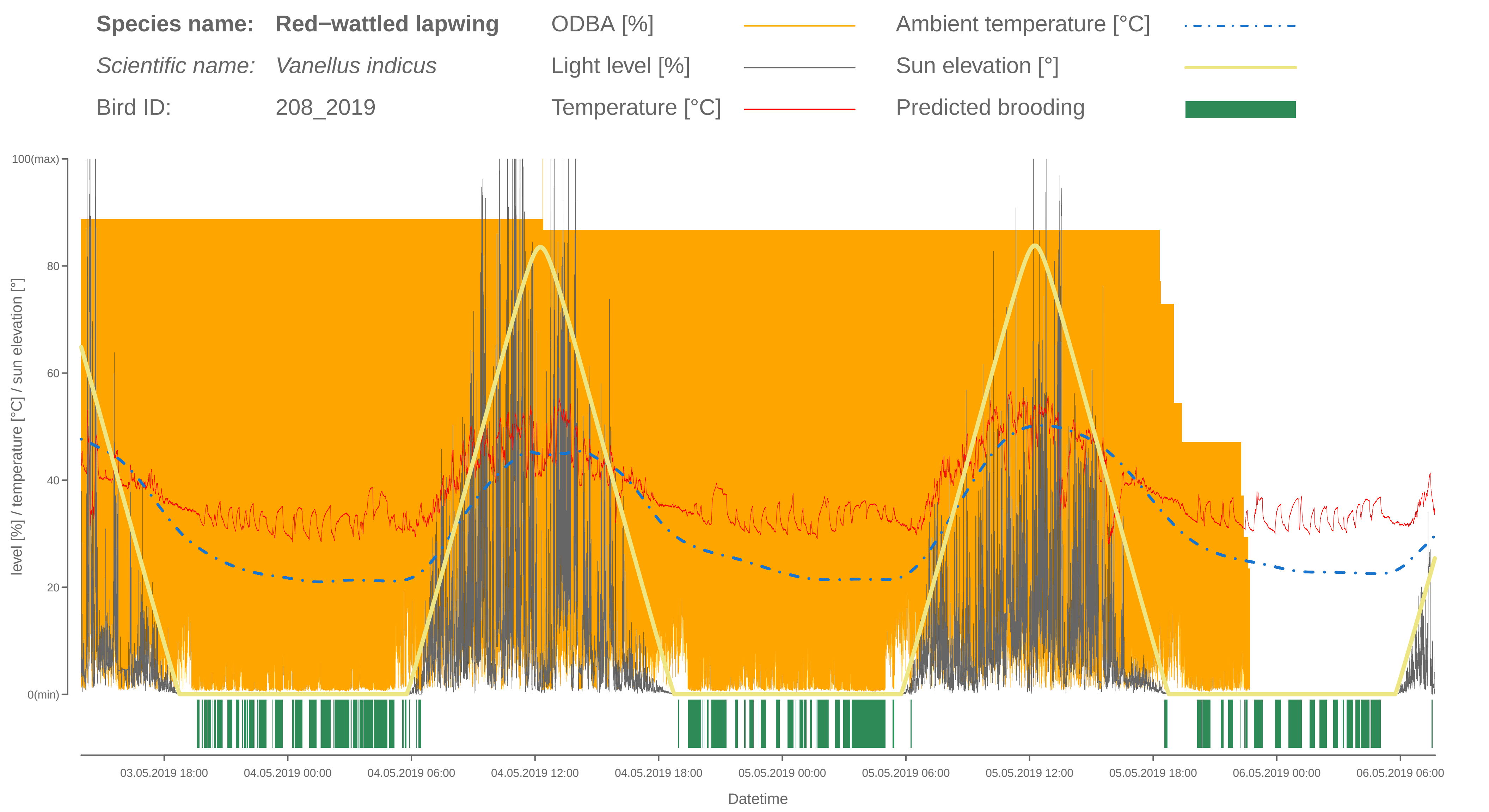

Species name: Red−wattled lapwing
Scientific name: Vanellus indicus
Bird ID: 208_2019
ODBA [%]
Light level [%]
Temperature [°C]
Ambient temperature [°C]
Sun elevation [°]
Predicted brooding
100(max)
80
60
40
20
0(min)
level [%] / temperature [°C] / sun elevation [°]
03.05.2019 18:00
04.05.2019 00:00
04.05.2019 06:00
04.05.2019 12:00
04.05.2019 18:00
05.05.2019 00:00
05.05.2019 06:00
05.05.2019 12:00
05.05.2019 18:00
06.05.2019 00:00
06.05.2019 06:00
Datetime

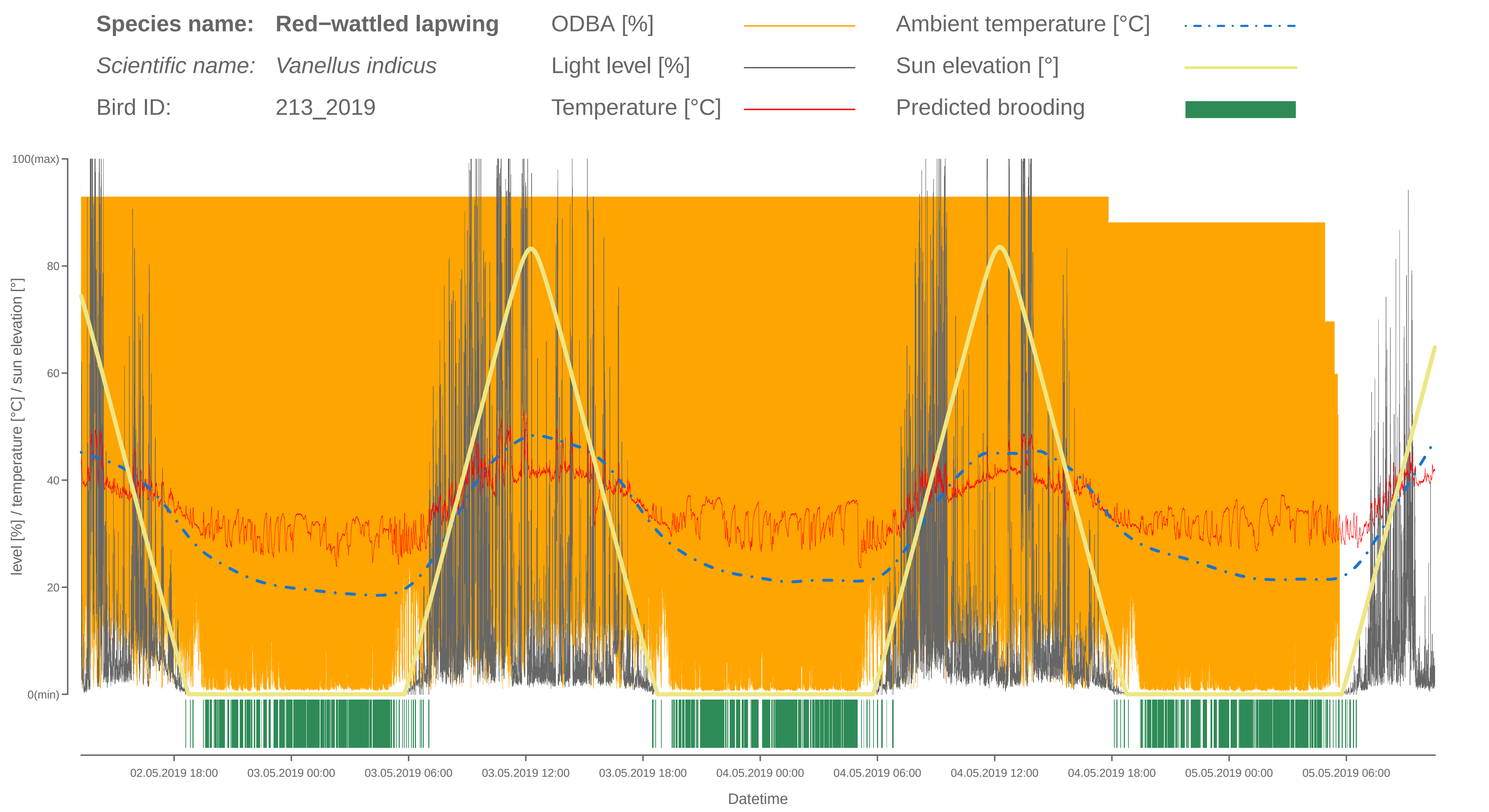

Species name: Red−wattled lapwing
Scientific name: Vanellus indicus
Bird ID: 213_2019
ODBA [%]
Light level [%]
Temperature [°C]
Ambient temperature [°C]
Sun elevation [°]
Predicted brooding
100(max)
80
60
40
20
0(min)
level [%] / temperature [°C] / sun elevation [°]
02.05.2019 18:00
03.05.2019 00:00
03.05.2019 06:00
03.05.2019 12:00
03.05.2019 18:00
04.05.2019 00:00
04.05.2019 06:00
04.05.2019 12:00
04.05.2019 18:00
05.05.2019 00:00
05.05.2019 06:00
Datetime

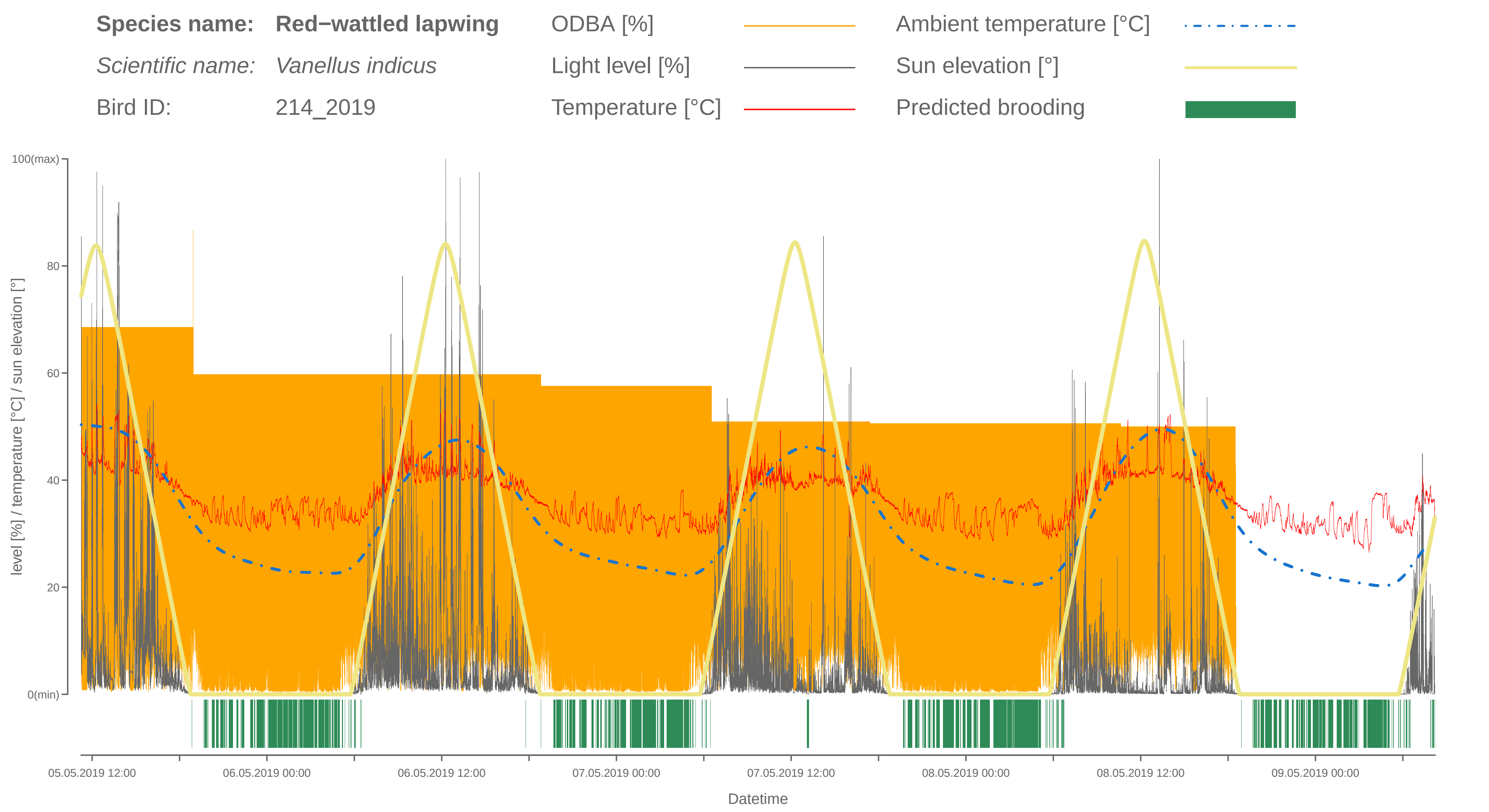
Species name: Red−wattled lapwing
Scientific name: Vanellus indicus
Bird ID: 214_2019
ODBA [%]
Light level [%]
Temperature [°C]
Ambient temperature [°C]
Sun elevation [°]
Predicted brooding
100(max)
80
60
40
20
0(min)
level [%] / temperature [°C] / sun elevation [°]
05.05.2019 12:00
06.05.2019 00:00
06.05.2019 12:00
07.05.2019 00:00
07.05.2019 12:00
08.05.2019 00:00
08.05.2019 12:00
09.05.2019 00:00
Datetime

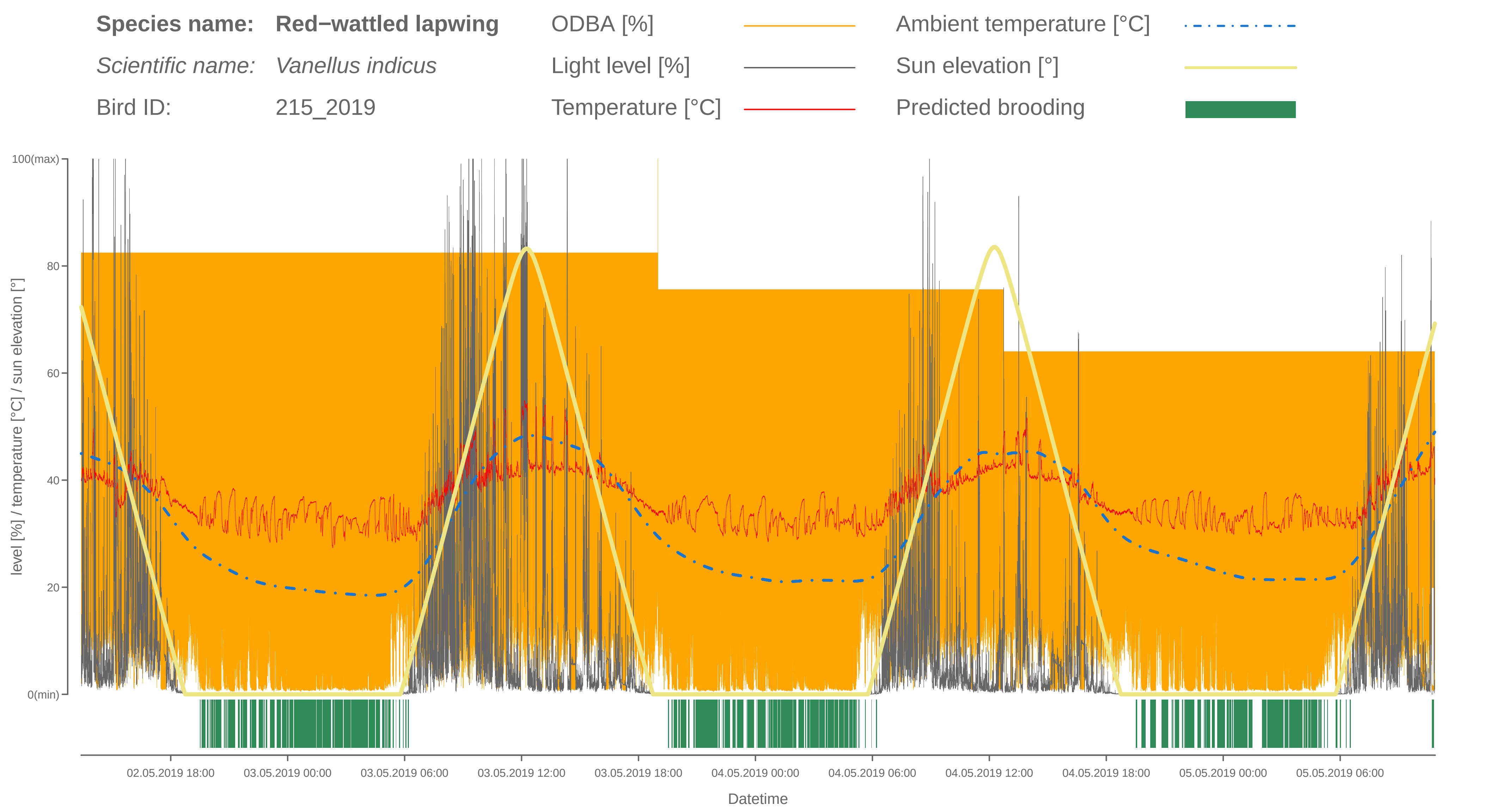
Species name: Red−wattled lapwing
Scientific name: Vanellus indicus
Bird ID: 215_2019
ODBA [%]
Light level [%]
Temperature [°C]
Ambient temperature [°C]
Sun elevation [°]
Predicted brooding
level [%] / temperature [°C] / sun elevation [°]
100(max)
80
60
40
20
0(min)
02.05.2019 18:00
03.05.2019 00:00
03.05.2019 06:00
03.05.2019 12:00
03.05.2019 18:00
04.05.2019 00:00
04.05.2019 06:00
04.05.2019 12:00
04.05.2019 18:00
05.05.2019 00:00
05.05.2019 06:00
Datetime

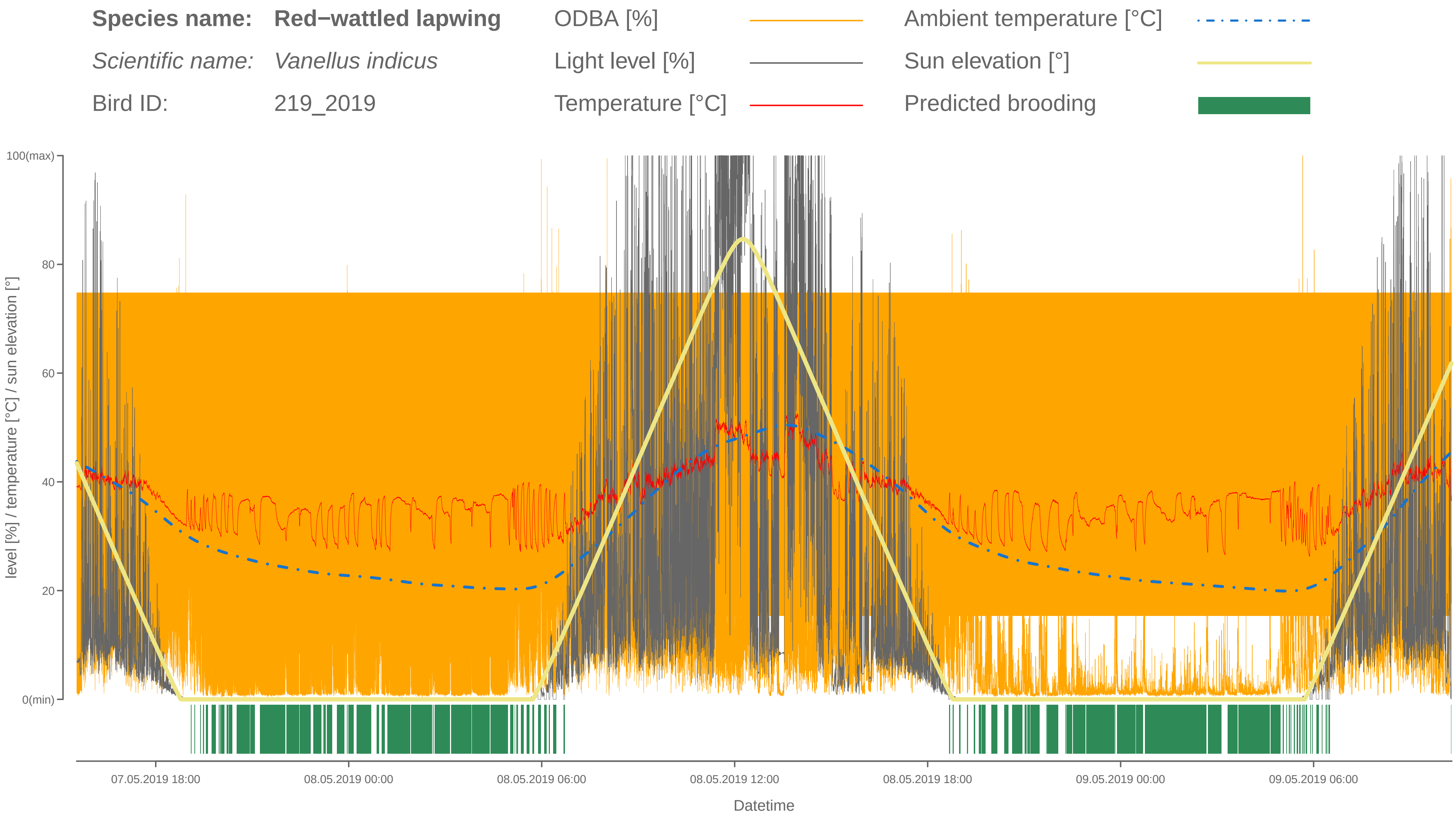
Species name: Red−wattled lapwing
Scientific name: Vanellus indicus
Bird ID: 219_2019
ODBA [%]
Light level [%]
Temperature [°C]
Ambient temperature [°C]
Sun elevation [°]
Predicted brooding
100(max)
80
60
40
20
0(min)
level [%] / temperature [°C] / sun elevation [°]
07.05.2019 18:00
08.05.2019 00:00
08.05.2019 06:00
08.05.2019 12:00
08.05.2019 18:00
09.05.2019 00:00
09.05.2019 06:00
Datetime

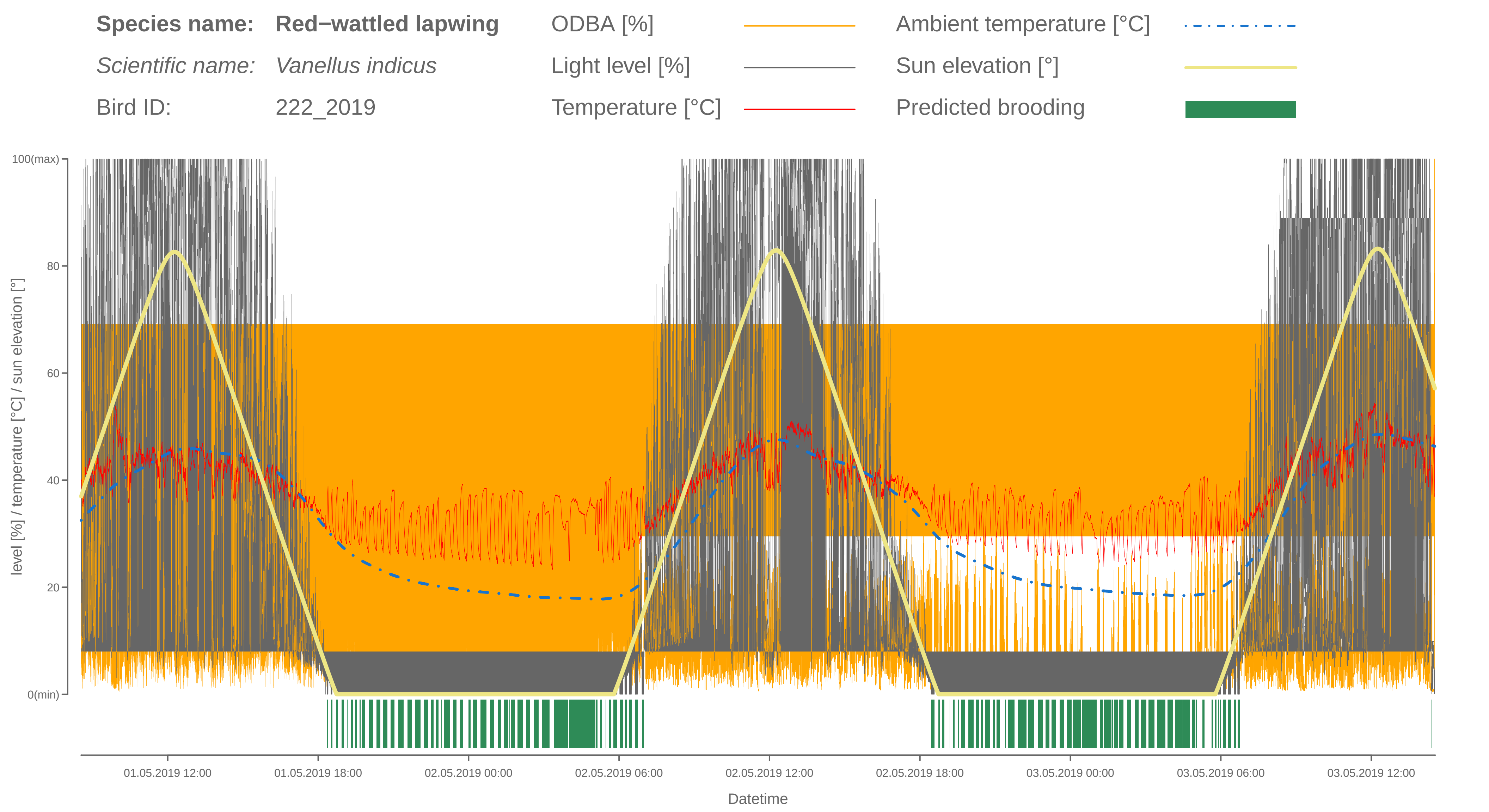
Species name: Red−wattled lapwing
Scientific name: Vanellus indicus
Bird ID: 222_2019
ODBA [%]
Light level [%]
Temperature [°C]
Ambient temperature [°C]
Sun elevation [°]
Predicted brooding
100(max)
80
60
40
20
0(min)
level [%] / temperature [°C] / sun elevation [°]
01.05.2019 12:00
01.05.2019 18:00
02.05.2019 00:00
02.05.2019 06:00
02.05.2019 12:00
02.05.2019 18:00
03.05.2019 00:00
03.05.2019 06:00
03.05.2019 12:00
Datetime

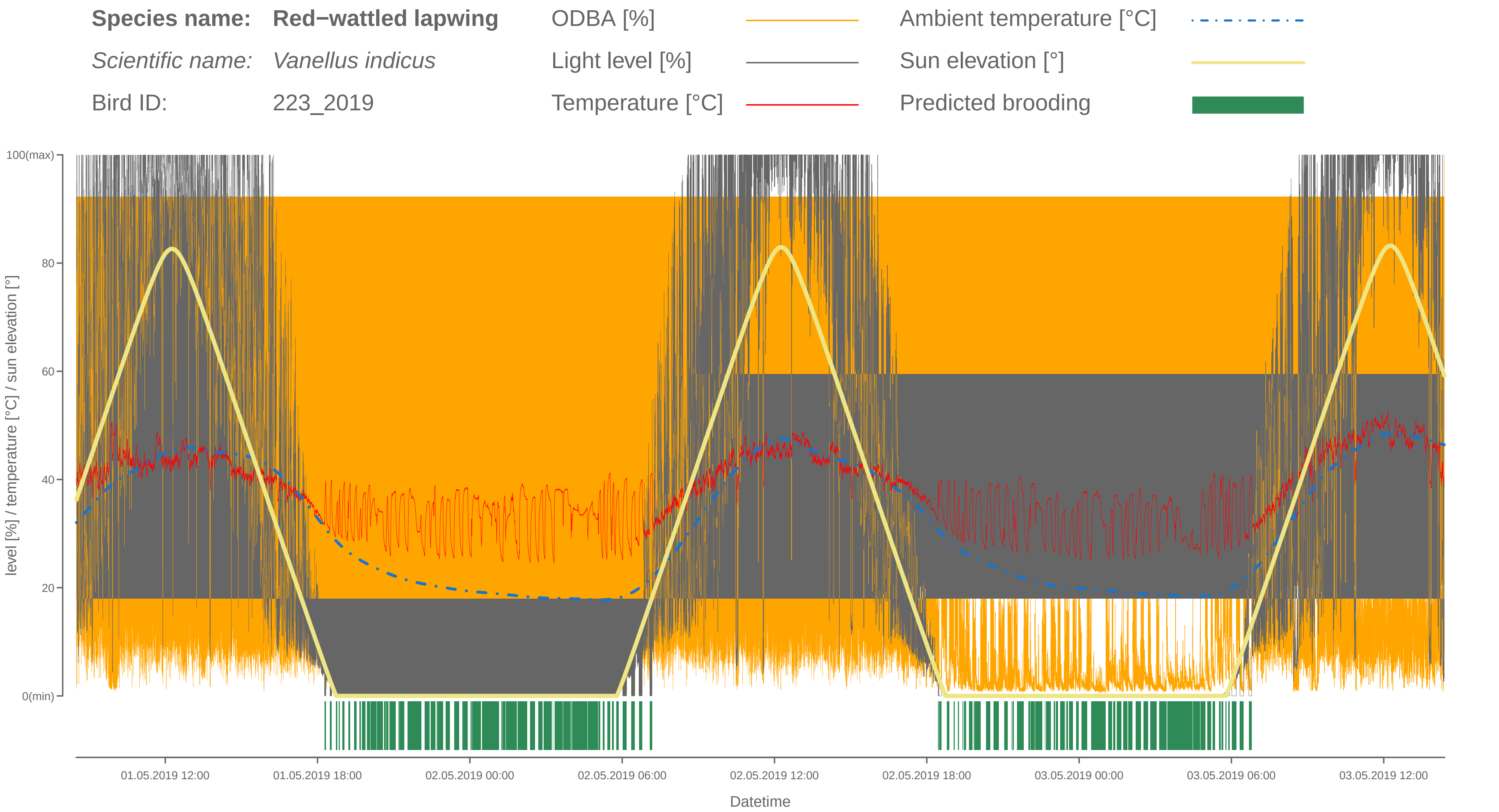

Species name: Red−wattled lapwing
Scientific name: Vanellus indicus
Bird ID: 223_2019
ODBA [%]
Light level [%]
Temperature [°C]
Ambient temperature [°C]
Sun elevation [°]
Predicted brooding
100(max)
80
60
40
20
0(min)
level [%] / temperature [°C] / sun elevation [°]
01.05.2019 12:00
01.05.2019 18:00
02.05.2019 00:00
02.05.2019 06:00
02.05.2019 12:00
02.05.2019 18:00
03.05.2019 00:00
03.05.2019 06:00
03.05.2019 12:00
Datetime

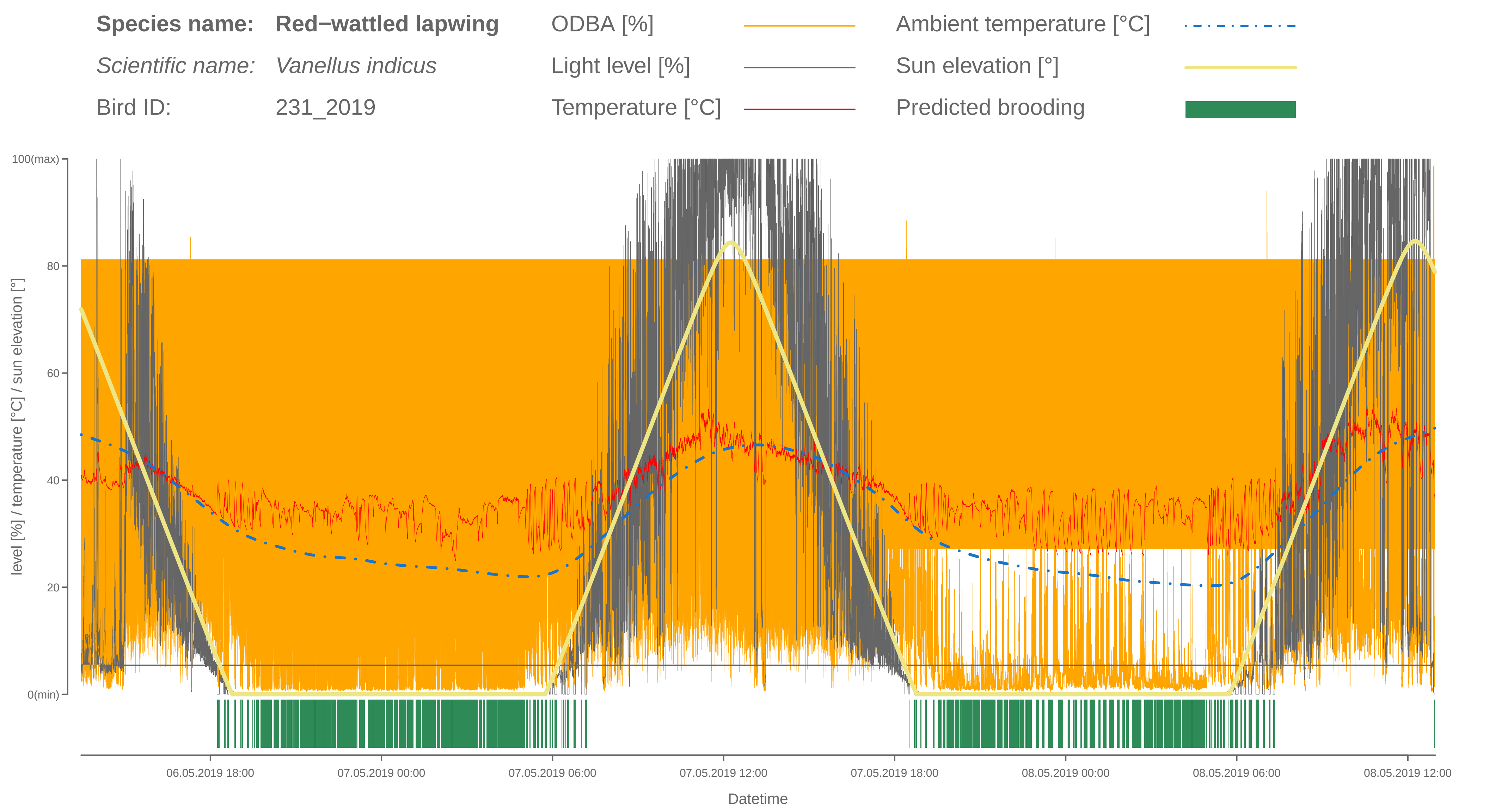
Species name: Red−wattled lapwing
Scientific name: Vanellus indicus
Bird ID: 231_2019
ODBA [%]
Light level [%]
Temperature [°C]
Ambient temperature [°C]
Sun elevation [°]
Predicted brooding
100(max)
80
60
40
20
0(min)
level [%] / temperature [°C] / sun elevation [°]
06.05.2019 18:00
07.05.2019 00:00
07.05.2019 06:00
07.05.2019 12:00
07.05.2019 18:00
08.05.2019 00:00
08.05.2019 06:00
08.05.2019 12:00
Datetime

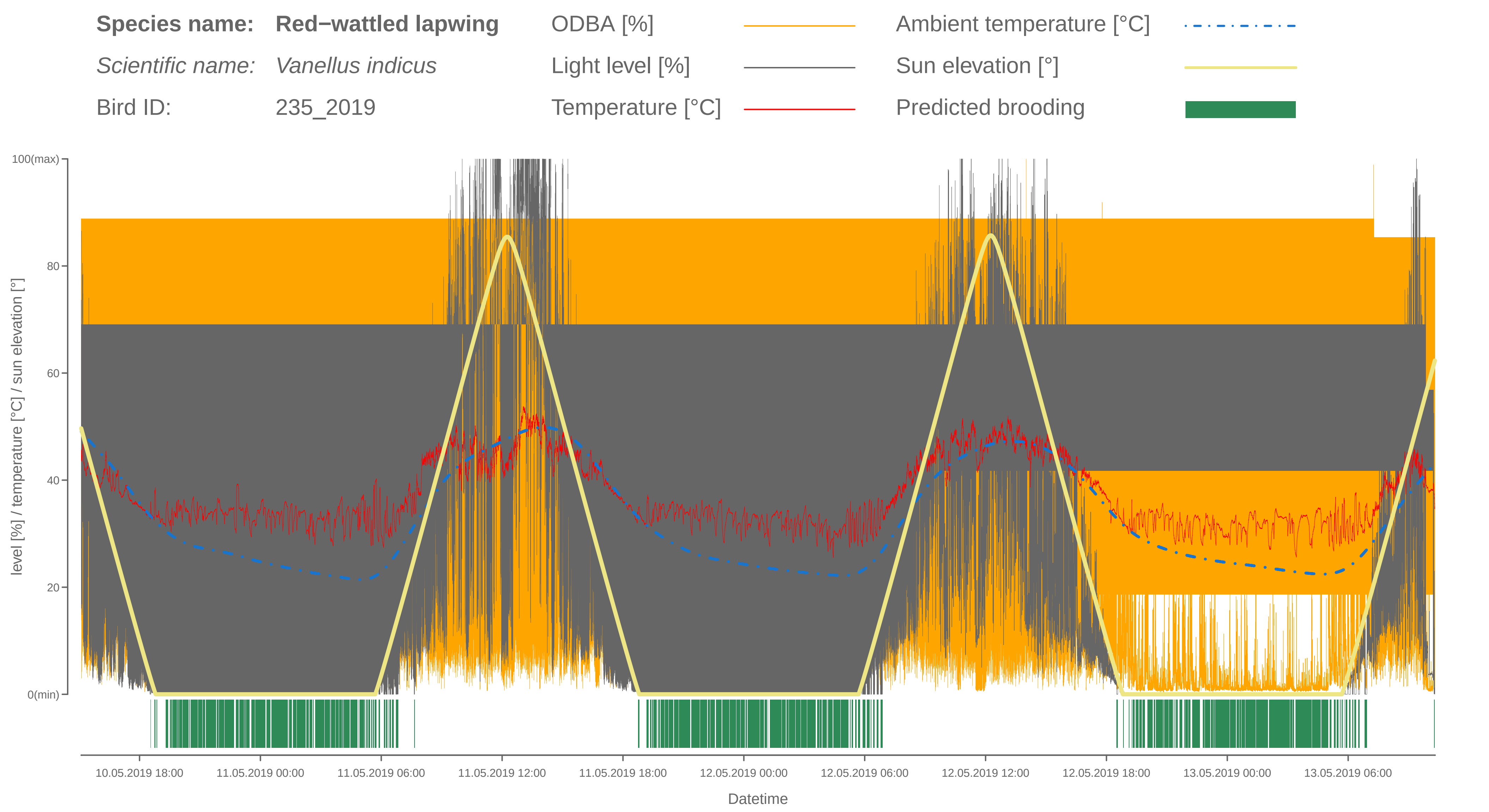
Species name: Red−wattled lapwing
Scientific name: Vanellus indicus
Bird ID: 235_2019
ODBA [%]
Light level [%]
Temperature [°C]
Ambient temperature [°C]
Sun elevation [°]
Predicted brooding
100(max)
80
60
40
20
0(min)
level [%] / temperature [°C] / sun elevation [°]
10.05.2019 18:00
11.05.2019 00:00
11.05.2019 06:00
11.05.2019 12:00
11.05.2019 18:00
12.05.2019 00:00
12.05.2019 06:00
12.05.2019 12:00
12.05.2019 18:00
13.05.2019 00:00
13.05.2019 06:00
Datetime

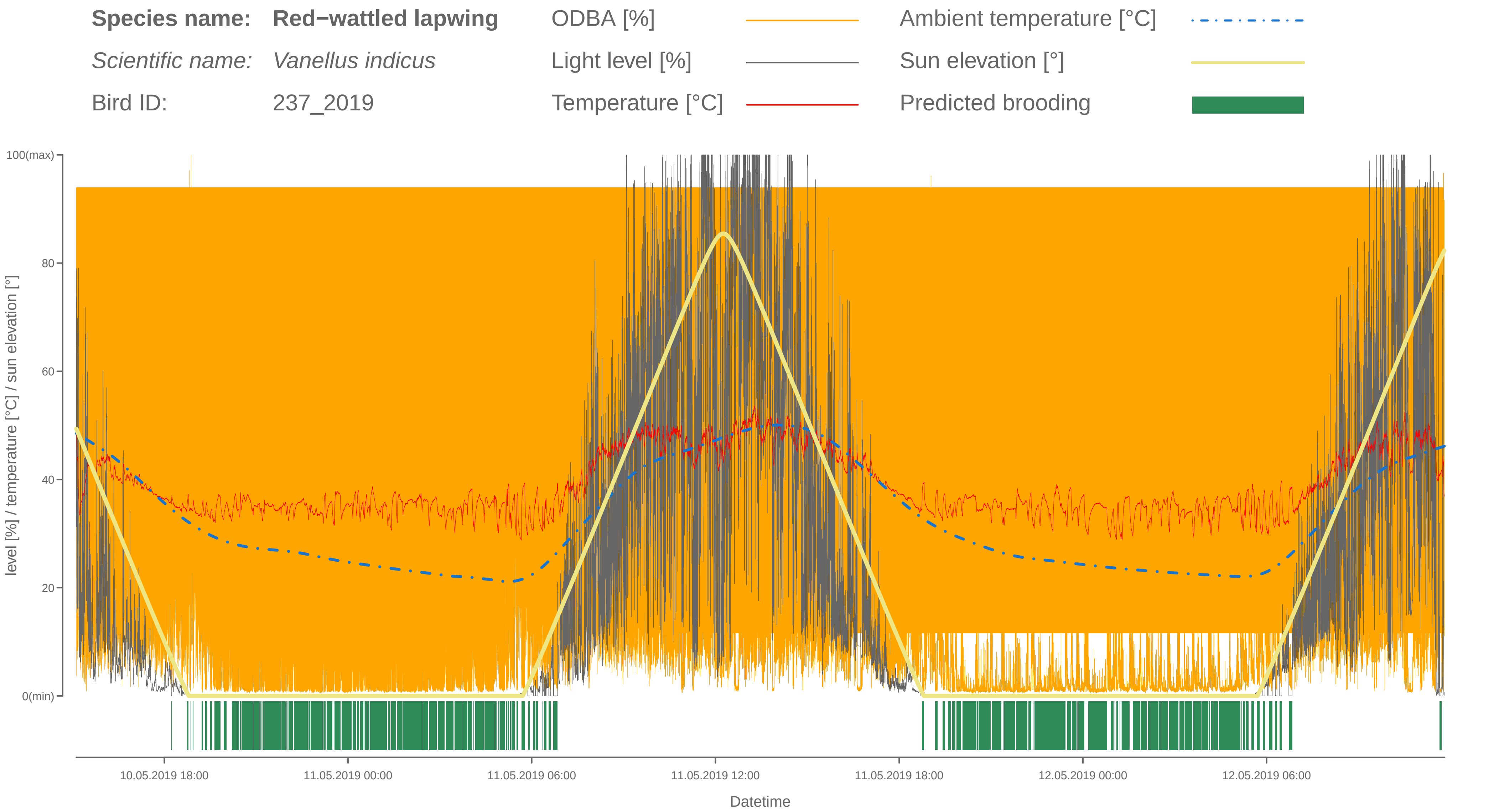

Species name: Red−wattled lapwing
Scientific name: Vanellus indicus
Bird ID: 237_2019
ODBA [%]
Light level [%]
Temperature [°C]
Ambient temperature [°C]
Sun elevation [°]
Predicted brooding
100(max)
80
60
40
20
0(min)
level [%] / temperature [°C] / sun elevation [°]
10.05.2019 18:00
11.05.2019 00:00
11.05.2019 06:00
11.05.2019 12:00
11.05.2019 18:00
12.05.2019 00:00
12.05.2019 06:00
Datetime

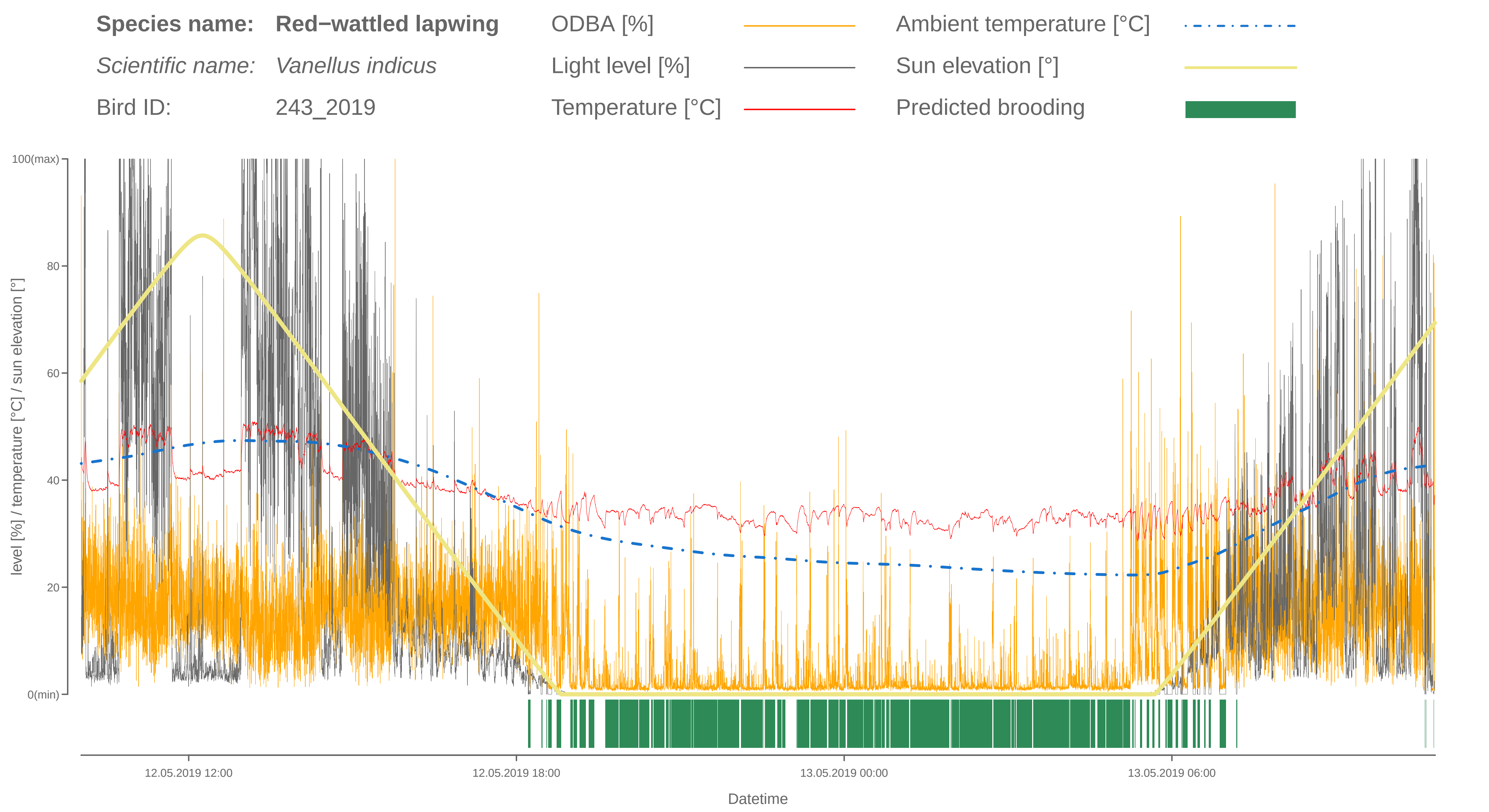
Species name: Red−wattled lapwing
Scientific name: Vanellus indicus
Bird ID: 243_2019
ODBA [%]
Light level [%]
Temperature [°C]
Ambient temperature [°C]
Sun elevation [°]
Predicted brooding
100(max)
80
60
40
20
0(min)
level [%] / temperature [°C] / sun elevation [°]
12.05.2019 12:00
12.05.2019 18:00
13.05.2019 00:00
13.05.2019 06:00
Datetime

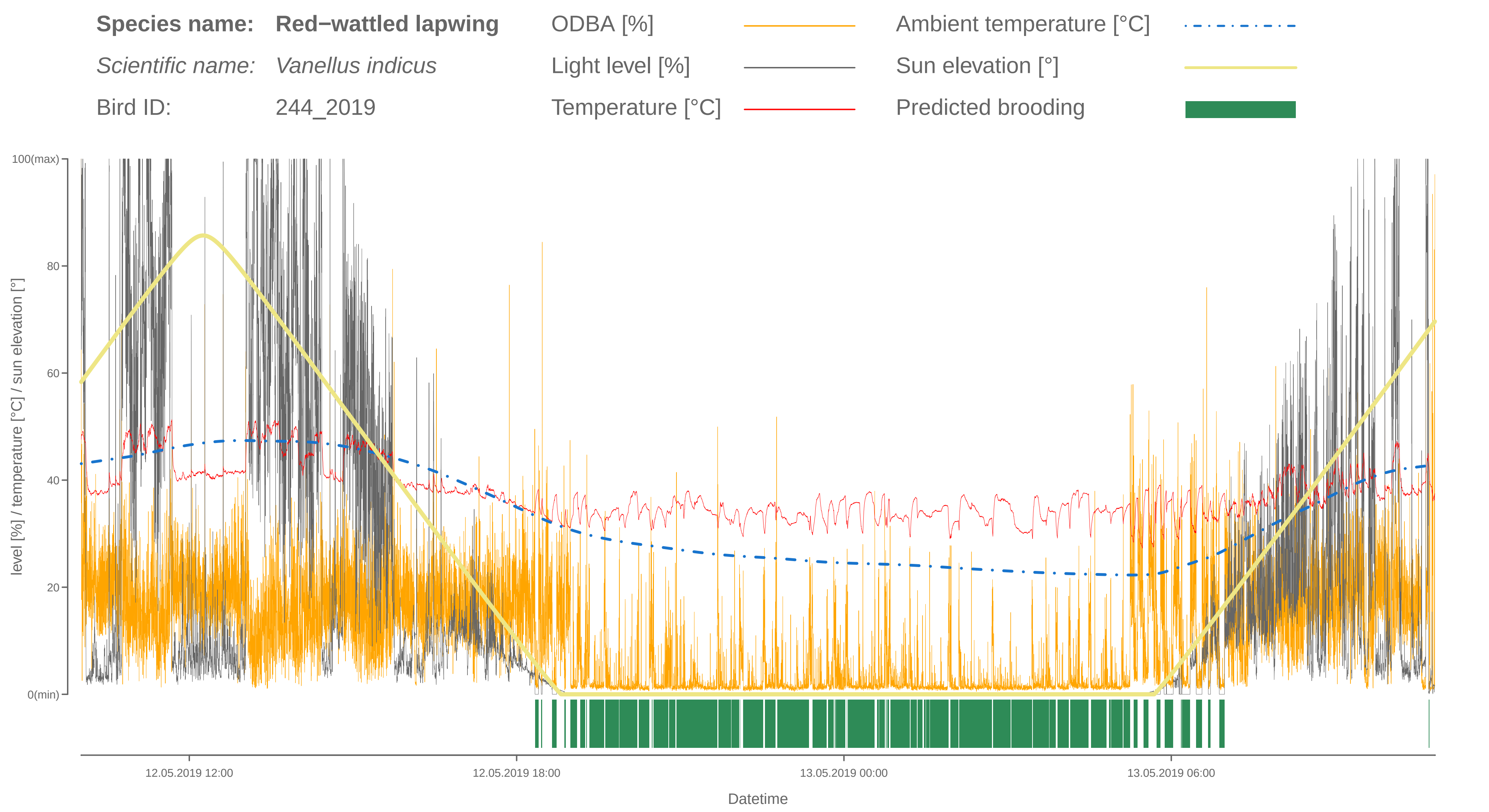

Species name: Red−wattled lapwing
Scientific name: Vanellus indicus
Bird ID: 244_2019
ODBA [%]
Light level [%]
Temperature [°C]
Ambient temperature [°C]
Sun elevation [°]
Predicted brooding
100(max)
80
60
40
20
0(min)
level [%] / temperature [°C] / sun elevation [°]
12.05.2019 12:00
12.05.2019 18:00
13.05.2019 00:00
13.05.2019 06:00
Datetime

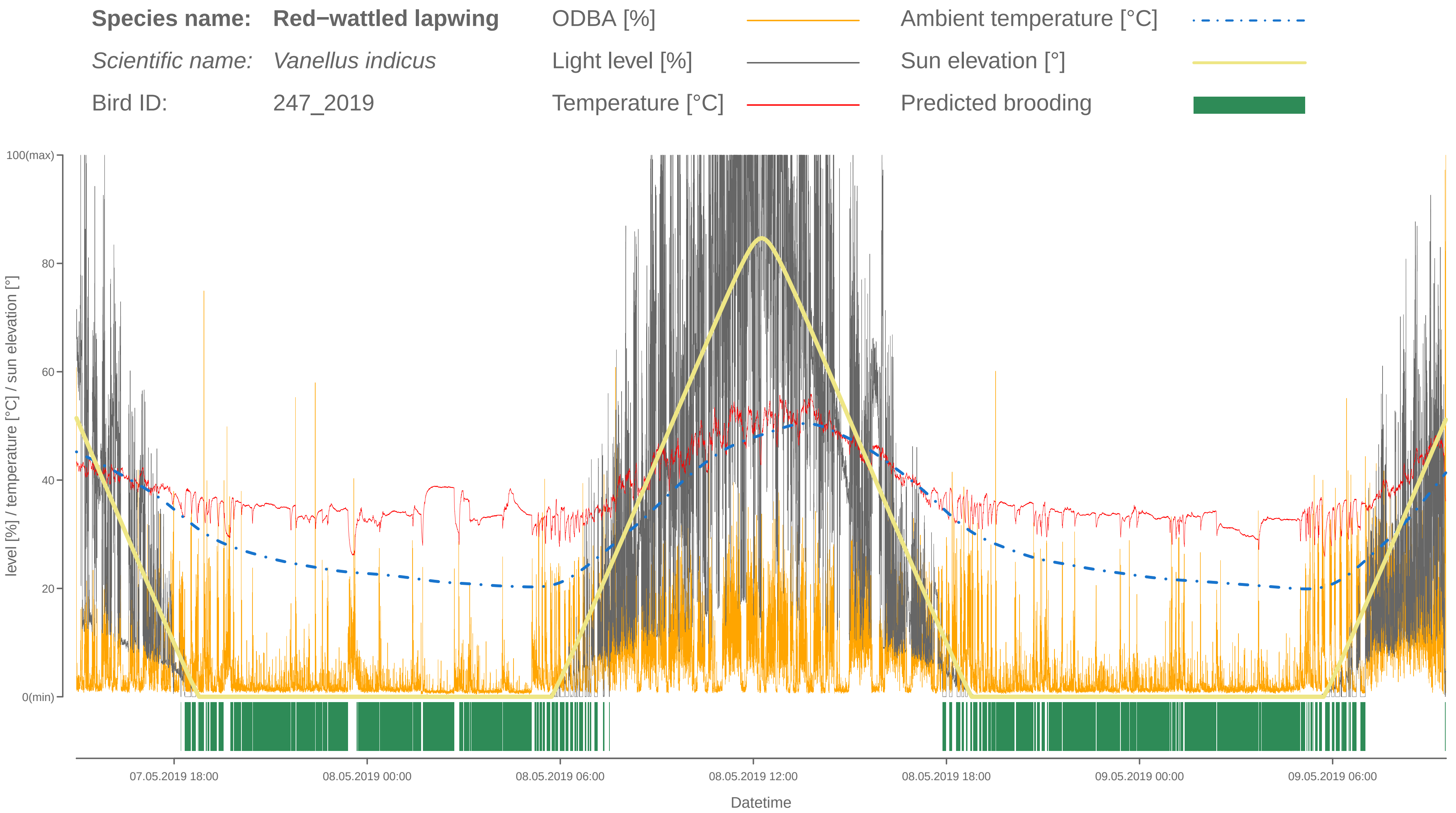

Species name: Red−wattled lapwing
Scientific name: Vanellus indicus
Bird ID: 247_2019
ODBA [%]
Light level [%]
Temperature [°C]
Ambient temperature [°C]
Sun elevation [°]
Predicted brooding
100(max)
80
60
40
20
0(min)
level [%] / temperature [°C] / sun elevation [°]
07.05.2019 18:00
08.05.2019 00:00
08.05.2019 06:00
08.05.2019 12:00
08.05.2019 18:00
09.05.2019 00:00
09.05.2019 06:00
Datetime

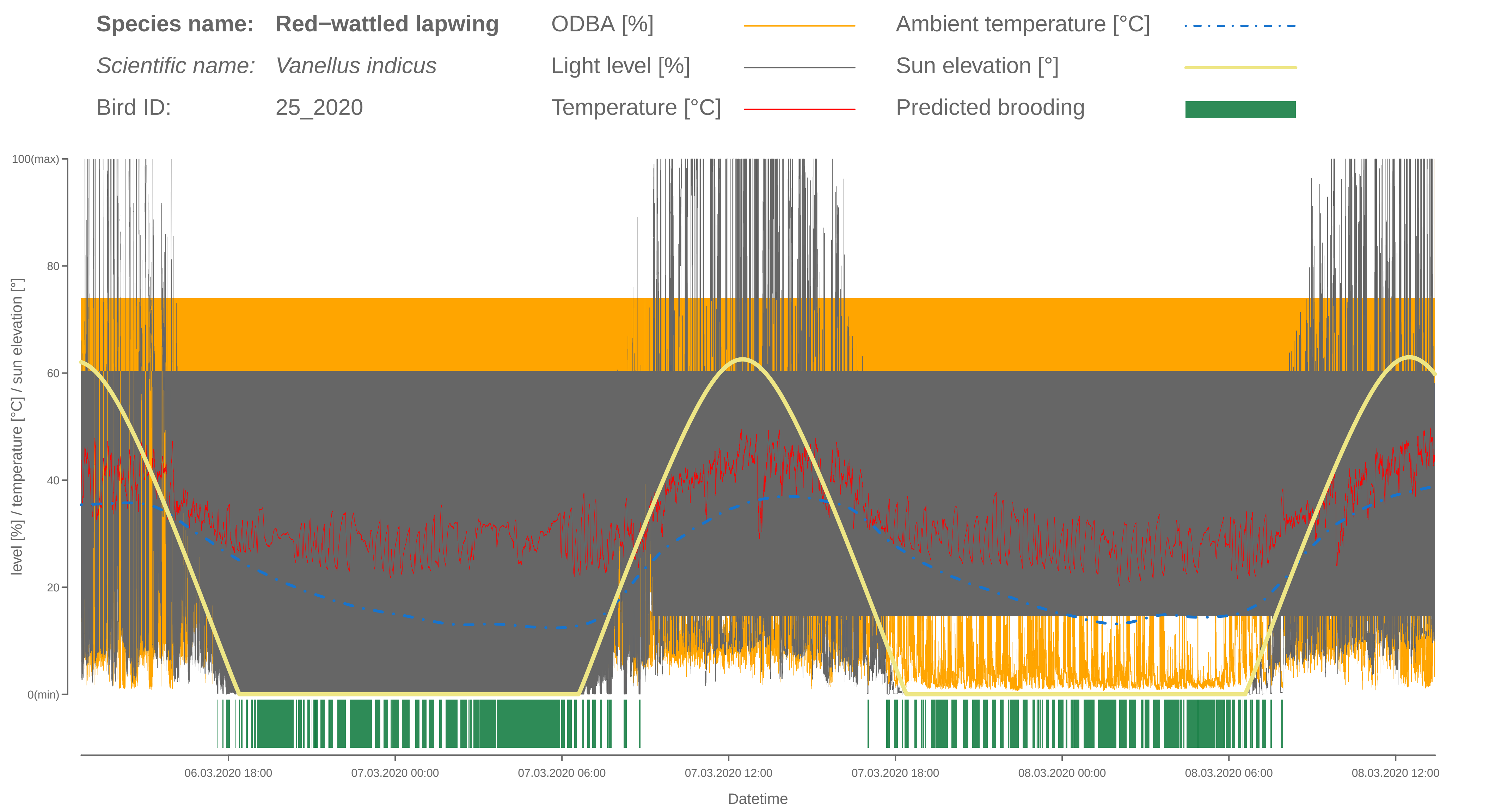

Species name: Red−wattled lapwing
Scientific name: Vanellus indicus
Bird ID: 25_2020
ODBA [%]
Light level [%]
Temperature [°C]
Ambient temperature [°C]
Sun elevation [°]
Predicted brooding
100(max)
80
60
40
20
0(min)
level [%] / temperature [°C] / sun elevation [°]
06.03.2020 18:00
07.03.2020 00:00
07.03.2020 06:00
07.03.2020 12:00
07.03.2020 18:00
08.03.2020 00:00
08.03.2020 06:00
08.03.2020 12:00
Datetime

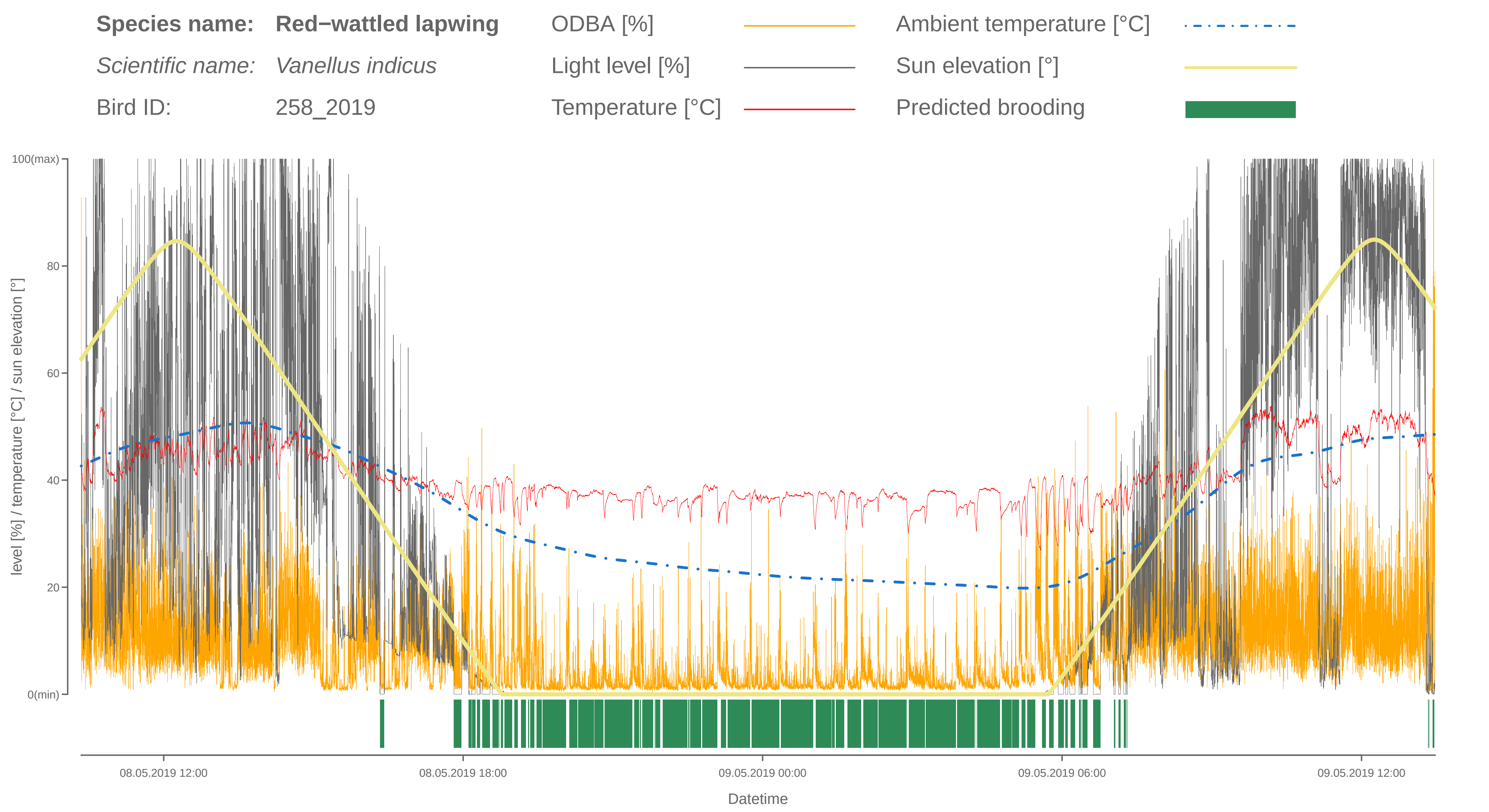
Species name: Red−wattled lapwing
Scientific name: Vanellus indicus
Bird ID: 258_2019
ODBA [%]
Light level [%]
Temperature [°C]
Ambient temperature [°C]
Sun elevation [°]
Predicted brooding
100(max)
80
60
40
20
0(min)
level [%] / temperature [°C] / sun elevation [°]
08.05.2019 12:00
08.05.2019 18:00
09.05.2019 00:00
09.05.2019 06:00
09.05.2019 12:00
Datetime

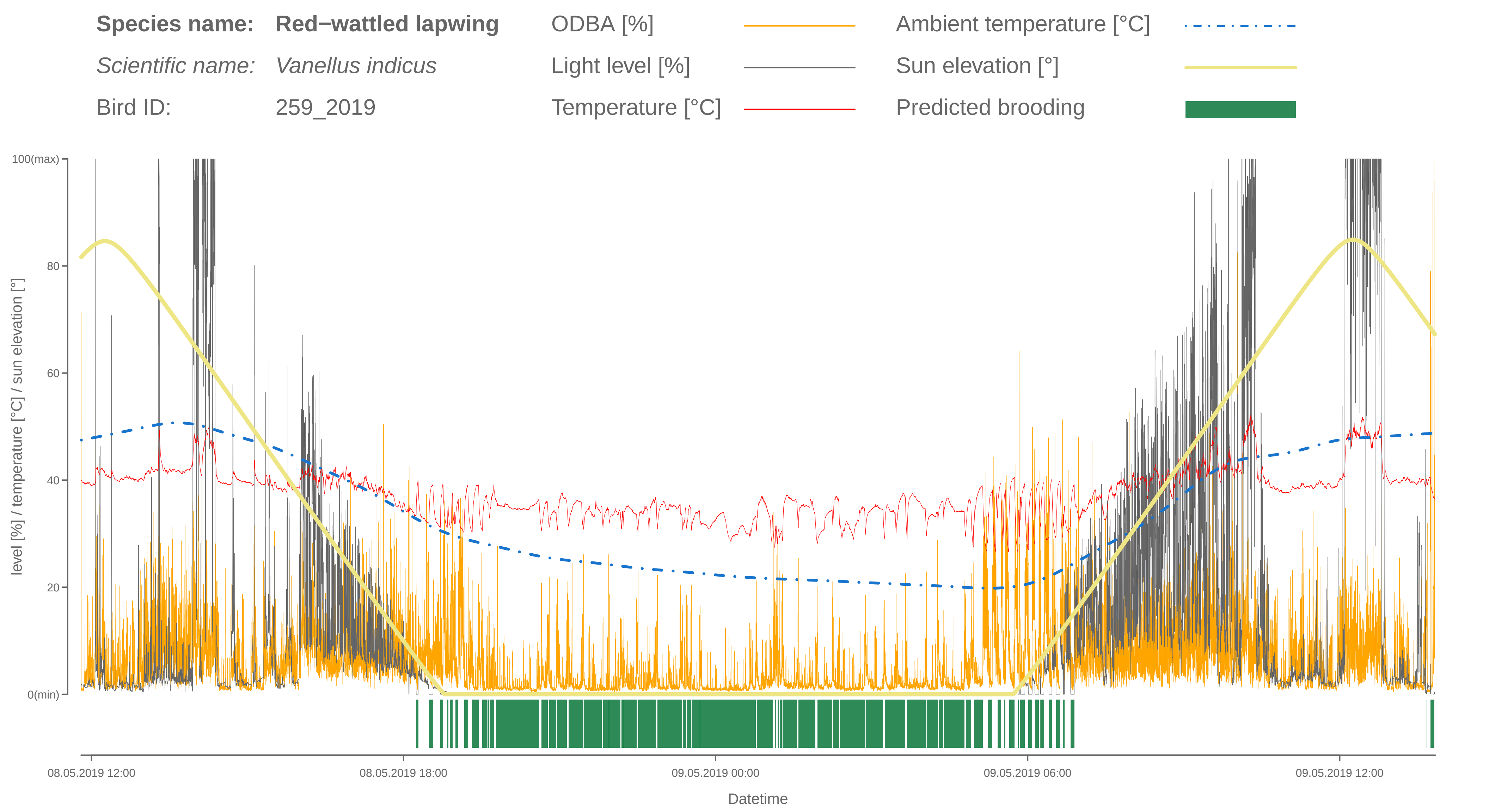
Species name: Red−wattled lapwing
Scientific name: Vanellus indicus
Bird ID: 259_2019
ODBA [%]
Light level [%]
Temperature [°C]
Ambient temperature [°C]
Sun elevation [°]
Predicted brooding
100(max)
80
60
40
20
0(min)
level [%] / temperature [°C] / sun elevation [°]
08.05.2019 12:00
08.05.2019 18:00
09.05.2019 00:00
09.05.2019 06:00
09.05.2019 12:00
Datetime

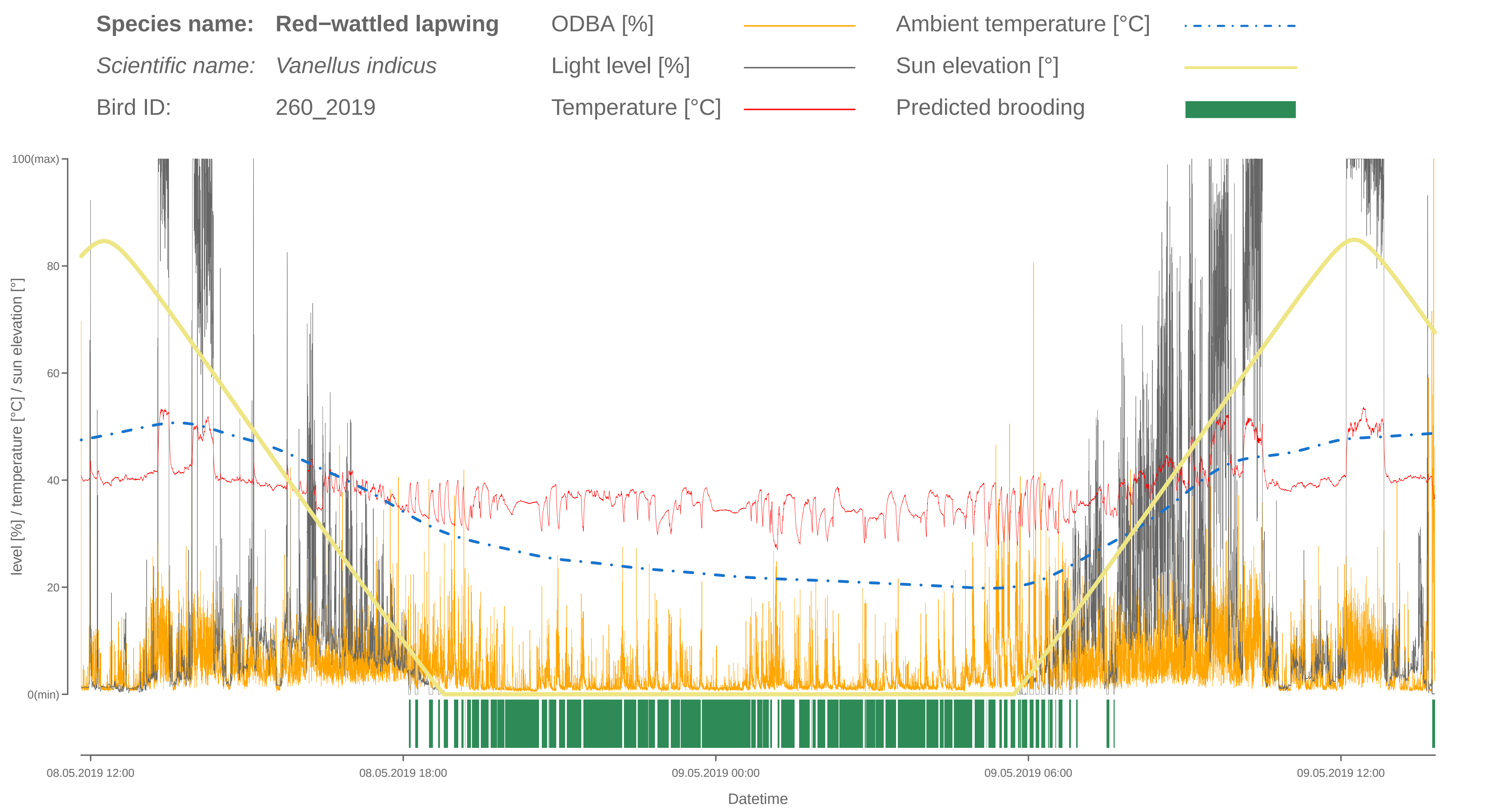
Species name: Red−wattled lapwing
Scientific name: Vanellus indicus
Bird ID: 260_2019
ODBA [%]
Light level [%]
Temperature [°C]
Ambient temperature [°C]
Sun elevation [°]
Predicted brooding
100(max)
80
60
40
20
0(min)
level [%] / temperature [°C] / sun elevation [°]
08.05.2019 12:00
08.05.2019 18:00
09.05.2019 00:00
09.05.2019 06:00
09.05.2019 12:00
Datetime

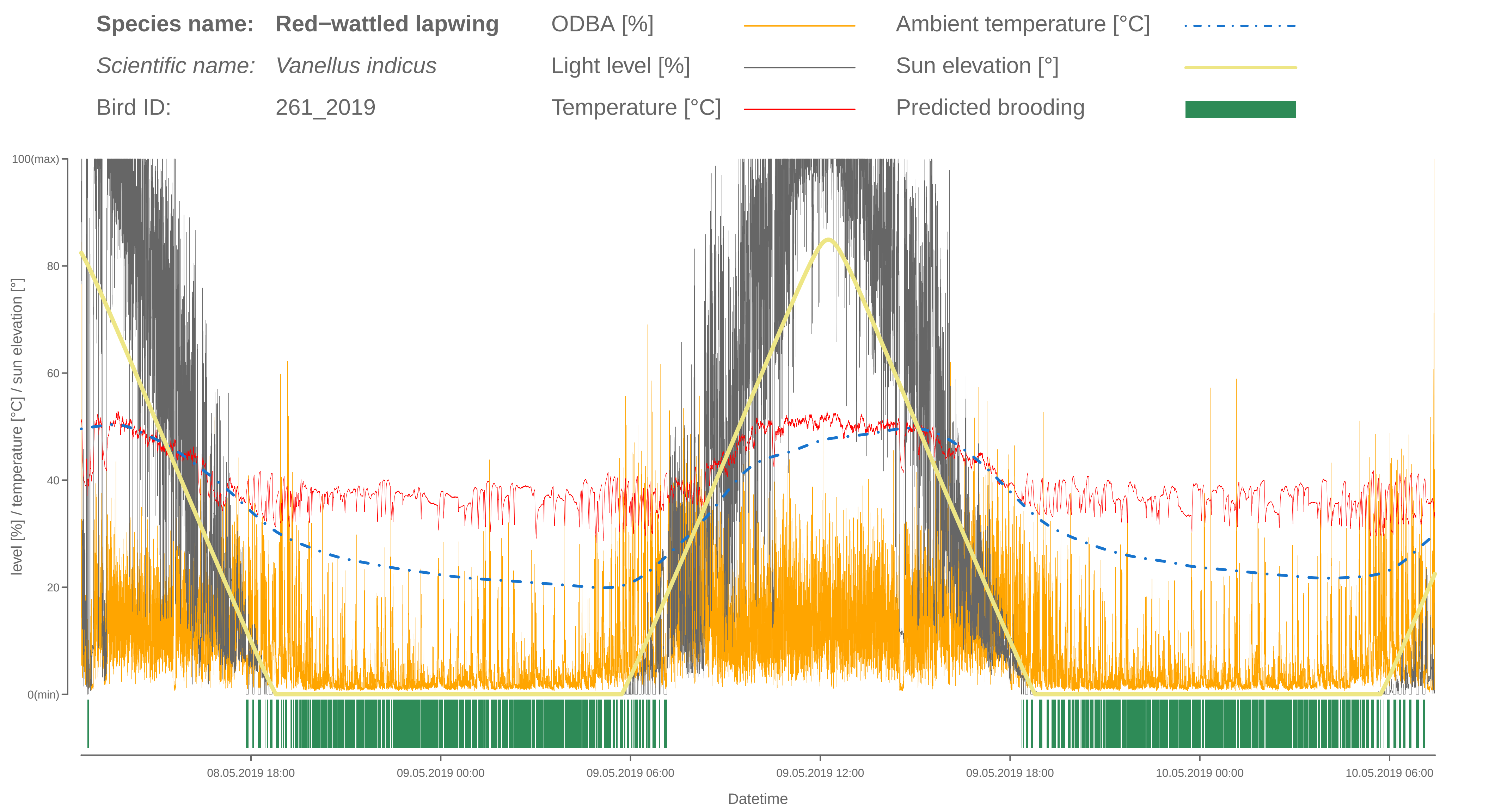
Species name: Red−wattled lapwing
Scientific name: Vanellus indicus
Bird ID: 261_2019
ODBA [%]
Light level [%]
Temperature [°C]
Ambient temperature [°C]
Sun elevation [°]
Predicted brooding
100(max)
80
60
40
20
0(min)
level [%] / temperature [°C] / sun elevation [°]
08.05.2019 18:00
09.05.2019 00:00
09.05.2019 06:00
09.05.2019 12:00
09.05.2019 18:00
10.05.2019 00:00
10.05.2019 06:00
Datetime

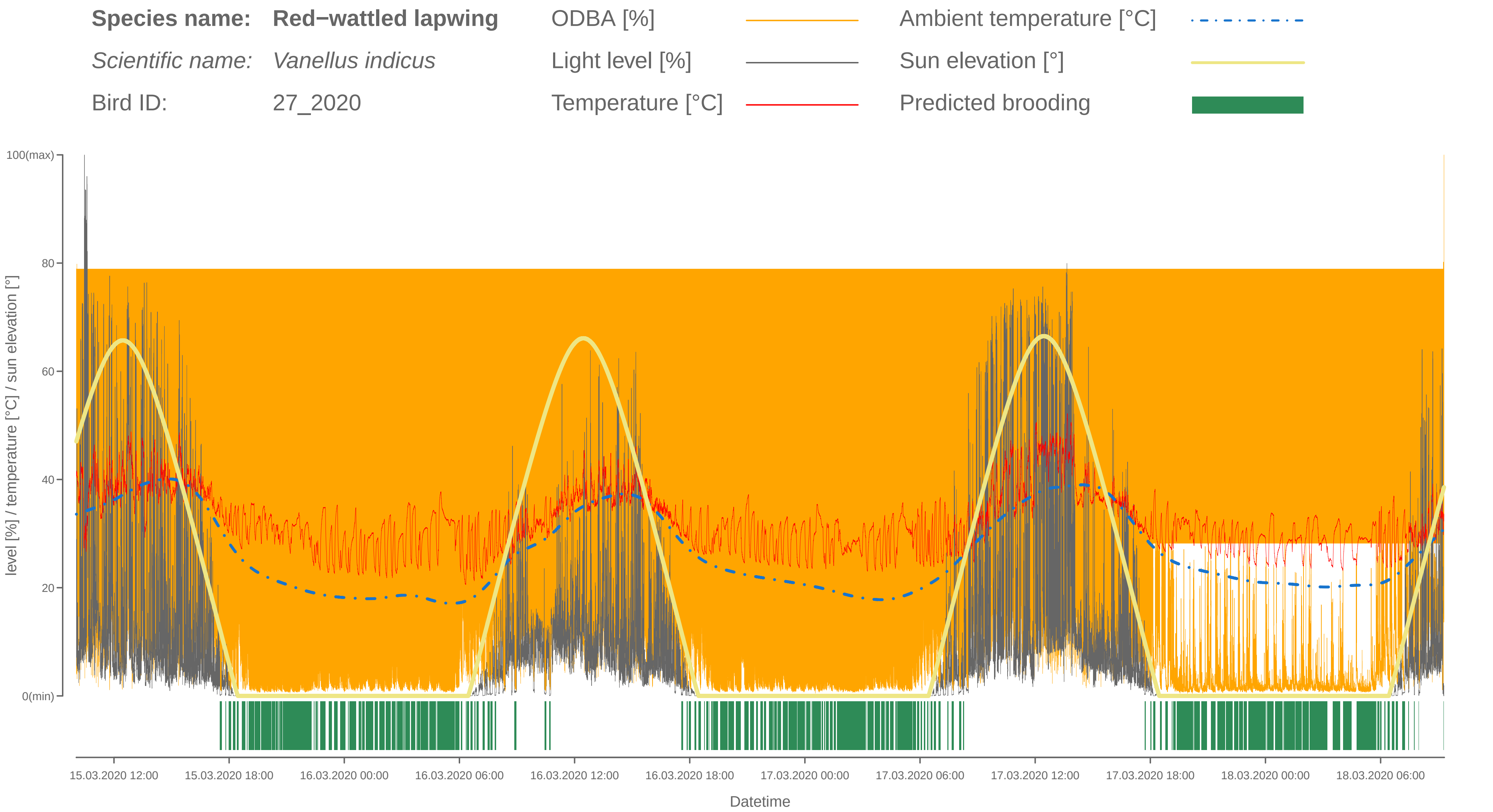
Species name: Red−wattled lapwing
Scientific name: Vanellus indicus
Bird ID: 27_2020
ODBA [%]
Light level [%]
Temperature [°C]
Ambient temperature [°C]
Sun elevation [°]
Predicted brooding
100(max)
80
60
40
20
0(min)
level [%] / temperature [°C] / sun elevation [°]
15.03.2020 12:00
15.03.2020 18:00
16.03.2020 00:00
16.03.2020 06:00
16.03.2020 12:00
16.03.2020 18:00
17.03.2020 00:00
17.03.2020 06:00
17.03.2020 12:00
17.03.2020 18:00
18.03.2020 00:00
18.03.2020 06:00
Datetime

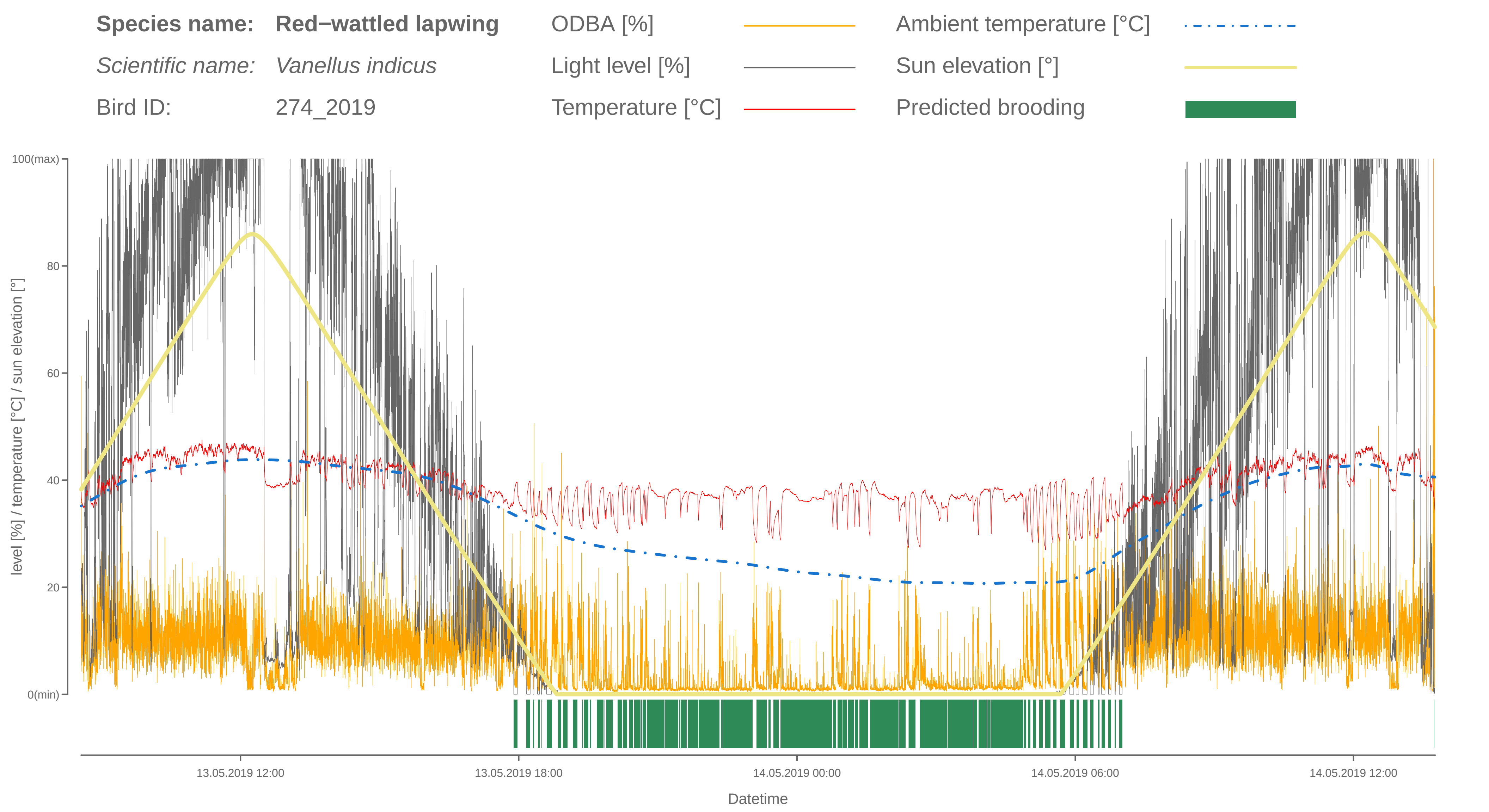
Species name: Red−wattled lapwing
Scientific name: Vanellus indicus
Bird ID: 274_2019
ODBA [%]
Light level [%]
Temperature [°C]
Ambient temperature [°C]
Sun elevation [°]
Predicted brooding
100(max)
80
60
40
20
0(min)
level [%] / temperature [°C] / sun elevation [°]
13.05.2019 12:00
13.05.2019 18:00
14.05.2019 00:00
14.05.2019 06:00
14.05.2019 12:00
Datetime

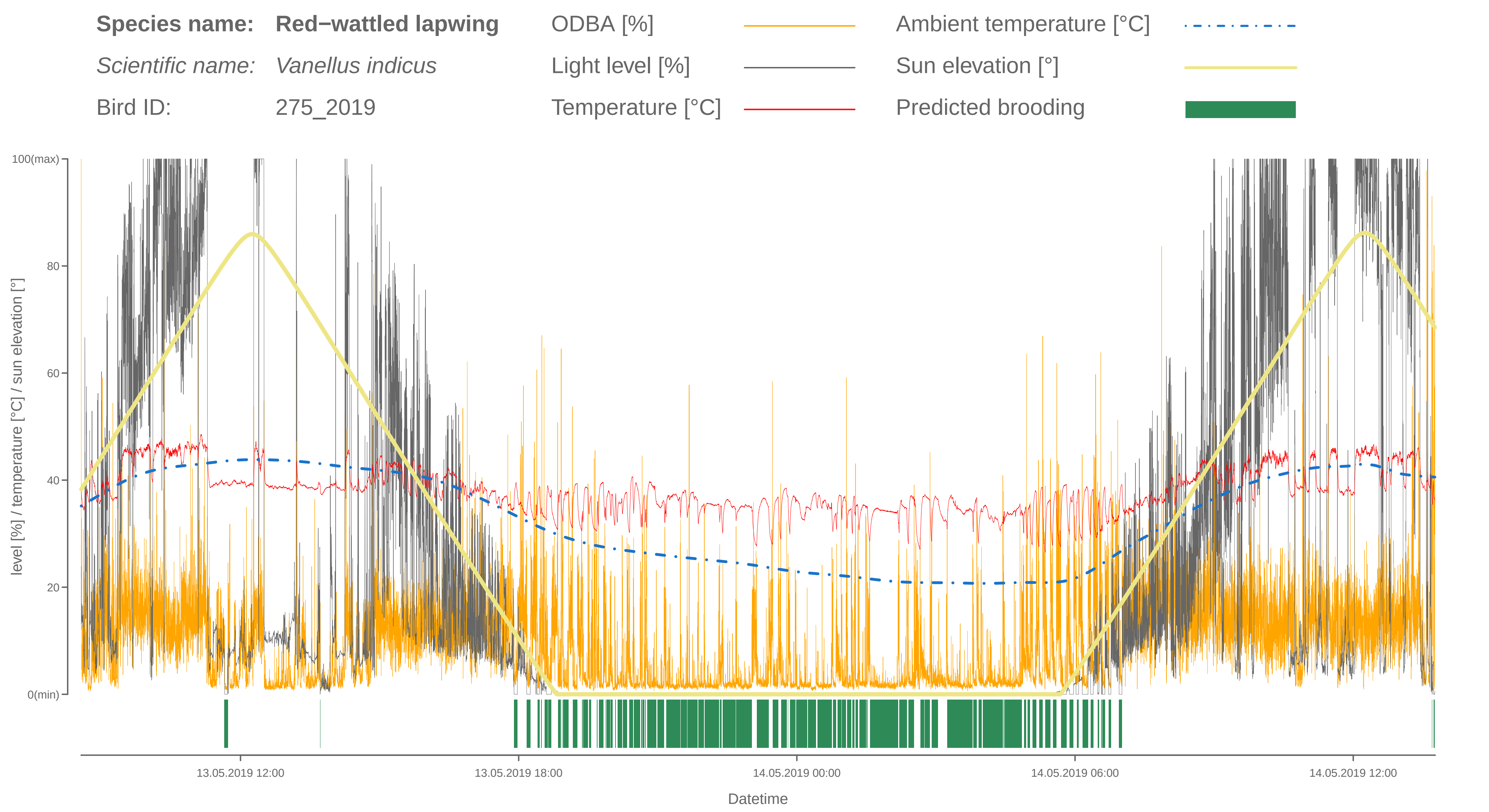

Species name: Red−wattled lapwing
Scientific name: Vanellus indicus
Bird ID: 275_2019
ODBA [%]
Light level [%]
Temperature [°C]
Ambient temperature [°C]
Sun elevation [°]
Predicted brooding
100(max)
80
60
40
20
0(min)
level [%] / temperature [°C] / sun elevation [°]
13.05.2019 12:00
13.05.2019 18:00
14.05.2019 00:00
14.05.2019 06:00
14.05.2019 12:00
Datetime

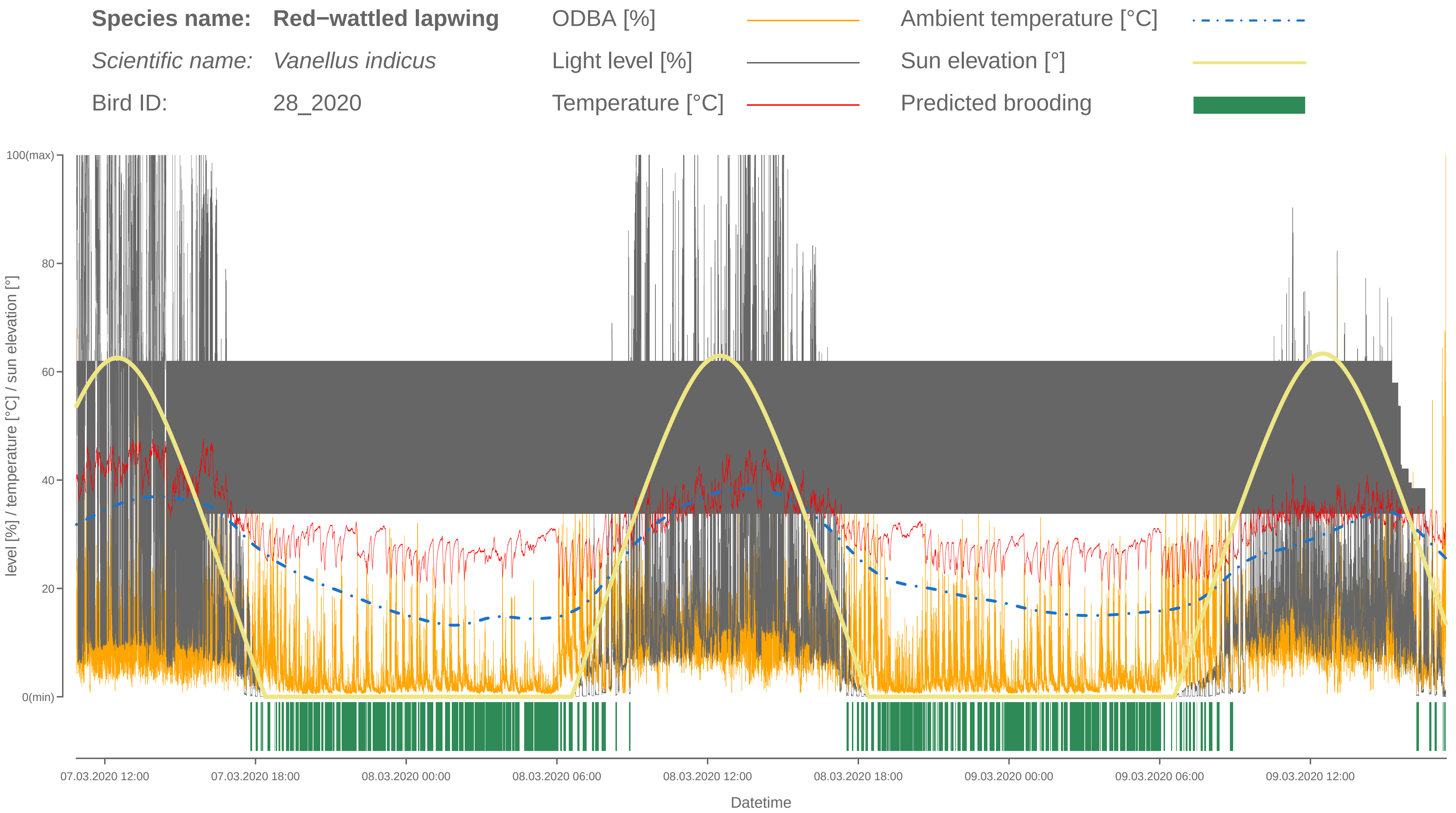
Species name: Red−wattled lapwing
Scientific name: Vanellus indicus
Bird ID: 28_2020
ODBA [%]
Light level [%]
Temperature [°C]
Ambient temperature [°C]
Sun elevation [°]
Predicted brooding
100(max)
80
60
40
20
0(min)
level [%] / temperature [°C] / sun elevation [°]
07.03.2020 12:00
07.03.2020 18:00
08.03.2020 00:00
08.03.2020 06:00
08.03.2020 12:00
08.03.2020 18:00
09.03.2020 00:00
09.03.2020 06:00
09.03.2020 12:00
Datetime

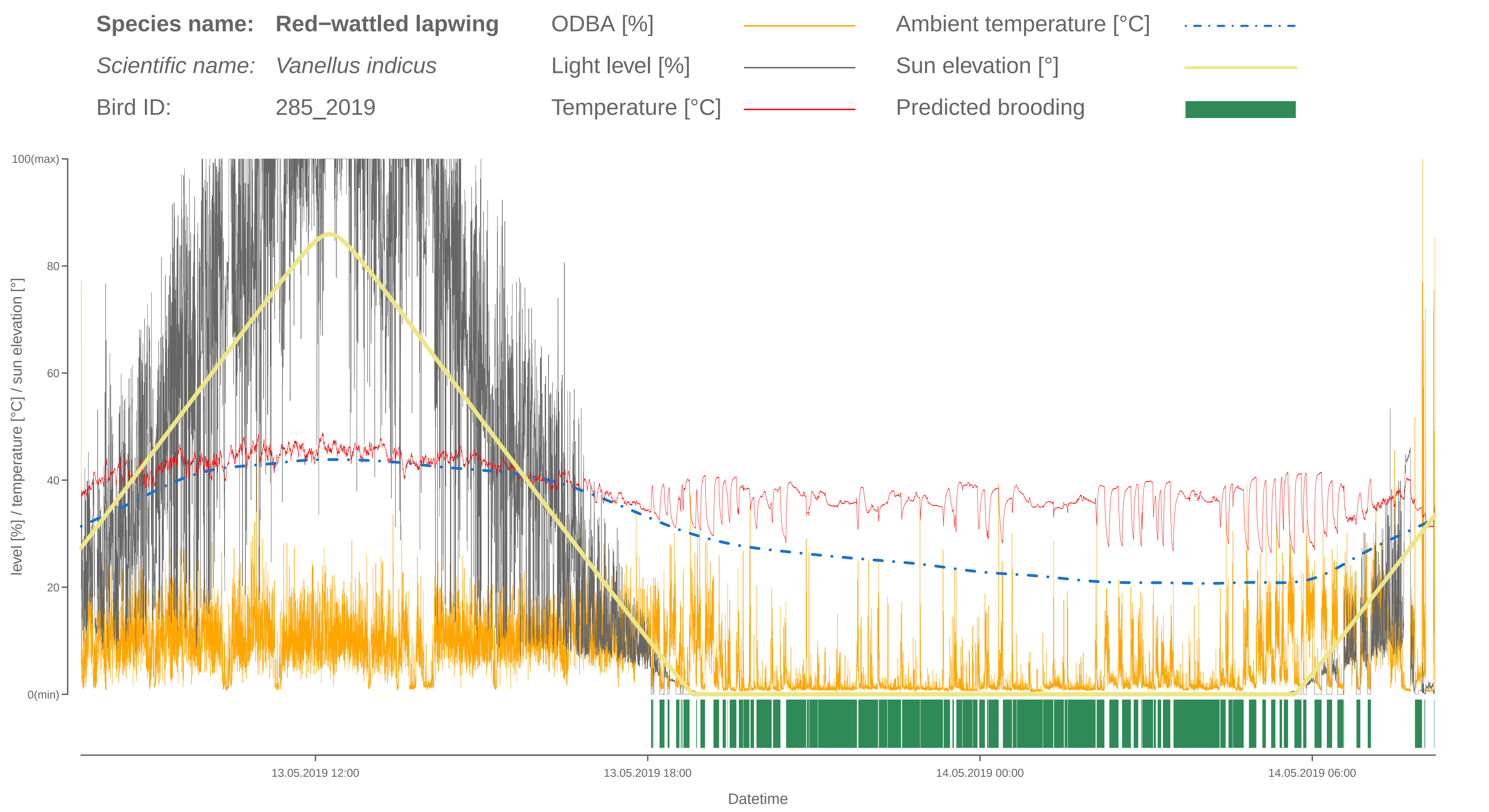

Species name: Red−wattled lapwing
Scientific name: Vanellus indicus
Bird ID: 285_2019
ODBA [%]
Light level [%]
Temperature [°C]
Ambient temperature [°C]
Sun elevation [°]
Predicted brooding
100(max)
80
60
40
20
0(min)
level [%] / temperature [°C] / sun elevation [°]
13.05.2019 12:00
13.05.2019 18:00
14.05.2019 00:00
14.05.2019 06:00
Datetime

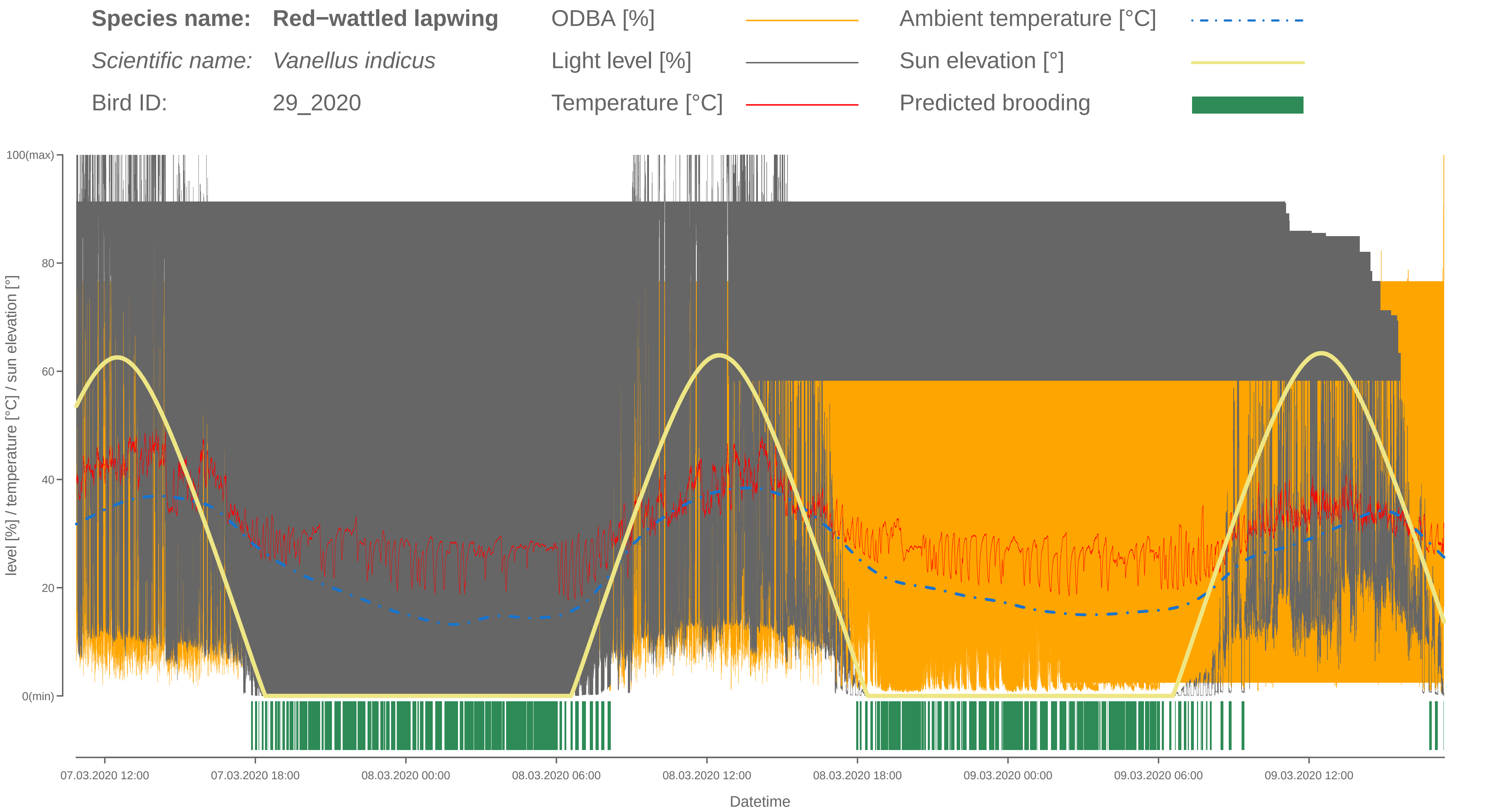

Species name: Red−wattled lapwing
Scientific name: Vanellus indicus
Bird ID: 29_2020
ODBA [%]
Light level [%]
Temperature [°C]
Ambient temperature [°C]
Sun elevation [°]
Predicted brooding
100(max)
80
60
40
20
0(min)
level [%] / temperature [°C] / sun elevation [°]
07.03.2020 12:00
07.03.2020 18:00
08.03.2020 00:00
08.03.2020 06:00
08.03.2020 12:00
08.03.2020 18:00
09.03.2020 00:00
09.03.2020 06:00
09.03.2020 12:00
Datetime

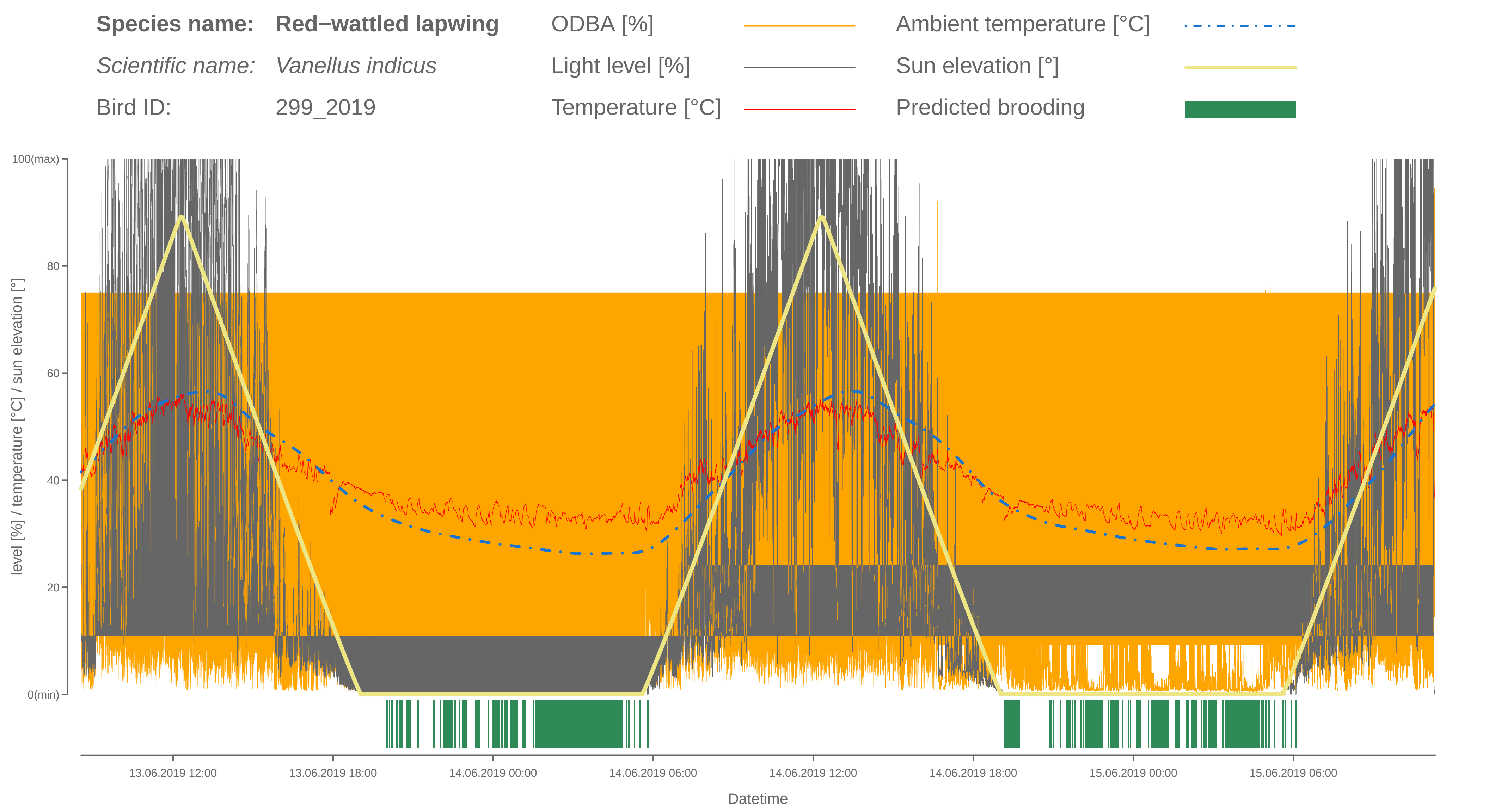
Species name: Red−wattled lapwing
Scientific name: Vanellus indicus
Bird ID: 299_2019
ODBA [%]
Light level [%]
Temperature [°C]
Ambient temperature [°C]
Sun elevation [°]
Predicted brooding
100(max)
80
60
40
20
0(min)
level [%] / temperature [°C] / sun elevation [°]
13.06.2019 12:00
13.06.2019 18:00
14.06.2019 00:00
14.06.2019 06:00
14.06.2019 12:00
14.06.2019 18:00
15.06.2019 00:00
15.06.2019 06:00
Datetime

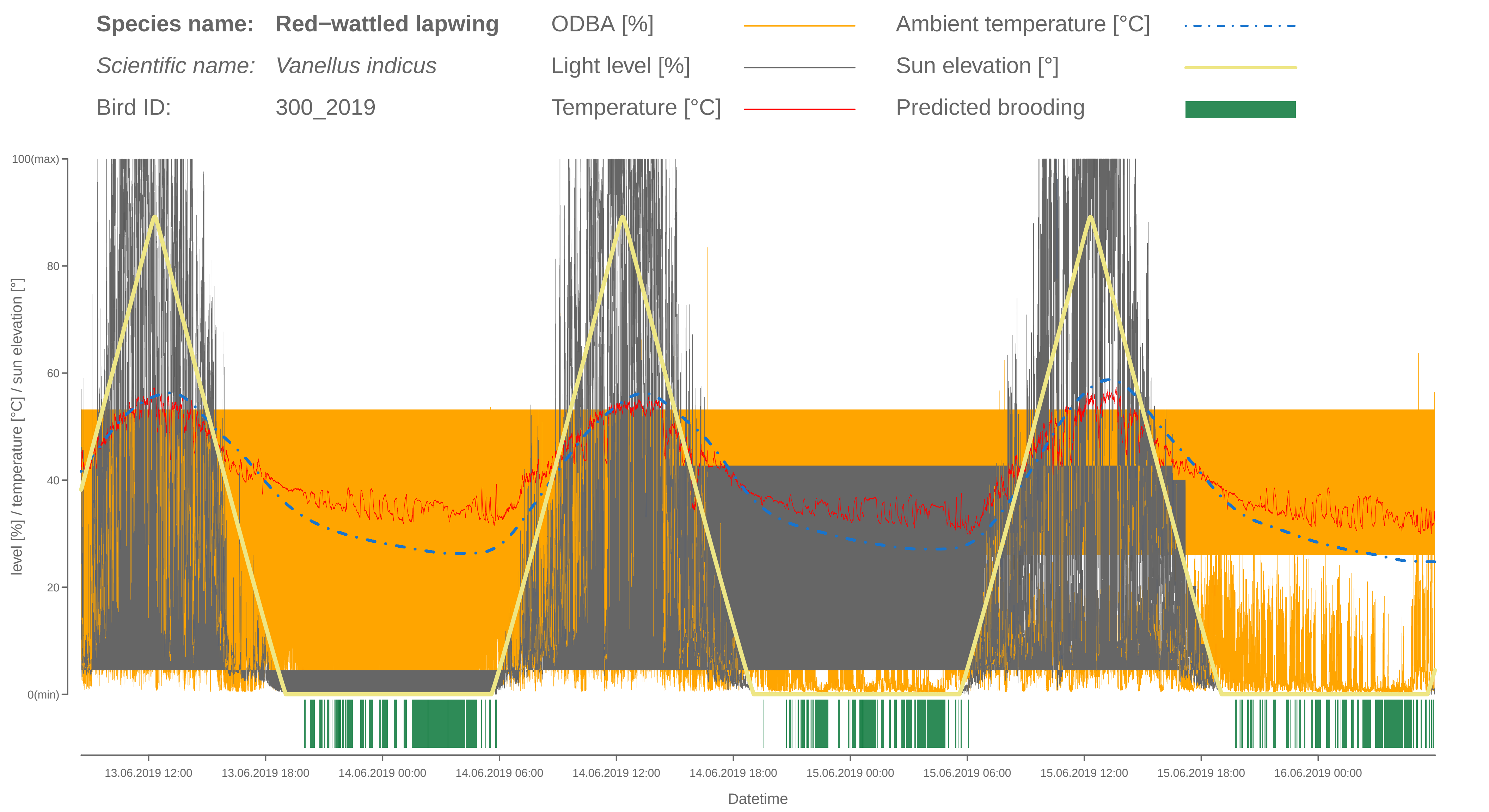

Species name: Red−wattled lapwing
Scientific name: Vanellus indicus
Bird ID: 300_2019
ODBA [%]
Light level [%]
Temperature [°C]
Ambient temperature [°C]
Sun elevation [°]
Predicted brooding
100(max)
80
60
40
20
0(min)
level [%] / temperature [°C] / sun elevation [°]
13.06.2019 12:00
13.06.2019 18:00
14.06.2019 00:00
14.06.2019 06:00
14.06.2019 12:00
14.06.2019 18:00
15.06.2019 00:00
15.06.2019 06:00
15.06.2019 12:00
15.06.2019 18:00
16.06.2019 00:00
Datetime

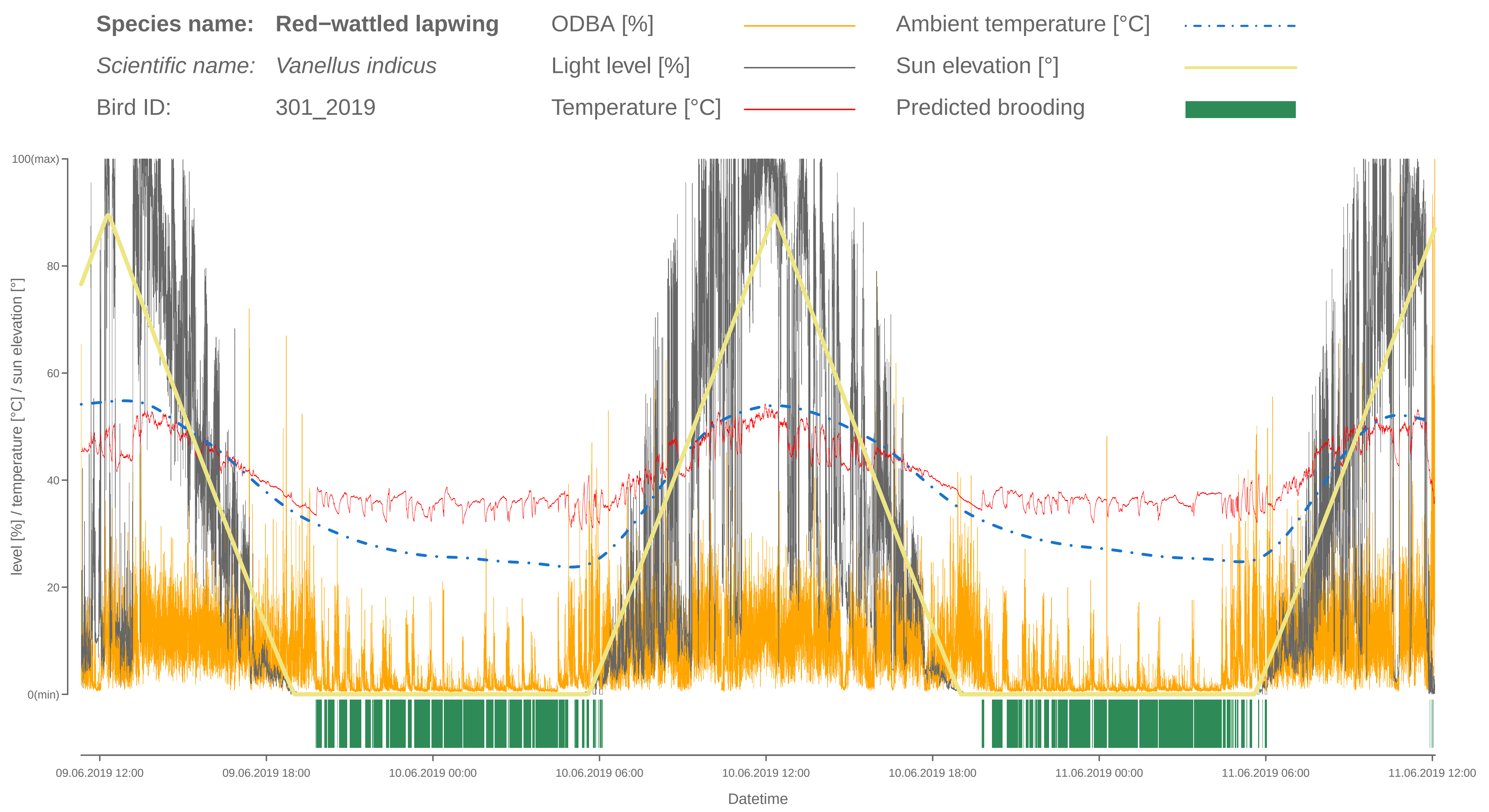

Species name: Red−wattled lapwing
Scientific name: Vanellus indicus
Bird ID: 301_2019
ODBA [%]
Light level [%]
Temperature [°C]
Ambient temperature [°C]
Sun elevation [°]
Predicted brooding
100(max)
80
60
40
20
0(min)
level [%] / temperature [°C] / sun elevation [°]
09.06.2019 12:00
09.06.2019 18:00
10.06.2019 00:00
10.06.2019 06:00
10.06.2019 12:00
10.06.2019 18:00
11.06.2019 00:00
11.06.2019 06:00
11.06.2019 12:00
Datetime

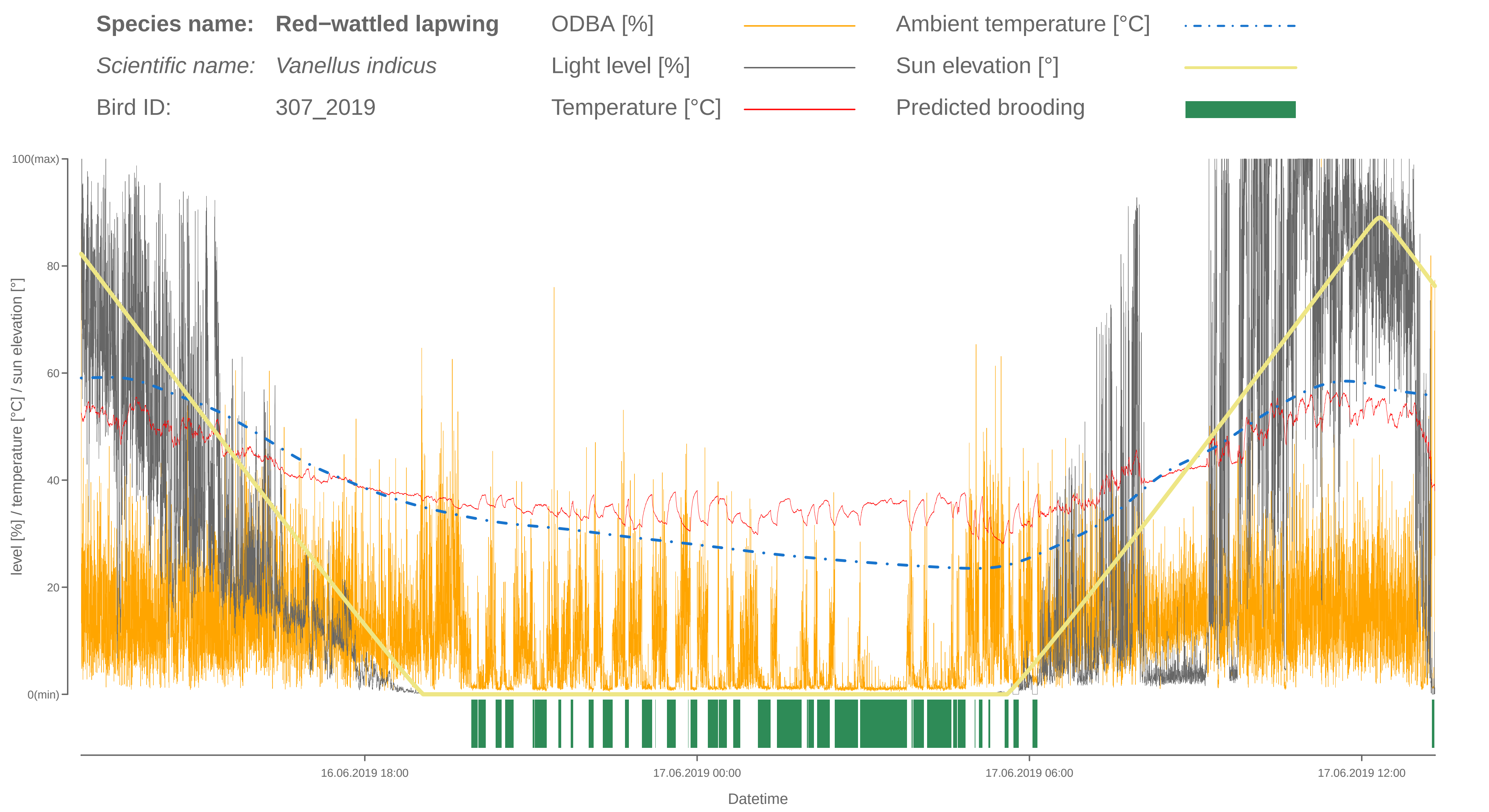

Species name: Red−wattled lapwing
Scientific name: Vanellus indicus
Bird ID: 307_2019
ODBA [%]
Light level [%]
Temperature [°C]
Ambient temperature [°C]
Sun elevation [°]
Predicted brooding
100(max)
80
60
40
20
0(min)
level [%] / temperature [°C] / sun elevation [°]
16.06.2019 18:00
17.06.2019 00:00
17.06.2019 06:00
17.06.2019 12:00
Datetime

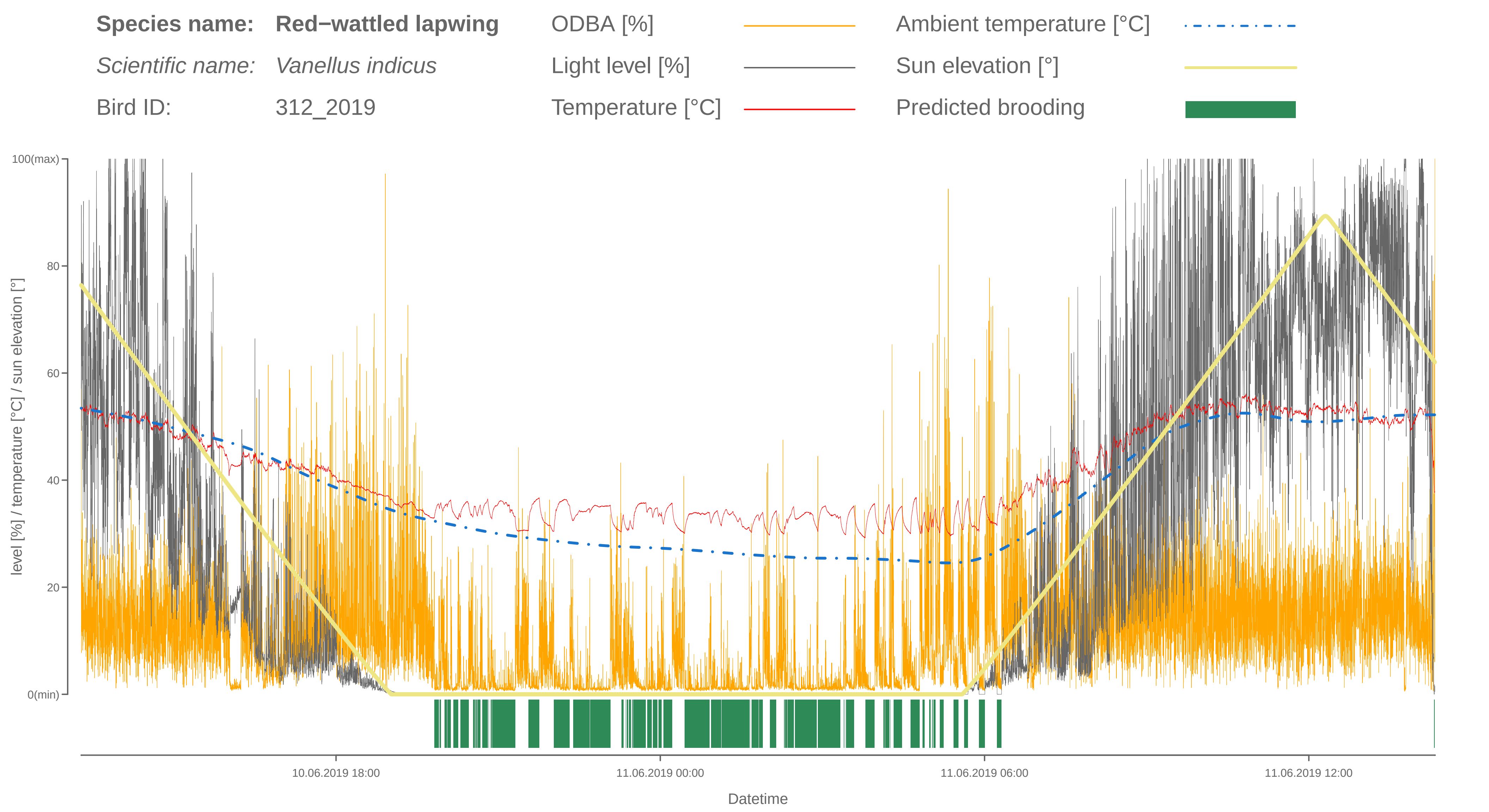
Species name: Red−wattled lapwing
Scientific name: Vanellus indicus
Bird ID: 312_2019
ODBA [%]
Light level [%]
Temperature [°C]
Ambient temperature [°C]
Sun elevation [°]
Predicted brooding
100(max)
80
60
40
20
0(min)
level [%] / temperature [°C] / sun elevation [°]
10.06.2019 18:00
11.06.2019 00:00
11.06.2019 06:00
11.06.2019 12:00
Datetime

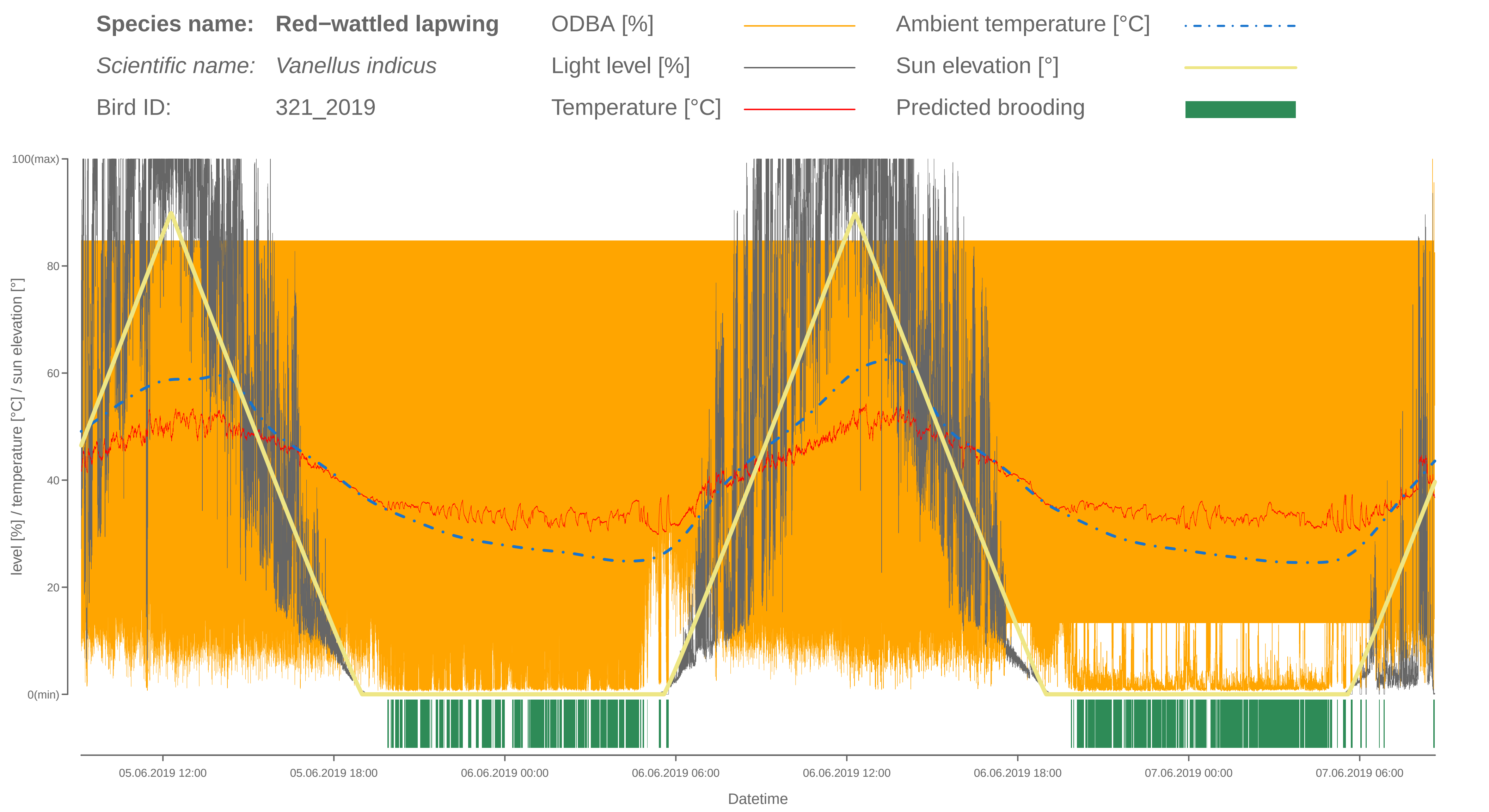

Species name: Red−wattled lapwing
Scientific name: Vanellus indicus
Bird ID: 321_2019
ODBA [%]
Light level [%]
Temperature [°C]
Ambient temperature [°C]
Sun elevation [°]
Predicted brooding
100(max)
80
60
40
20
0(min)
level [%] / temperature [°C] / sun elevation [°]
05.06.2019 12:00
05.06.2019 18:00
06.06.2019 00:00
06.06.2019 06:00
06.06.2019 12:00
06.06.2019 18:00
07.06.2019 00:00
07.06.2019 06:00
Datetime

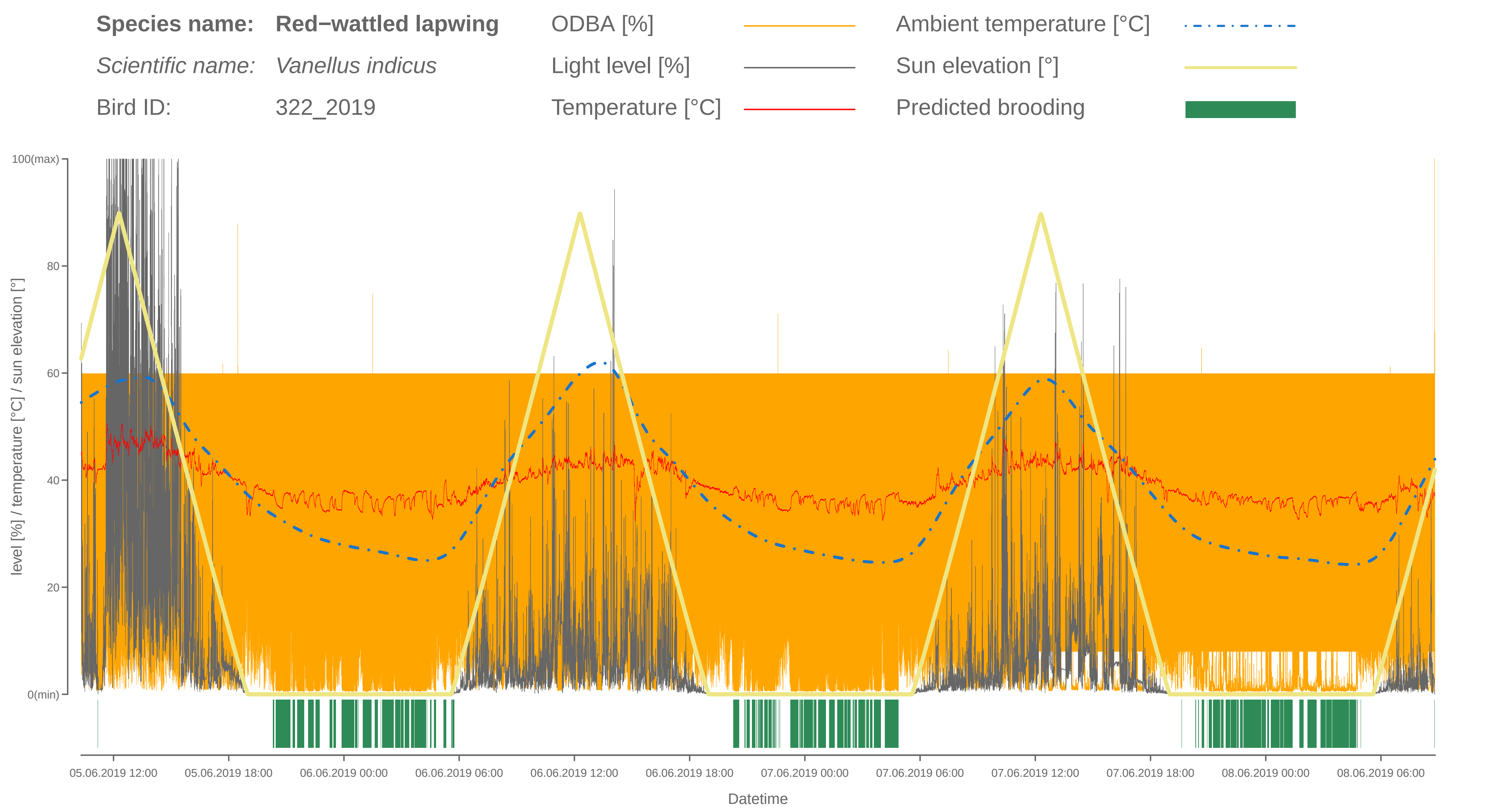

Species name: Red−wattled lapwing
Scientific name: Vanellus indicus
Bird ID: 322_2019
ODBA [%]
Light level [%]
Temperature [°C]
Ambient temperature [°C]
Sun elevation [°]
Predicted brooding
100(max)
80
60
40
20
0(min)
level [%] / temperature [°C] / sun elevation [°]
05.06.2019 12:00
05.06.2019 18:00
06.06.2019 00:00
06.06.2019 06:00
06.06.2019 12:00
06.06.2019 18:00
07.06.2019 00:00
07.06.2019 06:00
07.06.2019 12:00
07.06.2019 18:00
08.06.2019 00:00
08.06.2019 06:00
Datetime

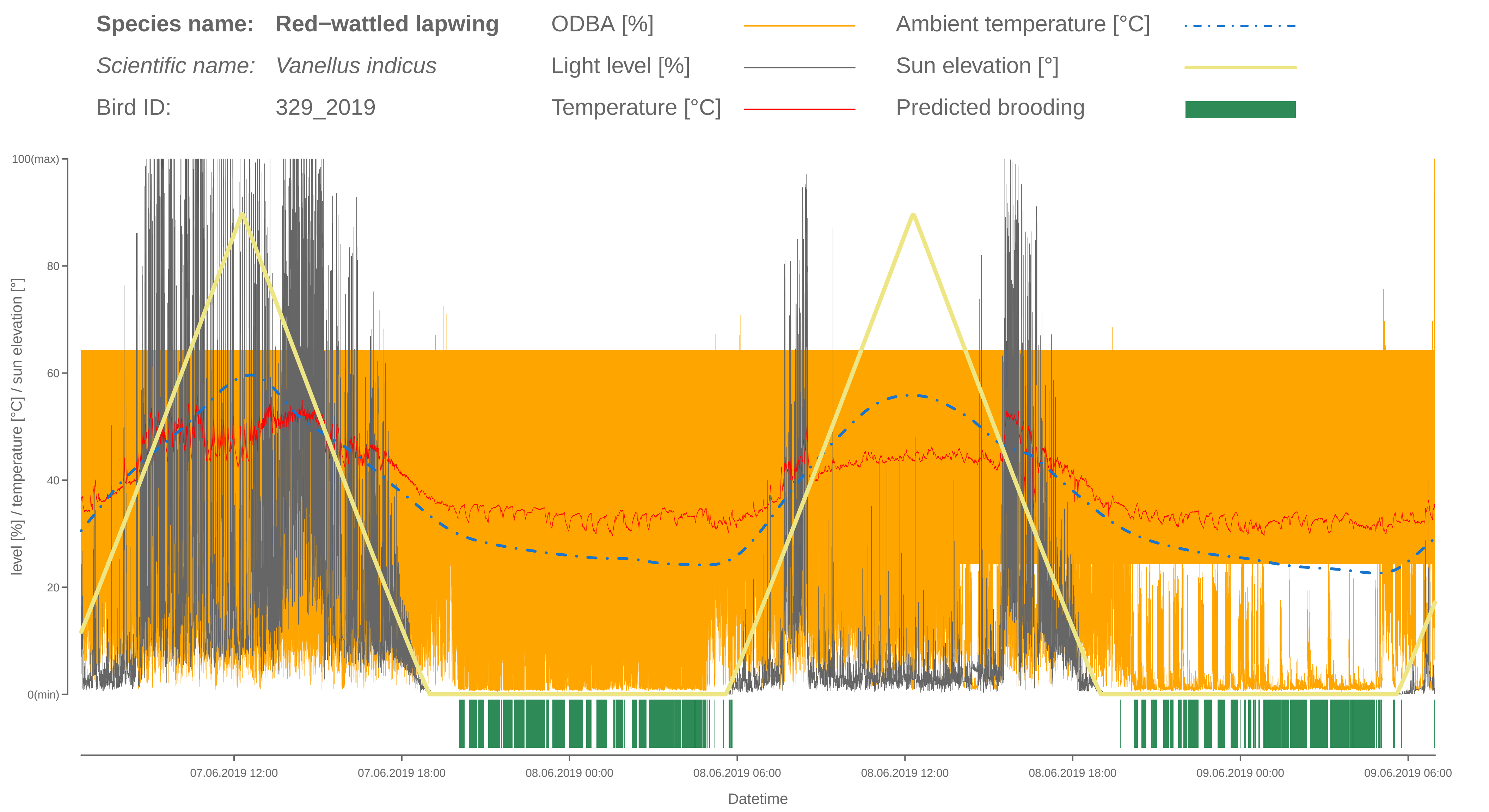

Species name: Red−wattled lapwing
Scientific name: Vanellus indicus
Bird ID: 329_2019
ODBA [%]
Light level [%]
Temperature [°C]
Ambient temperature [°C]
Sun elevation [°]
Predicted brooding
100(max)
80
60
40
20
0(min)
level [%] / temperature [°C] / sun elevation [°]
07.06.2019 12:00
07.06.2019 18:00
08.06.2019 00:00
08.06.2019 06:00
08.06.2019 12:00
08.06.2019 18:00
09.06.2019 00:00
09.06.2019 06:00
Datetime

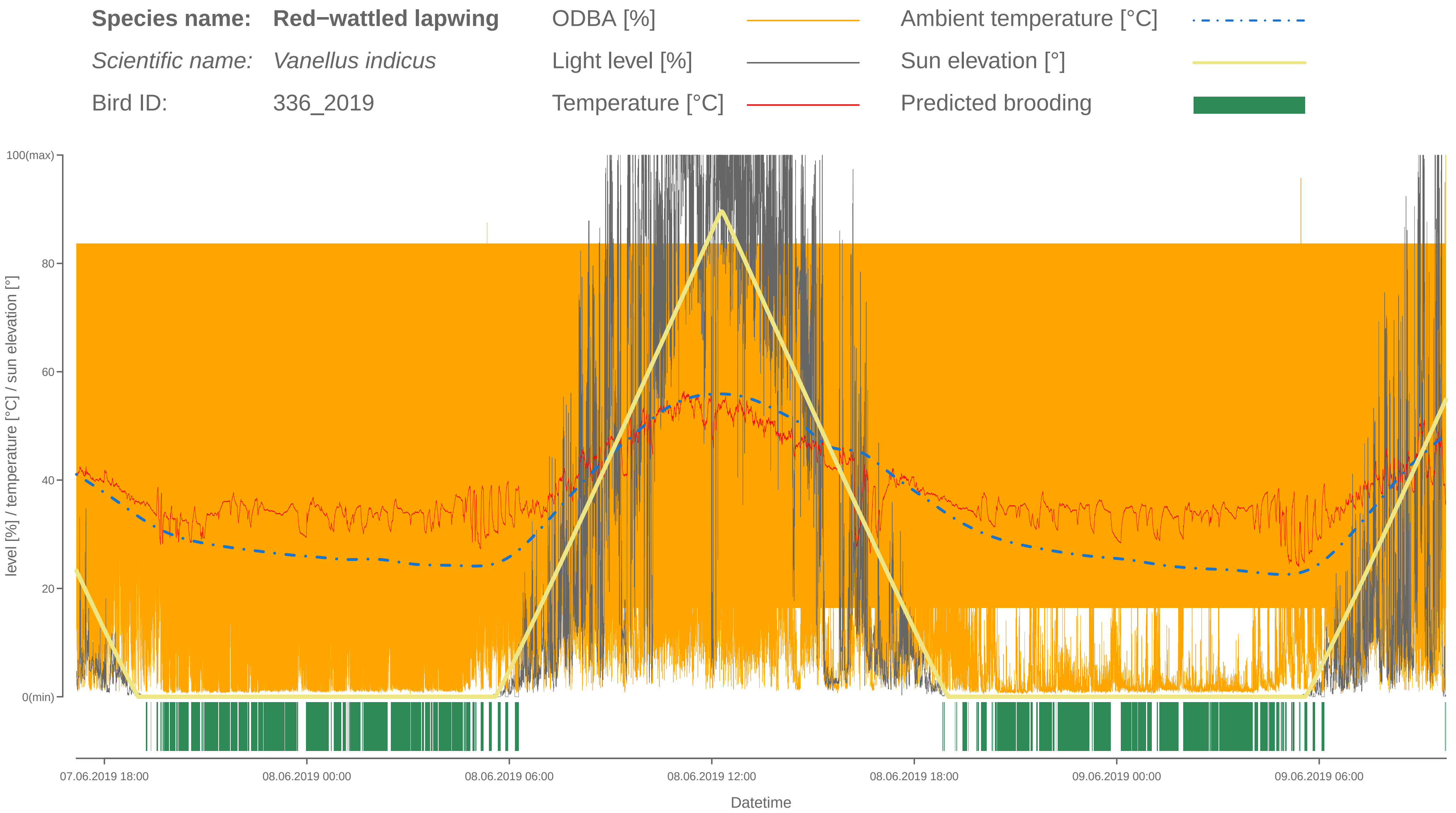

Species name: Red−wattled lapwing
Scientific name: Vanellus indicus
Bird ID: 336_2019
ODBA [%]
Light level [%]
Temperature [°C]
Ambient temperature [°C]
Sun elevation [°]
Predicted brooding
100(max)
80
60
40
20
0(min)
level [%] / temperature [°C] / sun elevation [°]
07.06.2019 18:00
08.06.2019 00:00
08.06.2019 06:00
08.06.2019 12:00
08.06.2019 18:00
09.06.2019 00:00
09.06.2019 06:00
Datetime

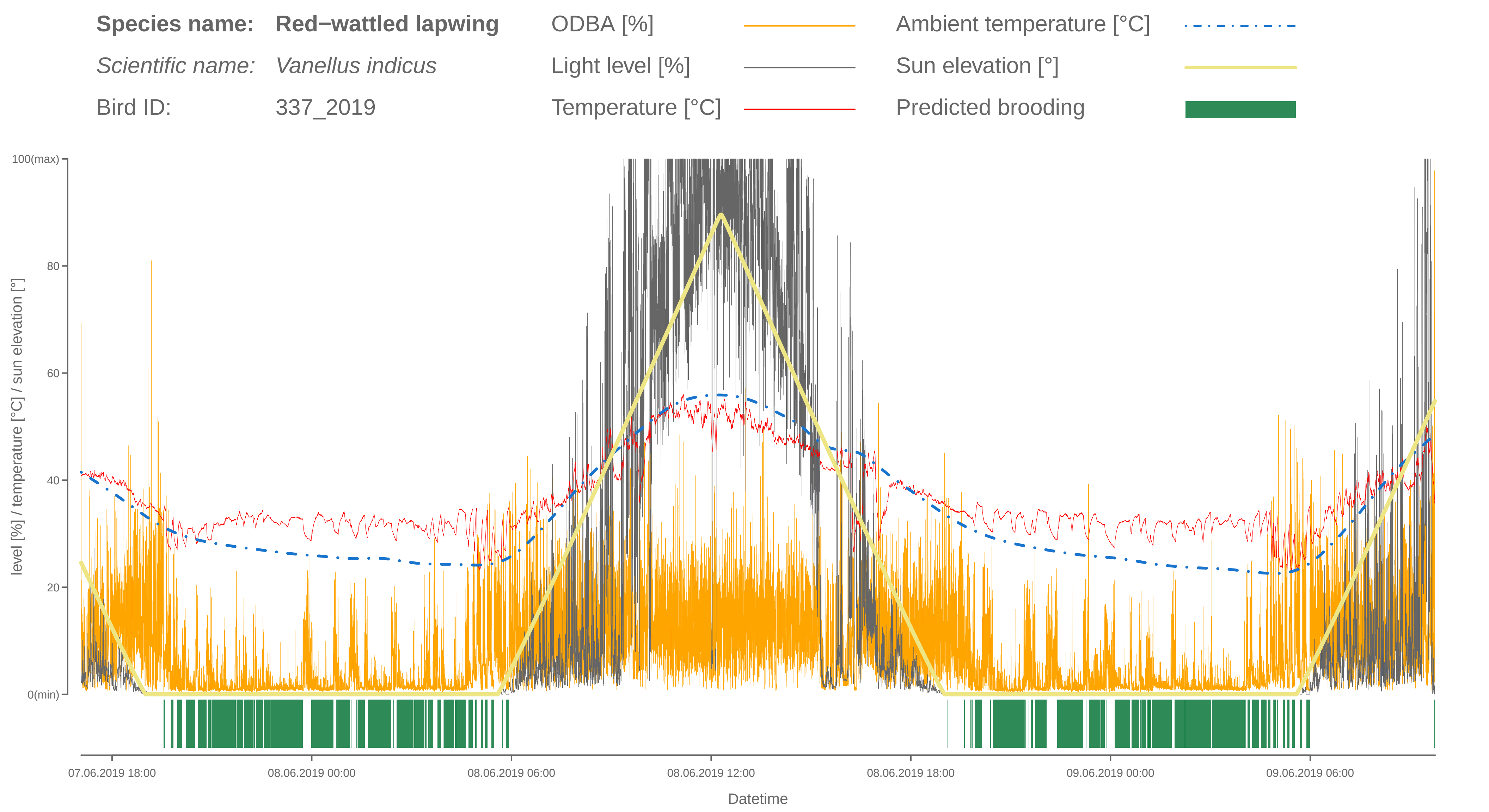

Species name: Red−wattled lapwing
Scientific name: Vanellus indicus
Bird ID: 337_2019
ODBA [%]
Light level [%]
Temperature [°C]
Ambient temperature [°C]
Sun elevation [°]
Predicted brooding
100(max)
80
60
40
20
0(min)
level [%] / temperature [°C] / sun elevation [°]
07.06.2019 18:00
08.06.2019 00:00
08.06.2019 06:00
08.06.2019 12:00
08.06.2019 18:00
09.06.2019 00:00
09.06.2019 06:00
Datetime

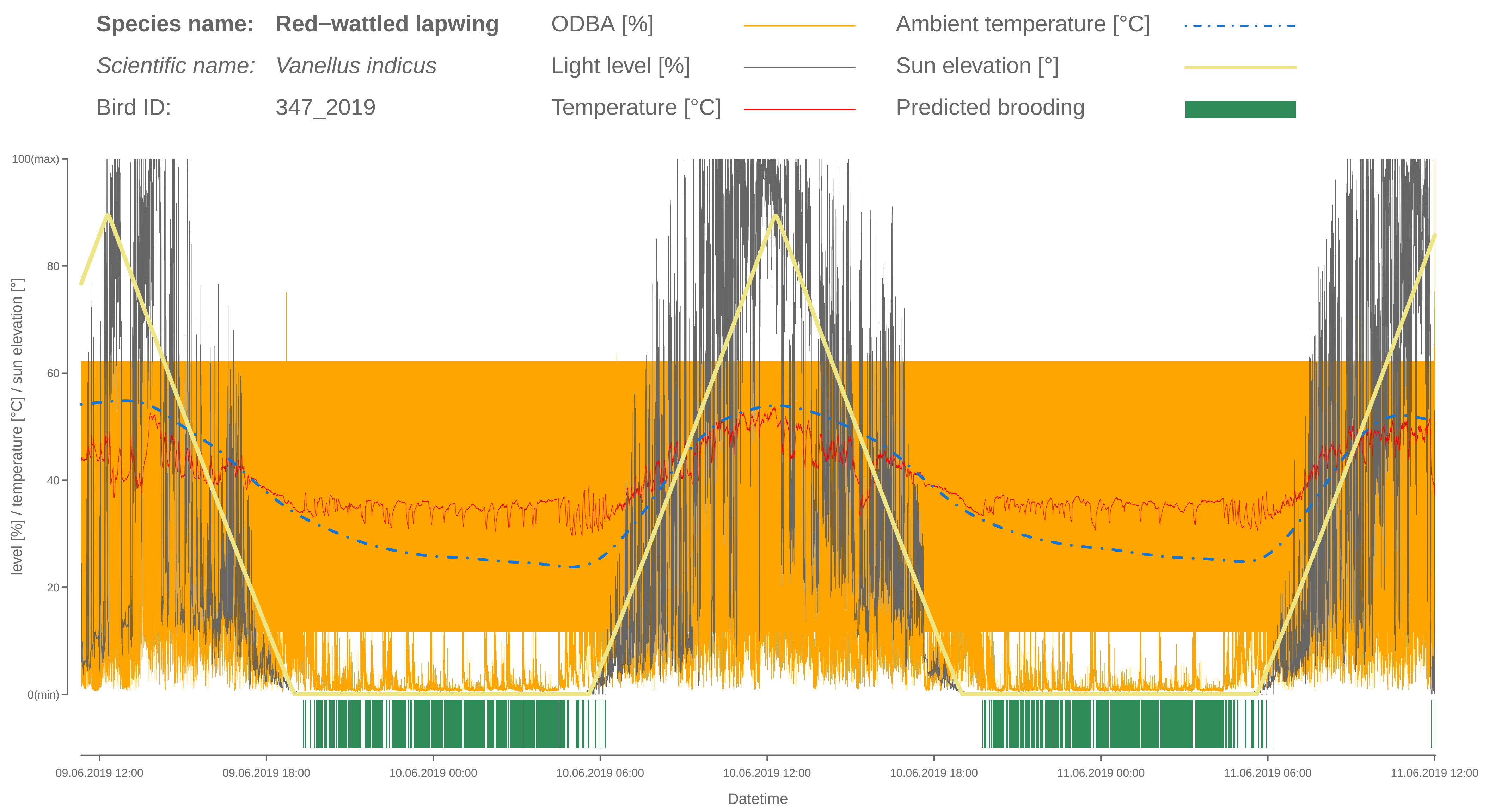

Species name: Red−wattled lapwing
Scientific name: Vanellus indicus
Bird ID: 347_2019
ODBA [%]
Light level [%]
Temperature [°C]
Ambient temperature [°C]
Sun elevation [°]
Predicted brooding
100(max)
80
60
40
20
0(min)
level [%] / temperature [°C] / sun elevation [°]
09.06.2019 12:00
09.06.2019 18:00
10.06.2019 00:00
10.06.2019 06:00
10.06.2019 12:00
10.06.2019 18:00
11.06.2019 00:00
11.06.2019 06:00
11.06.2019 12:00
Datetime

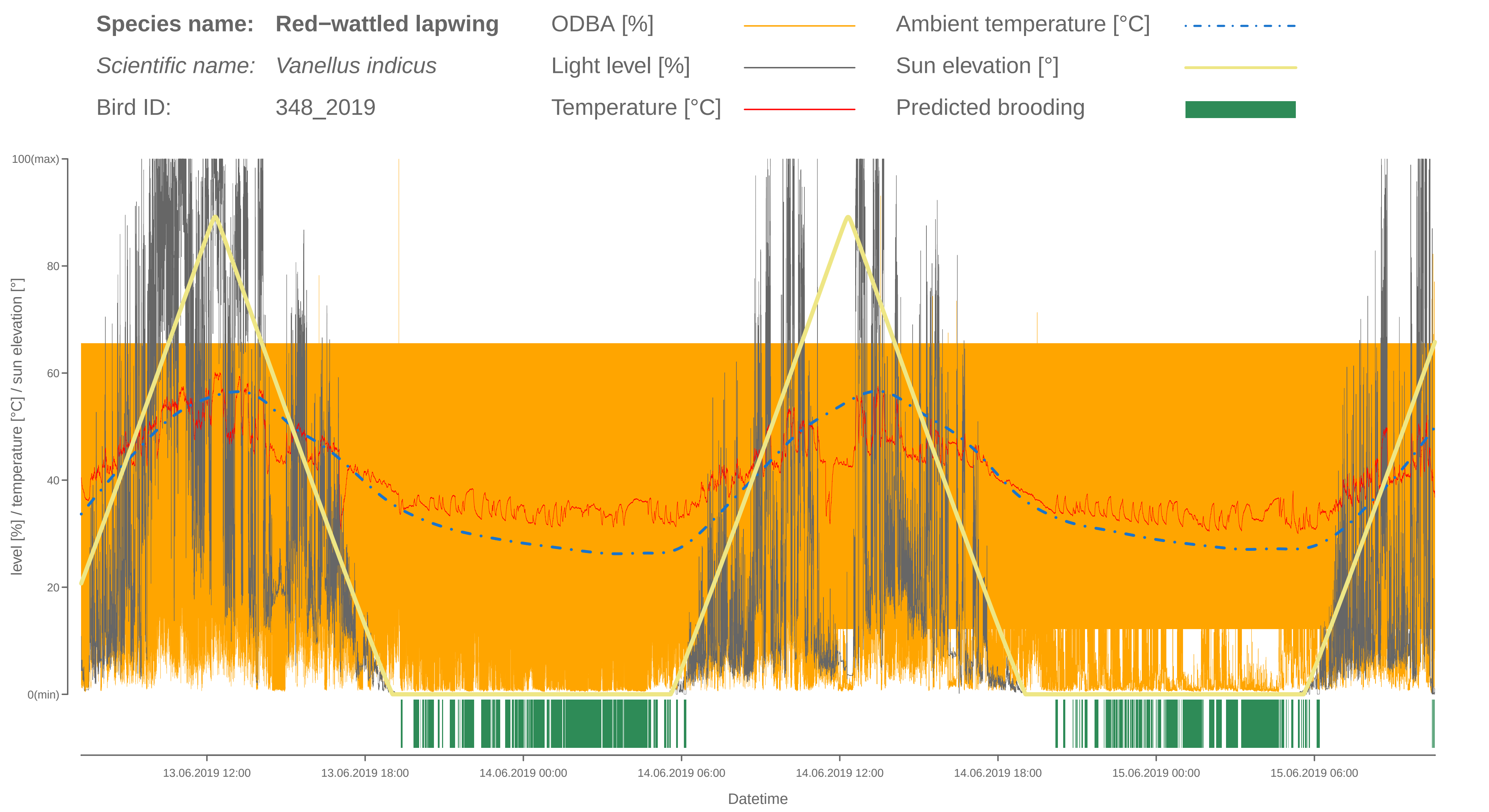
Species name: Red−wattled lapwing
Scientific name: Vanellus indicus
Bird ID: 348_2019
ODBA [%]
Light level [%]
Temperature [°C]
Ambient temperature [°C]
Sun elevation [°]
Predicted brooding
100(max)
80
60
40
20
0(min)
level [%] / temperature [°C] / sun elevation [°]
13.06.2019 12:00
13.06.2019 18:00
14.06.2019 00:00
14.06.2019 06:00
14.06.2019 12:00
14.06.2019 18:00
15.06.2019 00:00
15.06.2019 06:00
Datetime

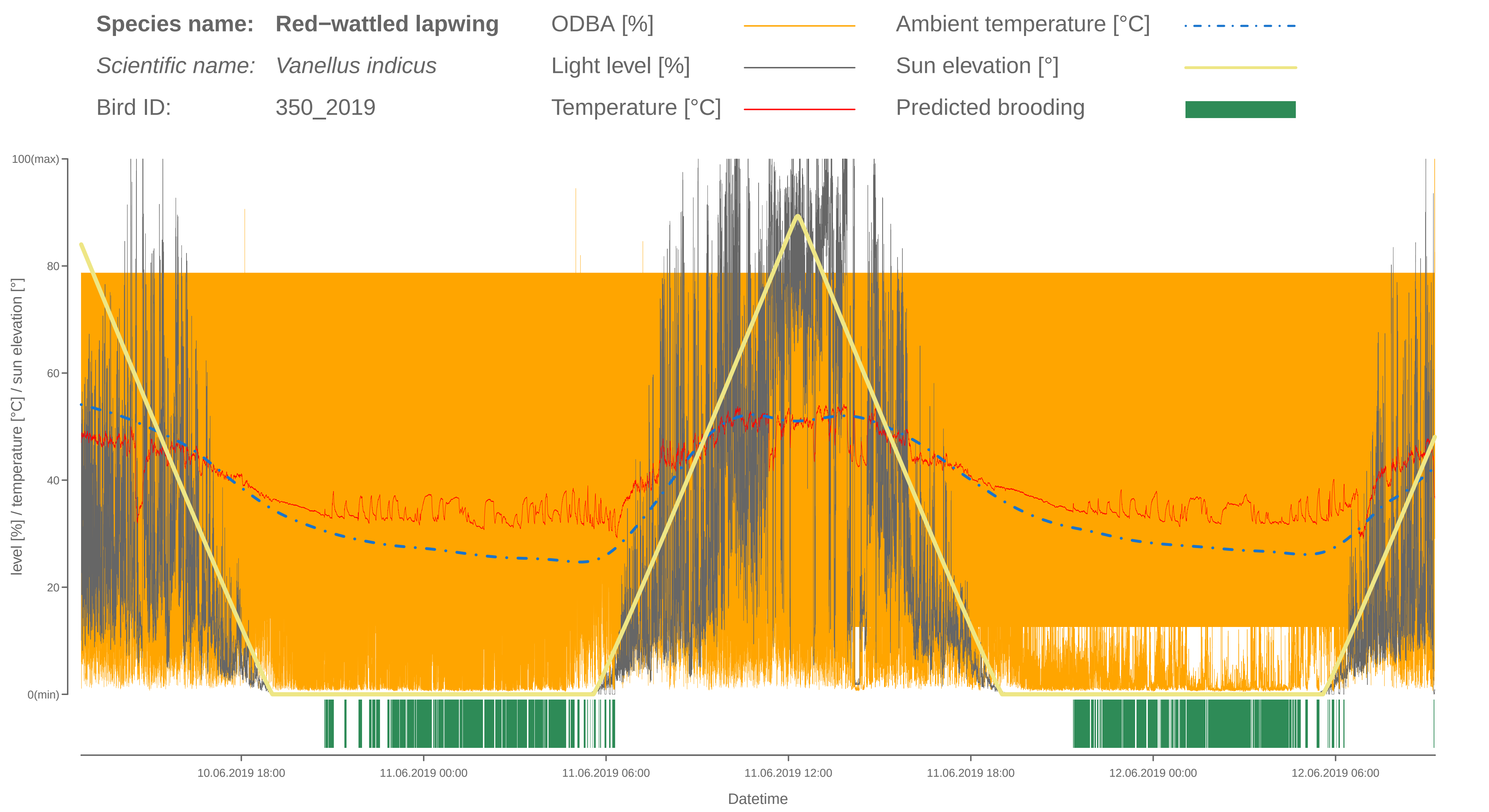
Species name: Red−wattled lapwing
Scientific name: Vanellus indicus
Bird ID: 350_2019
ODBA [%]
Light level [%]
Temperature [°C]
Ambient temperature [°C]
Sun elevation [°]
Predicted brooding
100(max)
80
60
40
20
0(min)
level [%] / temperature [°C] / sun elevation [°]
10.06.2019 18:00
11.06.2019 00:00
11.06.2019 06:00
11.06.2019 12:00
11.06.2019 18:00
12.06.2019 00:00
12.06.2019 06:00
Datetime

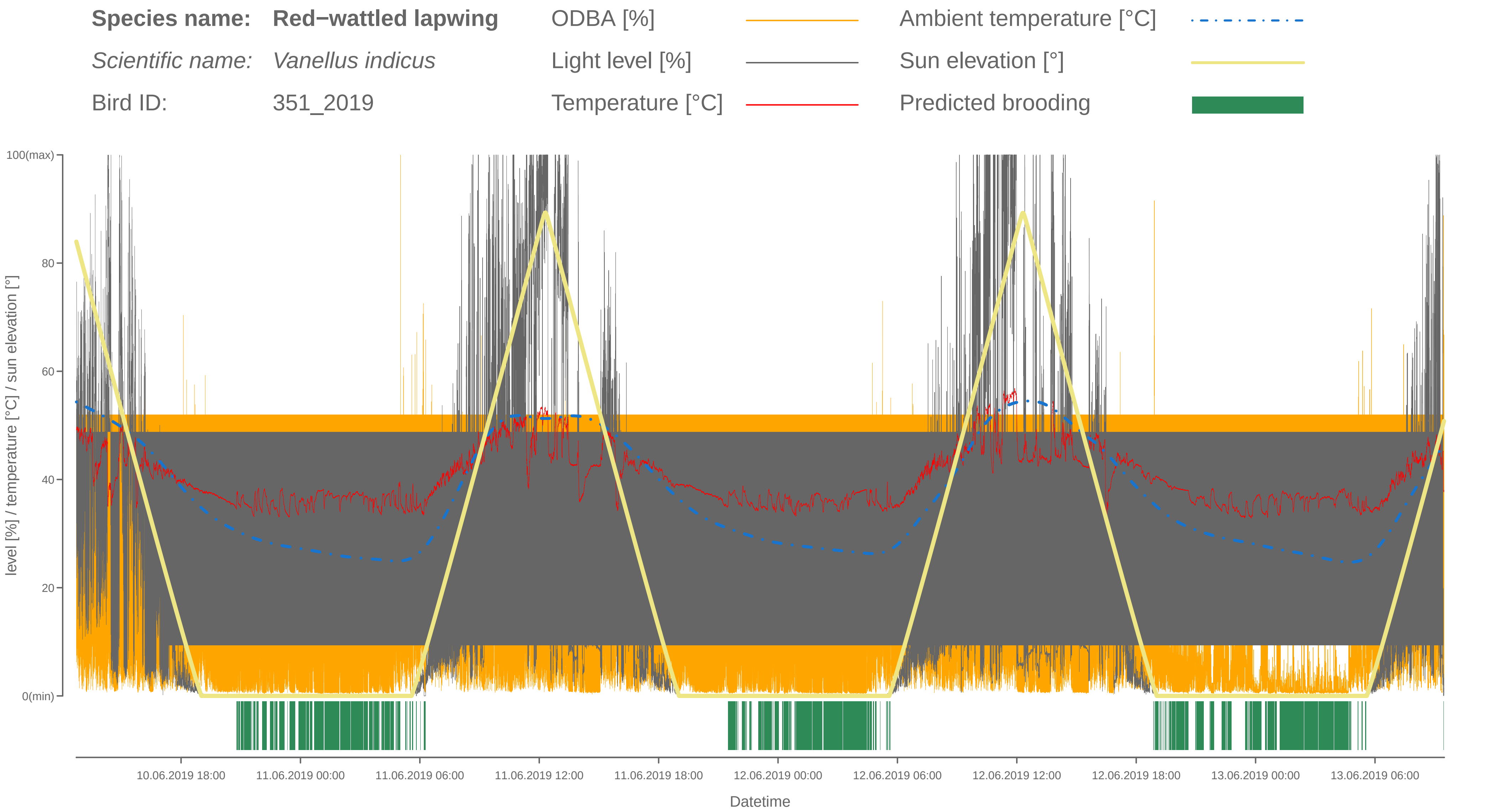
Species name: Red−wattled lapwing
Scientific name: Vanellus indicus
Bird ID: 351_2019
ODBA [%]
Light level [%]
Temperature [°C]
Ambient temperature [°C]
Sun elevation [°]
Predicted brooding
100(max)
80
60
40
20
0(min)
level [%] / temperature [°C] / sun elevation [°]
10.06.2019 18:00
11.06.2019 00:00
11.06.2019 06:00
11.06.2019 12:00
11.06.2019 18:00
12.06.2019 00:00
12.06.2019 06:00
12.06.2019 12:00
12.06.2019 18:00
13.06.2019 00:00
13.06.2019 06:00
Datetime

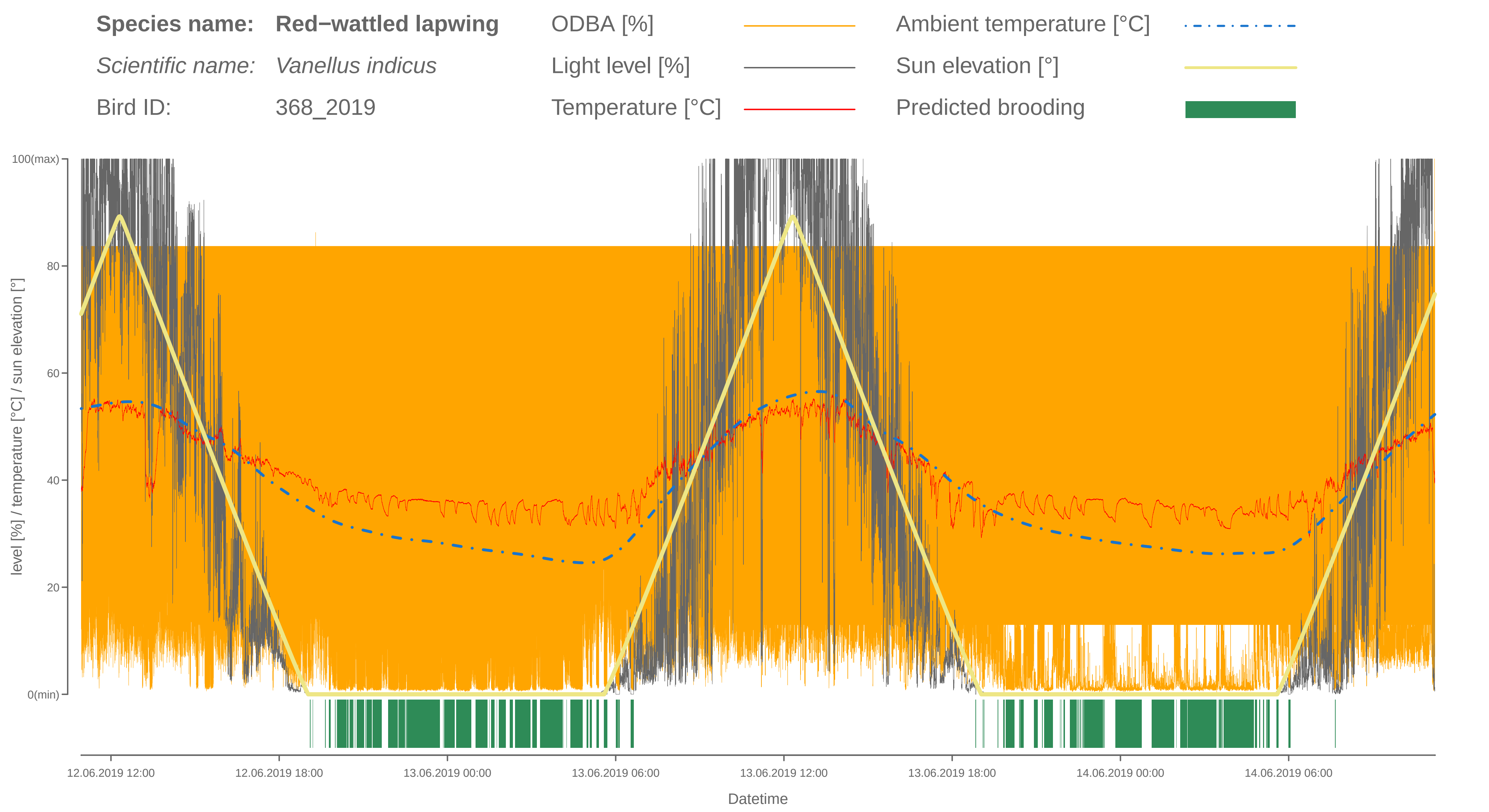

Species name: Red−wattled lapwing
Scientific name: Vanellus indicus
Bird ID: 368_2019
ODBA [%]
Light level [%]
Temperature [°C]
Ambient temperature [°C]
Sun elevation [°]
Predicted brooding
100(max)
80
60
40
20
0(min)
level [%] / temperature [°C] / sun elevation [°]
12.06.2019 12:00
12.06.2019 18:00
13.06.2019 00:00
13.06.2019 06:00
13.06.2019 12:00
13.06.2019 18:00
14.06.2019 00:00
14.06.2019 06:00
Datetime

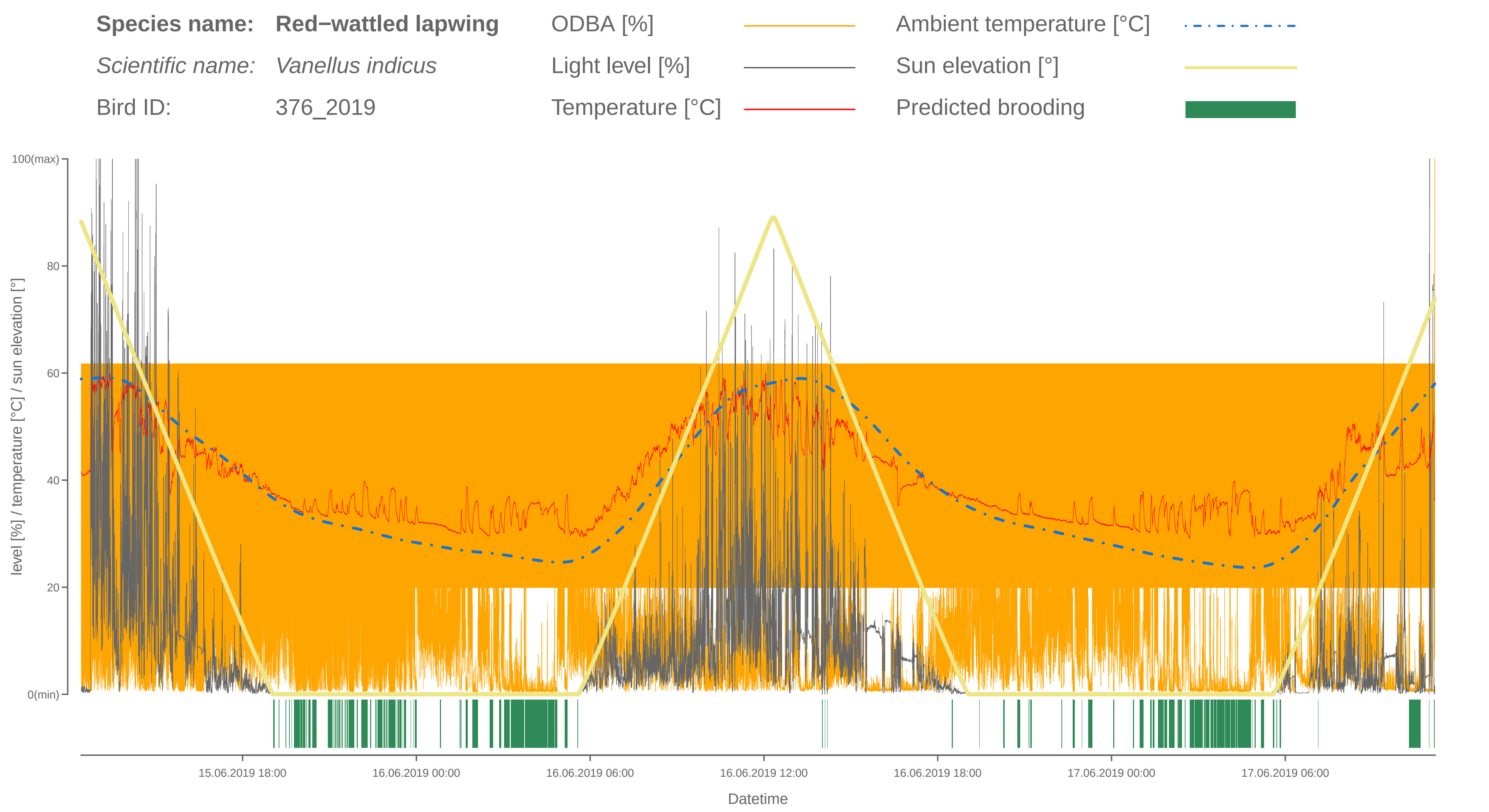
Species name: Red−wattled lapwing
Scientific name: Vanellus indicus
Bird ID: 376_2019
ODBA [%]
Light level [%]
Temperature [°C]
Ambient temperature [°C]
Sun elevation [°]
Predicted brooding
100(max)
80
60
40
20
0(min)
level [%] / temperature [°C] / sun elevation [°]
15.06.2019 18:00
16.06.2019 00:00
16.06.2019 06:00
16.06.2019 12:00
16.06.2019 18:00
17.06.2019 00:00
17.06.2019 06:00
Datetime

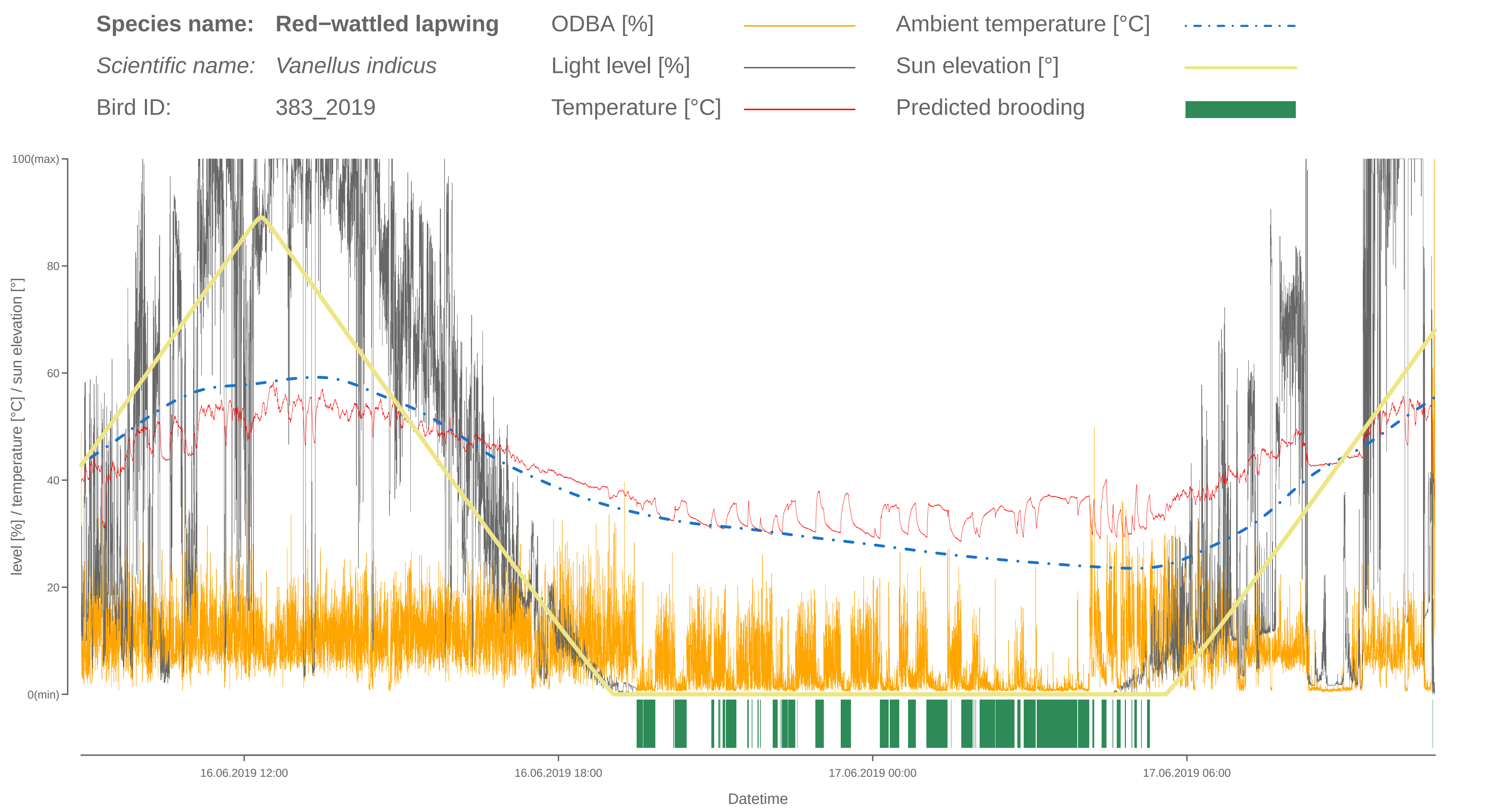
Species name: Red−wattled lapwing
Scientific name: Vanellus indicus
Bird ID: 383_2019
ODBA [%]
Light level [%]
Temperature [°C]
Ambient temperature [°C]
Sun elevation [°]
Predicted brooding
100(max)
80
60
40
20
0(min)
level [%] / temperature [°C] / sun elevation [°]
16.06.2019 12:00
16.06.2019 18:00
17.06.2019 00:00
17.06.2019 06:00
Datetime

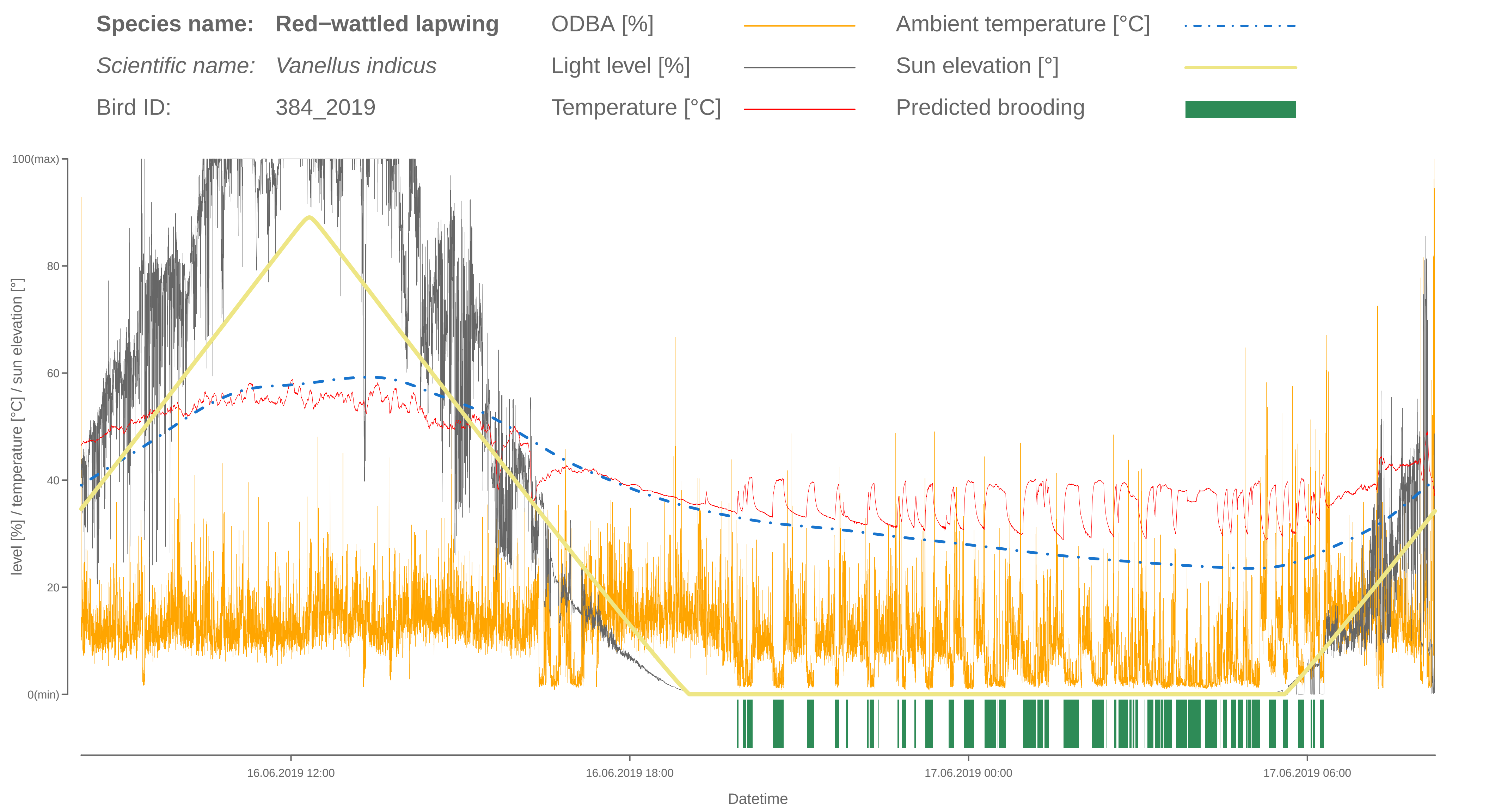
Species name: Red−wattled lapwing
Scientific name: Vanellus indicus
Bird ID: 384_2019
ODBA [%]
Light level [%]
Temperature [°C]
Ambient temperature [°C]
Sun elevation [°]
Predicted brooding
100(max)
80
60
40
20
0(min)
level [%] / temperature [°C] / sun elevation [°]
16.06.2019 12:00
16.06.2019 18:00
17.06.2019 00:00
17.06.2019 06:00
Datetime

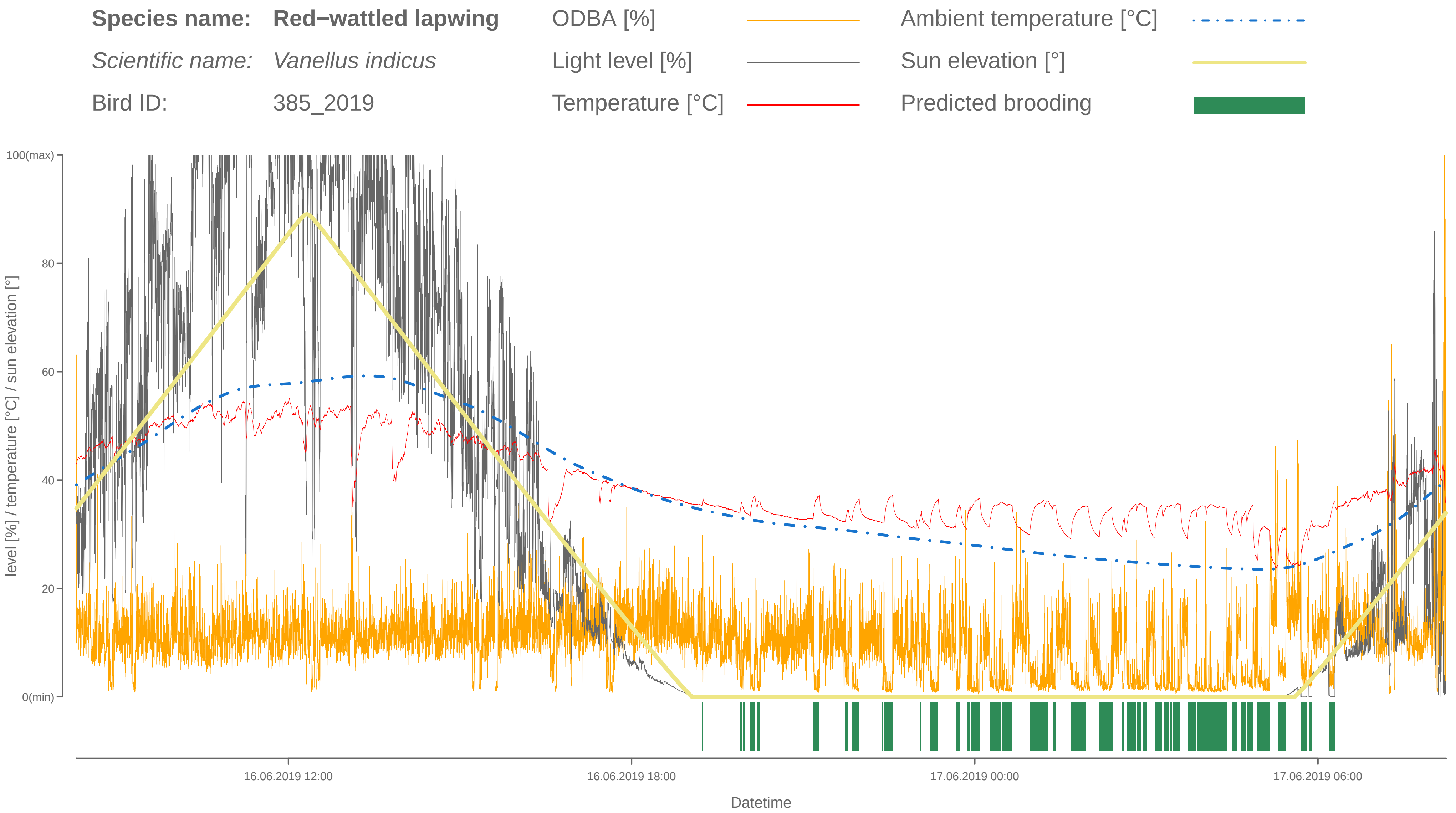

Species name: Red−wattled lapwing
Scientific name: Vanellus indicus
Bird ID: 385_2019
ODBA [%]
Light level [%]
Temperature [°C]
Ambient temperature [°C]
Sun elevation [°]
Predicted brooding
100(max)
80
60
40
20
0(min)
level [%] / temperature [°C] / sun elevation [°]
16.06.2019 12:00
16.06.2019 18:00
17.06.2019 00:00
17.06.2019 06:00
Datetime

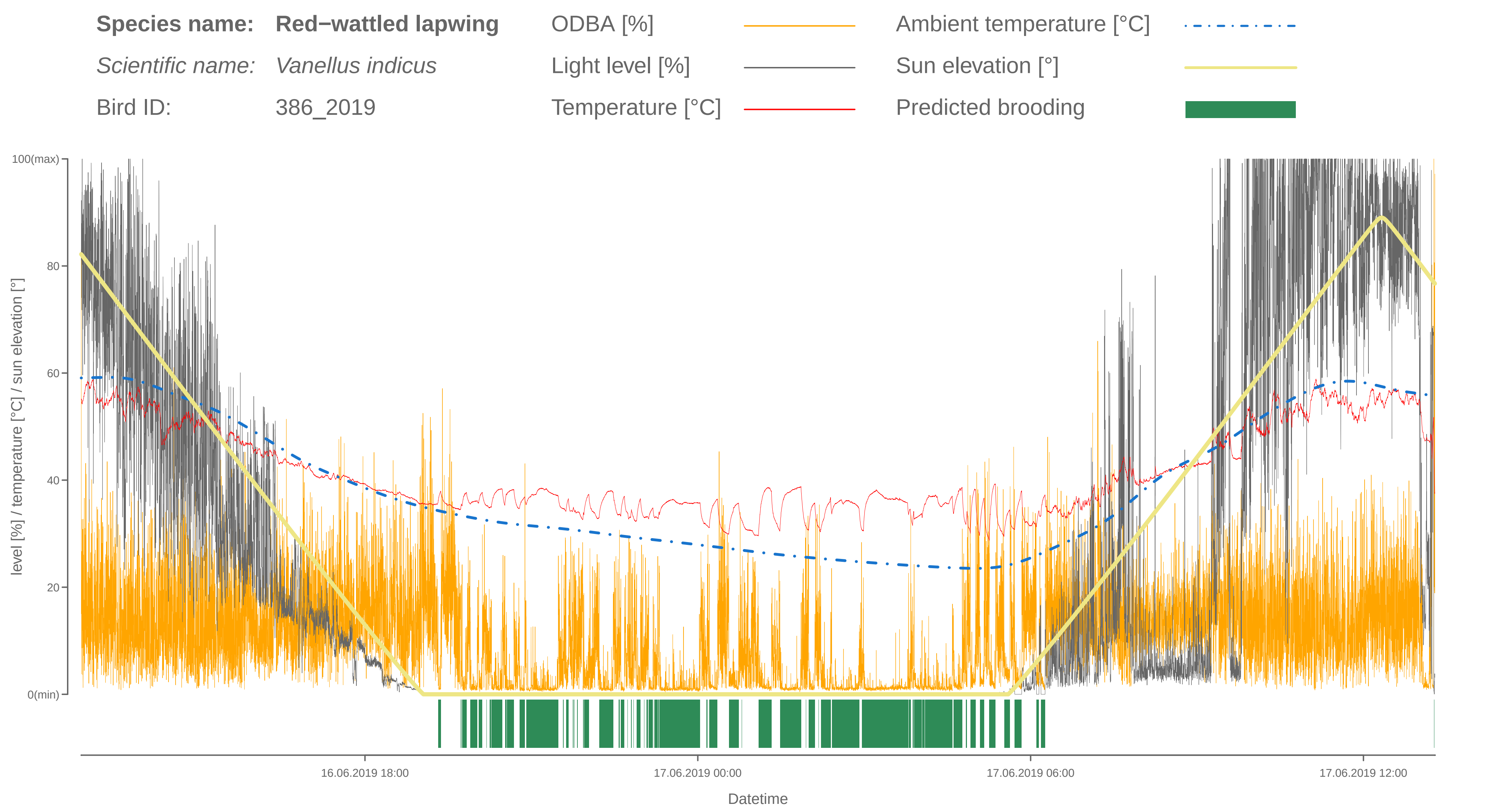

Species name: Red−wattled lapwing
Scientific name: Vanellus indicus
Bird ID: 386_2019
ODBA [%]
Light level [%]
Temperature [°C]
Ambient temperature [°C]
Sun elevation [°]
Predicted brooding
100(max)
80
60
40
20
0(min)
level [%] / temperature [°C] / sun elevation [°]
16.06.2019 18:00
17.06.2019 00:00
17.06.2019 06:00
17.06.2019 12:00
Datetime

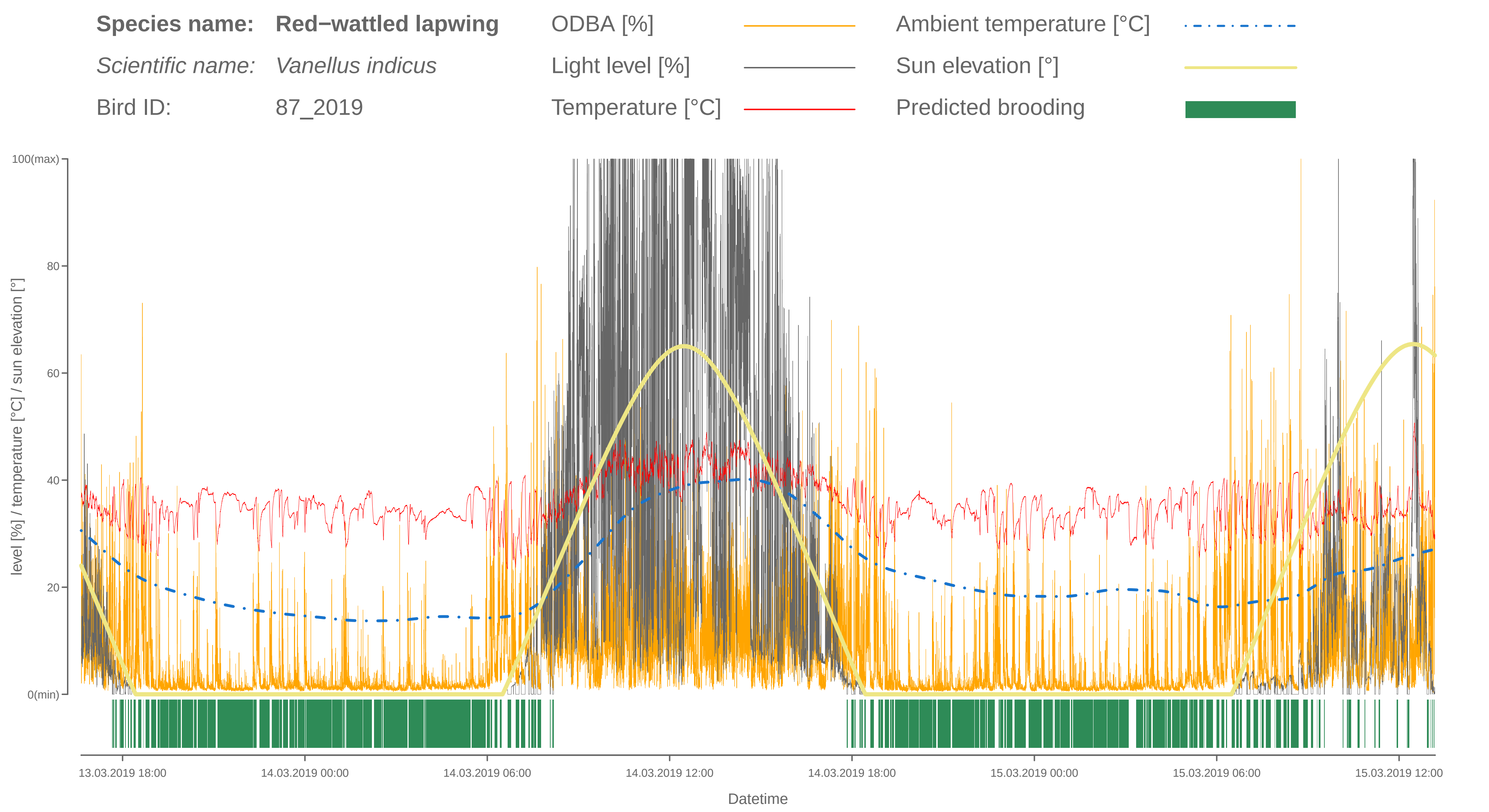
Species name: Red−wattled lapwing
Scientific name: Vanellus indicus
Bird ID: 87_2019
ODBA [%]
Light level [%]
Temperature [°C]
Ambient temperature [°C]
Sun elevation [°]
Predicted brooding
100(max)
80
60
40
20
0(min)
level [%] / temperature [°C] / sun elevation [°]
13.03.2019 18:00
14.03.2019 00:00
14.03.2019 06:00
14.03.2019 12:00
14.03.2019 18:00
15.03.2019 00:00
15.03.2019 06:00
15.03.2019 12:00
Datetime

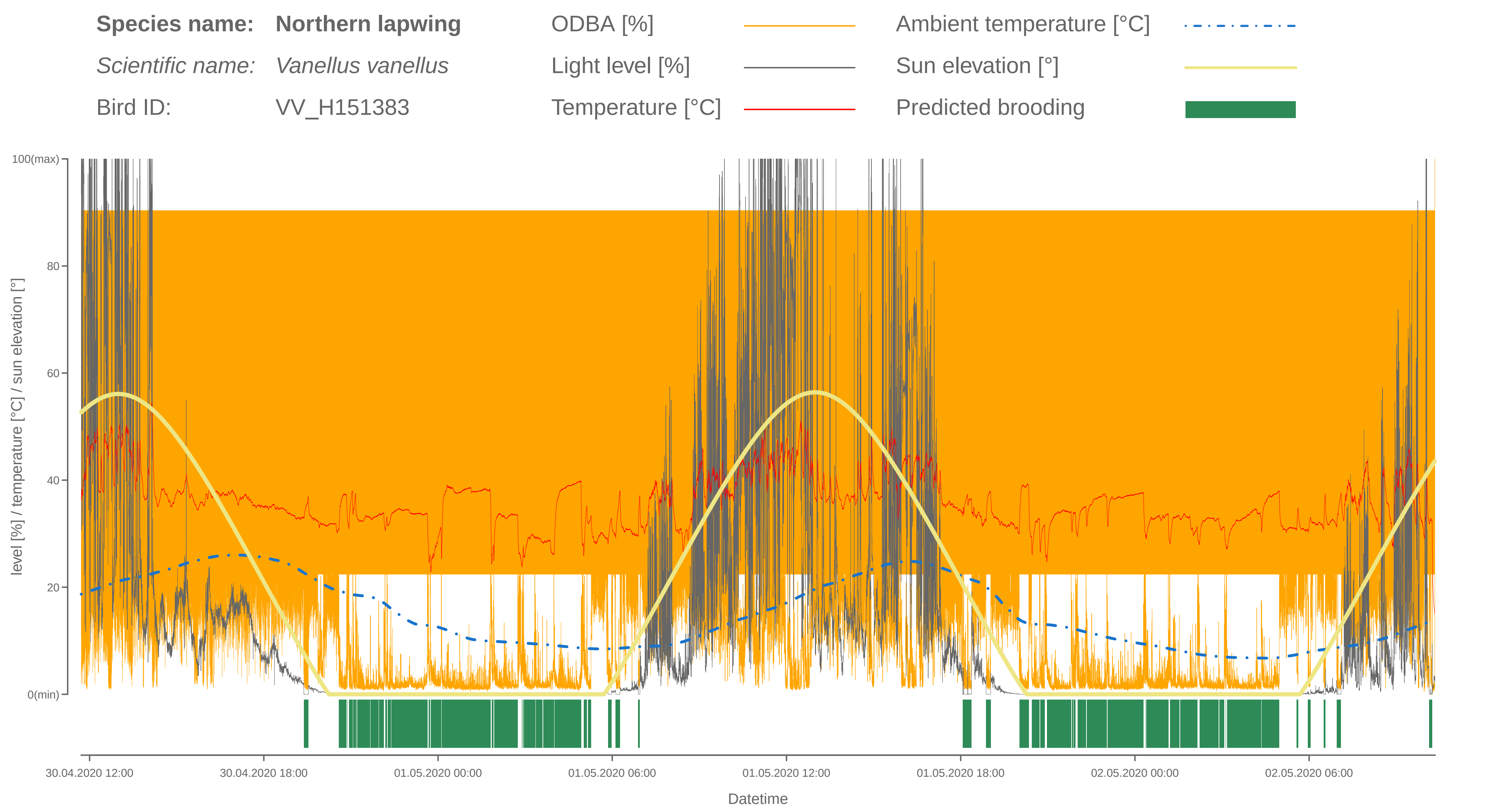
Species name: Northern lapwing
Scientific name: Vanellus vanellus
Bird ID: VV_H151383
ODBA [%]
Light level [%]
Temperature [°C]
Ambient temperature [°C]
Sun elevation [°]
Predicted brooding
100(max)
80
60
40
20
0(min)
level [%] / temperature [°C] / sun elevation [°]
30.04.2020 12:00
30.04.2020 18:00
01.05.2020 00:00
01.05.2020 06:00
01.05.2020 12:00
01.05.2020 18:00
02.05.2020 00:00
02.05.2020 06:00
Datetime

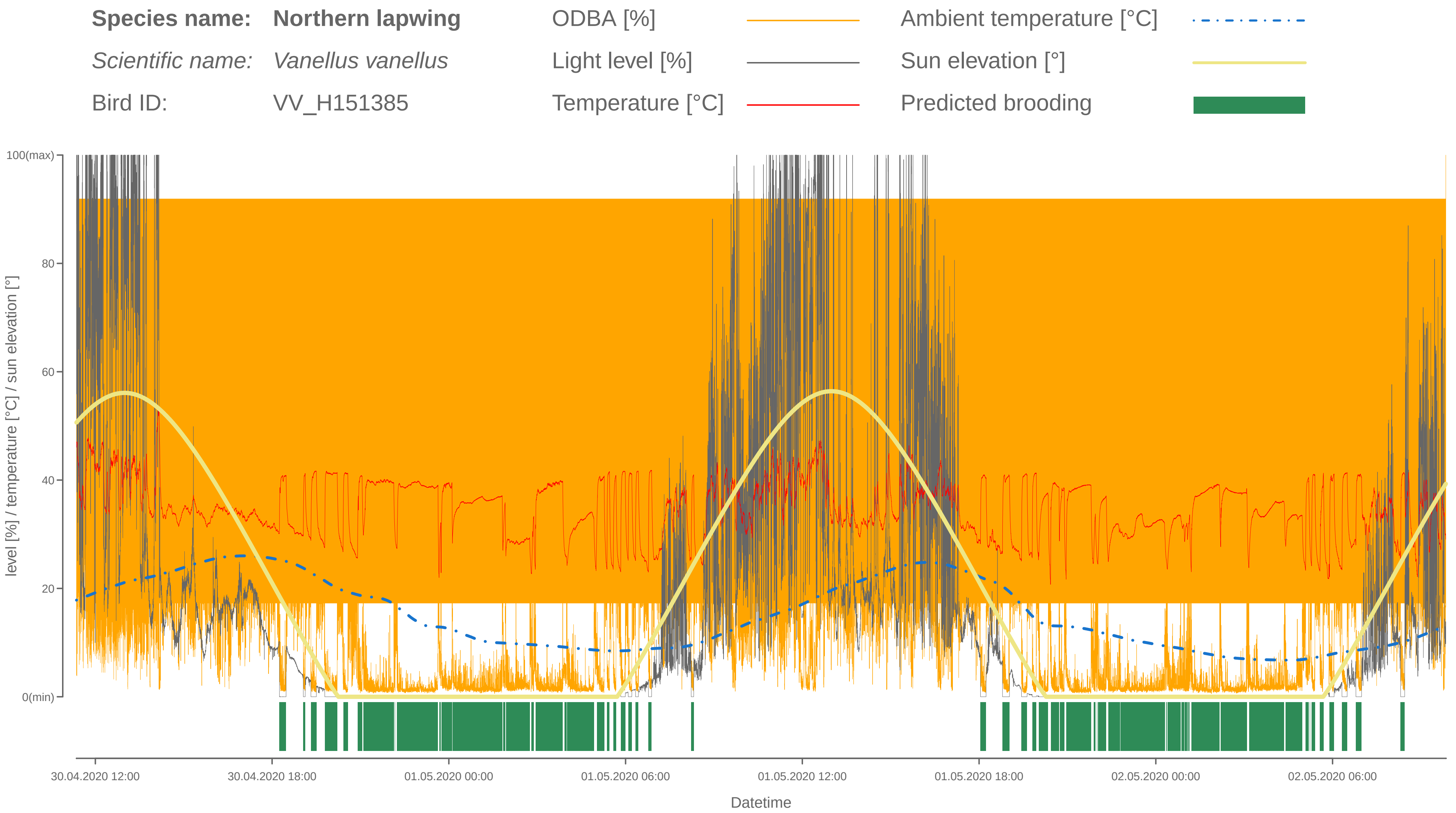
Species name: Northern lapwing
Scientific name: Vanellus vanellus
Bird ID: VV_H151385
ODBA [%]
Light level [%]
Temperature [°C]
Ambient temperature [°C]
Sun elevation [°]
Predicted brooding
100(max)
80
60
40
20
0(min)
level [%] / temperature [°C] / sun elevation [°]
30.04.2020 12:00
30.04.2020 18:00
01.05.2020 00:00
01.05.2020 06:00
01.05.2020 12:00
01.05.2020 18:00
02.05.2020 00:00
02.05.2020 06:00
Datetime

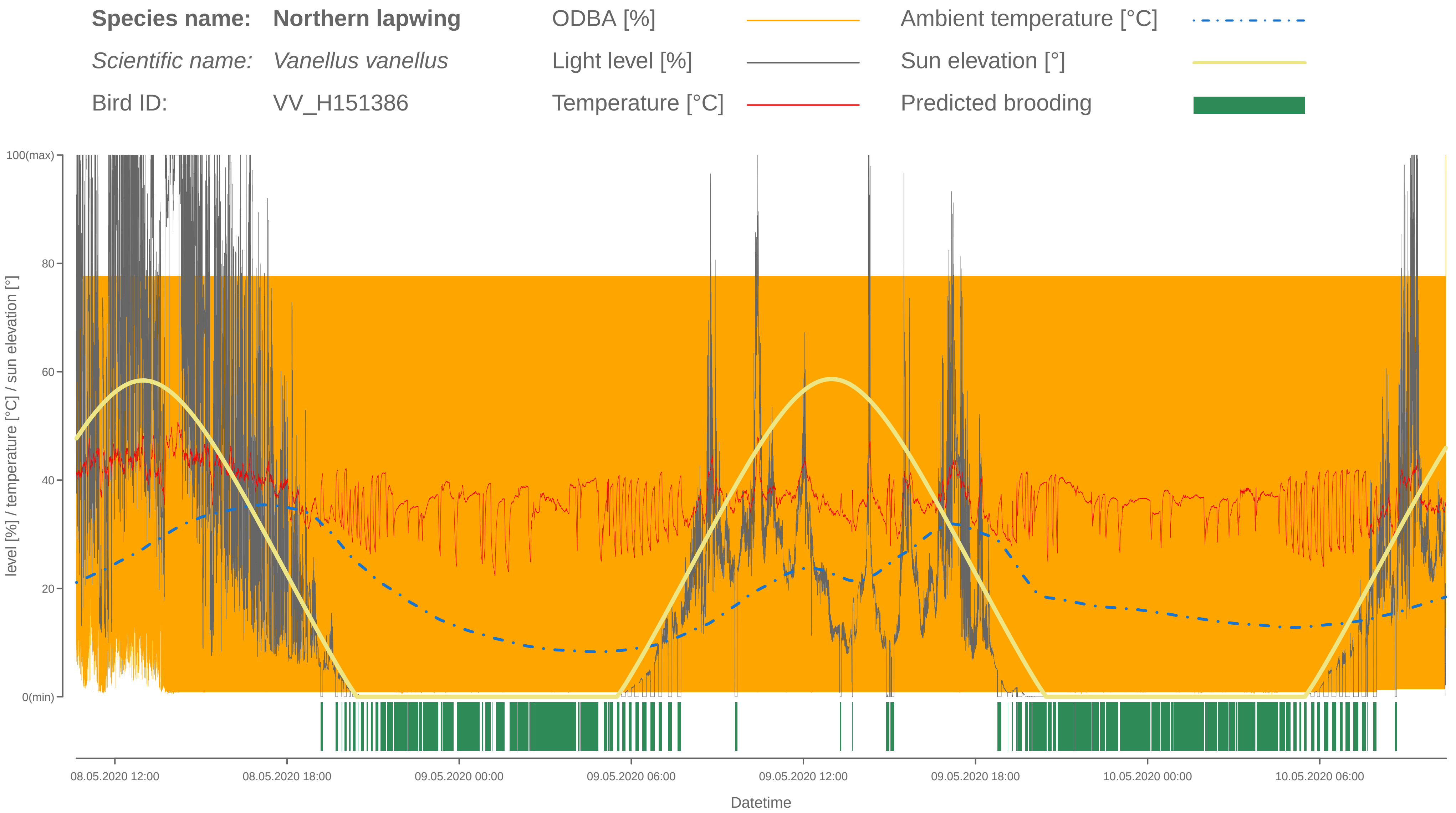

Species name: Northern lapwing
Scientific name: Vanellus vanellus
Bird ID: VV_H151386
ODBA [%]
Light level [%]
Temperature [°C]
Ambient temperature [°C]
Sun elevation [°]
Predicted brooding
100(max)
80
60
40
20
0(min)
level [%] / temperature [°C] / sun elevation [°]
08.05.2020 12:00
08.05.2020 18:00
09.05.2020 00:00
09.05.2020 06:00
09.05.2020 12:00
09.05.2020 18:00
10.05.2020 00:00
10.05.2020 06:00
Datetime

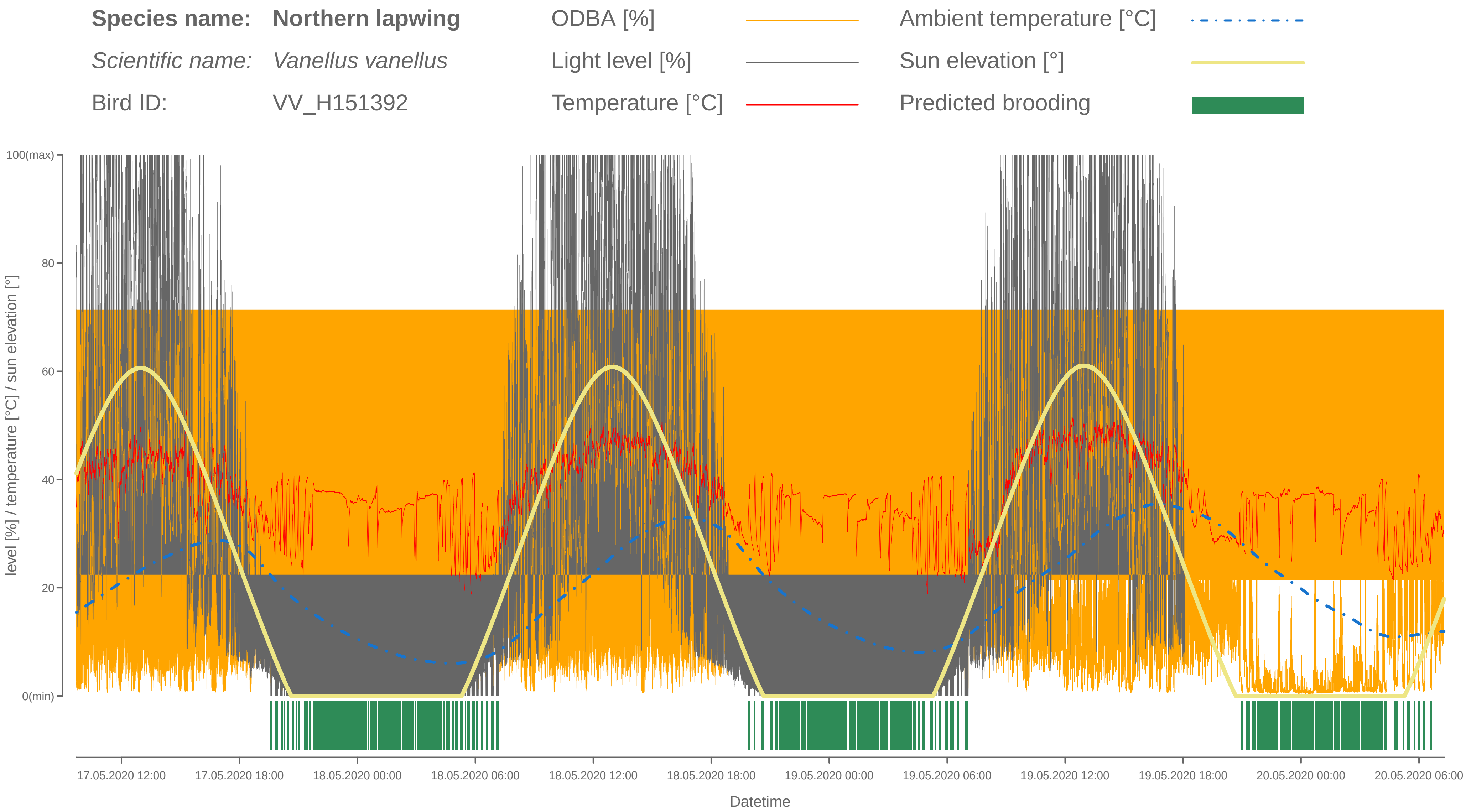

Species name: Northern lapwing
Scientific name: Vanellus vanellus
Bird ID: VV_H151392
ODBA [%]
Light level [%]
Temperature [°C]
Ambient temperature [°C]
Sun elevation [°]
Predicted brooding
level [%] / temperature [°C] / sun elevation [°]
100(max)
80
60
40
20
0(min)
17.05.2020 12:00
17.05.2020 18:00
18.05.2020 00:00
18.05.2020 06:00
18.05.2020 12:00
18.05.2020 18:00
19.05.2020 00:00
19.05.2020 06:00
19.05.2020 12:00
19.05.2020 18:00
20.05.2020 00:00
20.05.2020 06:00
Datetime

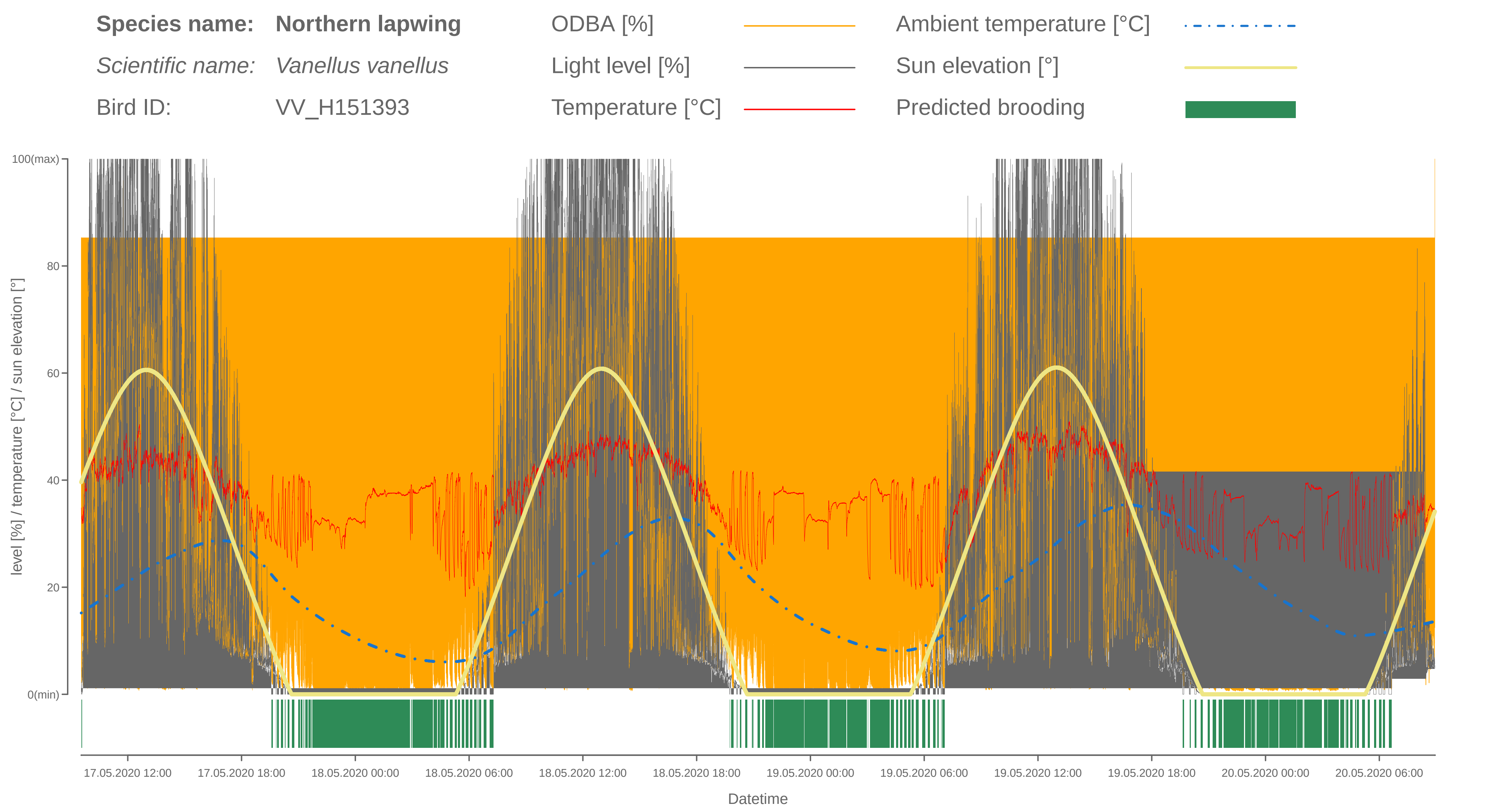

Species name: Northern lapwing
Scientific name: Vanellus vanellus
Bird ID: VV_H151393
ODBA [%]
Light level [%]
Temperature [°C]
Ambient temperature [°C]
Sun elevation [°]
Predicted brooding
100(max)
80
60
40
20
0(min)
level [%] / temperature [°C] / sun elevation [°]
17.05.2020 12:00
17.05.2020 18:00
18.05.2020 00:00
18.05.2020 06:00
18.05.2020 12:00
18.05.2020 18:00
19.05.2020 00:00
19.05.2020 06:00
19.05.2020 12:00
19.05.2020 18:00
20.05.2020 00:00
20.05.2020 06:00
Datetime

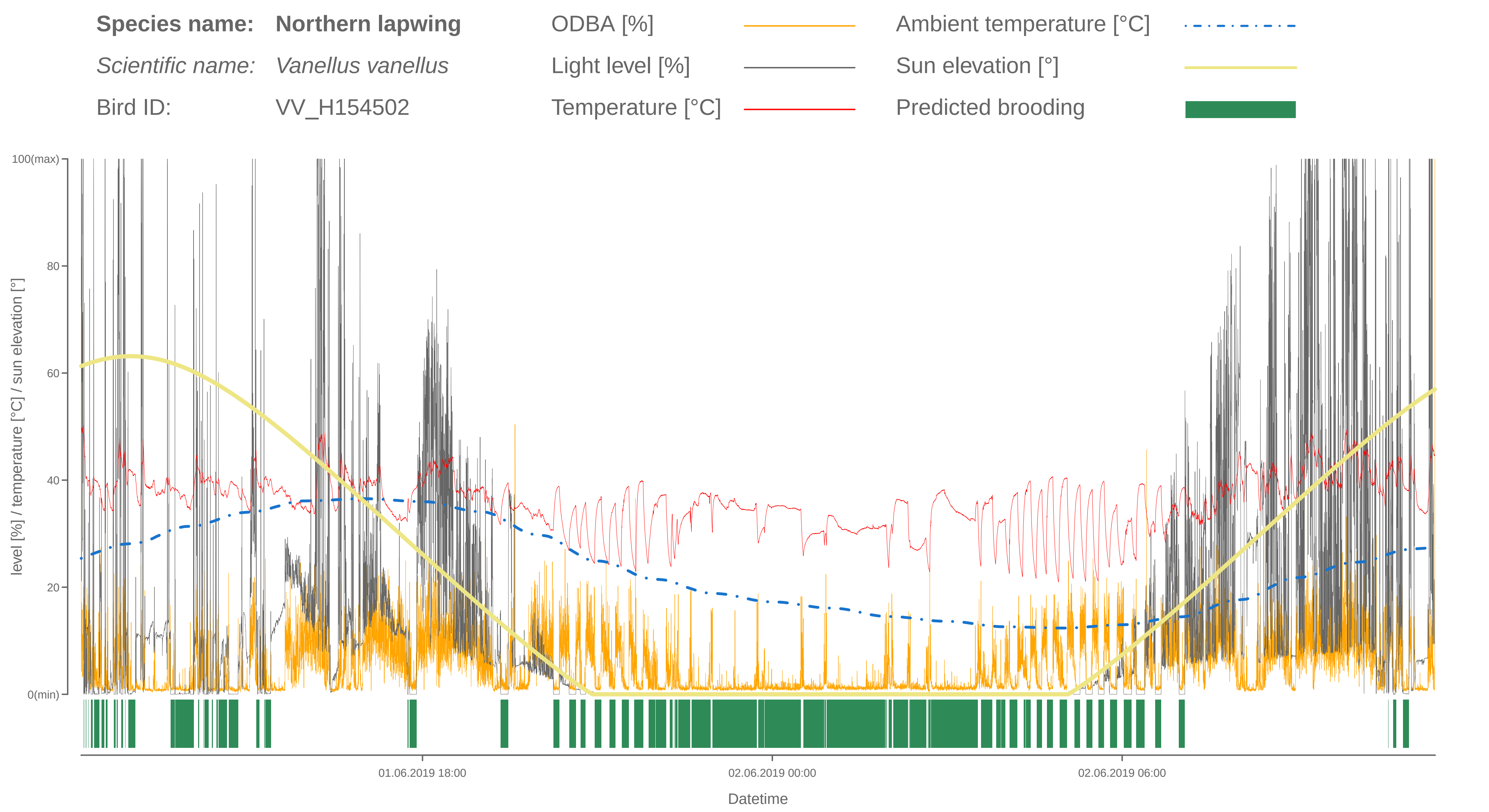
Species name: Northern lapwing
Scientific name: Vanellus vanellus
Bird ID: VV_H154502
ODBA [%]
Light level [%]
Temperature [°C]
Ambient temperature [°C]
Sun elevation [°]
Predicted brooding
100(max)
80
60
40
20
0(min)
level [%] / temperature [°C] / sun elevation [°]
01.06.2019 18:00
02.06.2019 00:00
02.06.2019 06:00
Datetime

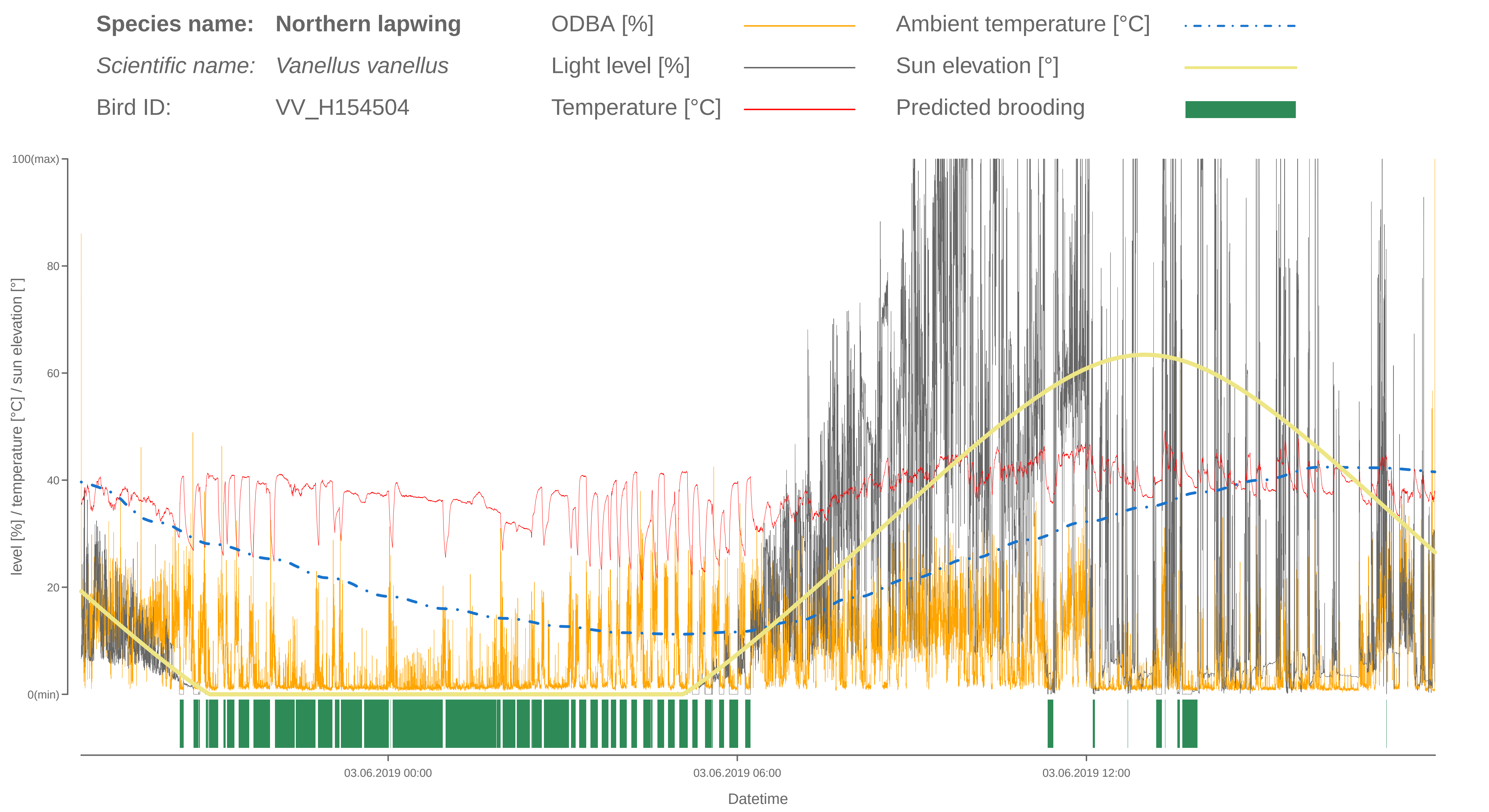
Species name: Northern lapwing
Scientific name: Vanellus vanellus
Bird ID: VV_H154504
ODBA [%]
Light level [%]
Temperature [°C]
Ambient temperature [°C]
Sun elevation [°]
Predicted brooding
100(max)
80
60
40
20
0(min)
level [%] / temperature [°C] / sun elevation [°]
03.06.2019 00:00
03.06.2019 06:00
03.06.2019 12:00
Datetime

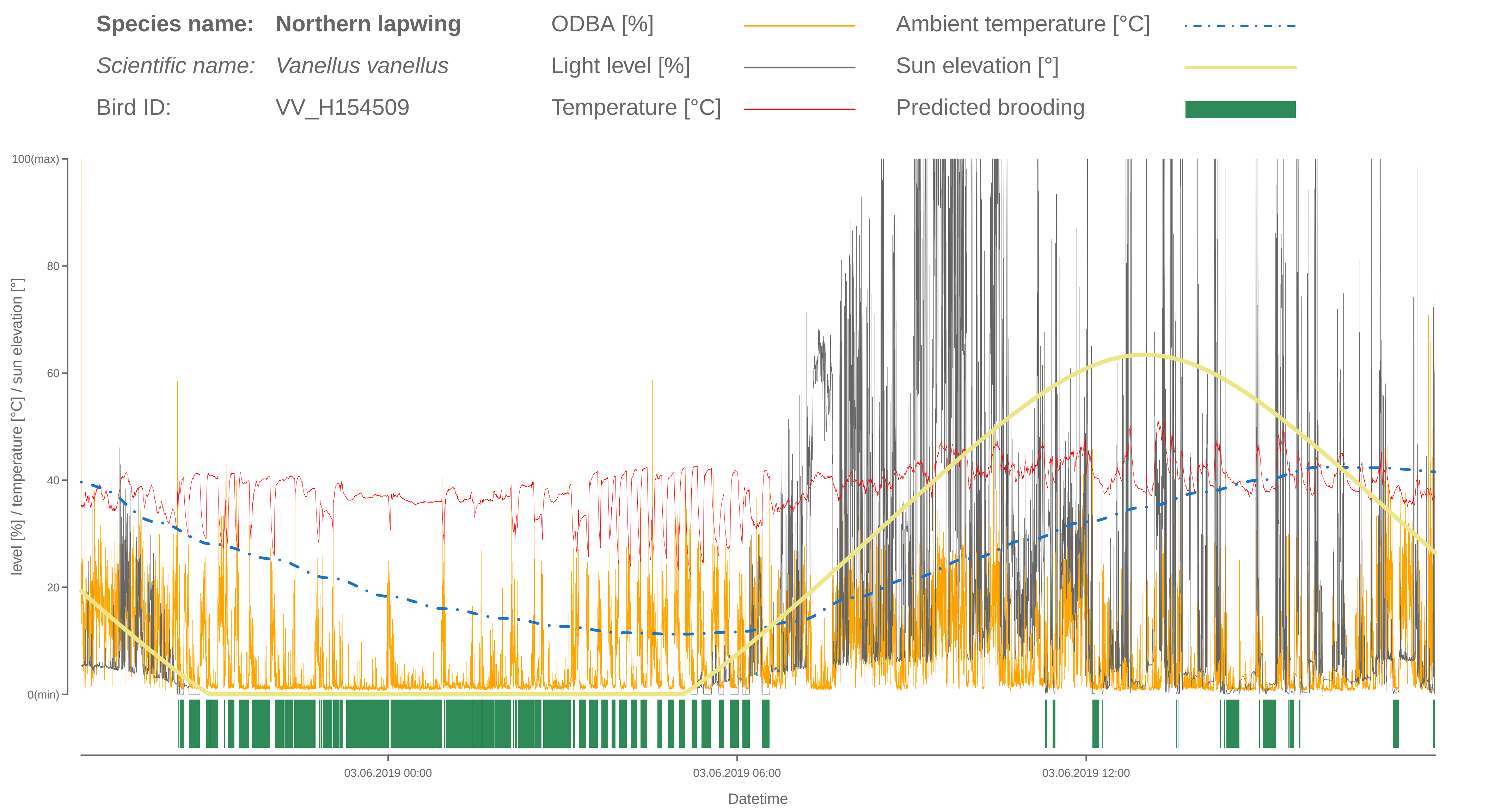
Species name: Northern lapwing
Scientific name: Vanellus vanellus
Bird ID: VV_H154509
ODBA [%]
Light level [%]
Temperature [°C]
Ambient temperature [°C]
Sun elevation [°]
Predicted brooding
100(max)
80
60
40
20
0(min)
level [%] / temperature [°C] / sun elevation [°]
03.06.2019 00:00
03.06.2019 06:00
03.06.2019 12:00
Datetime

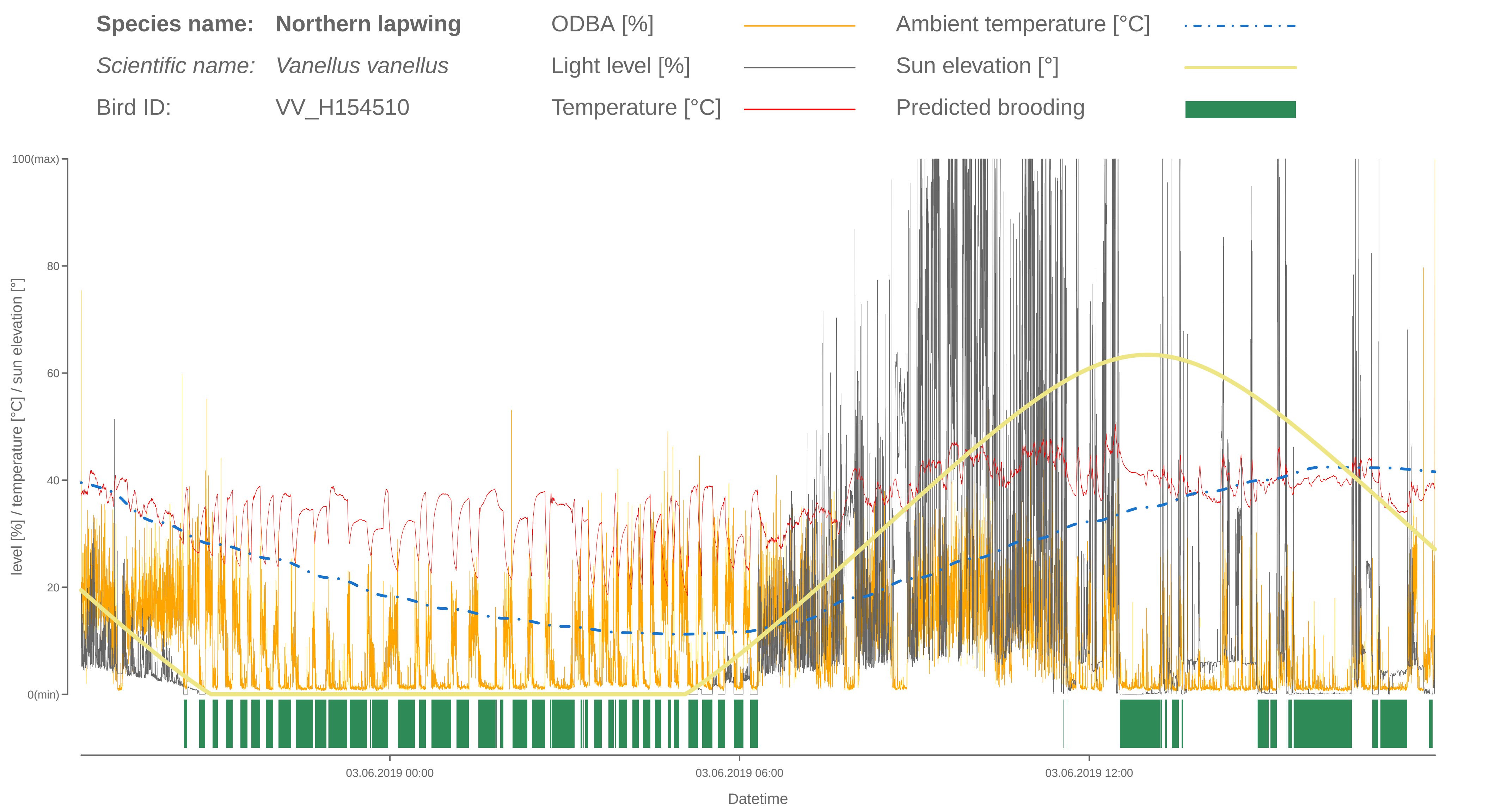
Species name: Northern lapwing
Scientific name: Vanellus vanellus
Bird ID: VV_H154510
ODBA [%]
Light level [%]
Temperature [°C]
Ambient temperature [°C]
Sun elevation [°]
Predicted brooding
level [%] / temperature [°C] / sun elevation [°]
100(max)
80
60
40
20
0(min)
03.06.2019 00:00
03.06.2019 06:00
03.06.2019 12:00
Datetime

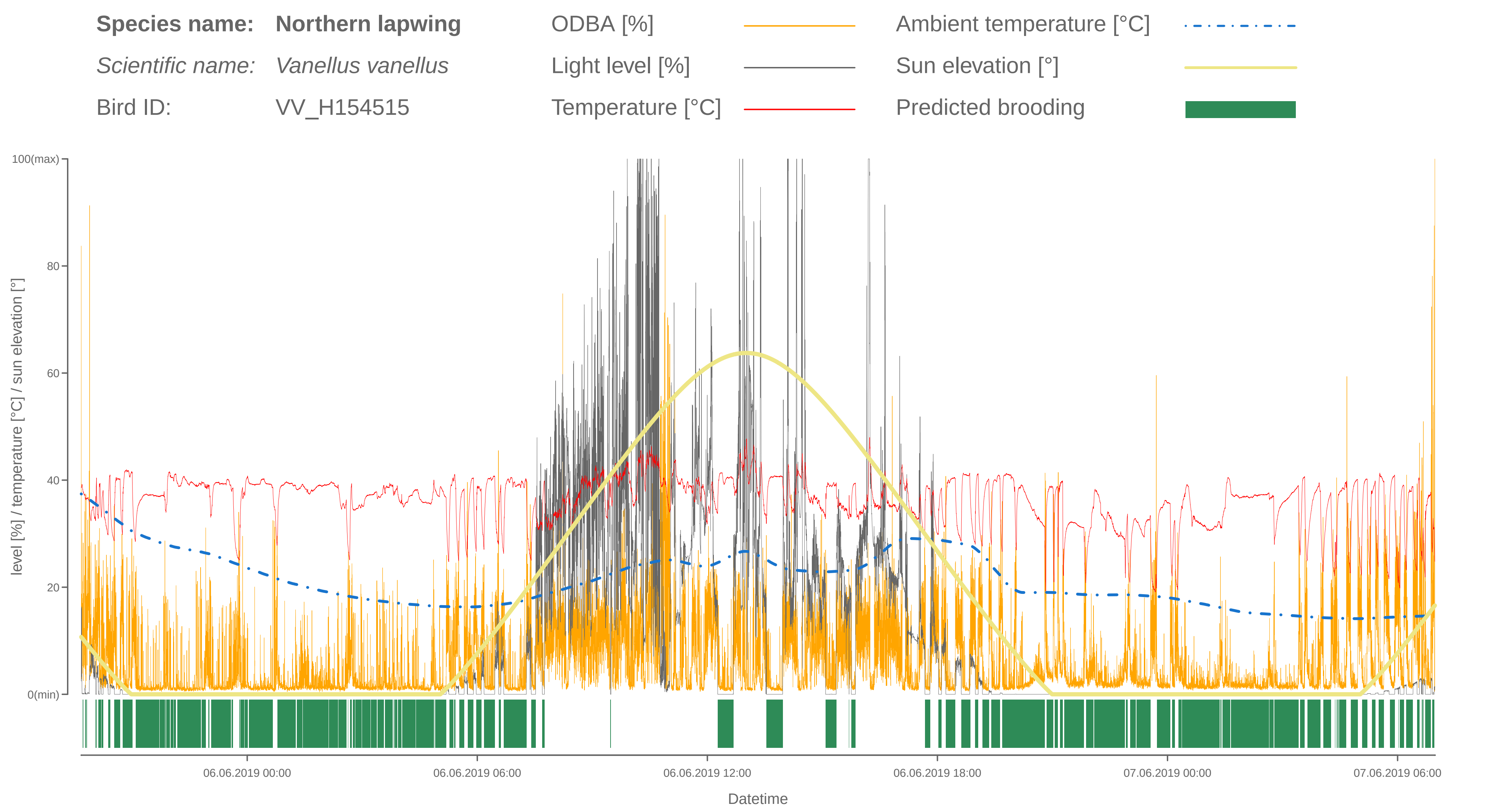
Species name: Northern lapwing
Scientific name: Vanellus vanellus
Bird ID: VV_H154515
ODBA [%]
Light level [%]
Temperature [°C]
Ambient temperature [°C]
Sun elevation [°]
Predicted brooding
100(max)
80
60
40
20
0(min)
level [%] / temperature [°C] / sun elevation [°]
06.06.2019 00:00
06.06.2019 06:00
06.06.2019 12:00
06.06.2019 18:00
07.06.2019 00:00
07.06.2019 06:00
Datetime

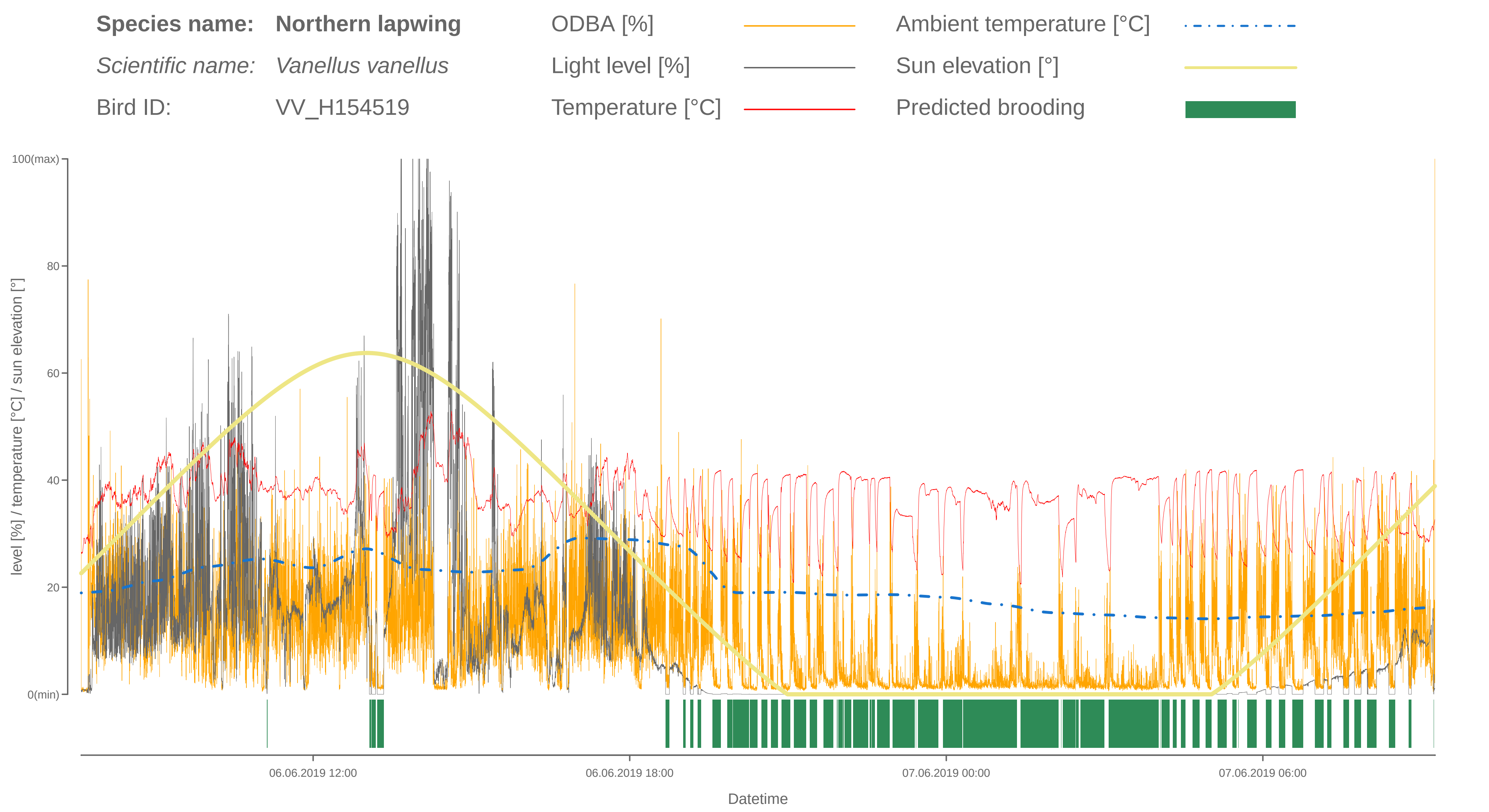
Species name: Northern lapwing
Scientific name: Vanellus vanellus
Bird ID: VV_H154519
ODBA [%]
Light level [%]
Temperature [°C]
Ambient temperature [°C]
Sun elevation [°]
Predicted brooding
100(max)
80
60
40
20
0(min)
level [%] / temperature [°C] / sun elevation [°]
06.06.2019 12:00
06.06.2019 18:00
07.06.2019 00:00
07.06.2019 06:00
Datetime

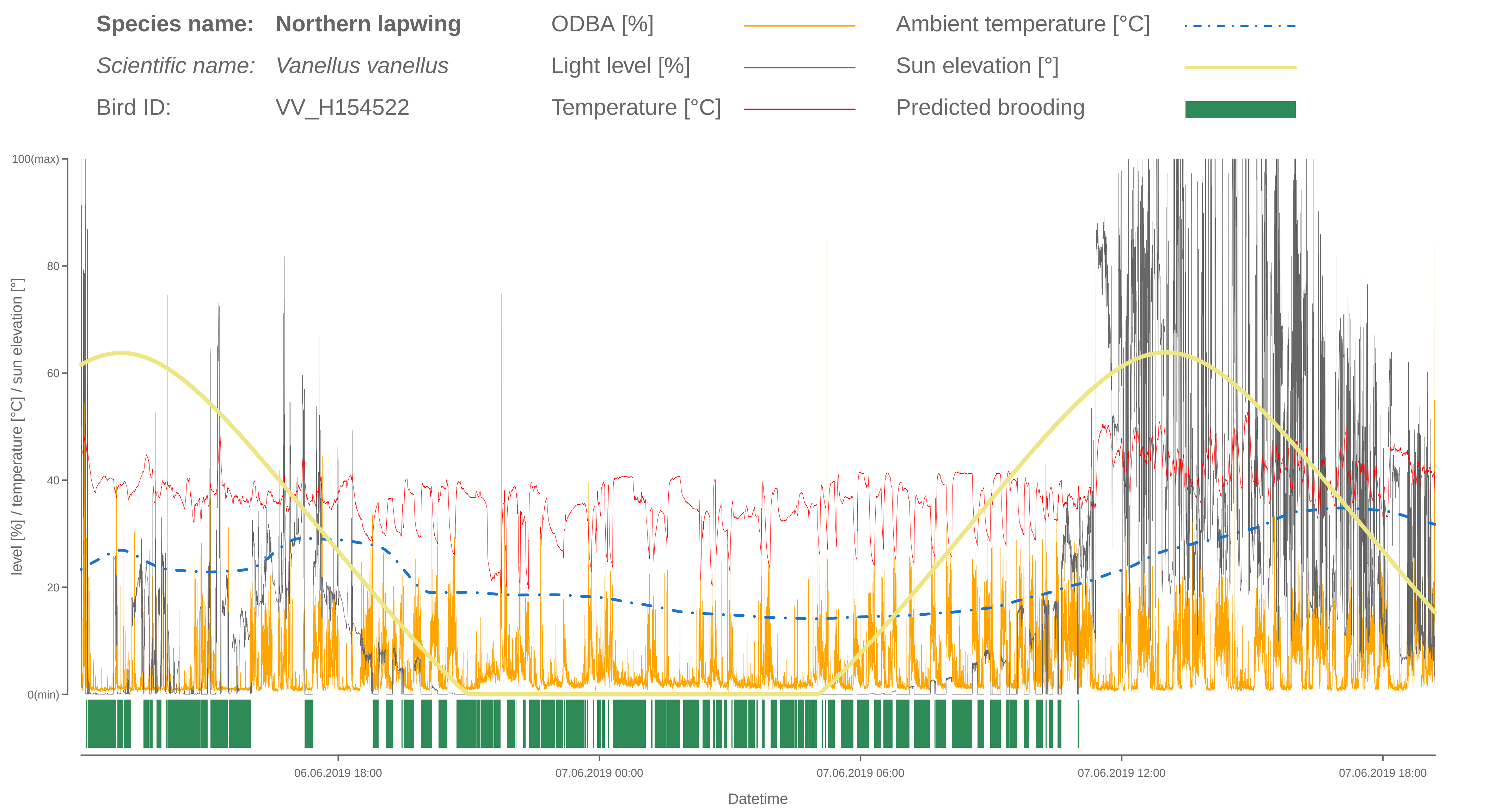

Species name: Northern lapwing
Scientific name: Vanellus vanellus
Bird ID: VV_H154522
ODBA [%]
Light level [%]
Temperature [°C]
Ambient temperature [°C]
Sun elevation [°]
Predicted brooding
100(max)
80
60
40
20
0(min)
level [%] / temperature [°C] / sun elevation [°]
06.06.2019 18:00
07.06.2019 00:00
07.06.2019 06:00
07.06.2019 12:00
07.06.2019 18:00
Datetime

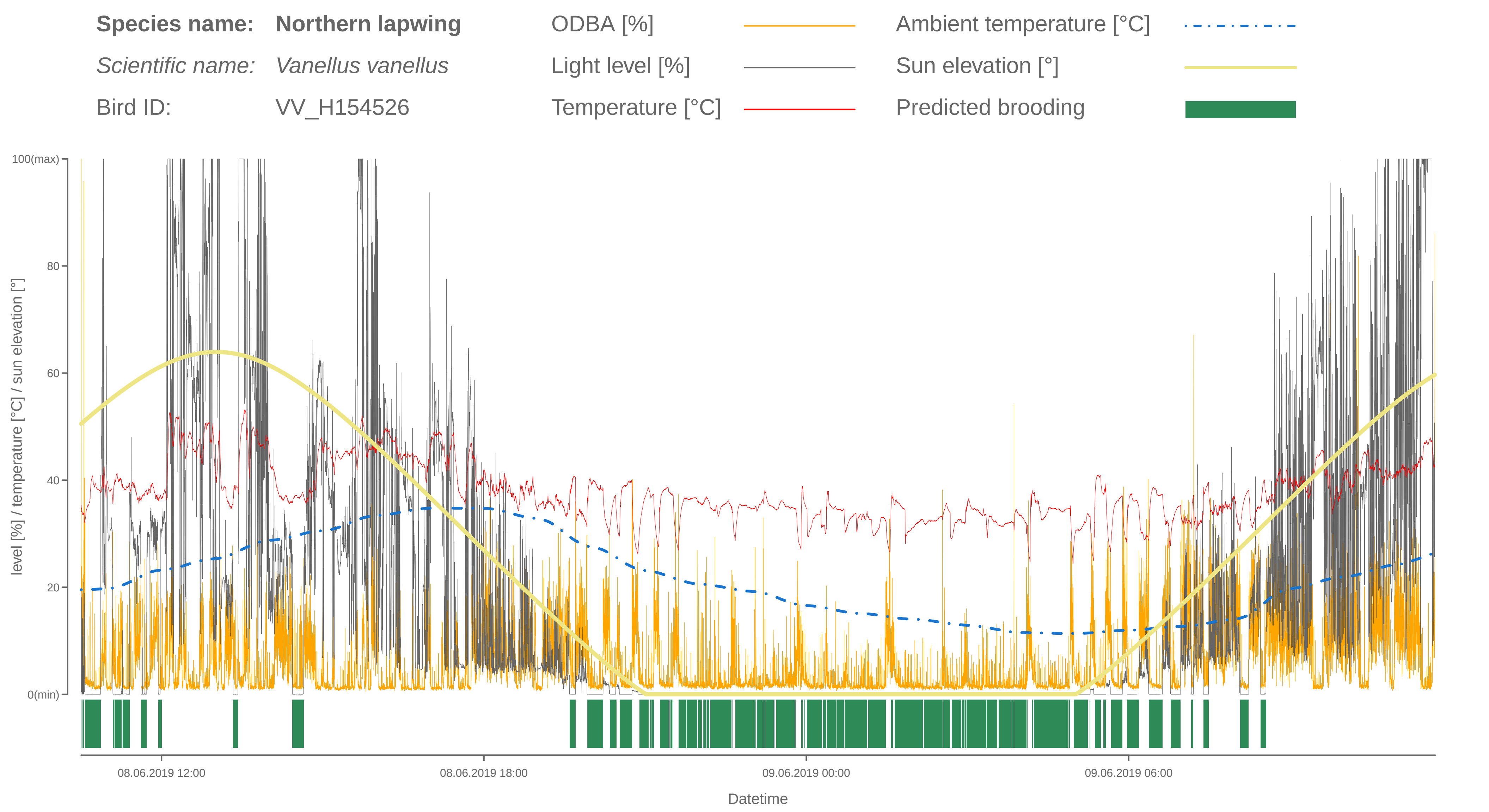
Species name: Northern lapwing
Scientific name: Vanellus vanellus
Bird ID: VV_H154526
ODBA [%]
Light level [%]
Temperature [°C]
Ambient temperature [°C]
Sun elevation [°]
Predicted brooding
100(max)
80
60
40
20
0(min)
level [%] / temperature [°C] / sun elevation [°]
08.06.2019 12:00
08.06.2019 18:00
09.06.2019 00:00
09.06.2019 06:00
Datetime

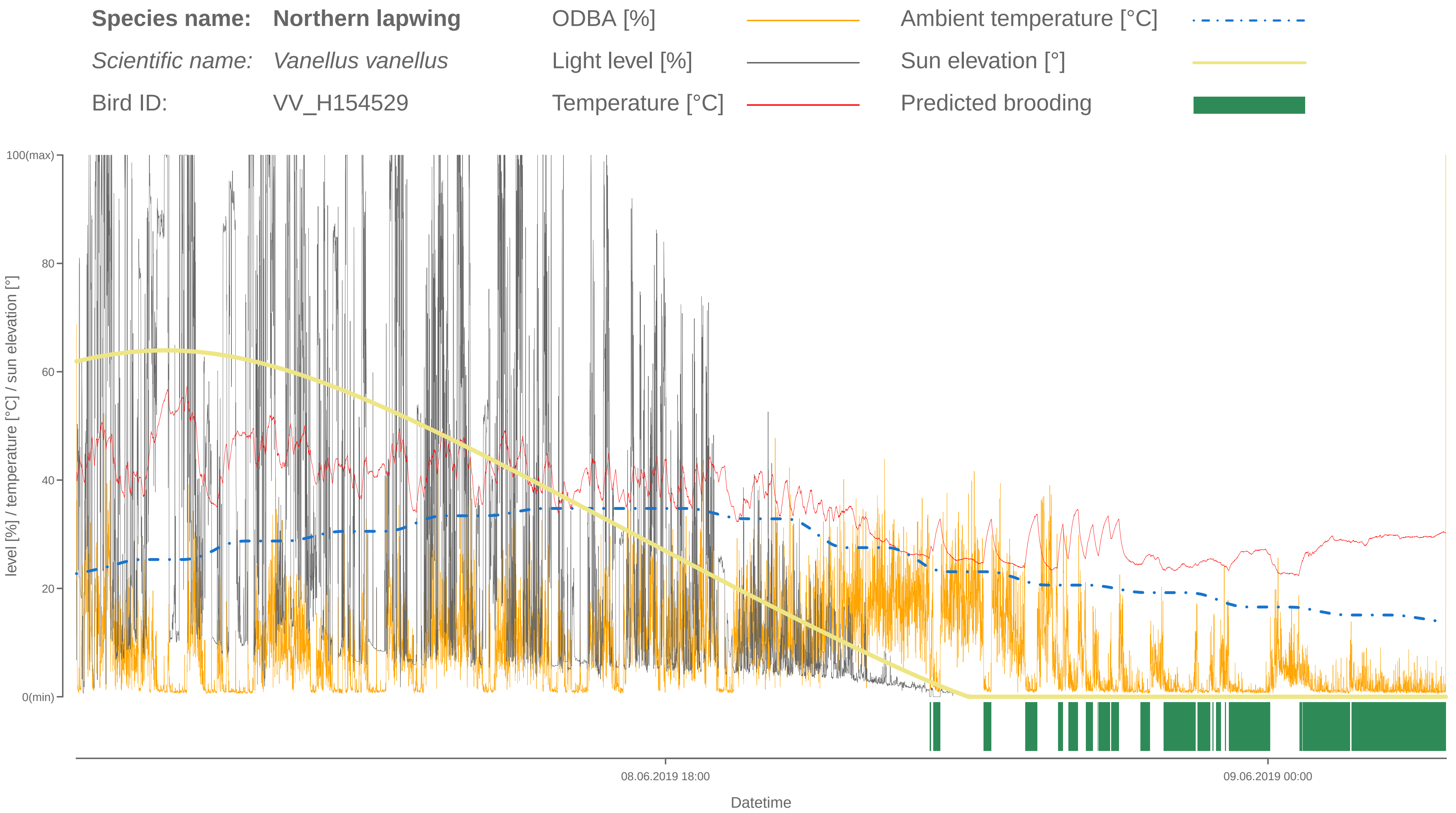

Species name: Northern lapwing
Scientific name: Vanellus vanellus
Bird ID: VV_H154529
ODBA [%]
Light level [%]
Temperature [°C]
Ambient temperature [°C]
Sun elevation [°]
Predicted brooding
100(max)
80
60
40
20
0(min)
level [%] / temperature [°C] / sun elevation [°]
08.06.2019 18:00
09.06.2019 00:00
Datetime

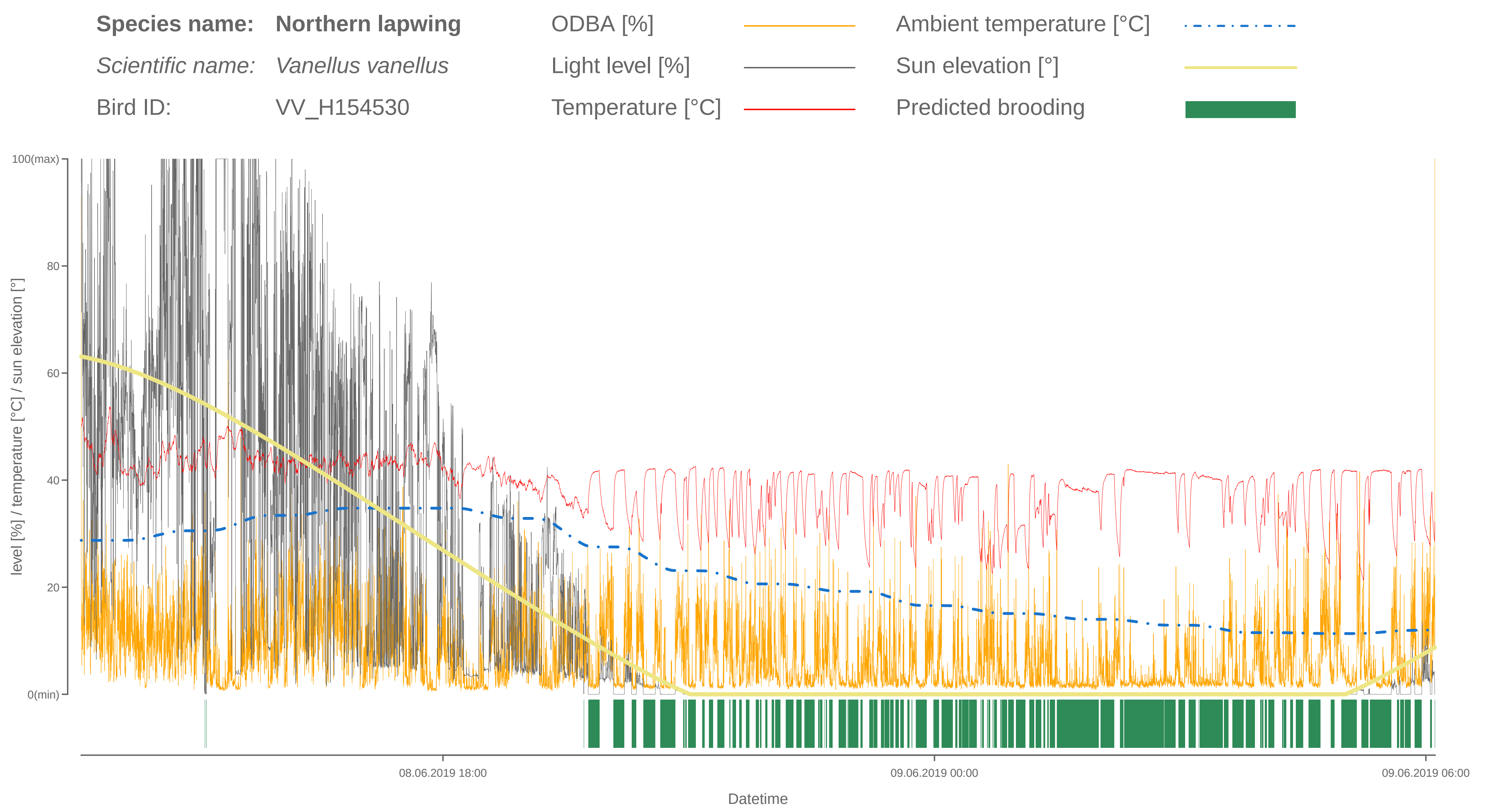

Species name: Northern lapwing
Scientific name: Vanellus vanellus
Bird ID: VV_H154530
ODBA [%]
Light level [%]
Temperature [°C]
Ambient temperature [°C]
Sun elevation [°]
Predicted brooding
100(max)
80
60
40
20
0(min)
level [%] / temperature [°C] / sun elevation [°]
08.06.2019 18:00
09.06.2019 00:00
09.06.2019 06:00
Datetime

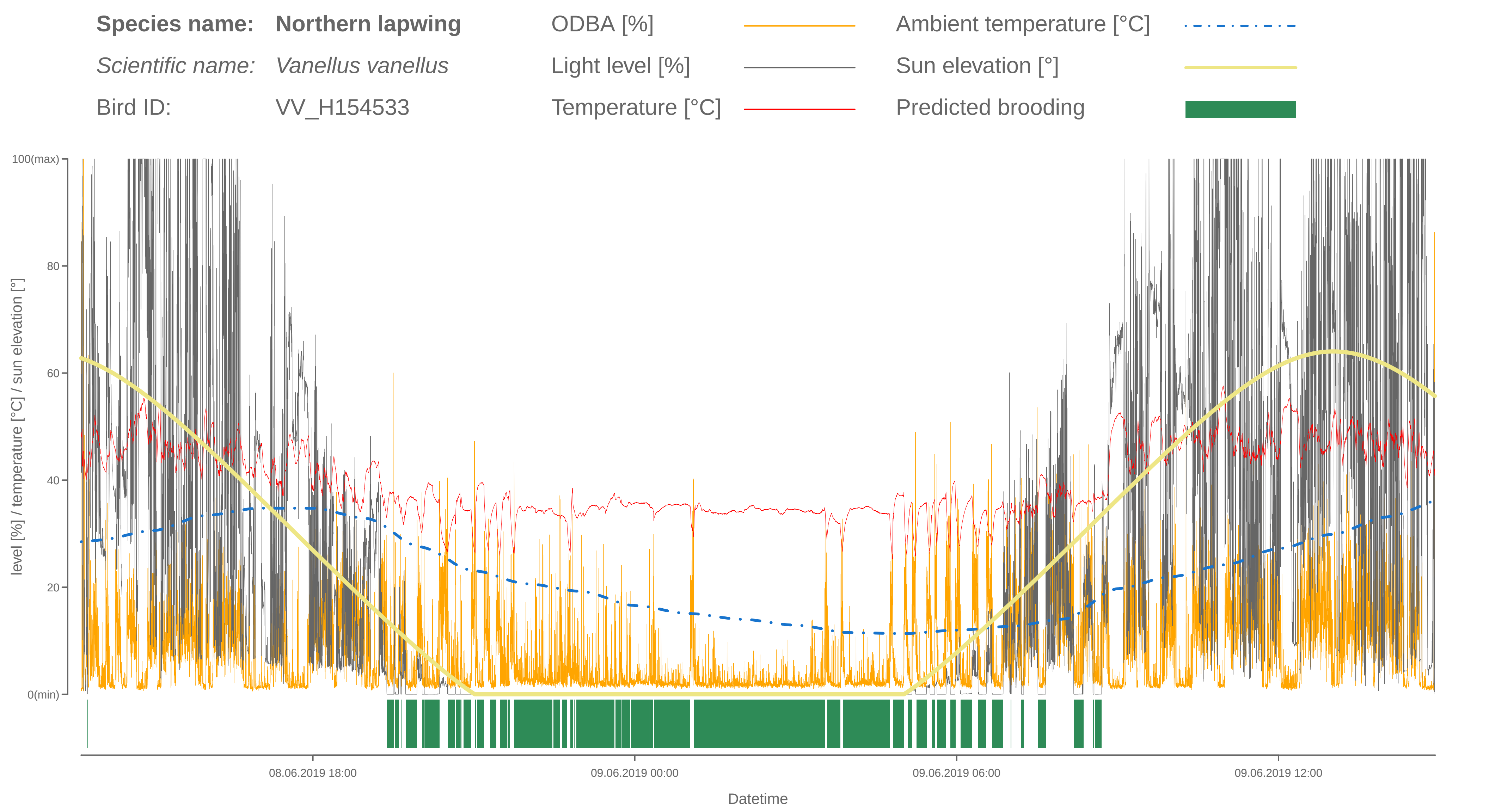
Species name: Northern lapwing
Scientific name: Vanellus vanellus
Bird ID: VV_H154533
ODBA [%]
Light level [%]
Temperature [°C]
Ambient temperature [°C]
Sun elevation [°]
Predicted brooding
100(max)
80
60
40
20
0(min)
level [%] / temperature [°C] / sun elevation [°]
08.06.2019 18:00
09.06.2019 00:00
09.06.2019 06:00
09.06.2019 12:00
Datetime

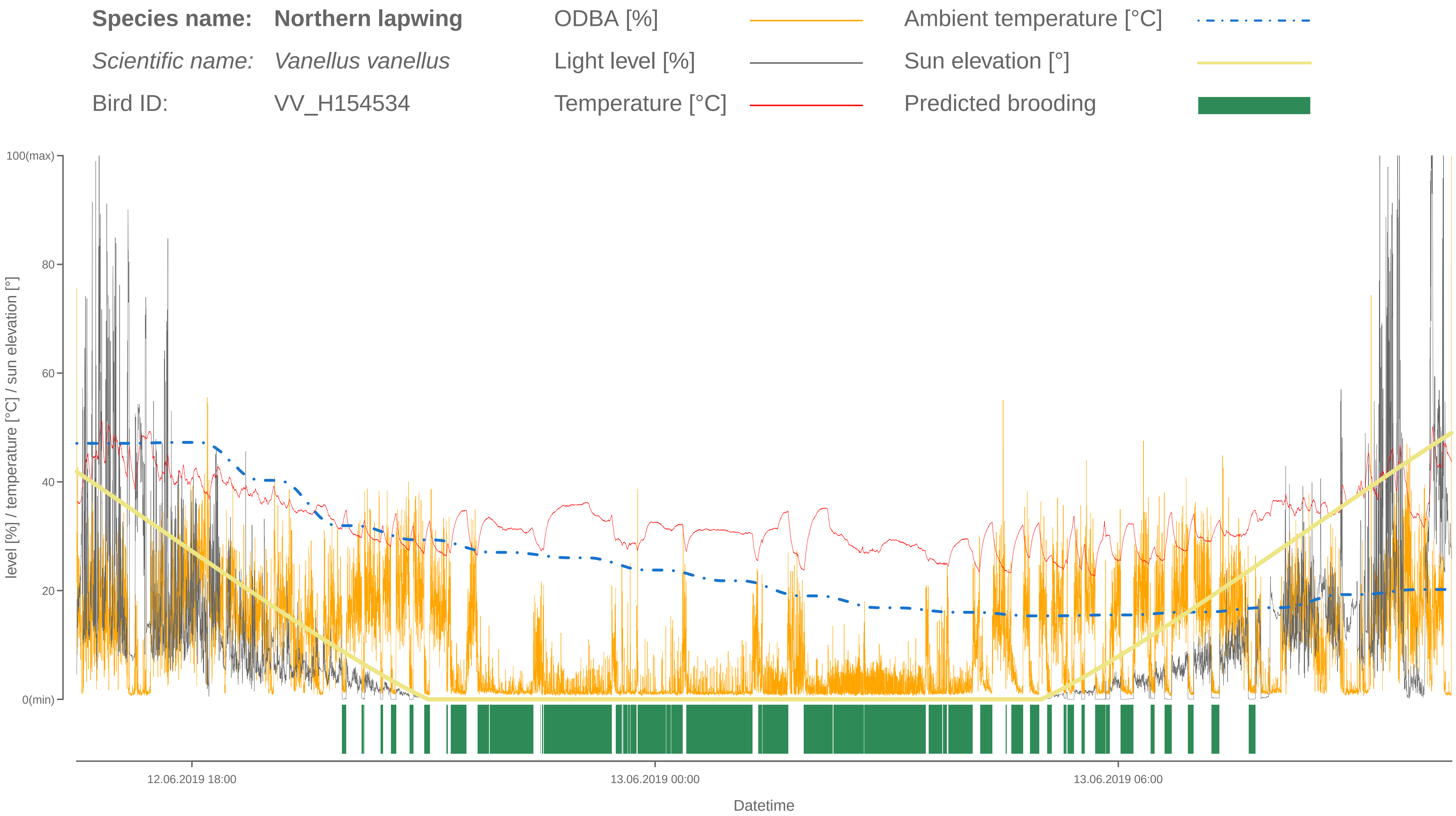
Species name: Northern lapwing
Scientific name: Vanellus vanellus
Bird ID: VV_H154534
ODBA [%]
Light level [%]
Temperature [°C]
Ambient temperature [°C]
Sun elevation [°]
Predicted brooding
100(max)
80
60
40
20
0(min)
level [%] / temperature [°C] / sun elevation [°]
12.06.2019 18:00
13.06.2019 00:00
13.06.2019 06:00
Datetime

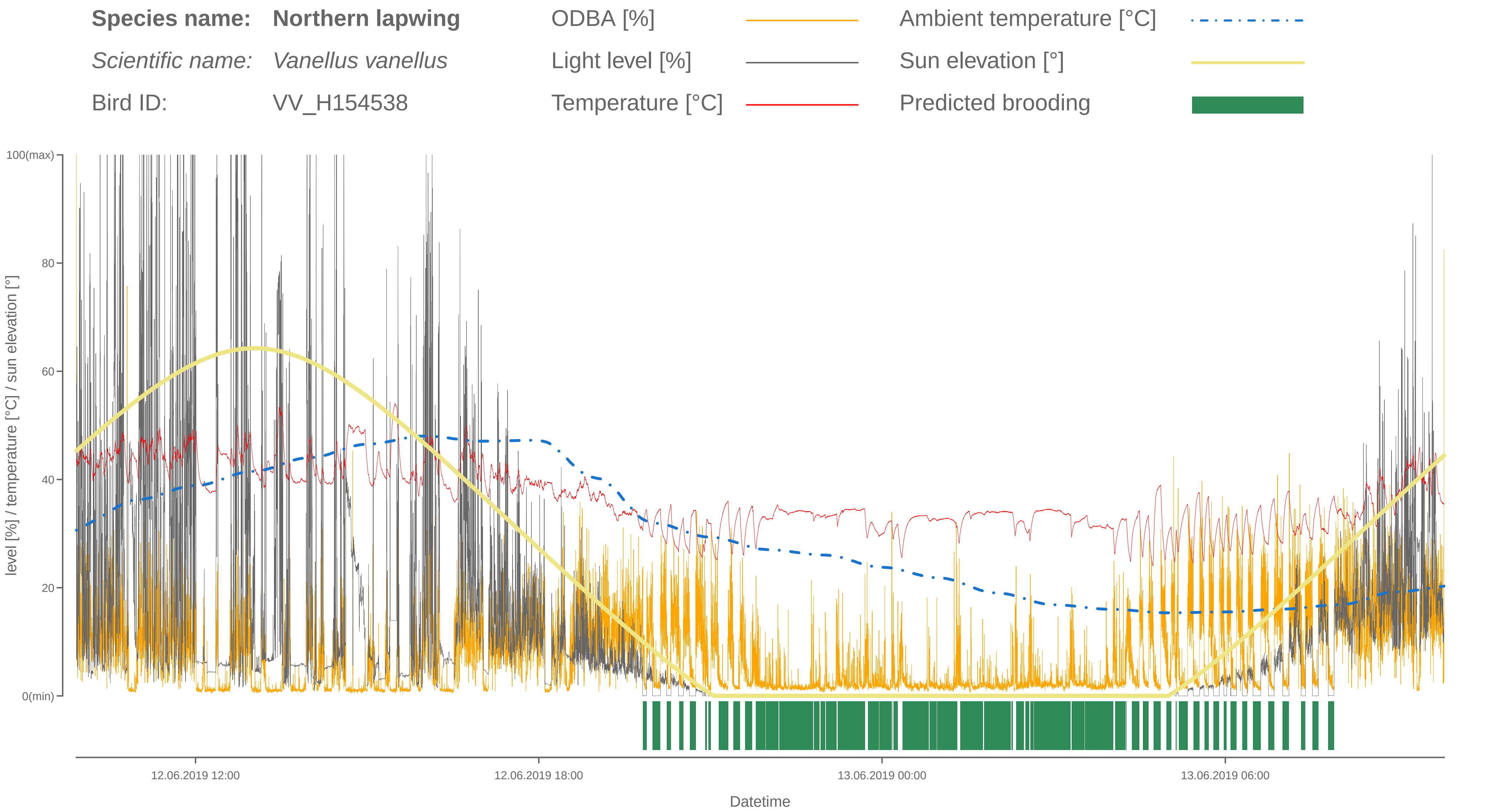

Species name: Northern lapwing
Scientific name: Vanellus vanellus
Bird ID: VV_H154538
ODBA [%]
Light level [%]
Temperature [°C]
Ambient temperature [°C]
Sun elevation [°]
Predicted brooding
100(max)
80
60
40
20
0(min)
level [%] / temperature [°C] / sun elevation [°]
12.06.2019 12:00
12.06.2019 18:00
13.06.2019 00:00
13.06.2019 06:00
Datetime

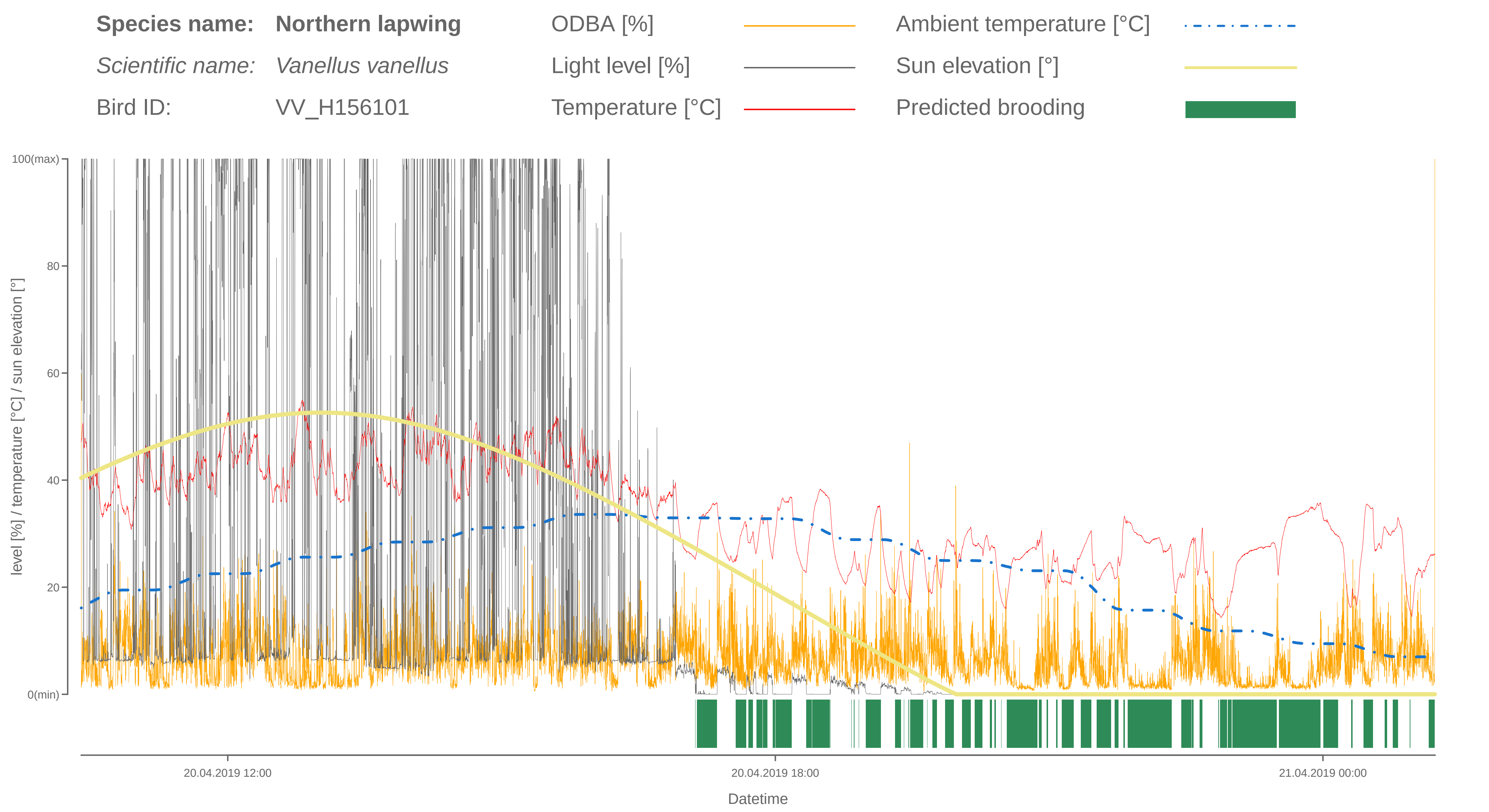
Species name: Northern lapwing
Scientific name: Vanellus vanellus
Bird ID: VV_H156101
ODBA [%]
Light level [%]
Temperature [°C]
Ambient temperature [°C]
Sun elevation [°]
Predicted brooding
100(max)
80
60
40
20
0(min)
level [%] / temperature [°C] / sun elevation [°]
20.04.2019 12:00
20.04.2019 18:00
21.04.2019 00:00
Datetime

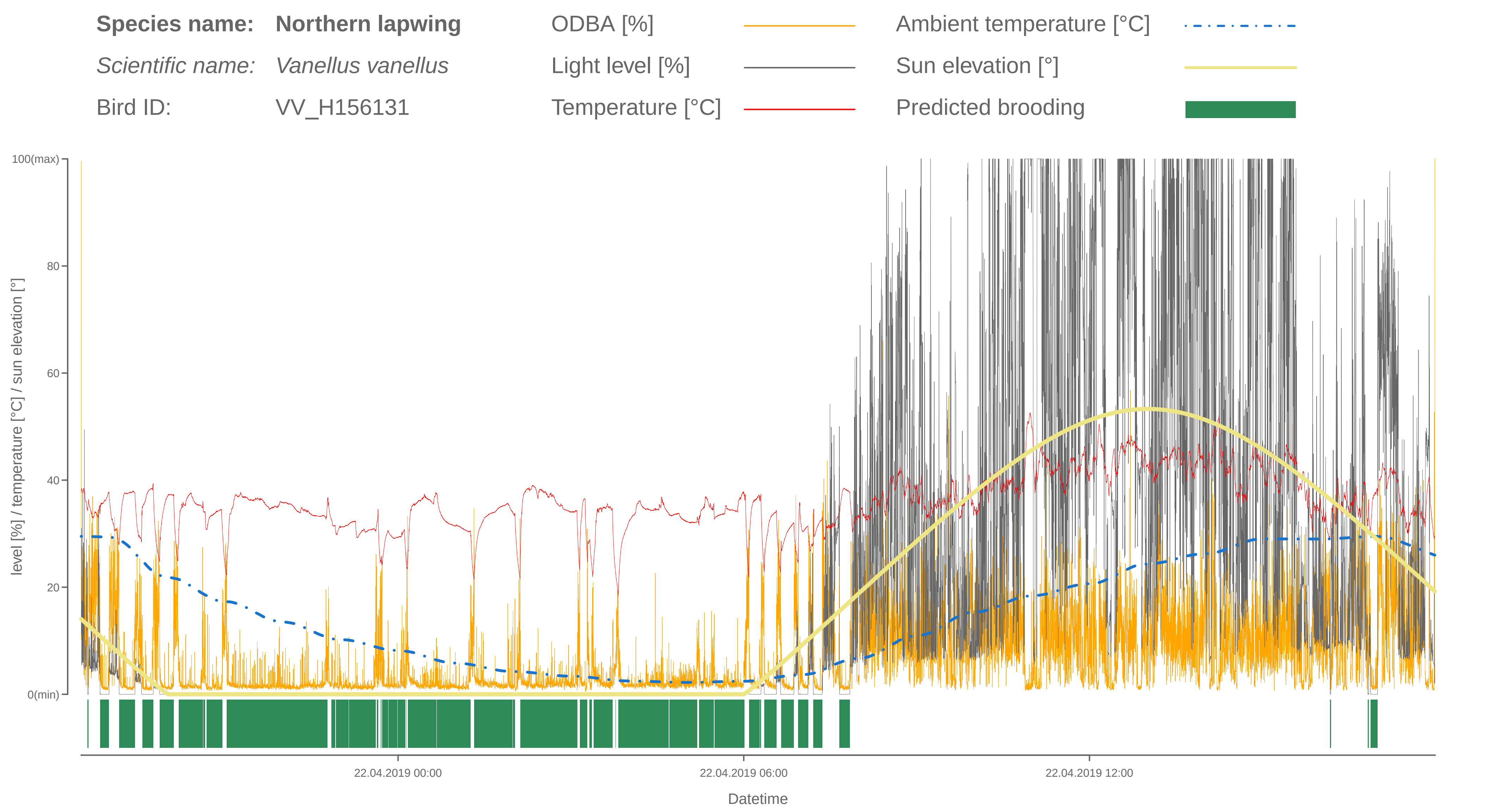

Species name: Northern lapwing
Scientific name: Vanellus vanellus
Bird ID: VV_H156131
ODBA [%]
Light level [%]
Temperature [°C]
Ambient temperature [°C]
Sun elevation [°]
Predicted brooding
100(max)
80
60
40
20
0(min)
level [%] / temperature [°C] / sun elevation [°]
22.04.2019 00:00
22.04.2019 06:00
22.04.2019 12:00
Datetime

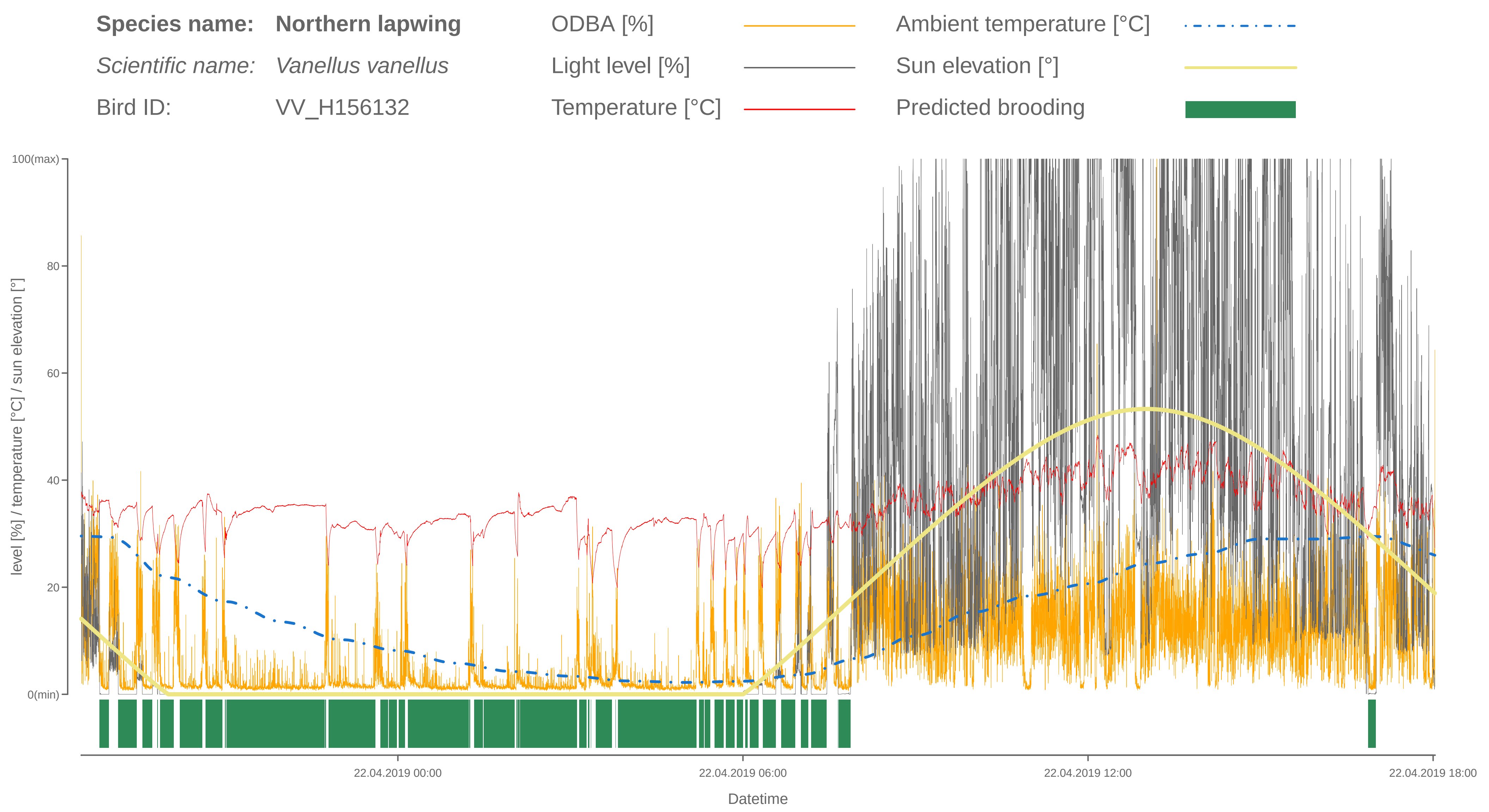

Species name: Northern lapwing
Scientific name: Vanellus vanellus
Bird ID: VV_H156132
ODBA [%]
Light level [%]
Temperature [°C]
Ambient temperature [°C]
Sun elevation [°]
Predicted brooding
100(max)
80
60
40
20
0(min)
level [%] / temperature [°C] / sun elevation [°]
22.04.2019 00:00
22.04.2019 06:00
22.04.2019 12:00
22.04.2019 18:00
Datetime

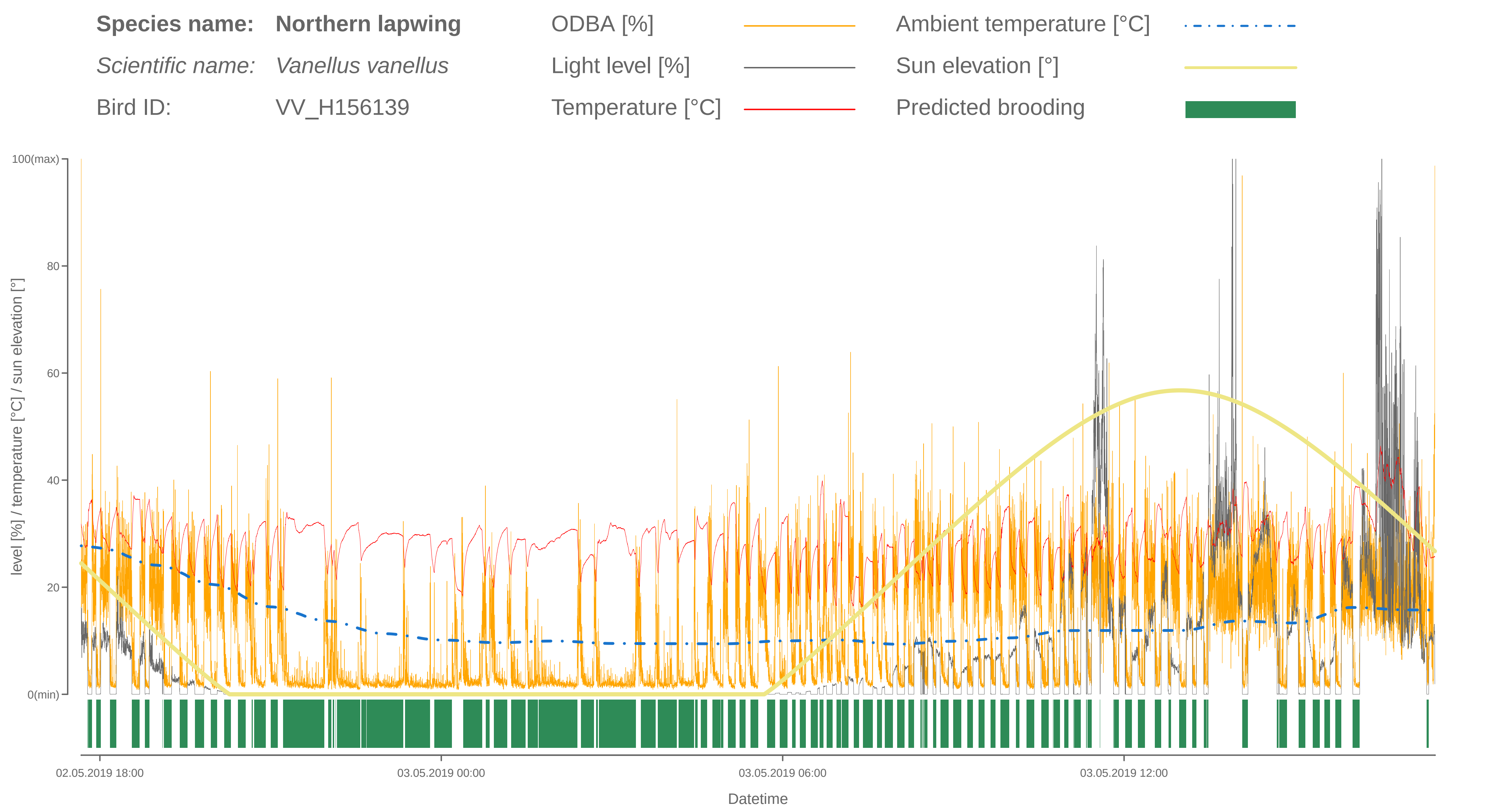
Species name: Northern lapwing
Scientific name: Vanellus vanellus
Bird ID: VV_H156139
ODBA [%]
Light level [%]
Temperature [°C]
Ambient temperature [°C]
Sun elevation [°]
Predicted brooding
level [%] / temperature [°C] / sun elevation [°]
100(max)
80
60
40
20
0(min)
02.05.2019 18:00
03.05.2019 00:00
03.05.2019 06:00
03.05.2019 12:00
Datetime

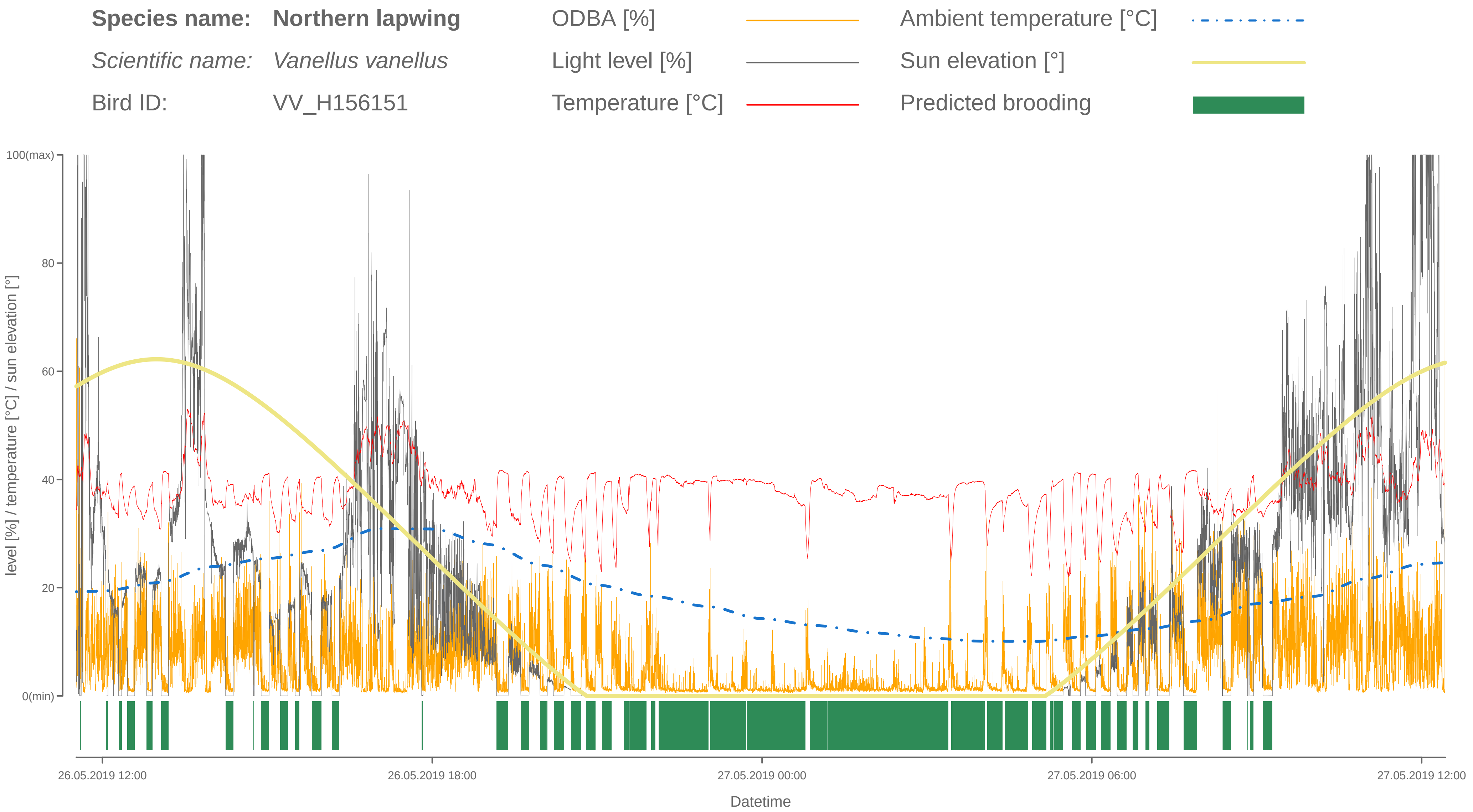
Species name: Northern lapwing
Scientific name: Vanellus vanellus
Bird ID: VV_H156151
ODBA [%]
Light level [%]
Temperature [°C]
Ambient temperature [°C]
Sun elevation [°]
Predicted brooding
100(max)
80
60
40
20
0(min)
level [%] / temperature [°C] / sun elevation [°]
26.05.2019 12:00
26.05.2019 18:00
27.05.2019 00:00
27.05.2019 06:00
27.05.2019 12:00
Datetime

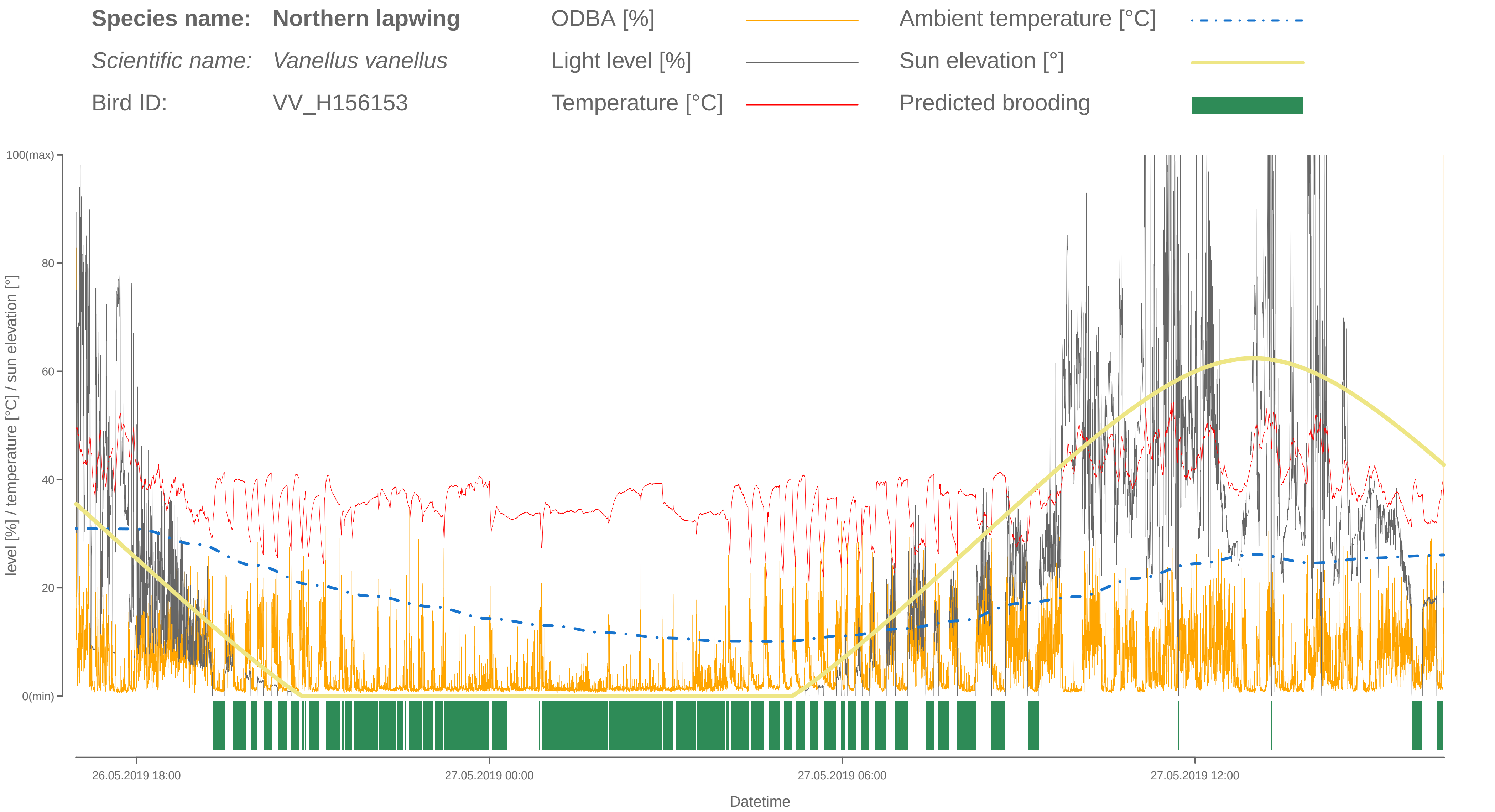

Species name: Northern lapwing
Scientific name: Vanellus vanellus
Bird ID: VV_H156153
ODBA [%]
Light level [%]
Temperature [°C]
Ambient temperature [°C]
Sun elevation [°]
Predicted brooding
100(max)
80
60
40
20
0(min)
level [%] / temperature [°C] / sun elevation [°]
26.05.2019 18:00
27.05.2019 00:00
27.05.2019 06:00
27.05.2019 12:00
Datetime

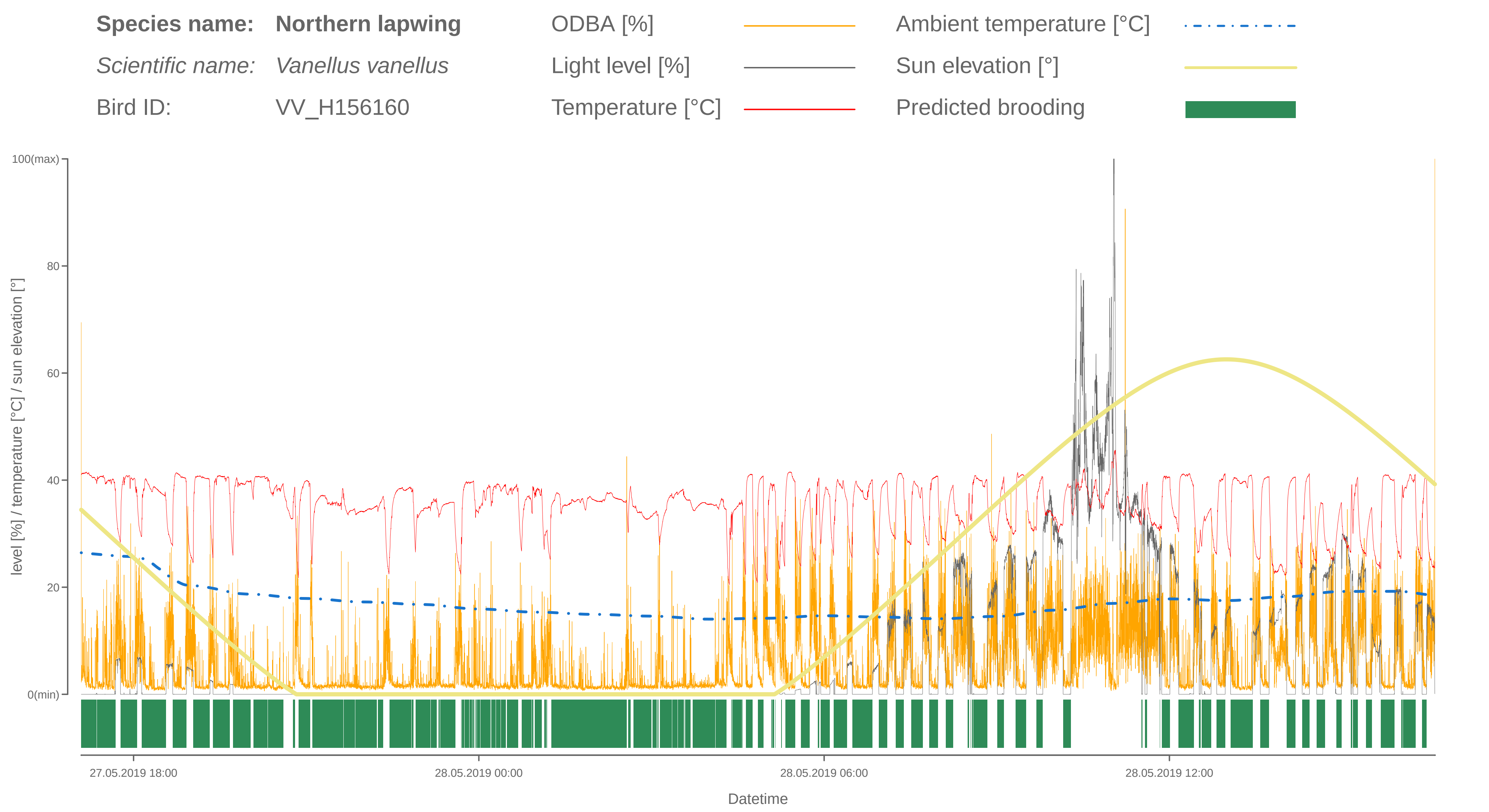
Species name: Northern lapwing
Scientific name: Vanellus vanellus
Bird ID: VV_H156160
ODBA [%]
Light level [%]
Temperature [°C]
Ambient temperature [°C]
Sun elevation [°]
Predicted brooding
100(max)
80
60
40
20
0(min)
level [%] / temperature [°C] / sun elevation [°]
27.05.2019 18:00
28.05.2019 00:00
28.05.2019 06:00
28.05.2019 12:00
Datetime

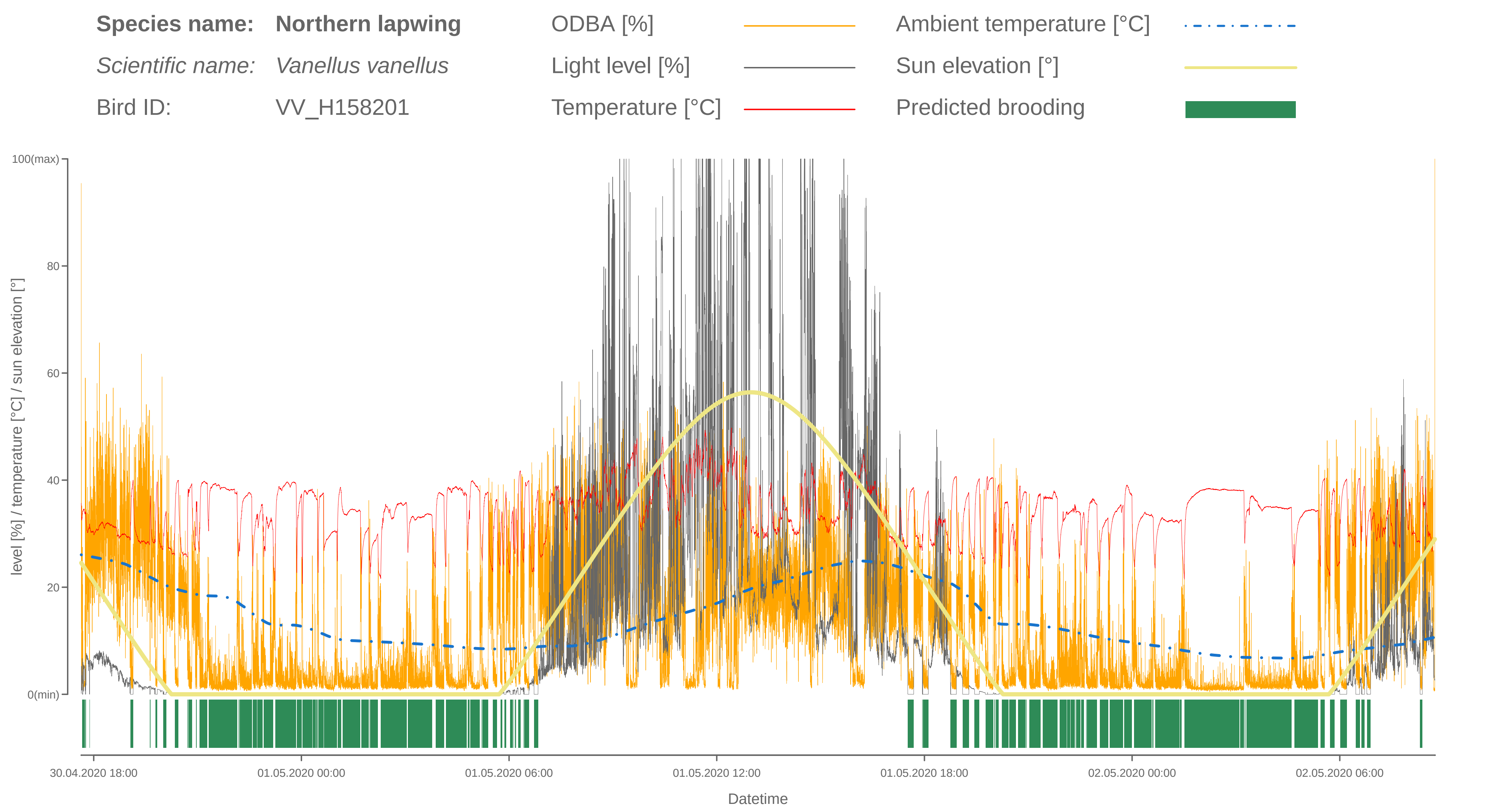
Species name: Northern lapwing
Scientific name: Vanellus vanellus
Bird ID: VV_H158201
ODBA [%]
Light level [%]
Temperature [°C]
Ambient temperature [°C]
Sun elevation [°]
Predicted brooding
100(max)
80
60
40
20
0(min)
level [%] / temperature [°C] / sun elevation [°]
30.04.2020 18:00
01.05.2020 00:00
01.05.2020 06:00
01.05.2020 12:00
01.05.2020 18:00
02.05.2020 00:00
02.05.2020 06:00
Datetime

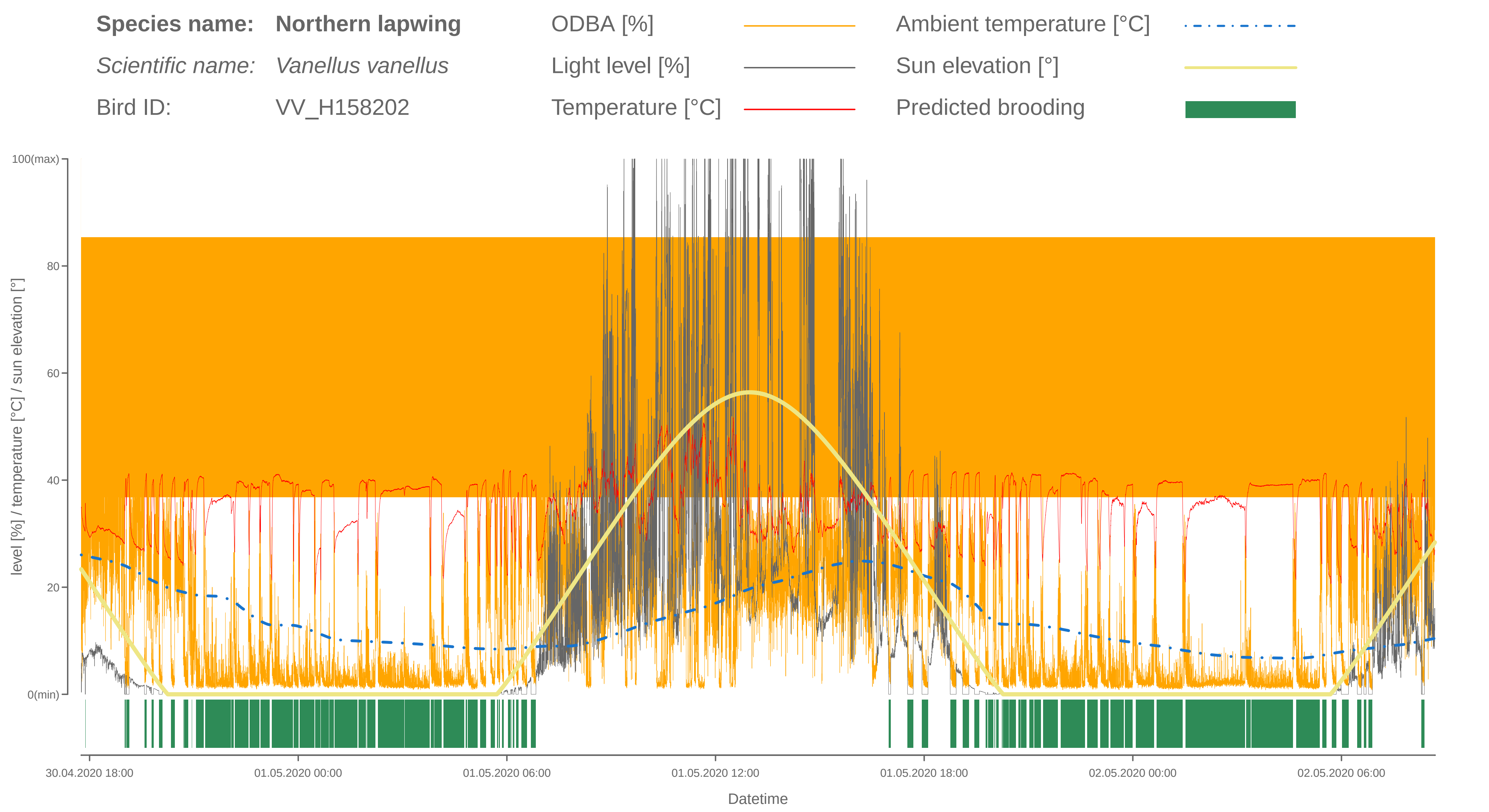

Species name: Northern lapwing
Scientific name: Vanellus vanellus
Bird ID: VV_H158202
ODBA [%]
Light level [%]
Temperature [°C]
Ambient temperature [°C]
Sun elevation [°]
Predicted brooding
100(max)
80
60
40
20
0(min)
level [%] / temperature [°C] / sun elevation [°]
30.04.2020 18:00
01.05.2020 00:00
01.05.2020 06:00
01.05.2020 12:00
01.05.2020 18:00
02.05.2020 00:00
02.05.2020 06:00
Datetime

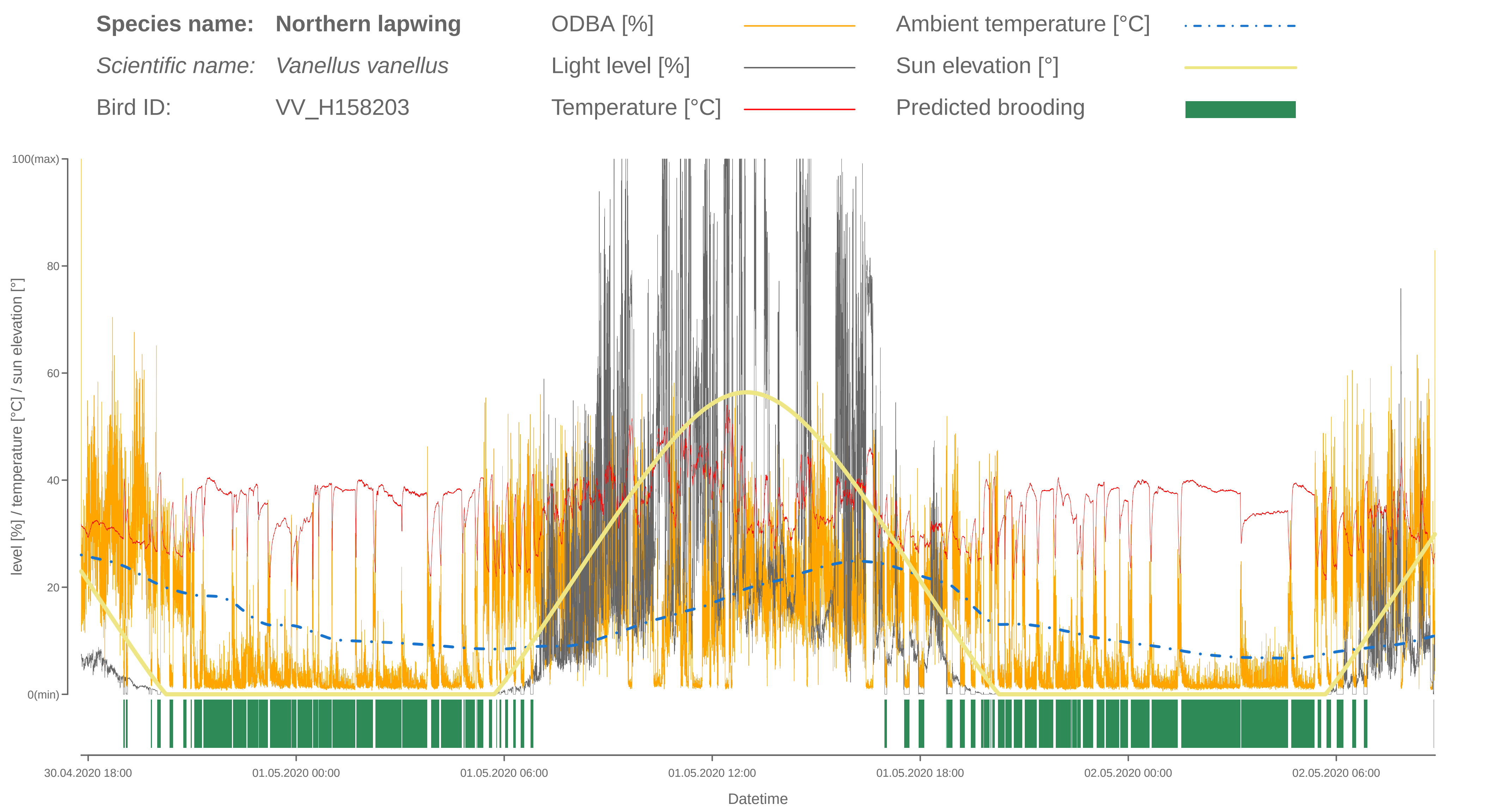
Species name: Northern lapwing
Scientific name: Vanellus vanellus
Bird ID: VV_H158203
ODBA [%]
Light level [%]
Temperature [°C]
Ambient temperature [°C]
Sun elevation [°]
Predicted brooding
100(max)
80
60
40
20
0(min)
level [%] / temperature [°C] / sun elevation [°]
30.04.2020 18:00
01.05.2020 00:00
01.05.2020 06:00
01.05.2020 12:00
01.05.2020 18:00
02.05.2020 00:00
02.05.2020 06:00
Datetime

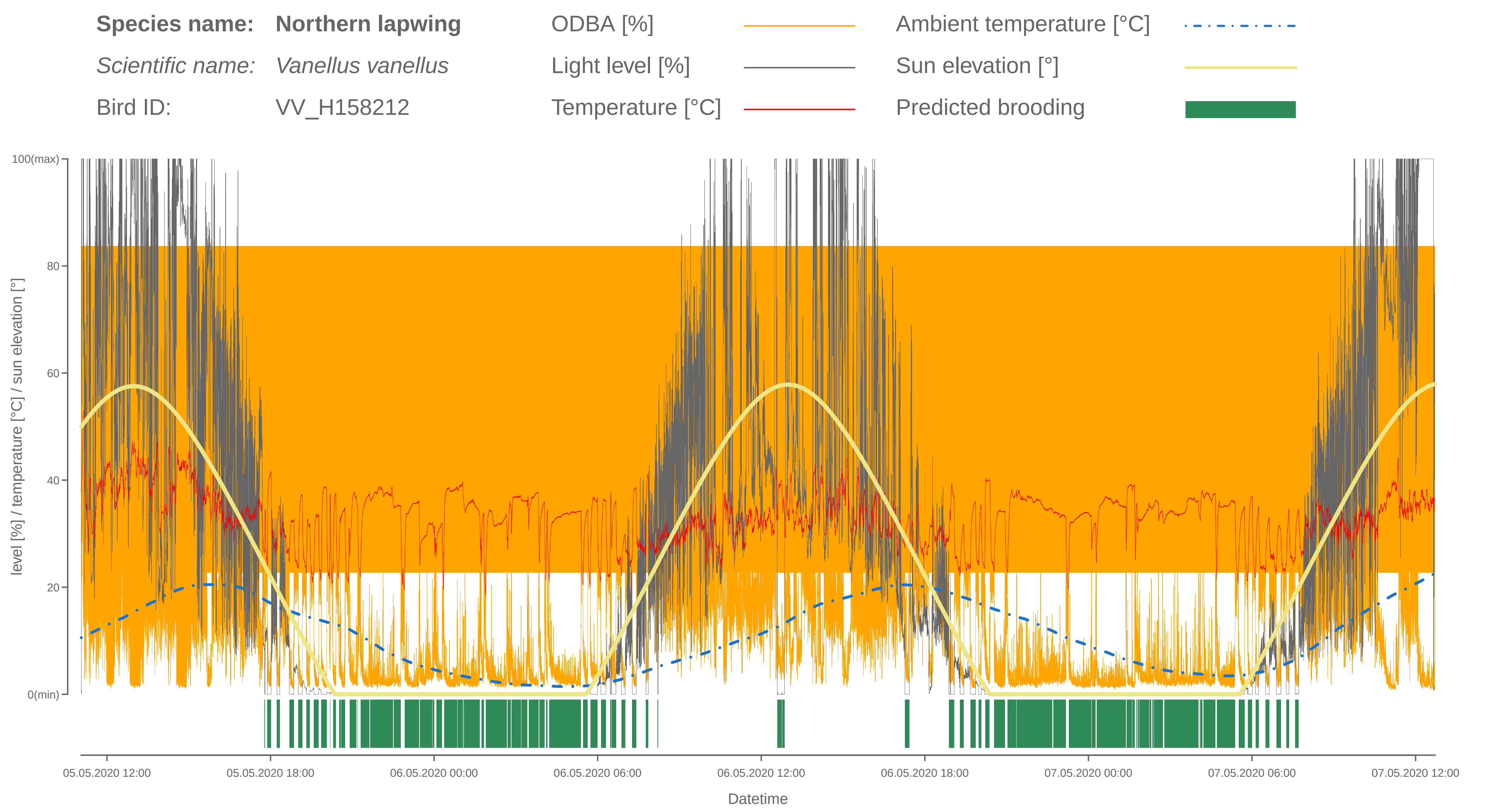

Species name: Northern lapwing
Scientific name: Vanellus vanellus
Bird ID: VV_H158212
ODBA [%]
Light level [%]
Temperature [°C]
Ambient temperature [°C]
Sun elevation [°]
Predicted brooding
100(max)
80
60
40
20
0(min)
level [%] / temperature [°C] / sun elevation [°]
05.05.2020 12:00
05.05.2020 18:00
06.05.2020 00:00
06.05.2020 06:00
06.05.2020 12:00
06.05.2020 18:00
07.05.2020 00:00
07.05.2020 06:00
07.05.2020 12:00
Datetime

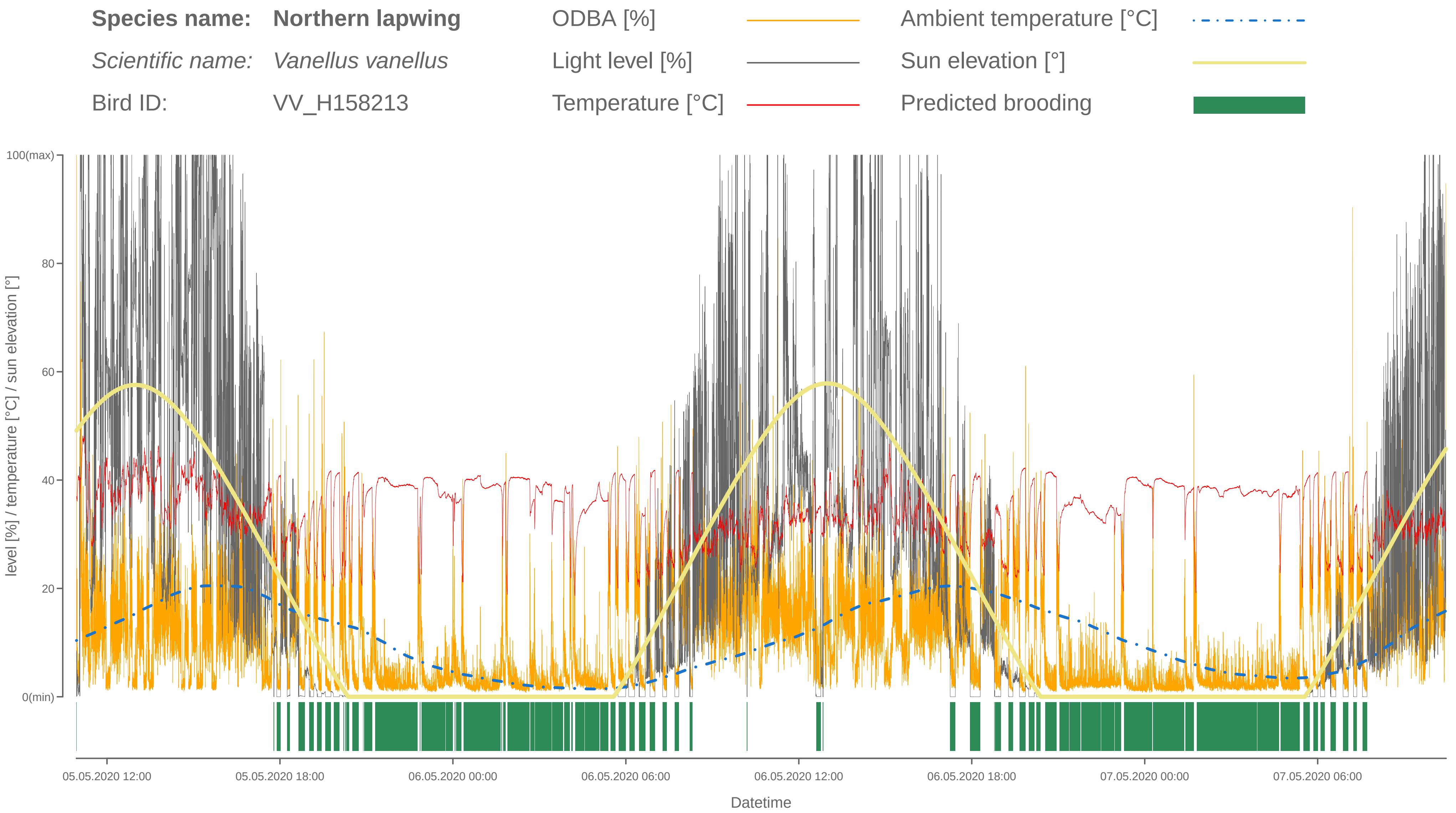
Species name: Northern lapwing
Scientific name: Vanellus vanellus
Bird ID: VV_H158213
ODBA [%]
Light level [%]
Temperature [°C]
Ambient temperature [°C]
Sun elevation [°]
Predicted brooding
100(max)
80
60
40
20
0(min)
level [%] / temperature [°C] / sun elevation [°]
05.05.2020 12:00
05.05.2020 18:00
06.05.2020 00:00
06.05.2020 06:00
06.05.2020 12:00
06.05.2020 18:00
07.05.2020 00:00
07.05.2020 06:00
Datetime

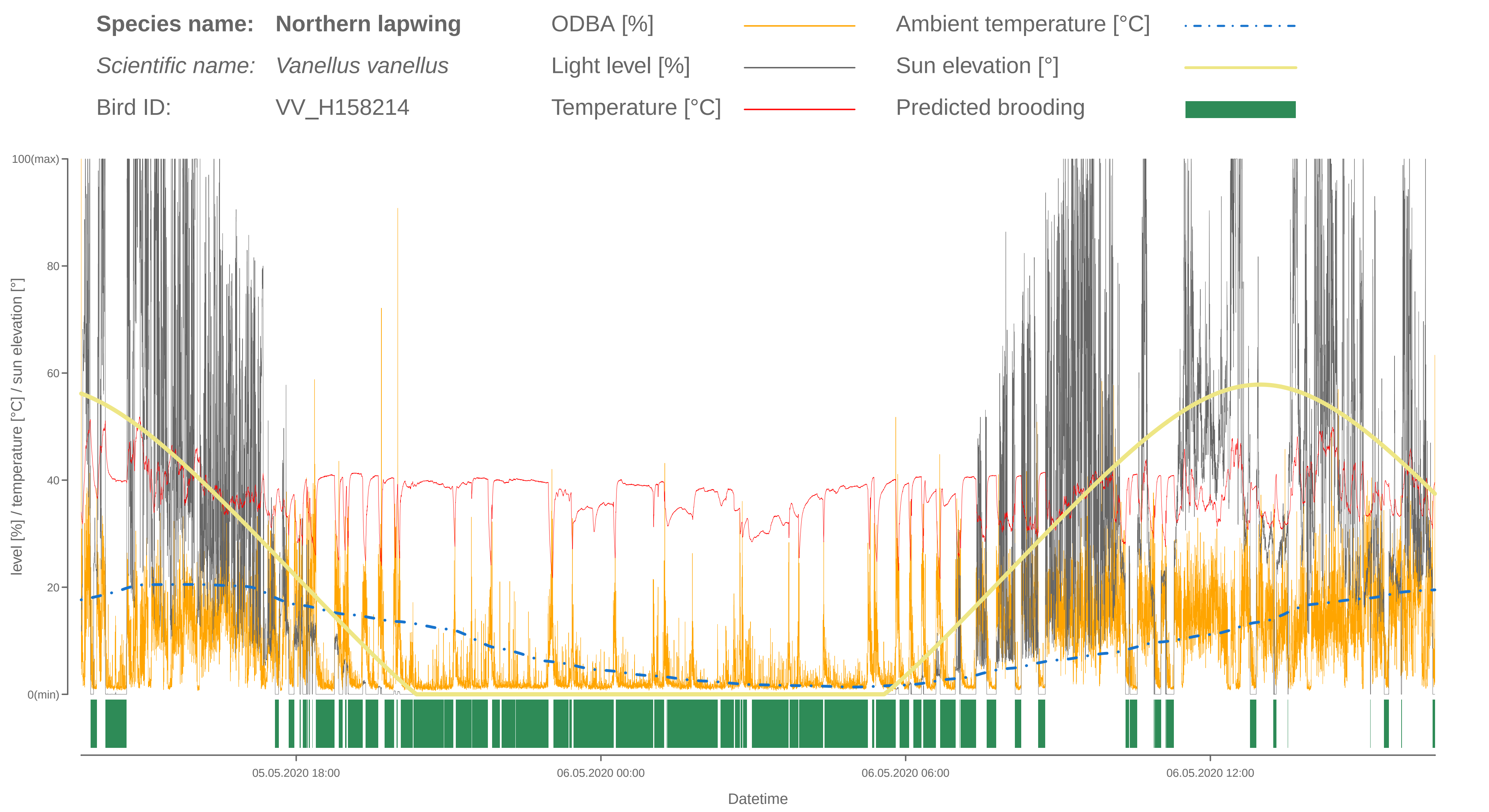
Species name: Northern lapwing
Scientific name: Vanellus vanellus
Bird ID: VV_H158214
ODBA [%]
Light level [%]
Temperature [°C]
Ambient temperature [°C]
Sun elevation [°]
Predicted brooding
100(max)
80
60
40
20
0(min)
level [%] / temperature [°C] / sun elevation [°]
05.05.2020 18:00
06.05.2020 00:00
06.05.2020 06:00
06.05.2020 12:00
Datetime

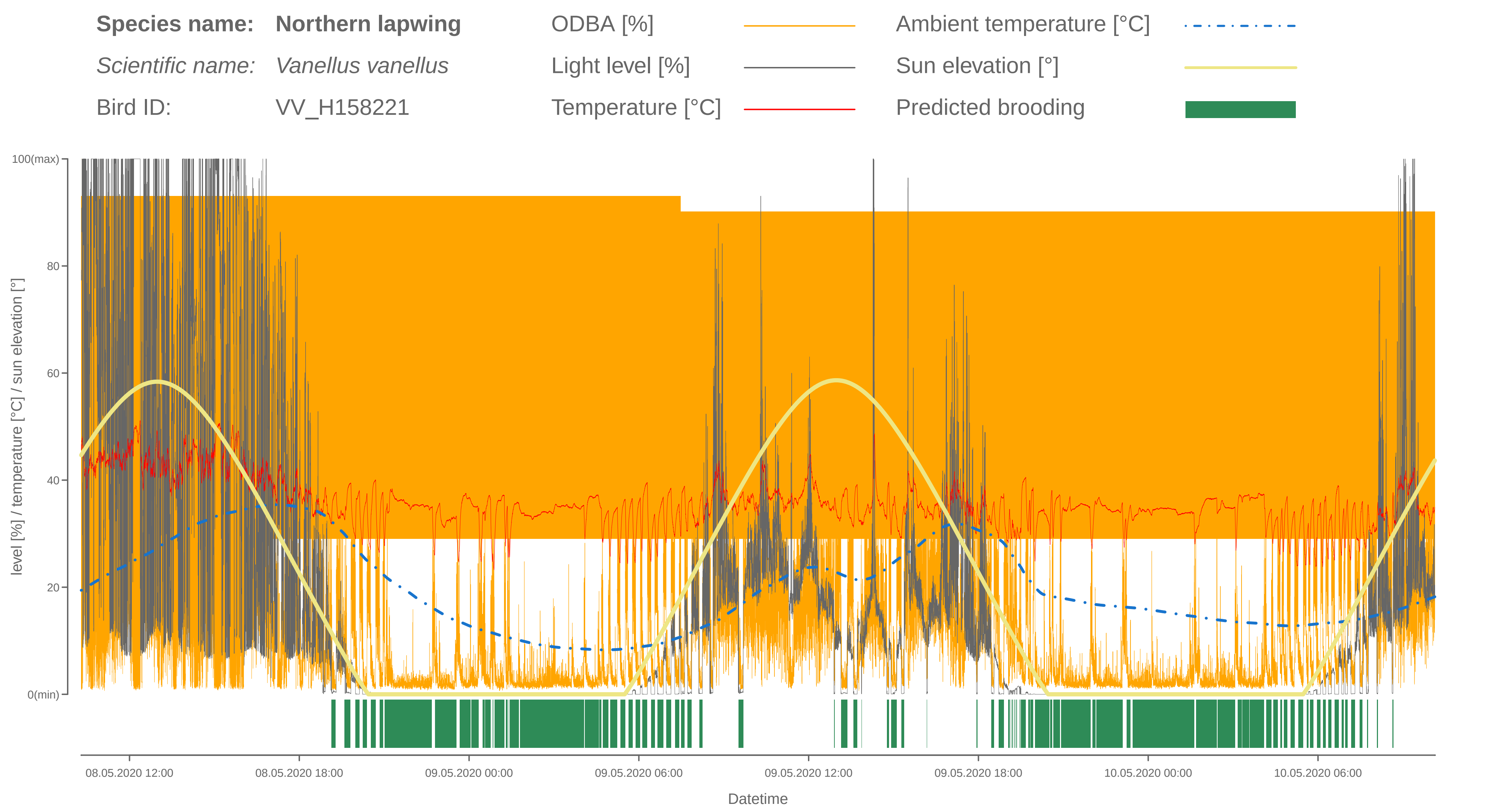

Species name: Northern lapwing
Scientific name: Vanellus vanellus
Bird ID: VV_H158221
ODBA [%]
Light level [%]
Temperature [°C]
Ambient temperature [°C]
Sun elevation [°]
Predicted brooding
100(max)
80
60
40
20
0(min)
level [%] / temperature [°C] / sun elevation [°]
08.05.2020 12:00
08.05.2020 18:00
09.05.2020 00:00
09.05.2020 06:00
09.05.2020 12:00
09.05.2020 18:00
10.05.2020 00:00
10.05.2020 06:00
Datetime

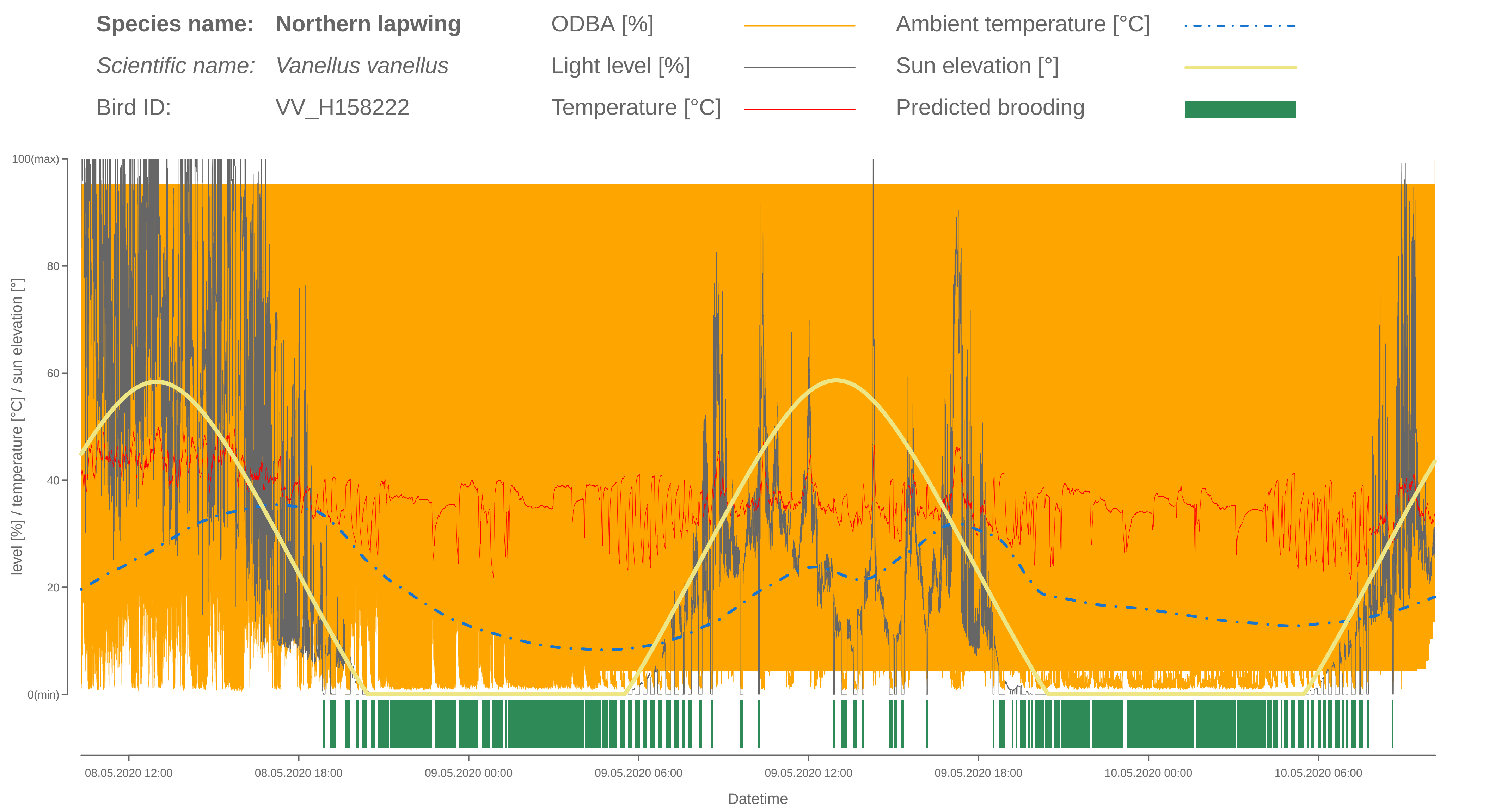

Species name: Northern lapwing
Scientific name: Vanellus vanellus
Bird ID: VV_H158222
ODBA [%]
Light level [%]
Temperature [°C]
Ambient temperature [°C]
Sun elevation [°]
Predicted brooding
100(max)
80
60
40
20
0(min)
level [%] / temperature [°C] / sun elevation [°]
08.05.2020 12:00
08.05.2020 18:00
09.05.2020 00:00
09.05.2020 06:00
09.05.2020 12:00
09.05.2020 18:00
10.05.2020 00:00
10.05.2020 06:00
Datetime

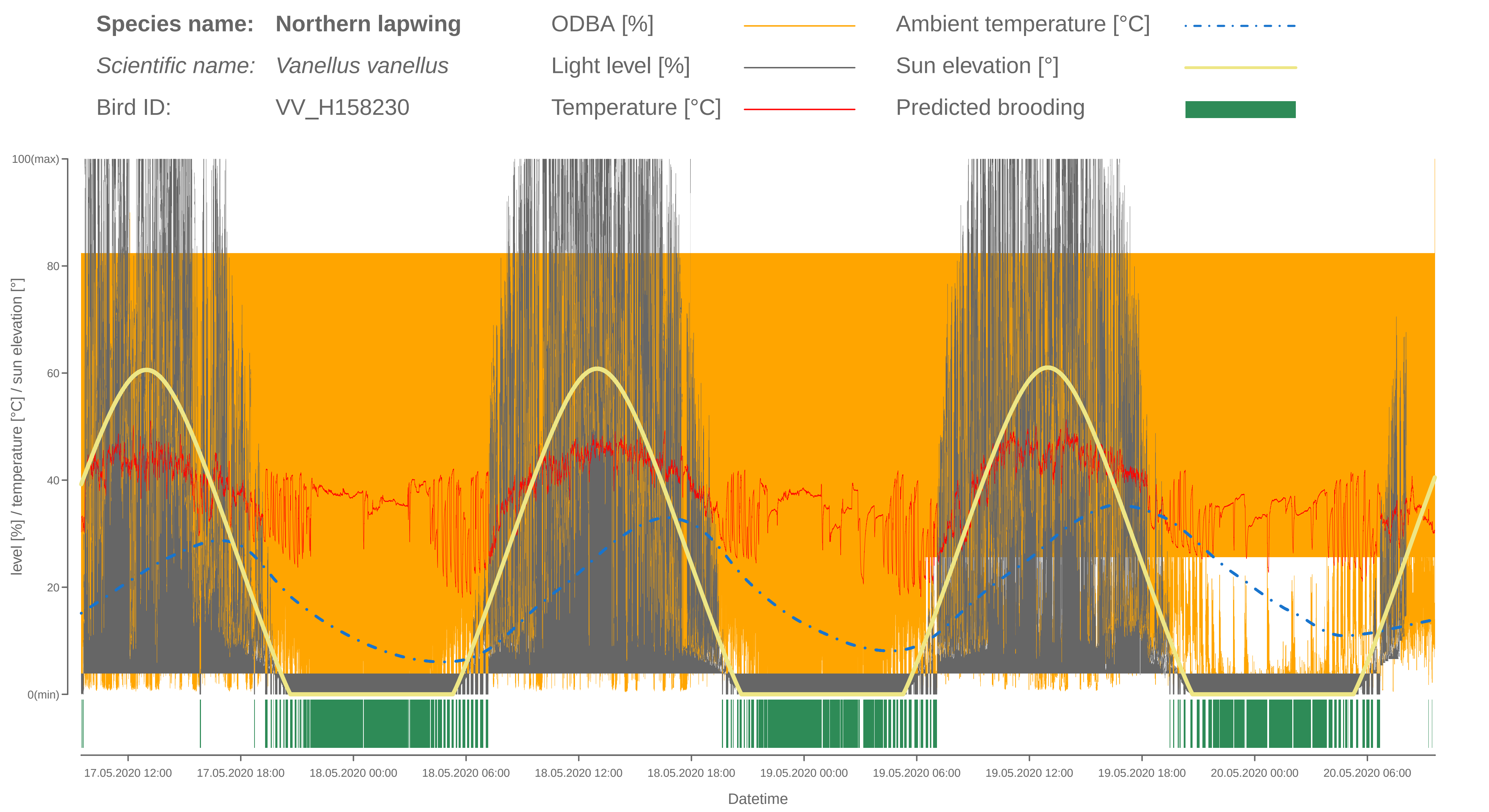

Species name: Northern lapwing
Scientific name: Vanellus vanellus
Bird ID: VV_H158230
ODBA [%]
Light level [%]
Temperature [°C]
Ambient temperature [°C]
Sun elevation [°]
Predicted brooding
100(max)
80
60
40
20
0(min)
level [%] / temperature [°C] / sun elevation [°]
17.05.2020 12:00
17.05.2020 18:00
18.05.2020 00:00
18.05.2020 06:00
18.05.2020 12:00
18.05.2020 18:00
19.05.2020 00:00
19.05.2020 06:00
19.05.2020 12:00
19.05.2020 18:00
20.05.2020 00:00
20.05.2020 06:00
Datetime

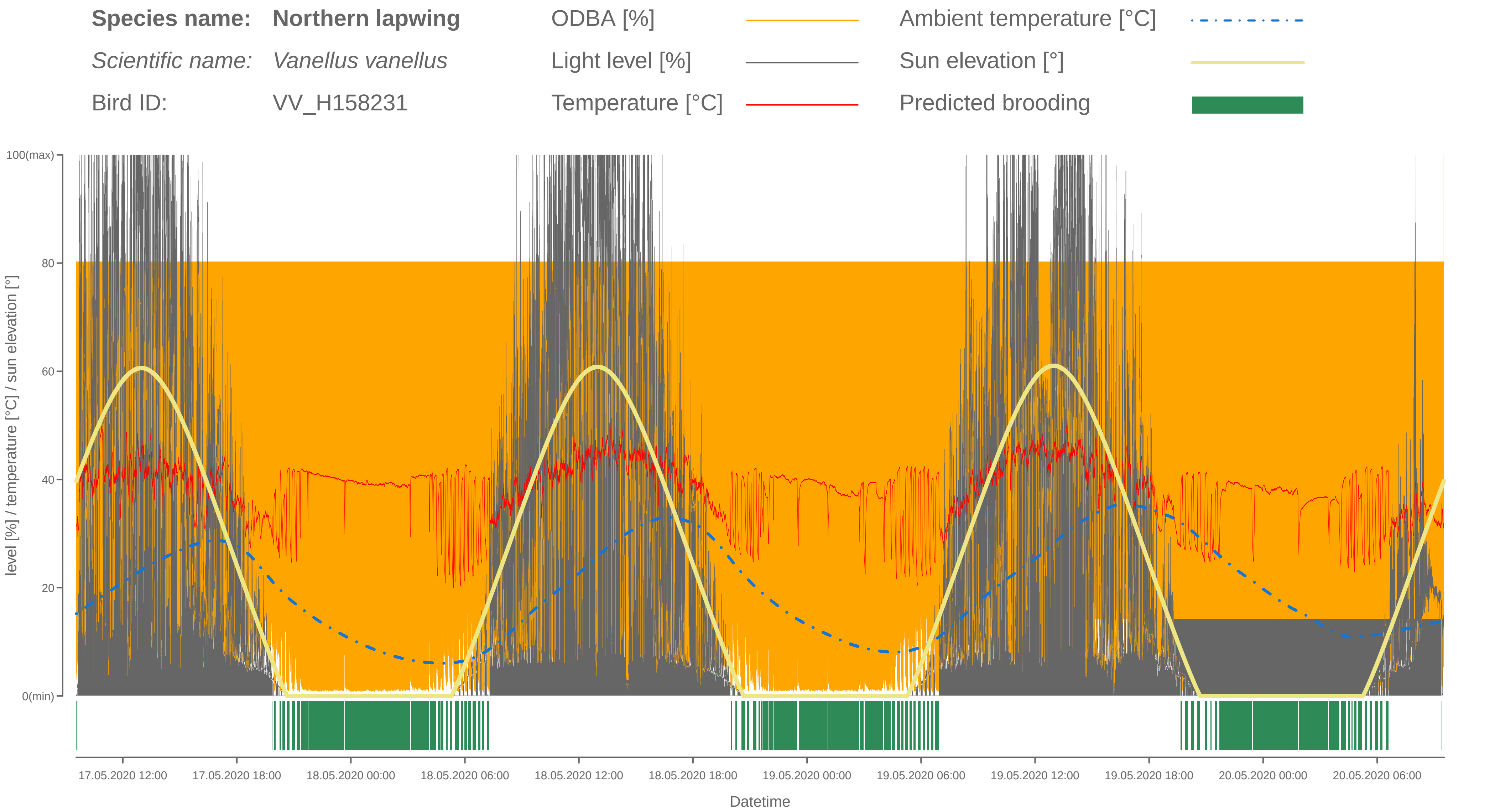
Species name: Northern lapwing
Scientific name: Vanellus vanellus
Bird ID: VV_H158231
ODBA [%]
Light level [%]
Temperature [°C]
Ambient temperature [°C]
Sun elevation [°]
Predicted brooding
100(max)
80
60
40
20
0(min)
level [%] / temperature [°C] / sun elevation [°]
17.05.2020 12:00
17.05.2020 18:00
18.05.2020 00:00
18.05.2020 06:00
18.05.2020 12:00
18.05.2020 18:00
19.05.2020 00:00
19.05.2020 06:00
19.05.2020 12:00
19.05.2020 18:00
20.05.2020 00:00
20.05.2020 06:00
Datetime

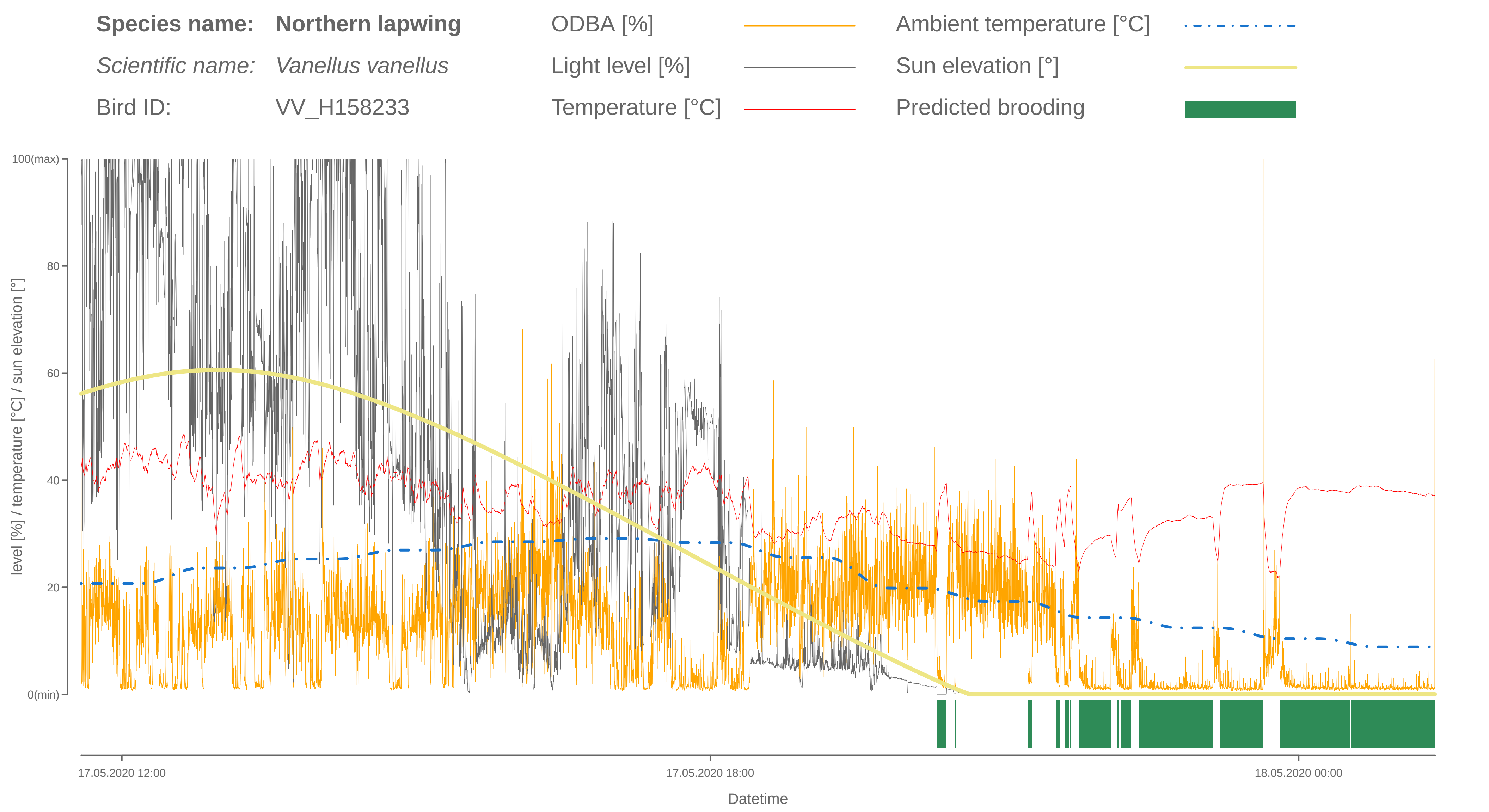
Species name: Northern lapwing
Scientific name: Vanellus vanellus
Bird ID: VV_H158233
ODBA [%]
Light level [%]
Temperature [°C]
Ambient temperature [°C]
Sun elevation [°]
Predicted brooding
100(max)
80
60
40
20
0(min)
level [%] / temperature [°C] / sun elevation [°]
17.05.2020 12:00
17.05.2020 18:00
18.05.2020 00:00
Datetime

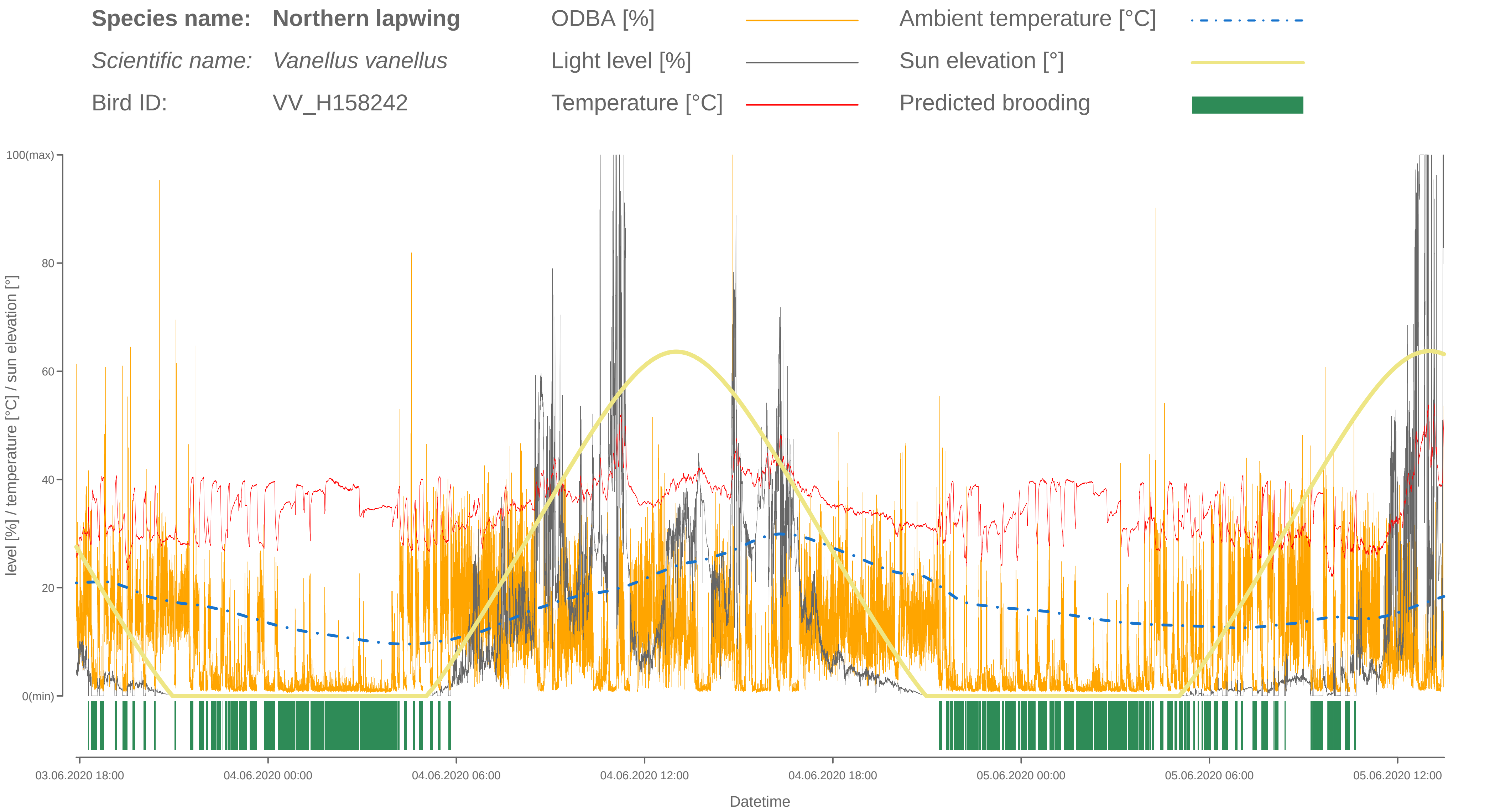

Species name: Northern lapwing
Scientific name: Vanellus vanellus
Bird ID: VV_H158242
ODBA [%]
Light level [%]
Temperature [°C]
Ambient temperature [°C]
Sun elevation [°]
Predicted brooding
100(max)
80
60
40
20
0(min)
level [%] / temperature [°C] / sun elevation [°]
03.06.2020 18:00
04.06.2020 00:00
04.06.2020 06:00
04.06.2020 12:00
04.06.2020 18:00
05.06.2020 00:00
05.06.2020 06:00
05.06.2020 12:00
Datetime

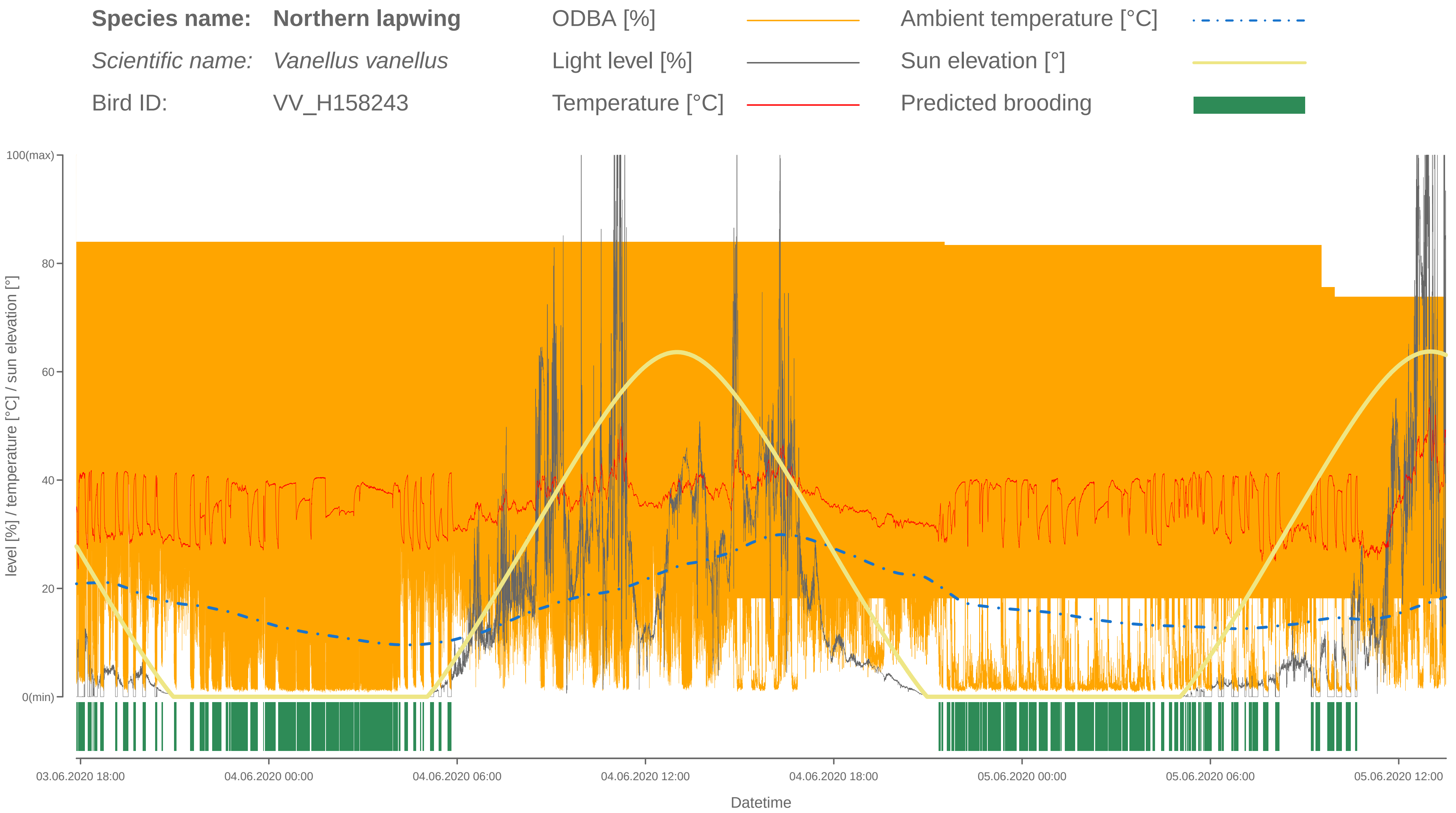

Species name: Northern lapwing
Scientific name: Vanellus vanellus
Bird ID: VV_H158243
ODBA [%]
Light level [%]
Temperature [°C]
Ambient temperature [°C]
Sun elevation [°]
Predicted brooding
100(max)
80
60
40
20
0(min)
level [%] / temperature [°C] / sun elevation [°]
03.06.2020 18:00
04.06.2020 00:00
04.06.2020 06:00
04.06.2020 12:00
04.06.2020 18:00
05.06.2020 00:00
05.06.2020 06:00
05.06.2020 12:00
Datetime
