# Supplementary material for: Offspring thermal demands and parental brooding efficiency differ for precocial birds living in contrasting climates
Source: Front Zool. 2023 Apr 10;20:12. doi: 10.1186/s12983-023-00492-1 (PMC10084700; doi:10.1186/s12983-023-00492-1)
Supplement: Supplementary file 2 — Additional file 2: Supplementary pictures: Picture S1. Illustration of the study area of the Northern lapwing, including typical habitats of species occurrence. Picture S2. Illustration of the study area of the Red-wattled lapwing, including typical habitats of species occurrence. Picture S3. Illustration of the used multisensory datalogger DAL2. Picture S4. Illustration of the deployment of the DAL 2 logger on Chicks of the Red-wattled lapwing. Picture S5. Camera footage showing the brooding of a 45-days old chick of a Red-wattled lapwing with a newly hatched chick from a subsequent breeding attempt. Supplementary methods: SM1: Procedure for obtaining reference values of light-levels in different types of natural shade. SM2: Procedure to estimate near-ground temperature from ambient temperature. [file 12983_2023_492_MOESM2_ESM.docx]

**Additional files for:**

**Offspring thermal demands and parental brooding efficiency differ for precocial birds living in contrasting climates**

**Veronika Kolešková**^1^**, Miroslav E. Šálek**^1^**, Kateřina Brynychová**^1^**,** **Petr Chajma**^1^**, Lucie Pešková**^1^**,**

**Esmat Elhassan**^1,2^**, Eva Petrusová Vozabulová** ^1^**, Veronika Janatová**^1^**, Aisha Almuhery**^2^ **& Martin Sládeček**^1^

^1^ Faculty of Environmental Sciences, Czech University of Life Sciences Prague, Kamýcká 129, 165 00 Prague, Czech Republic

^2^ Natural Resources Conservation Section, Environment Department, Dubai Municipality, Abu Hail, Dubai, United Arab Emirates


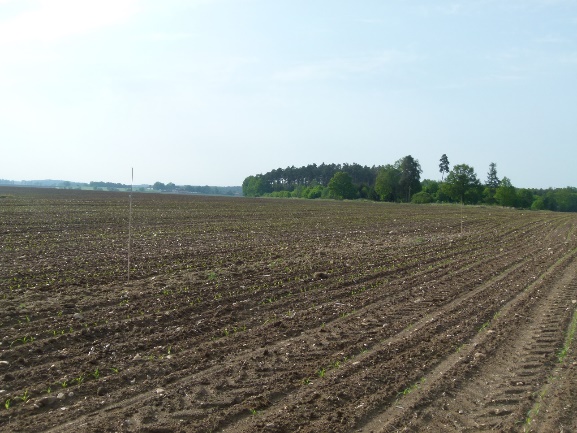

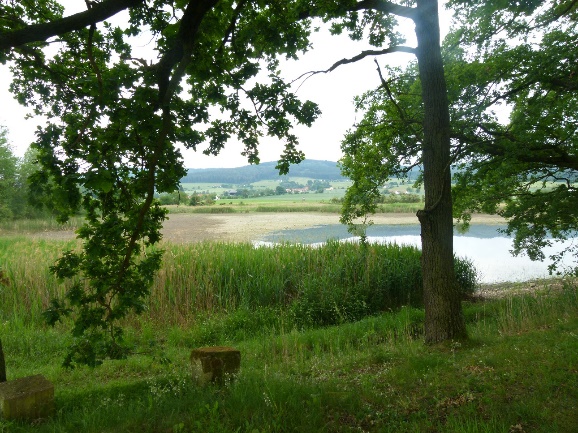

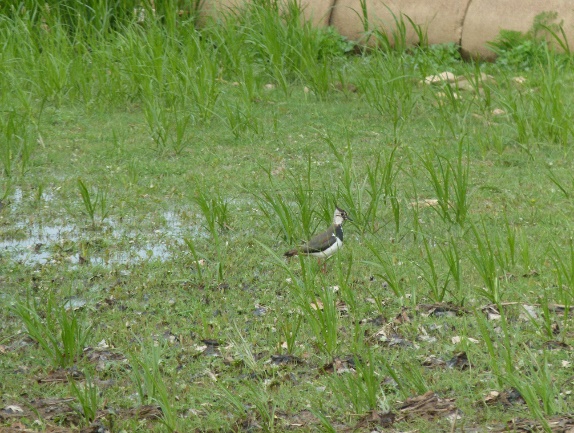
**Supplementary pictures**


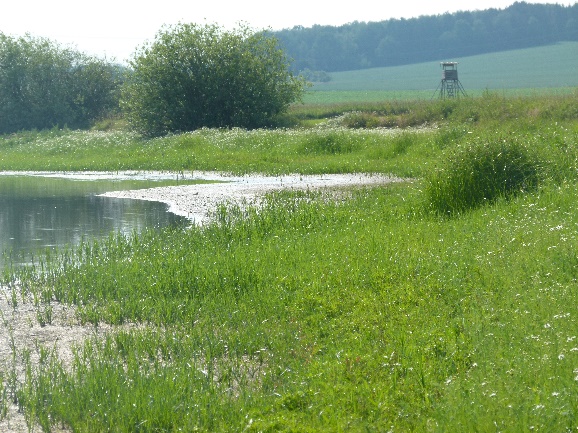


Picture S1: Illustration of the study area of the Northern lapwing, including typical habitats of species occurrence.


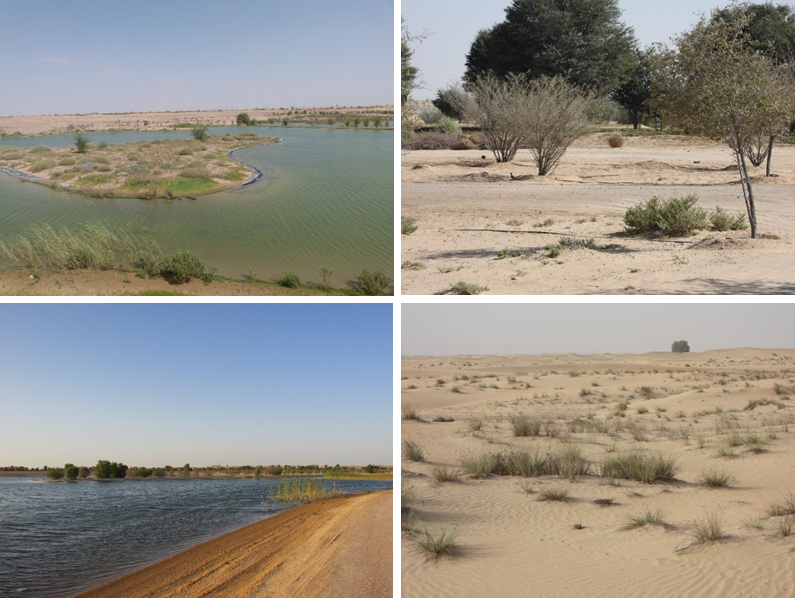


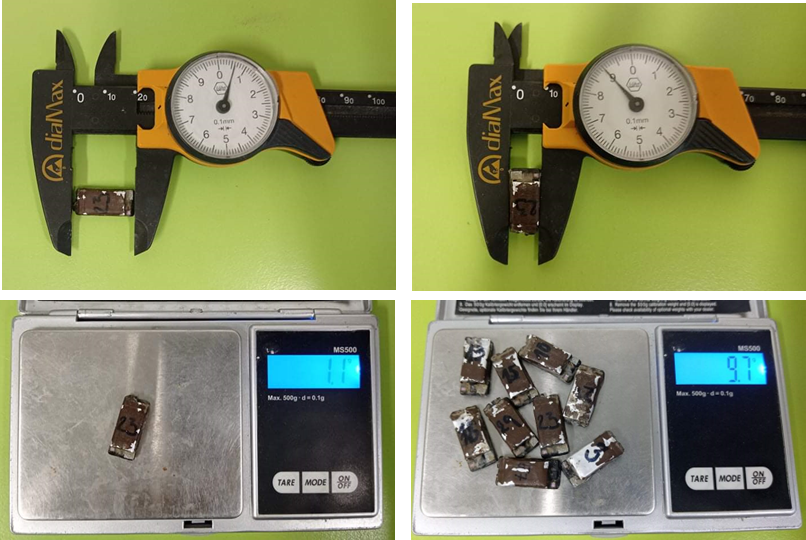


Picture S3: Illustration of the used multisensory datalogger DAL2, developed by the research team. With the size 20.6 x 19.0 x 2.5 mm and the weight 1.1g, the logger allows to measure temperature, humidity, light level, three-axial acceleration, and three-axial magnetometry at the same time, with adjustable frequencies, for approximately 72 hours of continuous recordings.

Picture S2 : Illustration of the study area of the Red-wattled lapwing, including typical habitats of species occurrence.


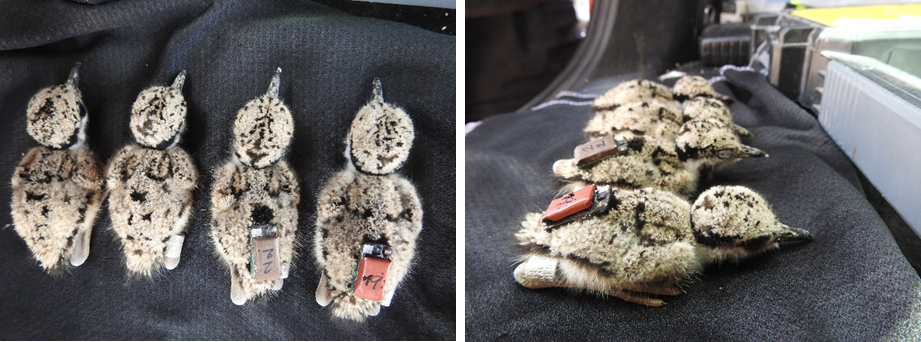


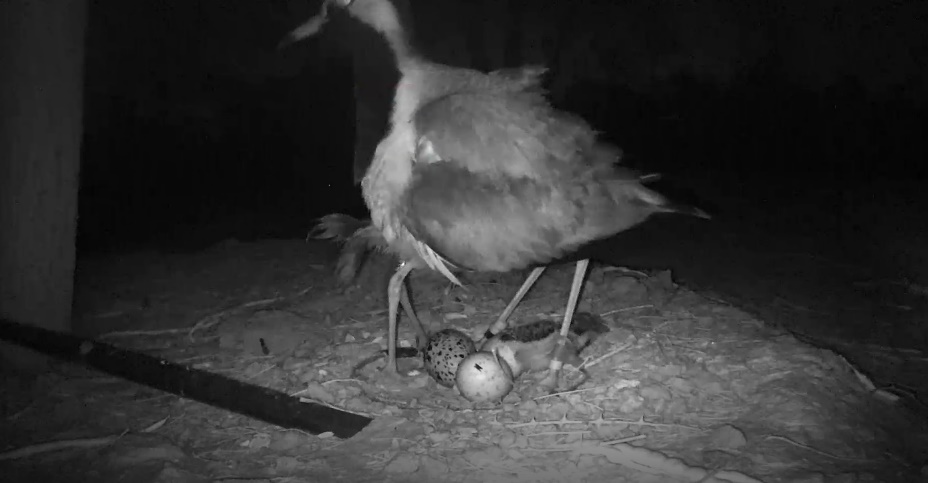
Picture S4: Illustration of the deployment of the DAL 2 logger on Chicks of the Red-wattled lapwing. The device is mounted to the down feathers in the lower back with a small drop of superglue, with a light-level sensor oriented towards the head.

Picture S5: Camera footage showing the brooding of a 45-days old chick of a Red-wattled lapwing with a newly hatched chick from a subsequent breeding attempt.


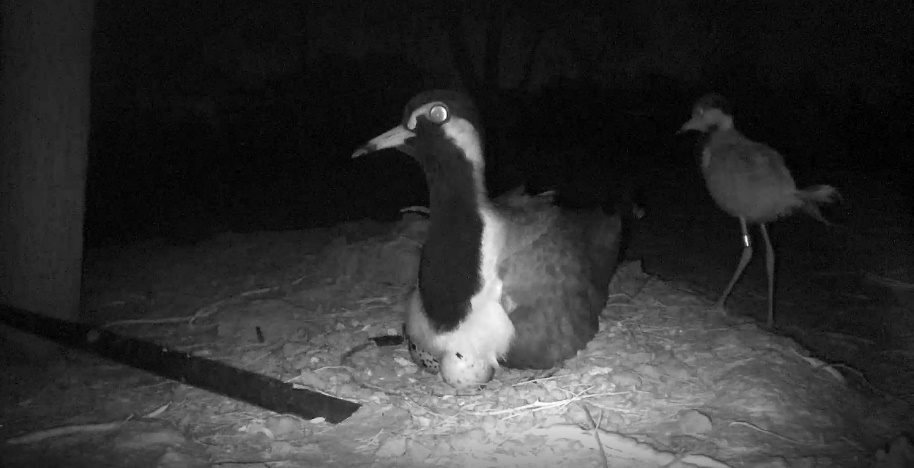


**Supplementary methods**


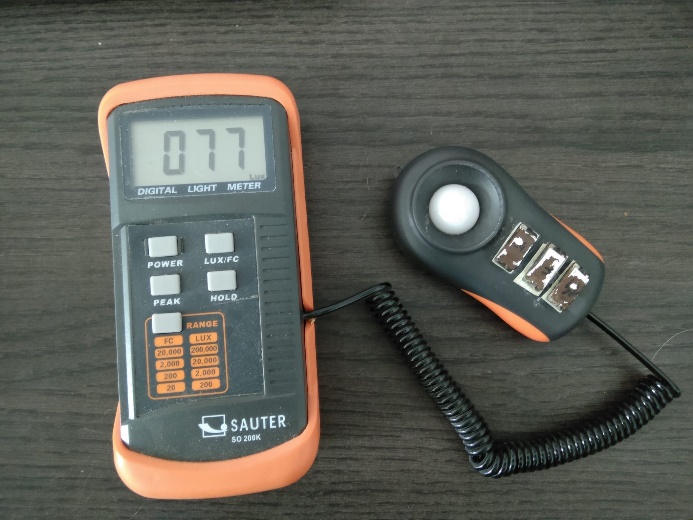
*SM1: Procedure for obtaining reference values of light-levels in different types of natural shade*

Three started DAL2 loggers were glued to the measuring device of the lux meter (Picture S7, we used dataloggers together with lux meter for another project). The assembly was exposed to light at different times and under different conditions (direct sunlight, clouds, different types of shade), leaving it at standstill for approximately one minute for each measurement. Between each measurement, the lux meter with the data loggers was left in the dark for at least a few seconds to clearly separate the measurements.

Picture S7: Installation of DAL2 loggers on the measuring device of the lux meter

This procedure was repeated 4 times (2 times in each locality), between March and June (i.e. the beginning and the end of the breeding season. Overall, 559 measurements were obtained from the dataloggers from different kinds of shade.

For each measurement, we took the median light-level. Then, we calculated the 1% quantile of the measurements taken in the shadow (~50 lux) and use it as the threshold for brooding detection during the HMM extraction post-processing.

*SM2: Procedure to estimate near-ground temperature from ambient temperature*

This supplementary material describes the process to estimate near ground temperatures (i.e. in the space where chicks are moving) from the standard meteorological measurements (i.e. two meters above the ground and in the shade).

*Datasets and sample sizes*

The hourly meteorological measurements were taken from the České Budějovice meteorological station (Czech Hydrometeorological Institute, pers. comm.) (48.95°N, 14.47°E) for the Northern lapwing population and from the Al Marmoom meteorological station (Dubai Municipality, pers. comm.) (24.84°N, 55.36°E) for the Red-wattled lapwing population. These data include temperature measurements two meters above the ground, in the shade (hereafter referred as “ambient temperature”).

The near-ground measurements were taken as a part of another project in the vicinity of the ground-nesting birds’ nests studied in both study areas, several centimetres above the ground (hereafter referred as “near-ground temperature”). All the used measurements used were taken in 2019-2020, by DHT or ZAYDA 1.1 loggers (1), both using the same temperature sensor, which is incorporated into the DAL 2 loggers. Overall, 148 dataloggers were installed in Al Marmoom and 42 in České Budějovice basin. For a particular hour, the mean temperature from all dataloggers installed at the time in the area was used. In Al Marmoom, 2527 hours with at least one installed datalogger were available, while 1132 hours were available for České Budějovice basin.

*Ground temperature predictions*

Two general linear models (each for one study area) were fitted using the 'lm' function (RCore-Team, 2019). The mean near-ground temperature obtained from dataloggers was used as a response. Included predictors were: ambient temperature (included as a 2nd degree polynomial), time of a day (transformed to radians by (2*π*time)/24 and included as a sine + cosine of radians), and relative air humidity (obtained also from meteorological stations). Models included also two-way interactions between all predictors. In order to maintain comparability, the same model was used to predict temperatures in both study areas. The coefficient of determination was high for both models: 0.91 in České Budějovice basin and 0.92 in Al Marmoom. The model estimates (Tables SX1, SX2) were then used to predict the temperatures during time periods without any direct near-ground temperature measurements.

Table SX1: Near-ground temperature estimation in the Al Marmoom, Dubai, UAE

|  |  | |  | 95% CrI | |
| --- | --- | --- | --- | --- | --- |
| Response | Effect | | Estimate | Lower | Upper |
| Near-ground temperature | **Intercept** | **28.992** | | **28.44** | **29.541** |
|  | **Temperature** | **655.857** | | **590.328** | **718.916** |
|  | Temperature^2^ | -34.503 | | -87.315 | 20.371 |
|  | **Sin (time)** | **0.264** | | **-0.306** | **0.862** |
|  | **Cos (time)** | **-8.479** | | **-9.171** | **-7.816** |
|  | **Humidity** | **0.023** | | **0.014** | **0.032** |
|  | Temperature: Sin (time) | 18.834 | | -10.954 | 48.892 |
|  | **Temperature^2:^: Sin (time)** | **-34.87** | | **-63.046** | **-7.357** |
|  | Temperature: Cos (time) | -28.016 | | -62.674 | 6.851 |
|  | **Temperature^2:^: Cos (time)** | **-34.383** | | **-66.85** | **-0.653** |
|  | **Temperature: Humidity** | **1.423** | | **0.295** | **2.537** |
|  | Temperature^2:^: Humidity | 0.516 | | -0.474 | 1.439 |
|  | Sin (time): Humidity | -0.001 | | -0.013 | 0.009 |
|  | **Cos (time): Humidity** | **0.069** | | **0.057** | **0.081** |

Table SX2: Near-ground temperature estimation in the České Budějovice basin, Czech republic

|  |  | |  | 95% CrI | |
| --- | --- | --- | --- | --- | --- |
| Response | Effect | | Estimate | Lower | Upper |
| Near-ground temperature | **Intercept** | **15.584** | | **14.791** | **16.438** |
|  | **Temperature** | **262.577** | | **233.049** | **293.467** |
|  | **Temperature^2^** | **44.9** | | **22.717** | **67.009** |
|  | **Sin (time)** | **3.602** | | **3.386** | **3.814** |
|  | **Cos (time)** | **0.964** | | **0.71** | **1.219** |
|  | Humidity | -0.008 | | -0.02 | 0.003 |
|  | **Temperature: Sin (time)** | **51.894** | | **43.997** | **59.815** |
|  | Temperature^2:^: Sin (time) | 0.552 | | -7.756 | 9.261 |
|  | Temperature: Cos (time) | -3.598 | | -13.085 | 5.685 |
|  | Temperature^2:^: Cos (time) | -8.169 | | -17.038 | 0.918 |
|  | **Temperature: Humidity** | **-0.57** | | **-1.055** | **-0.102** |
|  | **Temperature^2:^: Humidity** | **-0.442** | | **-0.79** | **-0.101** |
|  | **Sin (time): Humidity** | **15.584** | | **14.791** | **16.438** |
|  | **Cos (time): Humidity** | **262.577** | | **233.049** | **293.467** |
|  |  |  | |  |  |

**Supplementary** **references**

1. Sládeček M, Brynychová K, Elhassan E, Šálek ME, Janatová V, Vozabulová E, et al. Diel timing of nest predation changes across breeding season in a subtropical shorebird. Ecol Evol. 2021;11(19):13101–17.
